# E-Learning for Rare Diseases: An Example Using Fabry Disease

**DOI:** 10.3390/ijms18102049

**Published:** 2017-09-24

**Authors:** Chiara Cimmaruta, Ludovica Liguori, Maria Monticelli, Giuseppina Andreotti, Valentina Citro

**Affiliations:** 1 Dipartimento di Biologia, Università Federico II, 80126 Napoli, Italy; chiara.cimmaruta@unina.it (C.C.); 2.maria.monticelli@gmail.com (M.M.); vale.ctr@gmail.com (V.C.); 2 Istituto di Chimica Biomolecolare, Consiglio Nazionale delle Ricerche, Comprensorio Olivetti, Edificio 70, Via Campi Flegrei 34, 80078 Pozzuoli, Italy; lud.liguori@gmail.com; 3 Dipartimento di Scienze e Tecnologie Ambientali, Biologiche e Farmaceutiche, Università della Campania “Luigi Vanvitelli”, 81100 Caserta, Italy

**Keywords:** bioinformatics education, bioinformatics tools, rare disease, pharmacological chaperone, laboratory guide

## Abstract

Background: Rare diseases represent a challenge for physicians because patients are rarely seen, and they can manifest with symptoms similar to those of common diseases. In this work, genetic confirmation of diagnosis is derived from DNA sequencing. We present a tutorial for the molecular analysis of a rare disease using Fabry disease as an example. Methods: An exonic sequence derived from a hypothetical male patient was matched against human reference data using a genome browser. The missense mutation was identified by running BlastX, and information on the affected protein was retrieved from the database UniProt. The pathogenic nature of the mutation was assessed with PolyPhen-2. Disease-specific databases were used to assess whether the missense mutation led to a severe phenotype, and whether pharmacological therapy was an option. Results: An inexpensive bioinformatics approach is presented to get the reader acquainted with the diagnosis of Fabry disease. The reader is introduced to the field of pharmacological chaperones, a therapeutic approach that can be applied only to certain Fabry genotypes. Conclusion: The principle underlying the analysis of exome sequencing can be explained in simple terms using web applications and databases which facilitate diagnosis and therapeutic choices.

## 1. Introduction

A rare disease is any disease that affects only a small percentage of the population, occurring at a frequency of 1/2000 according to the most recent guidelines [1]. There are more than 6000 of these diseases, of which 5081 have a known phenotype description and molecular basis, while 1597 have a known phenotype description or locus with the molecular basis unknown, according to OMIM (Online Mendelian Inheritance in Men) [2]. Although these disease are individually rare, they affect a great number of people on the whole. Eighty per cent of these diseases are of genetic origin, and they are often chronic and life-threatening.

Each rare disease can have different genotypes and a large pheno-typical spectrum. Non-sense mutations, deletions, and insertions abolish the function of the affected protein, but missense mutations have variable effects that go from complete inactivation to mild reduction of activity. On average there are 10–12 missense mutations per disease, but in some cases there are hundreds. Bare figures give a sense of the great challenge represented by rare diseases both in terms of diagnosis and therapy. Understanding rare diseases at a genetic level is essential in order to search for personalized therapies for patients. Recent progress in exome sequencing and bioinformatics is helping in this challenge. We will use Fabry disease as an example to show how freely available web applications and databases can be used for diagnosis and personalized therapy.

Fabry disease (FD) (OMIM: 3015000) is a lysosomal storage disorder which is X-linked and relatively frequent, affecting 1–5 individuals per 10,000 (Orphanet: 324). Mutations of the gene encoding lysosomal α-galactosidase, with the official identifiers of the gene and the enzyme being GLA and α-galactosidase A (AGAL), respectively, cause the accumulation of the unprocessed substrate globotriaosylceramide (Gb3) and its derivatives within the lysosomes [3]. The large phenotypic and genotypic spectra of the disease represent a great challenge for clinicians. In severe cases, often referred to as classic FD, the first specific signs appearing in childhood or adolescence are angiokeratoma, cornea verticillata, neuropathic pain, acroparesthesias, and hypohidrosis. These symptoms are followed by progressive proteinuric renal insufficiency and rhythm and conductance disorders, with progressive hypertrophic cardiomyopathy and cerebrovascular stroke [4,5,6]. In mild cases, often referred to as atypical FD, patients retain some AGAL activity and are asymptomatic until adulthood, when they show only some symptoms. Residual activity of the mutant enzyme can help distinguish non-pathological, mild, and severe genotypes [7]. Since the age of onset can be late and its complications (such as cardiac manifestations, stroke and chronic renal disease) are very similar to those of other very common disorders, FD may be under-diagnosed and higher estimates have been put forward [8,9,10].

More than 700 variants have been reported for GLA so far [11] and, in contrast to other lysosomal disorders such as Gaucher’s disease [12], there are no prevalent mutations. In fact, most are usually found only in a single family. Therefore, it is not uncommon for a clinician to find a new variant. In case of a missense mutation, this represents a problem. Should the new variant be considered a benign polymorphism or a disease mutation? In the second case, does it cause severe or mild phenotype? Should the carrier be treated and with which therapy?

At present, the treatments of FD are symptomatic and life-long. So far, enzymatic replacement therapy (ERT) is the only approved therapy. This therapy involves intravenous infusions of purified recombinant AGAL every 2 weeks [13]. Early initiation of ERT, even before symptoms appear, has been suggested because irreversible organ damage, cardiac fibrosis, or severe renal dysfunction render the therapy ineffective [14,15,16]. A novel oral drug is the iminosugar-resembling galactose migalastat (1-deoxygalactonojirimycin (DGJ)). This drug is known by the commercial name of Galafold™, and is entering clinical practice [17,18,19]. It is interesting to remember that iminosugars represent a successful example of drug repositioning [20]. In fact, they were originally developed with the aim of curing HIV [21]. Clinical trials for the treatment of HIV were unsuccessful because the antiviral concentration required could not be achieved in man. Unexpectedly, it was found that imino sugars had an additional property, the inhibition of glycosphingolipid biosynthesis [22], and they acted as a pharmacological chaperone [23,24]. Pharmacological chaperones are able to stabilize some mutant forms. DGJ stabilizes AGAL, preventing early degradation by intracellular machineries and increasing their total intracellular levels [25]. Regrettably, therapy with pharmacological chaperones can be used only for some missense mutations and certainly not for those occurring in the active site of the enzyme.

In this work, on obtaining nucleotide data from a patient by exon sequencing, the reader will become familiar with several databases and bioinformatics tools and will be lead to formulate a diagnosis and a therapeutic proposal.

## 2. Results

The case is of an adolescent male patient who is affected by angiokeratoma and mild proteinurea. His parents are apparently healthy. There is a suspicion of Fabry disease and, if the diagnosis is confirmed, the clinician should decide whether it is a classic (severe) or an atypical (mild) form and whether a therapy should be started before other symptoms appear. In this case, a choice would need to be made between two types of intervention: ERT, or pharmacological chaperones.

Confirmation of the diagnosis can be carried out through sequencing the exons of the *GLA* gene or those of a restricted panel of genes that are associated with the symptoms of the patient. The appropriate assays can be found by searching by condition/phenotypes in the genetic testing registry (https://www.ncbi.nlm.nih.gov/gtr/tests/). This tutorial starts from a short sequence of DNA that could be derived from next-generation sequencing.

**Figure 1:**
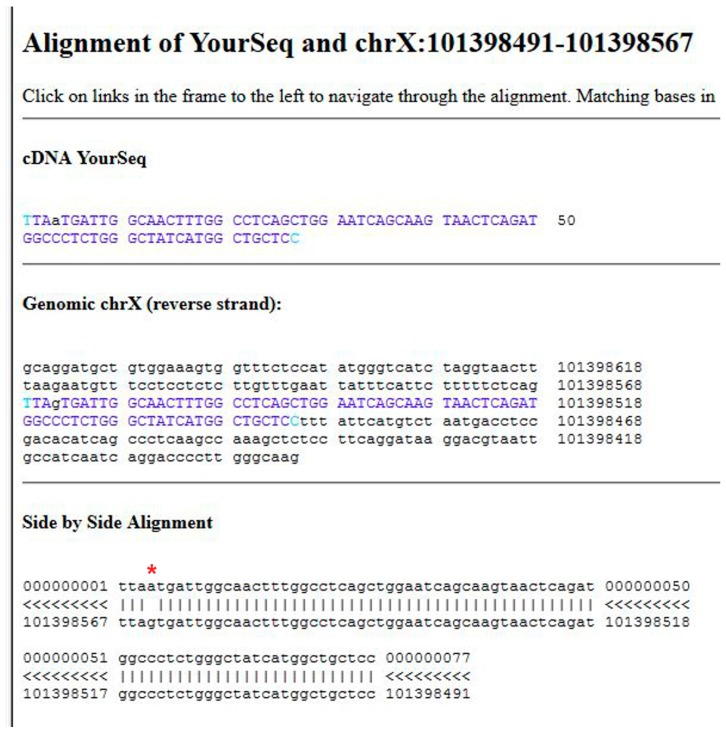
Alignment of the patient’s sequence with the reference genome using BLAT. The transition observed in the *GLA* gene is indicated by an asterisk.

**Figure 2:**
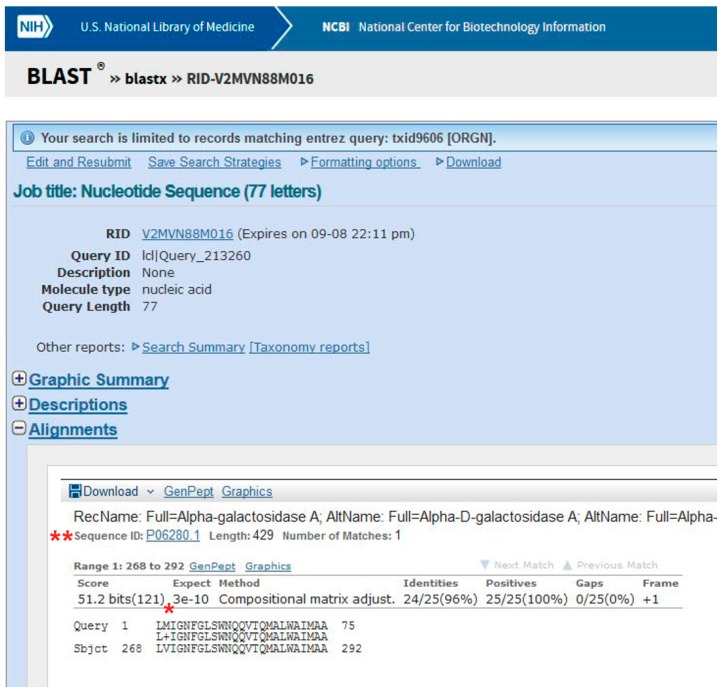
Alignment of the patient’s sequence with the wild-type protein sequence using BLAST. A missense mutation in α-galactosidase A (AGAL) was identified (*). The sequence ID is also highlighted (**).

Data obtained following the tutorial are presented in the methods section. In brief, a variant is found mapping the exonic sequences derived from the patient on the reference human genome using the program BLAT. Most variants are not associated with disease. Usually it is assumed that if a nucleotide change results in a synonymous codon, it is benign. Therefore, it is necessary to understand whether the mutation affects the protein product. Indeed, the transition observed in the GLA gene of the patient, G>A (Figure 1), generates a missense mutation in AGAL, p.V269M (Figure 2).

**Figure 3:**
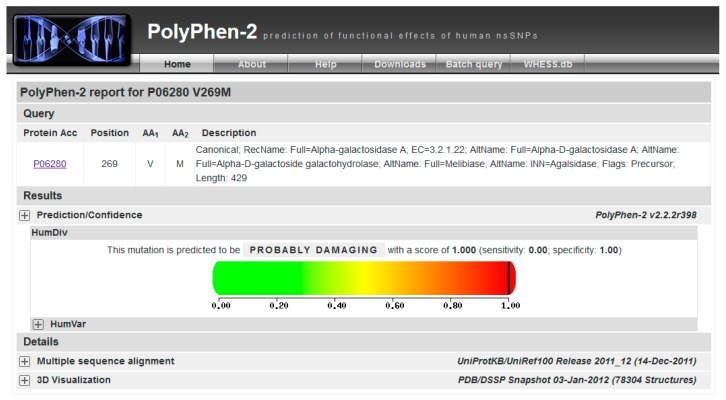
PolyPhen-2 graphical output.

This mutation is not reported in UniProt (“Pathology & Biotech” section), or ExAC (a database for alternative allele frequencies [26]), nor is it reported in OMIM® [2] and ClinVar [27]). It is predicted as being pathogenic according to PolyPhen-2 [28] (Figure 3). p.V269M might represent a novel case.

Although purposely simplified, the example presented so far illustrates the generic pipeline that is followed for the analysis of a disease mutation. In order to go into the diagnosis in more depth and personalize the therapy, more must be learnt about the affected protein and the specific disease.

UniProt is a manually curated and annotated protein database that can be searched with the accession code provided by BlastX, P06280.1. Scrolling the UniProt page one can learn that:

(1) Alpha-galactosidase A (AGAL) is encoded by the gene *GLA* (in the header of the file).
(2) AGAL has catalytic activity: “Hydrolysis of terminal, non-reducing α-d-galactose residues in α-d-galactosides, including galactose oligosaccharides, galactomannans and galactolipids.” (in the “Function” section).
(3) AGAL is involved in a disease, Fabry disease (FD). A link to OMIM [2] (301500) provides clinical information about the disease (“Pathology & Biotech: Involvement in disease” section).
(4) AGAL has a pharmaceutical use: “Available under the names Replagal^®^ (from Shire) and Fabrazyme^®^ (from Genzyme). Used as a long-term enzyme replacement therapy in patients with a confirmed diagnosis of Fabry disease. The differences between Replagal^®^ (also known as agalsidase alpha) and Fabrazyme^®^ (also known as agalsidase beta) lie in the glycosylation patterns. Agalsidase alpha is produced in the hamster CHO cell line while agalsidase alpha is produced in human cell lines.” (“Pathology & Biotech: Pharmaceutical use” section).
(5) Another therapy is available for FD. A link to DrugBank (“Pathology & Biotech: Chemistry databases” section) [29] (DB05018) provides some details about the drug and summarizes its mechanism of action: “migalastat hydrochloride is an experimental, oral therapy for the treatment of Fabry disease and belongs to a class of molecules known as pharmacological chaperones”. Indeed, migalastat hydrochloride or 1-deoxygalactonojirimycin (DGJ) is a pharmacological chaperone for FD; it stabilizes wild type AGAL as well as some mutant forms. Mutations affecting the active site or cysteines involved in disulphide bridge formation do not respond. These conditions are necessary, but not sufficient to exclude the usefulness of migalastat [30]. In general, each mutation must be experimentally tested; the techniques needed for analysis have been extensively described elsewhere [31,32,33] but are outside the scope of this tutorial.

**Figure 4:**
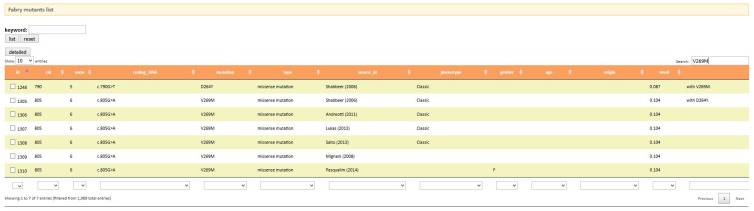
Output of fabry-database.org.

Once the reader has been introduced to the disease and has become acquainted with the main genetic and biochemical aspects, he can move onto disease-specific databases by looking for them in PubMed. Two references point to on-line user-friendly databases. On searching fabry-database.org (Figure 4) the reader learns that this missense mutation in AGAL p.V269M has already been reported in the literature [34,35,36] and is associated to the classic phenotype, thus confirming the prediction of PolyPhen-2. The mutation is also annotated [37] in the manually-curated database of disease-associated variants HGMD (The Human Gene Mutation Database) [11]. It should be remembered that when a new variant, which is not included in any of the data bases of clinical phenotype, is found, it is advisable to perform a biopsy to obtain a definitive diagnosis [38].

**Figure 5:**
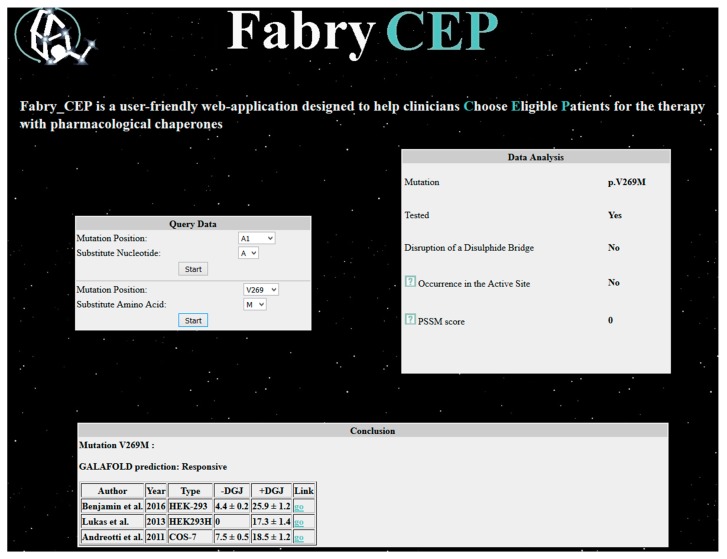
Output of Fabry_CEP.

Fabry_CEP is a specialized database that reports data found in the literature concerning the residual activity in cells of each possible AGAL mutant with or without the pharmacological chaperone DGJ [39]. References are provided and, in case no experimental data are available, the probability of being responsive is provided. When Fabry_CEP is queried with the mutation p.V269M, it returns experimental results (Figure 5), numerical values of activity with standard deviation obtained by three independent groups [32,40,41], and information provided by the group that commercializes migalastat under the registered name of Galafold^®^. Experimental conditions are slightly different, but in all cases the mutation is responsive to the drug. The estimate of the residual activity of the mutant enzyme in cells is low and confirms that the phenotype can be classic. Besides these data obtained from the literature, the reader will learn that the mutation does not occur in the active site (this result was obtained running the program DrosteP [42] on the X-ray structure of AGAL).

## 3. Discussion

The tutorial we presented shows how a variant found in a patient can be critically evaluated to graduate diagnosis and personalize the therapy. We chose FD as an example, but the approach is not limited to this disease. Some emerging questions were raised. In the first place, the clinician could encounter a variant that is not (yet) among disease mutations in the most frequently consulted databases. This can occur either because the variant is new or because it has been described in medical literature, but has not yet been included. This problem can be solved by predicting the association with disease and/or looking for disease-specific databases. In addition to this, the clinician should check whether the mutation is associated to a severe phenotype and if a mutation-specific therapy exists. FD represents a successful example of the use of pharmacological chaperones. This approach, which is definitely limited to a subset of missense mutations, is not limited to FD, and is being assessed with respect to other lysosomal [43,44] and metabolic disorders [45,46,47,48] as well.

The diagnosis of rare diseases takes advantage of the sequence of the DNA of the patient alone or of the so-called trios in which data from parents are obtained too. The analysis can be extended to the whole genome or exome, or limited to a panel of genes or to the exons of a single gene. The huge amount of data, particularly in the case of genome or exome sequencing, requires the help of experts who can run pipelines of specific dedicated software. Yet, in the end, when the number of candidate variants is restricted, it is up to the clinician to make a diagnosis critically and choose the therapy. We have shown that this is possible because user-friendly web applications and databases can be used without specific bioinformatics training.

## 4. Methods

### 4.1. Aims

The reader will get familiar with databases and programs that are used during exome sequencing analysis and with disease-specific tools. Only a basic knowledge of genetics and biochemistry is required. The tutorial will start from the results of an analysis of the DNA of a hypothetical male patient. It will proceed with variant calling, identification of the type of mutation, and prediction of its pathogenic nature. Information about the affected protein and potential therapies will be gained.

### 4.2. Requirements

It is an in silico experience and only a computer with an internet connection is required.

A list of bioinformatics tools which do not require registration and have the advantages of enabling fast, low-cost, and reliable analysis of biological data with user-friendly interfaces is provided.

UCSC Genome Browser, https://genome.ucsc.edu/
BLAST, https://blast.ncbi.nlm.nih.gov/Blast.cgi
UniProt, http://www.uniprot.org/
PolyPhen-2, http://genetics.bwh.harvard.edu/pph2/ [28]
PubMed, https://www.ncbi.nlm.nih.gov/pubmed
fabry-database, http://fabry-database.org/ [49]
Fabry_CEP, http://www1.na.icb.cnr.it/project/fabry_cep/ [39]. This tool can be run on-line or by locally downloading the supplementary material. Please download, unzip and press on index.html icon.

### 4.3. Input Exonic Sequence

We assume that the sequence has been obtained from a male patient:

ttaatgattggcaactttggcctcagctggaatcagcaagtaactcagatggccctctgggctatcatggctgctcc

### 4.4. Protocol

Step 1. Variant Calling

The nucleotide sequence will be mapped on the human reference genome.

**Figure 6:**
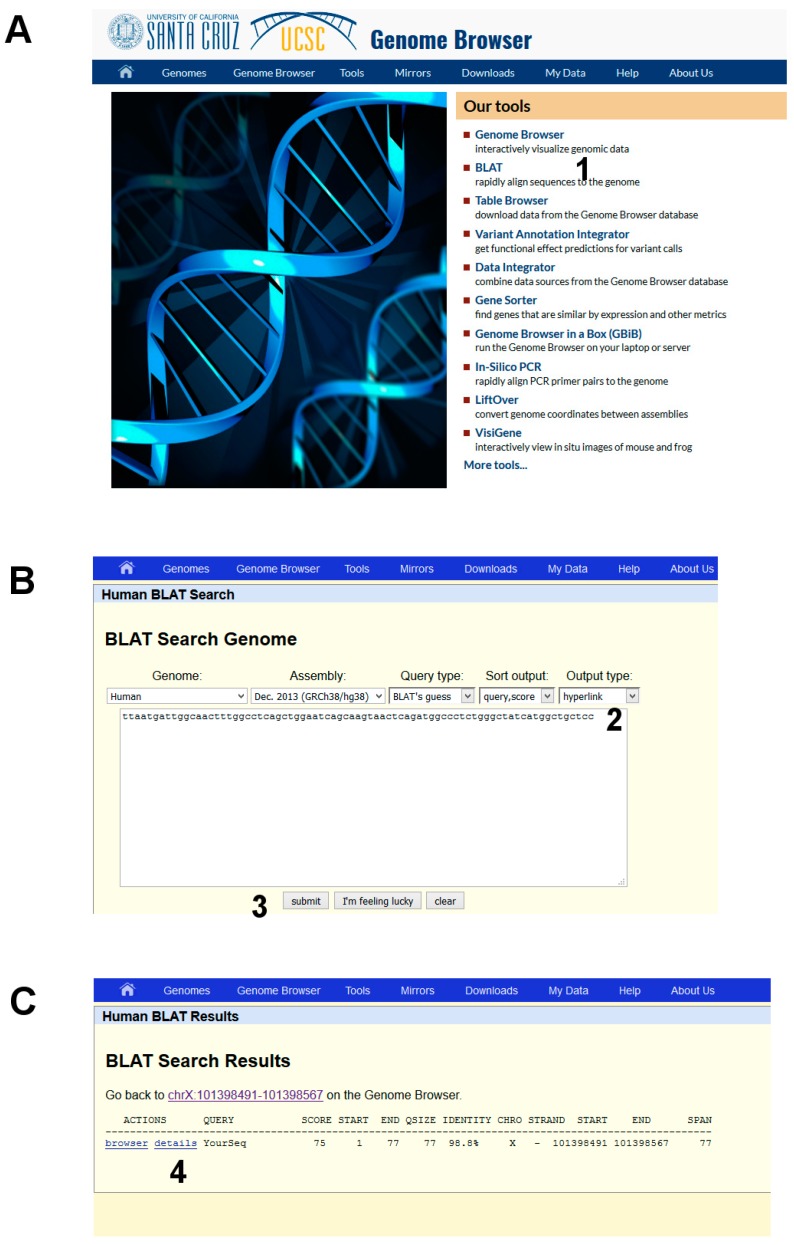
DNA analysis by using BLAT in the UCSC Genome Browser. (**A**) starting BLAT; (**B**) input data; and (**C**) list of significant hits.

(1) Open the UCSC genome browser and choose among the BLAT tools (Figure 6A, point 1).
(2) The latest assembly of the HUMAN genome is chosen by default and does not need to be changed. An overview of how the program BLAT works is offered in the search page. Paste the given sequence into the Query Sequence box (Figure 6B, point 2).
(3) Submit (Figure 6B, point 3).

The output is a list of significant hits. The highest score is obtained mapping the sequence on the X chromosome. Clicking on “details” (Figure 6C, point 4) a side-by-side alignment of the patient’s sequence with the reference genome is obtained. A transition A>G is observed (Figure 1).

Step 2. Is it a missense, nonsense, or a synonymous mutation?

The sequence will be translated to check in the protein database UniProt whether the mutation has an effect on the gene product.

**Figure 7:**
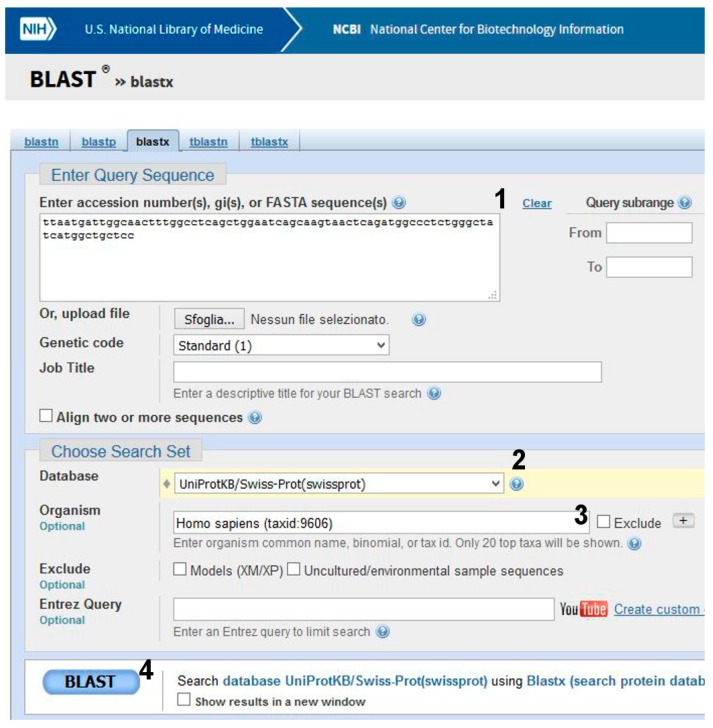
DNA analysis by using BlastX.

(1) Go to BLAST and choose BlastX. The program searches protein databases using a translated nucleotide query.
(2) Enter the patient’s sequence in the query box (Figure 7, point 1), choose UniProt as a database (Figure 7, point 2), and *Homo sapiens* as the species (Figure 7, point 3).
(3) Click on BLAST (Figure 7, point 4).

Be patient! When you get results, scroll the page. The best alignment is obtained with α-galactosidase A, Uniprot Sequence ID: P06280.1 (Figure 2). One amino acid (V269) in the subject found in UniProt is substituted by M in the query (i.e., the patient’s) sequence.

Step 3. Obtaining information about the protein affected by the mutation

**Figure 8:**
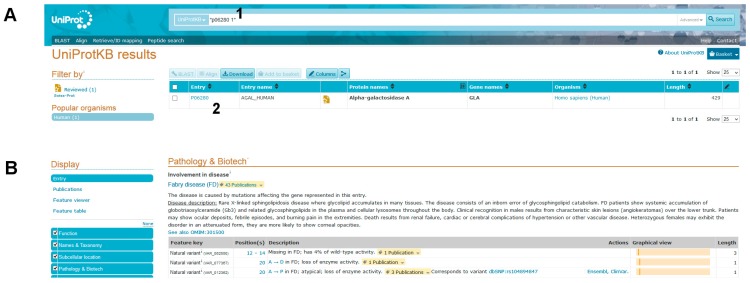
UniProt analysis. (**A**) input data. (**B**) a selection of the output, a list of natural variants of AGAL (partial view).

(1) Query Uniprot using the ID of the target protein found with BlastX: P06280.1 (Figure 8A, point1).

You will get the entry name AGAL_HUMAN and you should click on link besides it (Figure 8A, point 2).

Many details on the protein will appear: Function, Names & Taxonomy, Subcellular location, and so on. A long list of natural variants is reported in the Pathology & Biotech section, most of which are implicated in FD (Figure 8B). Among them 269 V to M (p.V269M) is not found. Links to OMIM (300644. gene; 301500. phenotype) can be followed to read about the disease and its pathological variants. Another popular site is ClinVar https://www.ncbi.nlm.nih.gov/clinvar/. It can be searched with “*GLA* AND V269M”, but in this database also the variant p.V269M cannot be found.

Step 4. Is the variant pathological?

The variant carried from the patient, p.V269M, is not in the list reported by UniProt, MIM or ClinVar. It might be a new disease mutation. You can run predictive programs such as PolyPhen-2.

**Figure 9:**
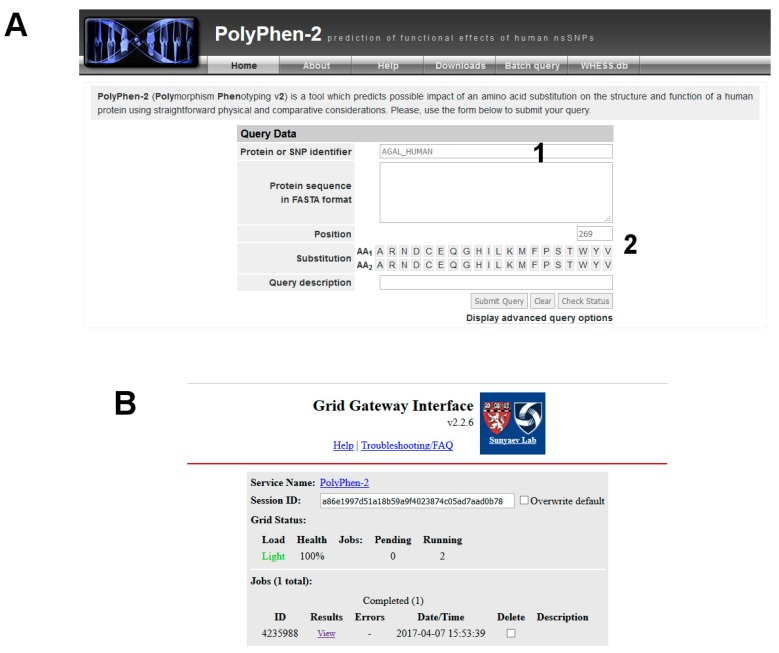
PolyPhen-2 analysis. (**A**) input data and (**B**) result status.

(1) Launch the program inserting the entry name of the protein, “AGAL_HUMAN” (Figure 9A, point 1), the site of the mutation 269, the wild-type amino acid, V, and the mutated one, M (Figure 9A, point 2).

Be patient! Then you can check the result by clicking on View (Figure 3).

Step 5. Do specific databases exist? Does the mutation cause severe inactivation of AGAL? Does it respond to DGJ?

PubMed can be searched with the keywords “Fabry AND Database AND User friendly”; fabry-database and FABRY_CEP are disease specific and require only the introduction of the missense mutation.

## Supplementary Materials

Supplementary File 1

## Abbreviations

FD	Fabry disease
Gb3	Globotriaosylceramide
ERT	Enzymatic replacement therapy
DGJ	1-Deoxygalactonojirimycin

## References

[1] Orphanet. http://www.Orpha.Net/consor/cgi-bin/index.Php.

[2] Online Mendelian Inheritance in Man, O Mckusick-Nathans Institute of Genetic Medicine, Johns Hopkins University (Baltimore, MD). https://omim.Org/.

[3] Germain D.P. (2010). Fabry disease. Orphanet J. Rare Dis..

[4] Linhart A., Elliott P.M. (2007). The heart in anderson-fabry disease and other lysosomal storage disorders. Heart.

[5] Schiffmann R., Moore D.F. (2006). Neurological manifestations of fabry disease. Fabry Disease: Perspectives from 5 Years of FOS.

[6] Sunder-Plassmann G. (2006). Renal manifestations of fabry disease. Fabry Disease: Perspectives from 5 Years of FOS.

[7] Citro V., Cammisa M., Liguori L., Cimmaruta C., Lukas J., Cubellis M.V., Andreotti G. (2016). The large phenotypic spectrum of fabry disease requires graduated diagnosis and personalized therapy: A meta-analysis can help to differentiate missense mutations. Int. J. Mol. Sci..

[8] Spada M., Pagliardini S., Yasuda M., Tukel T., Thiagarajan G., Sakuraba H., Ponzone A., Desnick R.J. (2006). High incidence of later-onset fabry disease revealed by newborn screening. Am. J. Hum. Genet..

[9] Scott C.R., Elliott S., Buroker N., Thomas L.I., Keutzer J., Glass M., Gelb M.H., Turecek F. (2013). Identification of infants at risk for developing Fabry, Pompe, or mucopolysaccharidosis-I from newborn blood spots by tandem mass spectrometry. J. Pediatr..

[10] Hopkins P.V., Campbell C., Klug T., Rogers S., Raburn-Miller J., Kiesling J. (2015). Lysosomal storage disorder screening implementation: Findings from the first six months of full population pilot testing in missouri. J. Pediatr..

[11] HGMD. http://www.Hgmd.Cf.Ac.Uk/.

[12] Zimran A., Gelbart T., Westwood B., Grabowski G.A., Beutler E. (1991). High frequency of the gaucher disease mutation at nucleotide 1226 among ashkenazi jews. Am. J. Hum. Genet..

[13] Biegstraaten M., Arngrimsson R., Barbey F., Boks L., Cecchi F., Deegan P.B., Feldt-Rasmussen U., Geberhiwot T., Germain D.P., Hendriksz C. (2015). Recommendations for initiation and cessation of enzyme replacement therapy in patients with fabry disease: The european fabry working group consensus document. Orphanet J. Rare Dis..

[14] Germain D.P., Waldek S., Banikazemi M., Bushinsky D.A., Charrow J., Desnick R.J., Lee P., Loew T., Vedder A.C., Abichandani R. (2007). Sustained, long-term renal stabilization after 54 months of agalsidase beta therapy in patients with fabry disease. J. Am. Soc. Nephrol..

[15] Rombach S.M., Smid B.E., Bouwman M.G., Linthorst G.E., Dijkgraaf M.G., Hollak C.E. (2013). Long term enzyme replacement therapy for fabry disease: Effectiveness on kidney, heart and brain. Orphanet J. Rare Dis..

[16] Weidemann F., Niemann M., Stork S., Breunig F., Beer M., Sommer C., Herrmann S., Ertl G., Wanner C. (2013). Long-term outcome of enzyme-replacement therapy in advanced fabry disease: Evidence for disease progression towards serious complications. J. Intern. Med..

[17] Germain D.P., Hughes D.A., Nicholls K., Bichet D.G., Giugliani R., Wilcox W.R., Feliciani C., Shankar S.P., Ezgu F., Amartino H. (2016). Treatment of fabry’s disease with the pharmacologic chaperone migalastat. N. Engl. J. Med..

[18] Hughes D.A., Nicholls K., Shankar S.P., Sunder-Plassmann G., Koeller D., Nedd K., Vockley G., Hamazaki T., Lachmann R., Ohashi T. (2016). Oral pharmacological chaperone migalastat compared with enzyme replacement therapy in fabry disease: 18-month results from the randomised phase III attract study. J. Med. Genet..

[19] Markham A. (2016). Migalastat: First global approval. Drugs.

[20] Hay Mele B., Citro V., Andreotti G., Cubellis M.V. (2015). Drug repositioning can accelerate discovery of pharmacological chaperones. Orphanet J. Rare Dis..

[21] Tierney M., Pottage J., Kessler H., Fischl M., Richman D., Merigan T., Powderly W., Smith S., Karim A., Sherman J. (1995). The tolerability and pharmacokinetics of N-butyl-deoxynojirimycin in patients with advanced hiv disease (ACTG 100). The aids clinical trials group (ACTG) of the national institute of allergy and infectious diseases. J. Acquir. Immune Defic. Syndr. Hum. Retrovirol..

[22] Butters T.D. (2007). Pharmacotherapeutic strategies using small molecules for the treatment of glycolipid lysosomal storage disorders. Expert Opin. Pharmacother..

[23] Lieberman R.L., D’Aquino J.A., Ringe D., Petsko G.A. (2009). Effects of pH and iminosugar pharmacological chaperones on lysosomal glycosidase structure and stability. Biochemistry.

[24] Nash R.J., Kato A., Yu C.Y., Fleet G.W. (2011). Iminosugars as therapeutic agents: Recent advances and promising trends. Future Med. Chem..

[25] Fan J.Q., Ishii S., Asano N., Suzuki Y. (1999). Accelerated transport and maturation of lysosomal alpha-galactosidase a in fabry lymphoblasts by an enzyme inhibitor. Nat. Med..

[26] Lek M., Karczewski K.J., Minikel E.V., Samocha K.E., Banks E., Fennell T., O’Donnell-Luria A.H., Ware J.S., Hill A.J., Cummings B.B. (2016). Analysis of protein-coding genetic variation in 60,706 humans. Nature.

[27] Landrum M.J., Lee J.M., Benson M., Brown G., Chao C., Chitipiralla S., Gu B., Hart J., Hoffman D., Hoover J. (2016). Clinvar: Public archive of interpretations of clinically relevant variants. Nucleic Acids Res..

[28] Adzhubei I.A., Schmidt S., Peshkin L., Ramensky V.E., Gerasimova A., Bork P., Kondrashov A.S., Sunyaev S.R. (2010). A method and server for predicting damaging missense mutations. Nat. Methods.

[29] Wishart D.S., Knox C., Guo A.C., Shrivastava S., Hassanali M., Stothard P., Chang Z., Woolsey J. (2006). Drugbank: A comprehensive resource for in silico drug discovery and exploration. Nucleic Acids Res..

[30] Andreotti G., Guarracino M.R., Cammisa M., Correra A., Cubellis M.V. (2010). Prediction of the responsiveness to pharmacological chaperones: Lysosomal human alpha-galactosidase, a case of study. Orphanet J. Rare Dis..

[31] Shin M.H., Lim H.S. (2017). Screening methods for identifying pharmacological chaperones. Mol. Biosyst..

[32] Benjamin E.R., Della Valle M.C., Wu X., Katz E., Pruthi F., Bond S., Bronfin B., Williams H., Yu J., Bichet D.G. (2016). The validation of pharmacogenetics for the identification of fabry patients to be treated with migalastat. Genet. Med..

[33] Andreotti G., Citro V., Correra A., Cubellis M.V. (2014). A thermodynamic assay to test pharmacological chaperones for fabry disease. Biochim. Biophys. Acta.

[34] Shabbeer J., Yasuda M., Benson S.D., Desnick R.J. (2006). Fabry disease: Identification of 50 novel alpha-galactosidase a mutations causing the classic phenotype and three-dimensional structural analysis of 29 missense mutations. Hum. Genom..

[35] Mignani R., Feriozzi S., Pisani A., Cioni A., Comotti C., Cossu M., Foschi A., Giudicissi A., Gotti E., Lozupone V.A. (2008). Agalsidase therapy in patients with fabry disease on renal replacement therapy: A nationwide study in italy. Nephrol. Dial. Transplant..

[36] Pasqualim G., Simon L., Sperb-Ludwig F., Burin M.G., Michelin-Tirelli K., Giugliani R., Matte U. (2014). Fabry disease: A new approach for the screening of females in high-risk groups. Clin. Biochem..

[37] Von Scheidt W., Eng C.M., Fitzmaurice T.F., Erdmann E., Hubner G., Olsen E.G., Christomanou H., Kandolf R., Bishop D.F., Desnick R.J. (1991). An atypical variant of fabry’s disease with manifestations confined to the myocardium. N. Engl. J. Med..

[38] Schiffmann R., Fuller M., Clarke L.A., Aerts J.M. (2016). Is it fabry disease?. Genet. Med..

[39] Cammisa M., Correra A., Andreotti G., Cubellis M.V. (2013). Fabry_cep: A tool to identify fabry mutations responsive to pharmacological chaperones. Orphanet J. Rare Dis..

[40] Andreotti G., Citro V., De Crescenzo A., Orlando P., Cammisa M., Correra A., Cubellis M.V. (2011). Therapy of fabry disease with pharmacological chaperones: From in silico predictions to in vitro tests. Orphanet J. Rare Dis..

[41] Lukas J., Giese A.K., Markoff A., Grittner U., Kolodny E., Mascher H., Lackner K.J., Meyer W., Wree P., Saviouk V. (2013). Functional characterisation of alpha-galactosidase a mutations as a basis for a new classification system in fabry disease. PLoS Genet..

[42] Cammisa M., Correra A., Andreotti G., Cubellis M.V. (2013). Identification and analysis of conserved pockets on protein surfaces. BMC Bioinform..

[43] Parenti G., Andria G., Valenzano K.J. (2015). Pharmacological chaperone therapy: Preclinical development, clinical translation, and prospects for the treatment of lysosomal storage disorders. Mol. Ther..

[44] Graziano A.C., Pannuzzo G., Avola R., Cardile V. (2016). Chaperones as potential therapeutics for krabbe disease. J. Neurosci. Res..

[45] Henriques B.J., Lucas T.G., Gomes C.M. (2016). Therapeutic approaches using riboflavin in mitochondrial energy metabolism disorders. Curr. Drug Targets.

[46] Hole M., Jorge-Finnigan A., Underhaug J., Teigen K., Martinez A. (2016). Pharmacological chaperones that protect tetrahydrobiopterin dependent aromatic amino acid hydroxylases through different mechanisms. Curr. Drug Targets.

[47] Banning A., Gulec C., Rouvinen J., Gray S.J., Tikkanen R. (2016). Identification of small molecule compounds for pharmacological chaperone therapy of aspartylglucosaminuria. Sci. Rep..

[48] Santos-Sierra S., Kirchmair J., Perna A.M., Reiss D., Kemter K., Roschinger W., Glossmann H., Gersting S.W., Muntau A.C., Wolber G. (2012). Novel pharmacological chaperones that correct phenylketonuria in mice. Hum. Mol. Genet..

[49] Saito S., Ohno K., Sakuraba H. (2011). Fabry-database.Org: Database of the clinical phenotypes, genotypes and mutant alpha-galactosidase a structures in fabry disease. J. Hum. Genet..

