# Supplementary material for: E-Learning for Rare Diseases: An Example Using Fabry Disease
Source: Int J Mol Sci. 2017 Sep 24;18(10):2049. doi: 10.3390/ijms18102049 (PMC5666731; doi:10.3390/ijms18102049)
Supplement: Supplementary File 1 [file ijms-18-02049-s001.zip › sito_fabry_2.3/index.html]

 
 
 
 Fabry CEP 
  


 


function controllo1()
{


var sequenza_nucleotidica = ['A', 'T', 'G', 'C', 'A', 'G', 'C', 'T', 'G', 'A', 'G', 'G', 'A', 'A', 'C', 'C', 'C', 'A', 'G', 'A', 'A', 'C', 'T', 'A', 'C', 'A', 'T', 'C', 'T', 'G', 'G', 'G', 'C', 'T', 'G', 'C', 'G', 'C', 'G', 'C', 'T', 'T', 'G', 'C', 'G', 'C', 'T', 'T', 'C', 'G', 'C', 'T', 'T', 'C', 'C', 'T', 'G', 'G', 'C', 'C', 'C', 'T', 'C', 'G', 'T', 'T', 'T', 'C', 'C', 'T', 'G', 'G', 'G', 'A', 'C', 'A', 'T', 'C', 'C', 'C', 'T', 'G', 'G', 'G', 'G', 'C', 'T', 'A', 'G', 'A', 'G', 'C', 'A', 'C', 'T', 'G', 'G', 'A', 'C', 'A', 'A', 'T', 'G', 'G', 'A', 'T', 'T', 'G', 'G', 'C', 'A', 'A', 'G', 'G', 'A', 'C', 'G', 'C', 'C', 'T', 'A', 'C', 'C', 'A', 'T', 'G', 'G', 'G', 'C', 'T', 'G', 'G', 'C', 'T', 'G', 'C', 'A', 'C', 'T', 'G', 'G', 'G', 'A', 'G', 'C', 'G', 'C', 'T', 'T', 'C', 'A', 'T', 'G', 'T', 'G', 'C', 'A', 'A', 'C', 'C', 'T', 'T', 'G', 'A', 'C', 'T', 'G', 'C', 'C', 'A', 'G', 'G', 'A', 'A', 'G', 'A', 'G', 'C', 'C', 'A', 'G', 'A', 'T', 'T', 'C', 'C', 'T', 'G', 'C', 'A', 'T', 'C', 'A', 'G', 'T', 'G', 'A', 'G', 'A', 'A', 'G', 'C', 'T', 'C', 'T', 'T', 'C', 'A', 'T', 'G', 'G', 'A', 'G', 'A', 'T', 'G', 'G', 'C', 'A', 'G', 'A', 'G', 'C', 'T', 'C', 'A', 'T', 'G', 'G', 'T', 'C', 'T', 'C', 'A', 'G', 'A', 'A', 'G', 'G', 'C', 'T', 'G', 'G', 'A', 'A', 'G', 'G', 'A', 'T', 'G', 'C', 'A', 'G', 'G', 'T', 'T', 'A', 'T', 'G', 'A', 'G', 'T', 'A', 'C', 'C', 'T', 'C', 'T', 'G', 'C', 'A', 'T', 'T', 'G', 'A', 'T', 'G', 'A', 'C', 'T', 'G', 'T', 'T', 'G', 'G', 'A', 'T', 'G', 'G', 'C', 'T', 'C', 'C', 'C', 'C', 'A', 'A', 'A', 'G', 'A', 'G', 'A', 'T', 'T', 'C', 'A', 'G', 'A', 'A', 'G', 'G', 'C', 'A', 'G', 'A', 'C', 'T', 'T', 'C', 'A', 'G', 'G', 'C', 'A', 'G', 'A', 'C', 'C', 'C', 'T', 'C', 'A', 'G', 'C', 'G', 'C', 'T', 'T', 'T', 'C', 'C', 'T', 'C', 'A', 'T', 'G', 'G', 'G', 'A', 'T', 'T', 'C', 'G', 'C', 'C', 'A', 'G', 'C', 'T', 'A', 'G', 'C', 'T', 'A', 'A', 'T', 'T', 'A', 'T', 'G', 'T', 'T', 'C', 'A', 'C', 'A', 'G', 'C', 'A', 'A', 'A', 'G', 'G', 'A', 'C', 'T', 'G', 'A', 'A', 'G', 'C', 'T', 'A', 'G', 'G', 'G', 'A', 'T', 'T', 'T', 'A', 'T', 'G', 'C', 'A', 'G', 'A', 'T', 'G', 'T', 'T', 'G', 'G', 'A', 'A', 'A', 'T', 'A', 'A', 'A', 'A', 'C', 'C', 'T', 'G', 'C', 'G', 'C', 'A', 'G', 'G', 'C', 'T', 'T', 'C', 'C', 'C', 'T', 'G', 'G', 'G', 'A', 'G', 'T', 'T', 'T', 'T', 'G', 'G', 'A', 'T', 'A', 'C', 'T', 'A', 'C', 'G', 'A', 'C', 'A', 'T', 'T', 'G', 'A', 'T', 'G', 'C', 'C', 'C', 'A', 'G', 'A', 'C', 'C', 'T', 'T', 'T', 'G', 'C', 'T', 'G', 'A', 'C', 'T', 'G', 'G', 'G', 'G', 'A', 'G', 'T', 'A', 'G', 'A', 'T', 'C', 'T', 'G', 'C', 'T', 'A', 'A', 'A', 'A', 'T', 'T', 'T', 'G', 'A', 'T', 'G', 'G', 'T', 'T', 'G', 'T', 'T', 'A', 'C', 'T', 'G', 'T', 'G', 'A', 'C', 'A', 'G', 'T', 'T', 'T', 'G', 'G', 'A', 'A', 'A', 'A', 'T', 'T', 'T', 'G', 'G', 'C', 'A', 'G', 'A', 'T', 'G', 'G', 'T', 'T', 'A', 'T', 'A', 'A', 'G', 'C', 'A', 'C', 'A', 'T', 'G', 'T', 'C', 'C', 'T', 'T', 'G', 'G', 'C', 'C', 'C', 'T', 'G', 'A', 'A', 'T', 'A', 'G', 'G', 'A', 'C', 'T', 'G', 'G', 'C', 'A', 'G', 'A', 'A', 'G', 'C', 'A', 'T', 'T', 'G', 'T', 'G', 'T', 'A', 'C', 'T', 'C', 'C', 'T', 'G', 'T', 'G', 'A', 'G', 'T', 'G', 'G', 'C', 'C', 'T', 'C', 'T', 'T', 'T', 'A', 'T', 'A', 'T', 'G', 'T', 'G', 'G', 'C', 'C', 'C', 'T', 'T', 'T', 'C', 'A', 'A', 'A', 'A', 'G', 'C', 'C', 'C', 'A', 'A', 'T', 'T', 'A', 'T', 'A', 'C', 'A', 'G', 'A', 'A', 'A', 'T', 'C', 'C', 'G', 'A', 'C', 'A', 'G', 'T', 'A', 'C', 'T', 'G', 'C', 'A', 'A', 'T', 'C', 'A', 'C', 'T', 'G', 'G', 'C', 'G', 'A', 'A', 'A', 'T', 'T', 'T', 'T', 'G', 'C', 'T', 'G', 'A', 'C', 'A', 'T', 'T', 'G', 'A', 'T', 'G', 'A', 'T', 'T', 'C', 'C', 'T', 'G', 'G', 'A', 'A', 'A', 'A', 'G', 'T', 'A', 'T', 'A', 'A', 'A', 'G', 'A', 'G', 'T', 'A', 'T', 'C', 'T', 'T', 'G', 'G', 'A', 'C', 'T', 'G', 'G', 'A', 'C', 'A', 'T', 'C', 'T', 'T', 'T', 'T', 'A', 'A', 'C', 'C', 'A', 'G', 'G', 'A', 'G', 'A', 'G', 'A', 'A', 'T', 'T', 'G', 'T', 'T', 'G', 'A', 'T', 'G', 'T', 'T', 'G', 'C', 'T', 'G', 'G', 'A', 'C', 'C', 'A', 'G', 'G', 'G', 'G', 'G', 'T', 'T', 'G', 'G', 'A', 'A', 'T', 'G', 'A', 'C', 'C', 'C', 'A', 'G', 'A', 'T', 'A', 'T', 'G', 'T', 'T', 'A', 'G', 'T', 'G', 'A', 'T', 'T', 'G', 'G', 'C', 'A', 'A', 'C', 'T', 'T', 'T', 'G', 'G', 'C', 'C', 'T', 'C', 'A', 'G', 'C', 'T', 'G', 'G', 'A', 'A', 'T', 'C', 'A', 'G', 'C', 'A', 'A', 'G', 'T', 'A', 'A', 'C', 'T', 'C', 'A', 'G', 'A', 'T', 'G', 'G', 'C', 'C', 'C', 'T', 'C', 'T', 'G', 'G', 'G', 'C', 'T', 'A', 'T', 'C', 'A', 'T', 'G', 'G', 'C', 'T', 'G', 'C', 'T', 'C', 'C', 'T', 'T', 'T', 'A', 'T', 'T', 'C', 'A', 'T', 'G', 'T', 'C', 'T', 'A', 'A', 'T', 'G', 'A', 'C', 'C', 'T', 'C', 'C', 'G', 'A', 'C', 'A', 'C', 'A', 'T', 'C', 'A', 'G', 'C', 'C', 'C', 'T', 'C', 'A', 'A', 'G', 'C', 'C', 'A', 'A', 'A', 'G', 'C', 'T', 'C', 'T', 'C', 'C', 'T', 'T', 'C', 'A', 'G', 'G', 'A', 'T', 'A', 'A', 'G', 'G', 'A', 'C', 'G', 'T', 'A', 'A', 'T', 'T', 'G', 'C', 'C', 'A', 'T', 'C', 'A', 'A', 'T', 'C', 'A', 'G', 'G', 'A', 'C', 'C', 'C', 'C', 'T', 'T', 'G', 'G', 'G', 'C', 'A', 'A', 'G', 'C', 'A', 'A', 'G', 'G', 'G', 'T', 'A', 'C', 'C', 'A', 'G', 'C', 'T', 'T', 'A', 'G', 'A', 'C', 'A', 'G', 'G', 'G', 'A', 'G', 'A', 'C', 'A', 'A', 'C', 'T', 'T', 'T', 'G', 'A', 'A', 'G', 'T', 'G', 'T', 'G', 'G', 'G', 'A', 'A', 'C', 'G', 'A', 'C', 'C', 'T', 'C', 'T', 'C', 'T', 'C', 'A', 'G', 'G', 'C', 'T', 'T', 'A', 'G', 'C', 'C', 'T', 'G', 'G', 'G', 'C', 'T', 'G', 'T', 'A', 'G', 'C', 'T', 'A', 'T', 'G', 'A', 'T', 'A', 'A', 'A', 'C', 'C', 'G', 'G', 'C', 'A', 'G', 'G', 'A', 'G', 'A', 'T', 'T', 'G', 'G', 'T', 'G', 'G', 'A', 'C', 'C', 'T', 'C', 'G', 'C', 'T', 'C', 'T', 'T', 'A', 'T', 'A', 'C', 'C', 'A', 'T', 'C', 'G', 'C', 'A', 'G', 'T', 'T', 'G', 'C', 'T', 'T', 'C', 'C', 'C', 'T', 'G', 'G', 'G', 'T', 'A', 'A', 'A', 'G', 'G', 'A', 'G', 'T', 'G', 'G', 'C', 'C', 'T', 'G', 'T', 'A', 'A', 'T', 'C', 'C', 'T', 'G', 'C', 'C', 'T', 'G', 'C', 'T', 'T', 'C', 'A', 'T', 'C', 'A', 'C', 'A', 'C', 'A', 'G', 'C', 'T', 'C', 'C', 'T', 'C', 'C', 'C', 'T', 'G', 'T', 'G', 'A', 'A', 'A', 'A', 'G', 'G', 'A', 'A', 'G', 'C', 'T', 'A', 'G', 'G', 'G', 'T', 'T', 'C', 'T', 'A', 'T', 'G', 'A', 'A', 'T', 'G', 'G', 'A', 'C', 'T', 'T', 'C', 'A', 'A', 'G', 'G', 'T', 'T', 'A', 'A', 'G', 'A', 'A', 'G', 'T', 'C', 'A', 'C', 'A', 'T', 'A', 'A', 'A', 'T', 'C', 'C', 'C', 'A', 'C', 'A', 'G', 'G', 'C', 'A', 'C', 'T', 'G', 'T', 'T', 'T', 'T', 'G', 'C', 'T', 'T', 'C', 'A', 'G', 'C', 'T', 'A', 'G', 'A', 'A', 'A', 'A', 'T', 'A', 'C', 'A', 'A', 'T', 'G', 'C', 'A', 'G', 'A', 'T', 'G', 'T', 'C', 'A', 'T', 'T', 'A', 'A', 'A', 'A', 'G', 'A', 'C', 'T', 'T', 'A', 'C', 'T', 'T'];
var sequenza = "MQLRNPELHLGCALALRFLALVSWDIPGARALDNGLARTPTMGWLHWERFMCNLDCQEEPDSCISEKLFMEMAELMVSEGWKDAGYEYLCIDDCWMAPQRDSEGRLQADPQRFPHGIRQLANYVHSKGLKLGIYADVGNKTCAGFPGSFGYYDIDAQTFADWGVDLLKFDGCYCDSLENLADGYKHMSLALNRTGRSIVYSCEWPLYMWPFQKPNYTEIRQYCNHWRNFADIDDSWKSIKSILDWTSFNQERIVDVAGPGGWNDPDMLVIGNFGLSWNQQVTQMALWAIMAAPLFMSNDLRHISPQAKALLQDKDVIAINQDPLGKQGYQLRQGDNFEVWERPLSGLAWAVAMINRQEIGGPRSYTIAVASLGKGVACNPACFITQLLPVKRKLGFYEWTSRLRSHINPTGTVLLQLENTMQMSLKDLL";
var posizione_amminoacidica = (parseInt(parseInt(document.form_1.posizione_nucleotidica.value-1)/3)+1)+"";
var amminoacido_originale = sequenza.slice(parseInt(posizione_amminoacidica-1),parseInt(posizione_amminoacidica));

var nucleotide_originale = sequenza_nucleotidica[parseInt(document.form_1.posizione_nucleotidica.value-1)];
var nucleotide_mutato = document.form_1.nucleotide_mutato.value;

var richiesta_sostituzione_iniziale = 'c.'+document.form_1.posizione_nucleotidica.value +nucleotide_originale+'>' + nucleotide_mutato ;
//non muovere la variabile da qui che dopo modifico la  lista sequenza_nucleotidica

sequenza_nucleotidica[parseInt(document.form_1.posizione_nucleotidica.value)-1]=document.form_1.nucleotide_mutato.value;
sequenza_nucleotidica = sequenza_nucleotidica.join('');

var codone_mutato = sequenza_nucleotidica.slice((posizione_amminoacidica*3)-3,(posizione_amminoacidica*3))

var amminoacido_mutato;
if (codone_mutato == 'GCT'){amminoacido_mutato ='A';}
else if (codone_mutato == 'GCC'){amminoacido_mutato ='A';}
else if (codone_mutato == 'GCA'){amminoacido_mutato ='A';}
else if (codone_mutato == 'GCG'){amminoacido_mutato ='A';}

else if (codone_mutato == 'CGT'){amminoacido_mutato ='R';}
else if (codone_mutato == 'CGC'){amminoacido_mutato ='R';}
else if (codone_mutato == 'CGA'){amminoacido_mutato ='R';}
else if (codone_mutato == 'CGG'){amminoacido_mutato ='R';}
else if (codone_mutato == 'AGA'){amminoacido_mutato ='R';}
else if (codone_mutato == 'AGG'){amminoacido_mutato ='R';}


else if (codone_mutato == 'AAT'){amminoacido_mutato ='N';}
else if (codone_mutato == 'AAC'){amminoacido_mutato ='N';}


else if (codone_mutato == 'GAT'){amminoacido_mutato ='D';}
else if (codone_mutato == 'GAC'){amminoacido_mutato ='D';}


else if (codone_mutato == 'TGT'){amminoacido_mutato ='C';}
else if (codone_mutato == 'TGC'){amminoacido_mutato ='C';}


else if (codone_mutato == 'CAA'){amminoacido_mutato ='Q';}
else if (codone_mutato == 'CAG'){amminoacido_mutato ='Q';}


else if (codone_mutato == 'GAA'){amminoacido_mutato ='E';}
else if (codone_mutato == 'GAG'){amminoacido_mutato ='E';}


else if (codone_mutato == 'GGT'){amminoacido_mutato ='G';}
else if (codone_mutato == 'GGC'){amminoacido_mutato ='G';}
else if (codone_mutato == 'GGA'){amminoacido_mutato ='G';}
else if (codone_mutato == 'GGG'){amminoacido_mutato ='G';}


else if (codone_mutato == 'CAT'){amminoacido_mutato ='H';}
else if (codone_mutato == 'CAC'){amminoacido_mutato ='H';}


else if (codone_mutato == 'ATT'){amminoacido_mutato ='I';}
else if (codone_mutato == 'ATC'){amminoacido_mutato ='I';}
else if (codone_mutato == 'ATA'){amminoacido_mutato ='I';}

else if (codone_mutato == 'TTA'){amminoacido_mutato ='L';}
else if (codone_mutato == 'TTG'){amminoacido_mutato ='L';}
else if (codone_mutato == 'CTT'){amminoacido_mutato ='L';}
else if (codone_mutato == 'CTC'){amminoacido_mutato ='L';}
else if (codone_mutato == 'CTA'){amminoacido_mutato ='L';}
else if (codone_mutato == 'CTG'){amminoacido_mutato ='L';}


else if (codone_mutato == 'AAA'){amminoacido_mutato ='K';}
else if (codone_mutato == 'AAG'){amminoacido_mutato ='K';}


else if (codone_mutato == 'ATG'){amminoacido_mutato ='M';}


else if (codone_mutato == 'TTT'){amminoacido_mutato ='F';}
else if (codone_mutato == 'TTC'){amminoacido_mutato ='F';}


else if (codone_mutato == 'CCT'){amminoacido_mutato ='P';}
else if (codone_mutato == 'CCC'){amminoacido_mutato ='P';}
else if (codone_mutato == 'CCA'){amminoacido_mutato ='P';}
else if (codone_mutato == 'CCG'){amminoacido_mutato ='P';}


else if (codone_mutato == 'TCT'){amminoacido_mutato ='S';}
else if (codone_mutato == 'TCC'){amminoacido_mutato ='S';}
else if (codone_mutato == 'TCA'){amminoacido_mutato ='S';}
else if (codone_mutato == 'TCG'){amminoacido_mutato ='S';}
else if (codone_mutato == 'AGT'){amminoacido_mutato ='S';}
else if (codone_mutato == 'AGC'){amminoacido_mutato ='S';}


else if (codone_mutato == 'ACT'){amminoacido_mutato ='T';}
else if (codone_mutato == 'ACC'){amminoacido_mutato ='T';}
else if (codone_mutato == 'ACA'){amminoacido_mutato ='T';}
else if (codone_mutato == 'ACG'){amminoacido_mutato ='T';}


else if (codone_mutato == 'TGG'){amminoacido_mutato ='W';}


else if (codone_mutato == 'TAT'){amminoacido_mutato ='Y';}
else if (codone_mutato == 'TAC'){amminoacido_mutato ='Y';}

else if (codone_mutato == 'GTT'){amminoacido_mutato ='V';}
else if (codone_mutato == 'GTC'){amminoacido_mutato ='V';}
else if (codone_mutato == 'GTA'){amminoacido_mutato ='V';}
else if (codone_mutato == 'GTG'){amminoacido_mutato ='V';}

else if (codone_mutato == 'TAA'){amminoacido_mutato ='*';}
else if (codone_mutato == 'TGA'){amminoacido_mutato ='*';}
else if (codone_mutato == 'TAG'){amminoacido_mutato ='*';}


var sostituzione = amminoacido_originale+posizione_amminoacidica+amminoacido_mutato;	
	
richiesta_sostituzione_iniziale = richiesta_sostituzione_iniziale+' (p.'+sostituzione+')';


if (nucleotide_originale == nucleotide_mutato) {
    document.getElementById("sostituzione").innerHTML= richiesta_sostituzione_iniziale;
	 document.getElementById("presence_in_dataset").innerHTML= '/';
	 document.getElementById("ponti_disolfuro").innerHTML= '/';
	 document.getElementById("sito_attivo").innerHTML= '/';
	 document.getElementById("valore_pssm").innerHTML= '/';
    document.getElementById("finale").innerHTML= "Identical to wild type";
    document.getElementById("jmol").setAttribute("href", "javascript:document.jmol.script('select all; color white; wireframe off;ribbon;spacefill off; select 47,92,93,134,142,168,170,203,206,207,227,231,267,230 ; color blue; select 52,94,56,63,142,172,202,223,378,382; color yellow;')");
	 document.getElementById("jmol").click();
	 
	 ;}
	 
	 
else if (amminoacido_originale == amminoacido_mutato) {
     document.getElementById("sostituzione").innerHTML= richiesta_sostituzione_iniziale;
	 document.getElementById("presence_in_dataset").innerHTML= '/';
	 document.getElementById("ponti_disolfuro").innerHTML= '/';
	 document.getElementById("sito_attivo").innerHTML= '/';
	 document.getElementById("valore_pssm").innerHTML= '/';
     document.getElementById("finale").innerHTML= "Silent mutation, identical to wild type"
     document.getElementById("jmol").setAttribute("href", "javascript:document.jmol.script('select all; color white; wireframe off;ribbon;spacefill off; select 47,92,93,134,142,168,170,203,206,207,227,231,267,230 ; color blue; select 52,94,56,63,142,172,202,223,378,382; color yellow;')");
	 document.getElementById("jmol").click();	
	 
	 ;}
	 
else if (amminoacido_mutato == '*') {
	
     document.getElementById("sostituzione").innerHTML= richiesta_sostituzione_iniziale;
	 document.getElementById("presence_in_dataset").innerHTML= '/';
	 document.getElementById("ponti_disolfuro").innerHTML= '/';
	 document.getElementById("sito_attivo").innerHTML= '/';
	 document.getElementById("valore_pssm").innerHTML= '/';
     document.getElementById("finale").innerHTML= "Nonsense mutation is Non Responsive, truncated protein "
     document.getElementById("jmol").setAttribute("href", "javascript:document.jmol.script('select all; color white; wireframe off;ribbon;spacefill off; select 47,92,93,134,142,168,170,203,206,207,227,231,267,230 ; color blue; select 52,94,56,63,142,172,202,223,378,382; color yellow;')");
	 document.getElementById("jmol").click();		
	
	;}
	
	
else{vai(richiesta_sostituzione_iniziale,posizione_amminoacidica,sostituzione)}
}


function controllo2()
{

var sequenza = "MQLRNPELHLGCALALRFLALVSWDIPGARALDNGLARTPTMGWLHWERFMCNLDCQEEPDSCISEKLFMEMAELMVSEGWKDAGYEYLCIDDCWMAPQRDSEGRLQADPQRFPHGIRQLANYVHSKGLKLGIYADVGNKTCAGFPGSFGYYDIDAQTFADWGVDLLKFDGCYCDSLENLADGYKHMSLALNRTGRSIVYSCEWPLYMWPFQKPNYTEIRQYCNHWRNFADIDDSWKSIKSILDWTSFNQERIVDVAGPGGWNDPDMLVIGNFGLSWNQQVTQMALWAIMAAPLFMSNDLRHISPQAKALLQDKDVIAINQDPLGKQGYQLRQGDNFEVWERPLSGLAWAVAMINRQEIGGPRSYTIAVASLGKGVACNPACFITQLLPVKRKLGFYEWTSRLRSHINPTGTVLLQLENTMQMSLKDLL";
var posizione_amminoacidica = document.form_1.posizione_amminoacidica.value;
var amminoacido_originale = sequenza.slice(parseInt(posizione_amminoacidica-1),parseInt(posizione_amminoacidica));
var amminoacido_mutato = document.form_1.amminoacido_mutato.value;
var sostituzione = amminoacido_originale+posizione_amminoacidica+amminoacido_mutato;

var richiesta_sostituzione_iniziale ='p.'+sostituzione;

if (amminoacido_originale == amminoacido_mutato) {

    document.getElementById("sostituzione").innerHTML= richiesta_sostituzione_iniziale;
	document.getElementById("presence_in_dataset").innerHTML= '/';
	document.getElementById("ponti_disolfuro").innerHTML= '/';
	document.getElementById("sito_attivo").innerHTML= '/';
	document.getElementById("valore_pssm").innerHTML= '/';
    document.getElementById("finale").innerHTML= "Identical to wild type "
    document.getElementById("jmol").setAttribute("href", "javascript:document.jmol.script('select all; color white; wireframe off;ribbon;spacefill off; select 47,92,93,134,142,168,170,203,206,207,227,231,267,230 ; color blue; select 52,94,56,63,142,172,202,223,378,382; color yellow;')");
	document.getElementById("jmol").click();
	
	
	
	;}

else{vai(richiesta_sostituzione_iniziale,posizione_amminoacidica,sostituzione)}
}


function vai(richiesta_sostituzione_iniziale,posizione_amminoacidica,sostituzione)
{

	
var presence_in_signal_peptide;


if (posizione_amminoacidica == "1") {presence_in_signal_peptide = 'Yes';}
else if (posizione_amminoacidica == "2") {presence_in_signal_peptide = 'Yes';}
else if (posizione_amminoacidica == "3") {presence_in_signal_peptide = 'Yes';}
else if (posizione_amminoacidica == "4") {presence_in_signal_peptide = 'Yes';}
else if (posizione_amminoacidica == "5") {presence_in_signal_peptide = 'Yes';}
else if (posizione_amminoacidica == "6") {presence_in_signal_peptide = 'Yes';}
else if (posizione_amminoacidica == "7") {presence_in_signal_peptide = 'Yes';}
else if (posizione_amminoacidica == "8") {presence_in_signal_peptide = 'Yes';}
else if (posizione_amminoacidica == "9") {presence_in_signal_peptide = 'Yes';}
else if (posizione_amminoacidica == "10") {presence_in_signal_peptide = 'Yes';}
else if (posizione_amminoacidica == "11") {presence_in_signal_peptide = 'Yes';}
else if (posizione_amminoacidica == "12") {presence_in_signal_peptide = 'Yes';}
else if (posizione_amminoacidica == "13") {presence_in_signal_peptide = 'Yes';}
else if (posizione_amminoacidica == "14") {presence_in_signal_peptide = 'Yes';}
else if (posizione_amminoacidica == "15") {presence_in_signal_peptide = 'Yes';}
else if (posizione_amminoacidica == "16") {presence_in_signal_peptide = 'Yes';}
else if (posizione_amminoacidica == "17") {presence_in_signal_peptide = 'Yes';}
else if (posizione_amminoacidica == "18") {presence_in_signal_peptide = 'Yes';}
else if (posizione_amminoacidica == "19") {presence_in_signal_peptide = 'Yes';}
else if (posizione_amminoacidica == "20") {presence_in_signal_peptide = 'Yes';}
else if (posizione_amminoacidica == "21") {presence_in_signal_peptide = 'Yes';}
else if (posizione_amminoacidica == "22") {presence_in_signal_peptide = 'Yes';}
else if (posizione_amminoacidica == "23") {presence_in_signal_peptide = 'Yes';}
else if (posizione_amminoacidica == "24") {presence_in_signal_peptide = 'Yes';}
else if (posizione_amminoacidica == "25") {presence_in_signal_peptide = 'Yes';}
else if (posizione_amminoacidica == "26") {presence_in_signal_peptide = 'Yes';}
else if (posizione_amminoacidica == "27") {presence_in_signal_peptide = 'Yes';}
else if (posizione_amminoacidica == "28") {presence_in_signal_peptide = 'Yes';}
else if (posizione_amminoacidica == "29") {presence_in_signal_peptide = 'Yes';}
else if (posizione_amminoacidica == "30") {presence_in_signal_peptide = 'Yes';}
else if (posizione_amminoacidica == "31") {presence_in_signal_peptide = 'Yes';}

else {presence_in_signal_peptide = 'No';}

var presence_in_dataset;

if (sostituzione == "A108T") {presence_in_dataset = "    Author  Year  Type  -DGJ  +DGJ  Link    Benjamin et al.  2016  HEK-293  57.1 ± 2.2  80.8 ± 2.9   go    ";}
else if (sostituzione == "A121T") {presence_in_dataset = "    Author  Year  Type  -DGJ  +DGJ  Link    Benjamin et al.  2016  HEK-293  18.9 ± 0.5  67.9 ± 3.4   go     Lukas et al.  2016  HEK293H  50 ± 8.4  55.5 ± 6.2   go    ";}
else if (sostituzione == "A135V") {presence_in_dataset = "    Author  Year  Type  -DGJ  +DGJ  Link    Benjamin et al.  2016  HEK-293  NR  3.7 ± 0.1   go     Lukas et al.  2013  HEK293H  0  6.9 ± 2.4   go    ";}
else if (sostituzione == "A13P") {presence_in_dataset = "    Author  Year  Type  -DGJ  +DGJ  Link    Benjamin et al.  2016  HEK-293  5.6 ± 0.4  14.9 ± 0.6   go    ";}
else if (sostituzione == "A13T") {presence_in_dataset = "    Author  Year  Type  -DGJ  +DGJ  Link    Benjamin et al.  2016  HEK-293  51.7 ± 2.5  62.2 ± 2.6   go    ";}
else if (sostituzione == "A143P") {presence_in_dataset = "    Author  Year  Type  -DGJ  +DGJ  Link    Lukas et al.  2013  HEK293H  0  0   go     Wu et al.  2011  HEK293-Sample-1  0  0   go     Benjamin et al.  2009  Lymphoblasts  0  0   go     Shin et al.  2008  T-Cell-Sample-1  2 ± 1  1 ± 1   go     Shin et al.  2008  T-Cell-Sample-2  1 ± 1  1 ± 0   go     Shin et al.  2007  T-Cell-Sample-1  1.6 ± 1.3  1 ± 1.1   go     Shin et al.  2007  T-Cell-Sample-2  0.6 ± 0.8  1 ± 0.1   go     Shin et al.  2007  Fibroblasts-Sample-1  1.8 ± 2.1  0 ± 0.11   go    ";}
else if (sostituzione == "A143T") {presence_in_dataset = "    Author  Year  Type  -DGJ  +DGJ  Link    Benjamin et al.  2016  HEK-293  21.4 ± 0.8  43.8 ± 1.4   go     Lukas et al.  2013  HEK293H  31.3 ± 5.6  49.4 ± 4.8   go     Wu et al.  2011  HEK293-Sample-1  51.9 ± 5.2  83.8 ± 16   go     Wu et al.  2011  HEK293-Sample-2  52  69.4   go     Benjamin et al.  2009  Lymphoblasts  36 ± 9  91   go     Shin et al.  2008  T-Cell-Sample-1  31 ± 5  69 ± 11   go     Shin et al.  2008  T-Cell-Sample-2  46 ± 13  62 ± 20   go     Shin et al.  2007  T-Cell-Sample-1  30.6 ± 4.6  68.8 ± 11.4   go     Spada et al.  2006  COS-7  36  57.96   go    ";}
else if (sostituzione == "A156D") {presence_in_dataset = "    Author  Year  Type  -DGJ  +DGJ  Link    Lukas et al.  2013  HEK293H  0  0   go    ";}
else if (sostituzione == "A156T") {presence_in_dataset = "    Author  Year  Type  -DGJ  +DGJ  Link    Benjamin et al.  2016  HEK-293  2.8 ± 0.1  21.9 ± 0.9   go    ";}
else if (sostituzione == "A156V") {presence_in_dataset = "    Author  Year  Type  -DGJ  +DGJ  Link    Benjamin et al.  2016  HEK-293  1.2 ± 0.1  12.8 ± 0.7   go     Lukas et al.  2013  HEK293H  4.3 ± 1  16.8 ± 2.3   go    ";}
else if (sostituzione == "A15E") {presence_in_dataset = "    Author  Year  Type  -DGJ  +DGJ  Link    Lukas et al.  2013  HEK293H  0  0   go    ";}
else if (sostituzione == "A15G") {presence_in_dataset = "    Author  Year  Type  -DGJ  +DGJ  Link    Benjamin et al.  2016  HEK-293  19 ± 0.7  28 ± 1.2   go    ";}
else if (sostituzione == "A15P") {presence_in_dataset = "    Author  Year  Type  -DGJ  +DGJ  Link    Benjamin et al.  2016  HEK-293  0.9 ± 0  1.2 ± 0.1   go    ";}
else if (sostituzione == "A15T") {presence_in_dataset = "    Author  Year  Type  -DGJ  +DGJ  Link    Benjamin et al.  2016  HEK-293  39.1 ± 1.3  57.5 ± 1.4   go    ";}
else if (sostituzione == "A20D") {presence_in_dataset = "    Author  Year  Type  -DGJ  +DGJ  Link    Benjamin et al.  2016  HEK-293  4.3 ± 0.2  10 ± 0.8   go     Lukas et al.  2016  HEK293H  2.8 ± 0.1  4.5 ± 0.2   go    ";}
else if (sostituzione == "A20P") {presence_in_dataset = "    Author  Year  Type  -DGJ  +DGJ  Link    Benjamin et al.  2016  HEK-293  11.5 ± 0.8  15.9 ± 1.1   go     Lukas et al.  2016  HEK293H  2.5 ± 0.4  4.9 ± 0.8   go    ";}
else if (sostituzione == "A20V") {presence_in_dataset = "    Author  Year  Type  -DGJ  +DGJ  Link    Benjamin et al.  2016  HEK-293  81.7 ± 3.7  86.1 ± 3.6   go    ";}
else if (sostituzione == "A230T") {presence_in_dataset = "    Author  Year  Type  -DGJ  +DGJ  Link    Andreotti et al.  2011  COS-7  3.6 ± 0.2  7.7 ± 2   go    ";}
else if (sostituzione == "A257D") {presence_in_dataset = "    Author  Year  Type  -DGJ  +DGJ  Link    Benjamin et al.  2016  HEK-293  NR  1.0 ± 0.1   go    ";}
else if (sostituzione == "A257G") {presence_in_dataset = "    Author  Year  Type  -DGJ  +DGJ  Link    Benjamin et al.  2016  HEK-293  59.0 ± 2.8  101.7 ± 2.8   go    ";}
else if (sostituzione == "A257P") {presence_in_dataset = "    Author  Year  Type  -DGJ  +DGJ  Link    Benjamin et al.  2016  HEK-293  16.6 ± 1.2  52.3 ± 1.6   go    ";}
else if (sostituzione == "A285D") {presence_in_dataset = "    Author  Year  Type  -DGJ  +DGJ  Link    Lukas et al.  2013  HEK293H  0  0   go    ";}
else if (sostituzione == "A288D") {presence_in_dataset = "    Author  Year  Type  -DGJ  +DGJ  Link    Benjamin et al.  2016  HEK-293  NR  2.1 ± 0.1   go    ";}
else if (sostituzione == "A288P") {presence_in_dataset = "    Author  Year  Type  -DGJ  +DGJ  Link    Benjamin et al.  2016  HEK-293  1.1 ± 0.1  10.4 ± 0.7   go     Wu et al.  2011  HEK293-Sample-1  0  13.6 ± 2.3   go     Benjamin et al.  2009  Lymphoblasts  0.3 ± 0.2  11   go    ";}
else if (sostituzione == "A291T") {presence_in_dataset = "    Author  Year  Type  -DGJ  +DGJ  Link    Benjamin et al.  2016  HEK-293  16.5 ± 1.5  40.5 ± 3.8   go     Lukas et al.  2013  HEK293H  13.2 ± 3.4  55.7 ± 0.7   go    ";}
else if (sostituzione == "A292T") {presence_in_dataset = "    Author  Year  Type  -DGJ  +DGJ  Link    Benjamin et al.  2016  HEK-293  NR  2.3 ± 0.1   go    ";}
else if (sostituzione == "A307T") {presence_in_dataset = "    Author  Year  Type  -DGJ  +DGJ  Link    Benjamin et al.  2016  HEK-293  36.2 ± 1.2  87.7 ± 1.5   go    ";}
else if (sostituzione == "A309P") {presence_in_dataset = "    Author  Year  Type  -DGJ  +DGJ  Link    Benjamin et al.  2016  HEK-293  0.9 ± 0.1  10.0 ± 0.6   go    ";}
else if (sostituzione == "A309V") {presence_in_dataset = "    Author  Year  Type  -DGJ  +DGJ  Link    Lukas et al.  2016  HEK293H  48 ± 0.5  46.6 ± 1.6   go    ";}
else if (sostituzione == "A31V") {presence_in_dataset = "    Author  Year  Type  -DGJ  +DGJ  Link    Benjamin et al.  2016  HEK-293  1.7 ± 0.1  3.0 ± 0.1   go    ";}
else if (sostituzione == "A348P") {presence_in_dataset = "    Author  Year  Type  -DGJ  +DGJ  Link    Benjamin et al.  2016  HEK-293  0.5 ± 0.1  2.4 ± 0.2   go    ";}
else if (sostituzione == "A352D") {presence_in_dataset = "    Author  Year  Type  -DGJ  +DGJ  Link    Benjamin et al.  2016  HEK-293  NR  1.6 ± 0.1   go    ";}
else if (sostituzione == "A352G") {presence_in_dataset = "    Author  Year  Type  -DGJ  +DGJ  Link    Lukas et al.  2016  HEK293H  53.7 ± 4.2  56.4 ± 2.9   go    ";}
else if (sostituzione == "A352V") {presence_in_dataset = "    Author  Year  Type  -DGJ  +DGJ  Link    Benjamin et al.  2016  HEK-293  44.5 ± 1.8  70.2 ± 3.2   go    ";}
else if (sostituzione == "A368T") {presence_in_dataset = "    Author  Year  Type  -DGJ  +DGJ  Link    Benjamin et al.  2016  HEK-293  54.6 ± 1.7  72.6 ± 1.8   go     Lukas et al.  2013  HEK293H  103.7 ± 33.6  93.3 ± 5   go    ";}
else if (sostituzione == "A377D") {presence_in_dataset = "    Author  Year  Type  -DGJ  +DGJ  Link    Benjamin et al.  2016  HEK-293  NR  0.7 ± 0.1   go    ";}
else if (sostituzione == "A37T") {presence_in_dataset = "    Author  Year  Type  -DGJ  +DGJ  Link    Benjamin et al.  2016  HEK-293  48.9 ± 1.8  96.4 ± 3.9   go     Lukas et al.  2013  HEK293H  69.6 ± 13.6  132.9 ± 27.8   go    ";}
else if (sostituzione == "A37V") {presence_in_dataset = "    Author  Year  Type  -DGJ  +DGJ  Link    Benjamin et al.  2016  HEK-293  46.7 ± 2.0  75.1 ± 3.9   go    ";}
else if (sostituzione == "A73E") {presence_in_dataset = "    Author  Year  Type  -DGJ  +DGJ  Link    Benjamin et al.  2016  HEK-293  0.5 ± 0  1.4 ± 0.1   go    ";}
else if (sostituzione == "A73V") {presence_in_dataset = "    Author  Year  Type  -DGJ  +DGJ  Link    Benjamin et al.  2016  HEK-293  53.6 ± 1.6  86.9 ± 2.1   go     Lukas et al.  2013  HEK293H  44 ± 8  64.7 ± 8.3   go     Spada et al.  2006  COS-7  44  77.88   go    ";}
else if (sostituzione == "A97P") {presence_in_dataset = "    Author  Year  Type  -DGJ  +DGJ  Link    Benjamin et al.  2016  HEK-293  0.7 ± 0.0  4.0 ± 0.1   go    ";}
else if (sostituzione == "A97V") {presence_in_dataset = "    Author  Year  Type  -DGJ  +DGJ  Link    Benjamin et al.  2016  HEK-293  12.2 ± 0.8  39.6 ± 2.3   go     Wu et al.  2011  HEK293-Sample-1  5.7 ± 1.3  48 ± 12.5   go     Wu et al.  2011  HEK293-Sample-2  5.7  31.6   go     Benjamin et al.  2009  Lymphoblasts  28 ± 7  111   go     Benjamin et al.  2009  Fibroblasts  32 ± 13  91   go     Shin et al.  2008  T-Cell-Sample-1  14 ± 4  75 ± 8   go     Shin et al.  2007  T-Cell-Sample-1  13.7 ± 4.2  74.9 ± 8.4   go     Shin et al.  2007  Fibroblasts-Sample-1  15.4  43.8   go     Shin et al.  2007  Fibroblasts-Sample-2  15.4  30.6   go     Ishii et al.  2007  Fibroblasts  11.1  32.2   go     Ishii et al.  2007  Lymphoblasts  7.5  61.5   go    ";}
else if (sostituzione == "C142R") {presence_in_dataset = "    Author  Year  Type  -DGJ  +DGJ  Link    Benjamin et al.  2016  HEK-293  0.4 ± 0.0  NR   go     Lukas et al.  2013  HEK293H  0  0   go     Wu et al.  2011  HEK293-Sample-1  0  0   go     Benjamin et al.  2009  Lymphoblasts  0  0   go    ";}
else if (sostituzione == "C142W") {presence_in_dataset = "    Author  Year  Type  -DGJ  +DGJ  Link    Park et al.  2009  COS-7  5.2 ± 0.39  7 ± 0.59   go    ";}
else if (sostituzione == "C172G") {presence_in_dataset = "    Author  Year  Type  -DGJ  +DGJ  Link    Benjamin et al.  2016  HEK-293  1.1 ± 0.1  2.7 ± 0.2   go     Lukas et al.  2013  HEK293H  0  4.4   go     Wu et al.  2011  HEK293-Sample-1  0  0   go     Benjamin et al.  2009  Lymphoblasts  0  0   go    ";}
else if (sostituzione == "C172Y") {presence_in_dataset = "    Author  Year  Type  -DGJ  +DGJ  Link    Lukas et al.  2013  HEK293H  0  0   go     Wu et al.  2011  HEK293-Sample-1  0  0   go     Benjamin et al.  2009  Lymphoblasts  2 ± 1.2  3   go    ";}
else if (sostituzione == "C174G") {presence_in_dataset = "    Author  Year  Type  -DGJ  +DGJ  Link    Benjamin et al.  2016  HEK-293  29.6 ± 1.5  91.2 ± 5.2   go    ";}
else if (sostituzione == "C174R") {presence_in_dataset = "    Author  Year  Type  -DGJ  +DGJ  Link    Benjamin et al.  2016  HEK-293  14.3 ± 0.6  51.6 ± 2.0   go    ";}
else if (sostituzione == "C202Y") {presence_in_dataset = "    Author  Year  Type  -DGJ  +DGJ  Link    Lukas et al.  2016  HEK293H  0  1.4 ± 0.5   go     Wu et al.  2011  HEK293-Sample-1  0  0   go     Benjamin et al.  2009  Lymphoblasts  0  0   go    ";}
else if (sostituzione == "C378R") {presence_in_dataset = "    Author  Year  Type  -DGJ  +DGJ  Link    Lukas et al.  2013  HEK293H  0  0   go    ";}
else if (sostituzione == "C52G") {presence_in_dataset = "    Author  Year  Type  -DGJ  +DGJ  Link    Giugliani et al.  2013  HEK-293  0  0   go    ";}
else if (sostituzione == "C52W") {presence_in_dataset = "    Author  Year  Type  -DGJ  +DGJ  Link    Lukas et al.  2013  HEK293H  0  0   go    ";}
else if (sostituzione == "C52Y") {presence_in_dataset = "    Author  Year  Type  -DGJ  +DGJ  Link    Filoni et al.  2010  COS-1  2.5  5   go     Filoni et al.  2010  Lymphocytes  5  3.5   go    ";}
else if (sostituzione == "C56F") {presence_in_dataset = "    Author  Year  Type  -DGJ  +DGJ  Link    Benjamin et al.  2016  HEK-293  NR  6.4 ± 0.7   go    ";}
else if (sostituzione == "C56Y") {presence_in_dataset = "    Author  Year  Type  -DGJ  +DGJ  Link    Benjamin et al.  2016  HEK-293  NR  7.3 ± 0.7   go     Lukas et al.  2016  HEK293H  0  3.3 ± 1.1   go    ";}
else if (sostituzione == "C63Y") {presence_in_dataset = "    Author  Year  Type  -DGJ  +DGJ  Link    Lukas et al.  2013  HEK293H  0  0   go    ";}
else if (sostituzione == "C94S") {presence_in_dataset = "    Author  Year  Type  -DGJ  +DGJ  Link    Lukas et al.  2016  HEK293H  0  0   go     Wu et al.  2011  HEK293-Sample-1  0  0   go     Wu et al.  2011  HEK293-Sample-2  0  0   go     Shin et al.  2008  T-Cell-Sample-1  2 ± 1  2 ± 0   go    ";}
else if (sostituzione == "C94Y") {presence_in_dataset = "    Author  Year  Type  -DGJ  +DGJ  Link    Lukas et al.  2016  HEK293H  0  0   go    ";}
else if (sostituzione == "D109G") {presence_in_dataset = "    Author  Year  Type  -DGJ  +DGJ  Link    Benjamin et al.  2016  HEK-293  2.6 ± 0.1  10.3 ± 0.5   go    ";}
else if (sostituzione == "D136E") {presence_in_dataset = "    Author  Year  Type  -DGJ  +DGJ  Link    Benjamin et al.  2016  HEK-293  1.4 ± 0.1  12.9 ± 0.8   go     Lukas et al.  2013  HEK293H  0  31.3 ± 3.2   go    ";}
else if (sostituzione == "D136H") {presence_in_dataset = "    Author  Year  Type  -DGJ  +DGJ  Link    Benjamin et al.  2016  HEK-293  1.6 ± 0.1  4.0 ± 0.2   go    ";}
else if (sostituzione == "D155H") {presence_in_dataset = "    Author  Year  Type  -DGJ  +DGJ  Link    Benjamin et al.  2016  HEK-293  NR  1.4 ± 0.1   go    ";}
else if (sostituzione == "D165G") {presence_in_dataset = "    Author  Year  Type  -DGJ  +DGJ  Link    Benjamin et al.  2016  HEK-293  2.5 ± 0.1  13 ± 0.4   go    ";}
else if (sostituzione == "D165H") {presence_in_dataset = "    Author  Year  Type  -DGJ  +DGJ  Link    Benjamin et al.  2016  HEK-293  1.3 ± 0.1  8.3 ± 0.9   go     Lukas et al.  2013  HEK293H  3.4 ± 1.1  11.9 ± 1.1   go    ";}
else if (sostituzione == "D165V") {presence_in_dataset = "    Author  Year  Type  -DGJ  +DGJ  Link    Lukas et al.  2013  HEK293H  0  0   go    ";}
else if (sostituzione == "D165Y") {presence_in_dataset = "    Author  Year  Type  -DGJ  +DGJ  Link    Benjamin et al.  2016  HEK-293  NR  1.3 ± 0.1   go     Lukas et al.  2013  HEK293H  0  0   go    ";}
else if (sostituzione == "D170N") {presence_in_dataset = "    Author  Year  Type  -DGJ  +DGJ  Link    Lukas et al.  2013  HEK293H  0  0   go    ";}
else if (sostituzione == "D170V") {presence_in_dataset = "    Author  Year  Type  -DGJ  +DGJ  Link    Wu et al.  2011  HEK293-Sample-1  0  0   go     Benjamin et al.  2009  Lymphoblasts  1.2 ± 0.4  2   go    ";}
else if (sostituzione == "D175E") {presence_in_dataset = "    Author  Year  Type  -DGJ  +DGJ  Link    Benjamin et al.  2016  HEK-293  44.3 ± 2.9  53.4 ± 2   go     Lukas et al.  2013  HEK293H  89.8 ± 2.2  89 ± 7.3   go    ";}
else if (sostituzione == "D175N") {presence_in_dataset = "    Author  Year  Type  -DGJ  +DGJ  Link    Lukas et al.  2013  HEK293H  70 ± 2.1  84.3 ± 8.5   go    ";}
else if (sostituzione == "D231G") {presence_in_dataset = "    Author  Year  Type  -DGJ  +DGJ  Link    Park et al.  2009  COS-7  4.4 ± 0.32  5.1 ± 0.2   go    ";}
else if (sostituzione == "D231N") {presence_in_dataset = "    Author  Year  Type  -DGJ  +DGJ  Link    Benjamin et al.  2016  HEK-293  0.5 ± 0.0  NR   go     Lukas et al.  2013  HEK293H  0  0   go    ";}
else if (sostituzione == "D231V") {presence_in_dataset = "    Author  Year  Type  -DGJ  +DGJ  Link    Shimotori et al.  2007  COS-7  3.4  4.2   go    ";}
else if (sostituzione == "D234E") {presence_in_dataset = "    Author  Year  Type  -DGJ  +DGJ  Link    Benjamin et al.  2016  HEK-293  0.4 ± 0.0  0.8 ± 0.1   go    ";}
else if (sostituzione == "D244H") {presence_in_dataset = "    Author  Year  Type  -DGJ  +DGJ  Link    Benjamin et al.  2016  HEK-293  18.1 ± 1.1  44.0 ± 1.3   go     Andreotti et al.  2011  COS-7  15.2 ± 8.1  50.2 ± 18   go    ";}
else if (sostituzione == "D244N") {presence_in_dataset = "    Author  Year  Type  -DGJ  +DGJ  Link    Benjamin et al.  2016  HEK-293  30.9 ± 1.6  48.7 ± 1.7   go     Wu et al.  2011  HEK293-Sample-1  43.2 ± 1.5  73.9 ± 2.2   go     Benjamin et al.  2009  Lymphoblasts  10 ± 2  114   go    ";}
else if (sostituzione == "D264A") {presence_in_dataset = "    Author  Year  Type  -DGJ  +DGJ  Link    Lukas et al.  2013  HEK293H  0  0   go    ";}
else if (sostituzione == "D264E") {presence_in_dataset = "    Author  Year  Type  -DGJ  +DGJ  Link    Lukas et al.  2013  HEK293H  0  0   go    ";}
else if (sostituzione == "D264G") {presence_in_dataset = "    Author  Year  Type  -DGJ  +DGJ  Link    Lukas et al.  2013  HEK293H  0  0   go    ";}
else if (sostituzione == "D264H") {presence_in_dataset = "    Author  Year  Type  -DGJ  +DGJ  Link    Lukas et al.  2013  HEK293H  1.7 ± 0.9  10.2 ± 2.9   go    ";}
else if (sostituzione == "D264N") {presence_in_dataset = "    Author  Year  Type  -DGJ  +DGJ  Link    Lukas et al.  2013  HEK293H  37.9 ± 3.7  84.5 ± 6.4   go    ";}
else if (sostituzione == "D264V") {presence_in_dataset = "    Author  Year  Type  -DGJ  +DGJ  Link    Lukas et al.  2013  HEK293H  0  0   go     Wu et al.  2011  HEK293-Sample-1  0  0   go     Benjamin et al.  2009  Lymphoblasts  0 ± 0  0   go    ";}
else if (sostituzione == "D264Y") {presence_in_dataset = "    Author  Year  Type  -DGJ  +DGJ  Link    Benjamin et al.  2016  HEK-293  0.5 ± 0.0  6.2 ± 0.3   go     Lukas et al.  2013  HEK293H  0  5.4 ± 2.9   go    ";}
else if (sostituzione == "D266N") {presence_in_dataset = "    Author  Year  Type  -DGJ  +DGJ  Link    Park et al.  2009  COS-7  5.2 ± 0.23  6.4 ± 0.67   go    ";}
else if (sostituzione == "D266V") {presence_in_dataset = "    Author  Year  Type  -DGJ  +DGJ  Link    Wu et al.  2011  HEK293-Sample-1  0  0   go     Benjamin et al.  2009  Lymphoblasts  0  0   go    ";}
else if (sostituzione == "D299E") {presence_in_dataset = "    Author  Year  Type  -DGJ  +DGJ  Link    Benjamin et al.  2016  HEK-293  34.2 ± 1.5  73 ± 2.8   go    ";}
else if (sostituzione == "D313G") {presence_in_dataset = "    Author  Year  Type  -DGJ  +DGJ  Link    Benjamin et al.  2016  HEK-293  25.5 ± 1.0  40.4 ± 1.4   go    ";}
else if (sostituzione == "D313N") {presence_in_dataset = "    Author  Year  Type  -DGJ  +DGJ  Link    Lukas et al.  2016  HEK293H  90.1 ± 23  95.2 ± 18.7   go    ";}
else if (sostituzione == "D313Y") {presence_in_dataset = "    Author  Year  Type  -DGJ  +DGJ  Link    Benjamin et al.  2016  HEK-293  59.0 ± 3.1  80.9 ± 2.4   go     Lukas et al.  2013  HEK293H  83.9 ± 21.1  100.3 ± 23.9   go    ";}
else if (sostituzione == "D315N") {presence_in_dataset = "    Author  Year  Type  -DGJ  +DGJ  Link    Lukas et al.  2016  HEK293H  65.3 ± 7.4  72.4 ± 12.4   go    ";}
else if (sostituzione == "D322E") {presence_in_dataset = "    Author  Year  Type  -DGJ  +DGJ  Link    Benjamin et al.  2016  HEK-293  6.7 ± 0.2  26.8 ± 1.3   go    ";}
else if (sostituzione == "D322N") {presence_in_dataset = "    Author  Year  Type  -DGJ  +DGJ  Link    Benjamin et al.  2016  HEK-293  36.1 ± 1.8  46.4 ± 1.6   go    ";}
else if (sostituzione == "D33G") {presence_in_dataset = "    Author  Year  Type  -DGJ  +DGJ  Link    Benjamin et al.  2016  HEK-293  29.3 ± 1.8  70.6 ± 2.4   go     Lukas et al.  2016  HEK293H  37.4 ± 5.1  62 ± 4.8   go    ";}
else if (sostituzione == "D33Y") {presence_in_dataset = "    Author  Year  Type  -DGJ  +DGJ  Link    Benjamin et al.  2016  HEK-293  18.0 ± 1.2  49.6 ± 1.7   go    ";}
else if (sostituzione == "D55V") {presence_in_dataset = "    Author  Year  Type  -DGJ  +DGJ  Link    Benjamin et al.  2016  HEK-293  0.5 ± 0.1  19.1 ± 1.0   go    ";}
else if (sostituzione == "D83N") {presence_in_dataset = "    Author  Year  Type  -DGJ  +DGJ  Link    Benjamin et al.  2016  HEK-293  69.2 ± 3.3  93.0 ± 3.7   go     Lukas et al.  2013  HEK293H  62.9 ± 12.8  71.6 ± 9.7   go    ";}
else if (sostituzione == "D92N") {presence_in_dataset = "    Author  Year  Type  -DGJ  +DGJ  Link    Wu et al.  2011  HEK293-Sample-1  0  0   go     Benjamin et al.  2009  Lymphoblasts  0  0   go    ";}
else if (sostituzione == "D92Y") {presence_in_dataset = "    Author  Year  Type  -DGJ  +DGJ  Link    Wu et al.  2011  HEK293-Sample-1  0  0   go     Benjamin et al.  2009  Lymphoblasts  0  0   go    ";}
else if (sostituzione == "D93E") {presence_in_dataset = "    Author  Year  Type  -DGJ  +DGJ  Link    Lukas et al.  2013  HEK293H  0  0   go    ";}
else if (sostituzione == "D93V") {presence_in_dataset = "    Author  Year  Type  -DGJ  +DGJ  Link    Shimotori et al.  2007  COS-7  1.2  1.5   go    ";}
else if (sostituzione == "D93Y") {presence_in_dataset = "    Author  Year  Type  -DGJ  +DGJ  Link    Lukas et al.  2013  HEK293H  0  0   go    ";}
else if (sostituzione == "E103Q") {presence_in_dataset = "    Author  Year  Type  -DGJ  +DGJ  Link    Benjamin et al.  2016  HEK-293  102.9 ± 3.7  114.6 ± 3.9   go    ";}
else if (sostituzione == "E203D") {presence_in_dataset = "    Author  Year  Type  -DGJ  +DGJ  Link    Benjamin et al.  2016  HEK-293  3.4 ± 0.1  9 ± 0.6   go    ";}
else if (sostituzione == "E203V") {presence_in_dataset = "    Author  Year  Type  -DGJ  +DGJ  Link    Benjamin et al.  2016  HEK-293  43.0 ± 2.4  64.5 ± 2.4   go    ";}
else if (sostituzione == "E338K") {presence_in_dataset = "    Author  Year  Type  -DGJ  +DGJ  Link    Benjamin et al.  2016  HEK-293  6.8 ± 0.4  18.6 ± 0.8   go    ";}
else if (sostituzione == "E341D") {presence_in_dataset = "    Author  Year  Type  -DGJ  +DGJ  Link    Benjamin et al.  2016  HEK-293  NR  1.6 ± 0.1   go     Lukas et al.  2013  HEK293H  0  0   go     Andreotti et al.  2011  COS-7  3.39 ± 0.46  7.5 ± 0.54   go    ";}
else if (sostituzione == "E341K") {presence_in_dataset = "    Author  Year  Type  -DGJ  +DGJ  Link    Lukas et al.  2013  HEK293H  0  0   go    ";}
else if (sostituzione == "E358A") {presence_in_dataset = "    Author  Year  Type  -DGJ  +DGJ  Link    Benjamin et al.  2016  HEK-293  1.9 ± 0.1  16.6 ± 0.8   go     Wu et al.  2011  HEK293-Sample-1  1.8 ± 0.4  26.9 ± 4.2   go     Benjamin et al.  2009  Lymphoblasts  6 ± 2  23   go    ";}
else if (sostituzione == "E358D") {presence_in_dataset = "    Author  Year  Type  -DGJ  +DGJ  Link    Benjamin et al.  2016  HEK-293  6.3 ± 0.2  31.0 ± 0.7   go    ";}
else if (sostituzione == "E358G") {presence_in_dataset = "    Author  Year  Type  -DGJ  +DGJ  Link    Benjamin et al.  2016  HEK-293  NR  5.5 ± 0.5   go    ";}
else if (sostituzione == "E358K") {presence_in_dataset = "    Author  Year  Type  -DGJ  +DGJ  Link    Lukas et al.  2016  HEK293H  0  0   go     Giugliani et al.  2013  HEK-293  0  0   go     Wu et al.  2011  HEK293-Sample-1  0  3.7 ± 0.5   go     Benjamin et al.  2009  Lymphoblasts  5 ± 1  17   go    ";}
else if (sostituzione == "E398A") {presence_in_dataset = "    Author  Year  Type  -DGJ  +DGJ  Link    Lukas et al.  2013  HEK293H  48.9 ± 4.7  62.5 ± 7.8   go    ";}
else if (sostituzione == "E398K") {presence_in_dataset = "    Author  Year  Type  -DGJ  +DGJ  Link    Benjamin et al.  2016  HEK-293  63.2 ± 3.2  113.6 ± 2.6   go    ";}
else if (sostituzione == "E418G") {presence_in_dataset = "    Author  Year  Type  -DGJ  +DGJ  Link    Benjamin et al.  2016  HEK-293  67.5 ± 1.7  89.4 ± 1.5   go     Lukas et al.  2013  HEK293H  74.6 ± 15.5  89.1 ± 4.9   go    ";}
else if (sostituzione == "E48D") {presence_in_dataset = "    Author  Year  Type  -DGJ  +DGJ  Link    Benjamin et al.  2016  HEK-293  0.5 ± 0.0  2.0 ± 0.1   go     Lukas et al.  2016  HEK293H  0  0   go    ";}
else if (sostituzione == "E48K") {presence_in_dataset = "    Author  Year  Type  -DGJ  +DGJ  Link    Benjamin et al.  2016  HEK-293  0.9 ± 0.1  1.3 ± 0.1   go    ";}
else if (sostituzione == "E48Q") {presence_in_dataset = "    Author  Year  Type  -DGJ  +DGJ  Link    Benjamin et al.  2016  HEK-293  2.3 ± 0.1  12.0 ± 0.4   go    ";}
else if (sostituzione == "E59K") {presence_in_dataset = "    Author  Year  Type  -DGJ  +DGJ  Link    Benjamin et al.  2016  HEK-293  8.6 ± 0.5  17.5 ± 0.8   go     Lukas et al.  2013  HEK293H  2.2 ± 0.5  18.5 ± 2.9   go     Wu et al.  2011  HEK293-Sample-1  3.6 ± 0.2  12.2 ± 0.7   go     Benjamin et al.  2009  Lymphoblasts  19 ± 3  54   go     Ishii et al.  2007  Fibroblasts  6  47.6   go    ";}
else if (sostituzione == "E66D") {presence_in_dataset = "    Author  Year  Type  -DGJ  +DGJ  Link    Ishii et al.  2000  COS-1  66.6  80   go    ";}
else if (sostituzione == "E66G") {presence_in_dataset = "    Author  Year  Type  -DGJ  +DGJ  Link    Benjamin et al.  2016  HEK-293  34.2 ± 1.0  46.2 ± 1.7   go     Spada et al.  2006  COS-7  39  76   go    ";}
else if (sostituzione == "E66K") {presence_in_dataset = "    Author  Year  Type  -DGJ  +DGJ  Link    Benjamin et al.  2016  HEK-293  4.8 ± 0.1  12.9 ± 0.8   go     Lukas et al.  2013  HEK293H  6.8 ± 0.3  18.3 ± 2.3   go    ";}
else if (sostituzione == "E66Q") {presence_in_dataset = "    Author  Year  Type  -DGJ  +DGJ  Link    Benjamin et al.  2016  HEK-293  52.0 ± 1.3  55.0 ± 2.1   go     Wu et al.  2011  HEK293-Sample-1  49.1 ± 3.8  61.2 ± 3.8   go     Benjamin et al.  2009  Lymphoblasts  32 ± 3  71   go     Park et al.  2009  COS-7  43.8 ± 3.03  39.3 ± 1.26   go     Shimotori et al.  2007  COS-7  49.8  60.5   go     Ishii et al.  2007  Lymphoblasts  29.9  43.2   go    ";}
else if (sostituzione == "E71G") {presence_in_dataset = "    Author  Year  Type  -DGJ  +DGJ  Link    Lukas et al.  2016  HEK293H  87 ± 4.8  104.6 ± 7.4   go    ";}
else if (sostituzione == "F113I") {presence_in_dataset = "    Author  Year  Type  -DGJ  +DGJ  Link    Lukas et al.  2016  HEK293H  15.6 ± 1.9  34 ± 5.5   go    ";}
else if (sostituzione == "F113L") {presence_in_dataset = "    Author  Year  Type  -DGJ  +DGJ  Link    Benjamin et al.  2016  HEK-293  18.3 ± 0.8  41.2 ± 1.5   go     Wu et al.  2011  HEK293-Sample-1  17.3 ± 3.6  67.6 ± 7.4   go     Benjamin et al.  2009  Lymphoblasts  4 ± 1  26   go     Park et al.  2009  COS-7  17.8 ± 0.88  47.1 ± 1.07   go     Ishii et al.  2007  Fibroblasts  1.8  35   go     Spada et al.  2006  COS-7  38  57   go    ";}
else if (sostituzione == "F113S") {presence_in_dataset = "    Author  Year  Type  -DGJ  +DGJ  Link    Wu et al.  2011  HEK293-Sample-1  0  0   go     Benjamin et al.  2009  Lymphoblasts  0  0   go    ";}
else if (sostituzione == "F169S") {presence_in_dataset = "    Author  Year  Type  -DGJ  +DGJ  Link    Benjamin et al.  2016  HEK-293  12.8 ± 1.4  78.7 ± 3.8   go    ";}
else if (sostituzione == "F18S") {presence_in_dataset = "    Author  Year  Type  -DGJ  +DGJ  Link    Benjamin et al.  2016  HEK-293  2.0 ± 0.1  2.5 ± 0.1   go    ";}
else if (sostituzione == "F229L") {presence_in_dataset = "    Author  Year  Type  -DGJ  +DGJ  Link    Benjamin et al.  2016  HEK-293  21.4 ± 1.1  37.6 ± 0.8   go    ";}
else if (sostituzione == "F273L") {presence_in_dataset = "    Author  Year  Type  -DGJ  +DGJ  Link    Benjamin et al.  2016  HEK-293  1.3 ± 0.1  1.8 ± 0.1   go     Lukas et al.  2013  HEK293H  0  0   go    ";}
else if (sostituzione == "F295C") {presence_in_dataset = "    Author  Year  Type  -DGJ  +DGJ  Link    Benjamin et al.  2016  HEK-293  3.4 ± 0.1  14.5 ± 0.6   go     Wu et al.  2011  HEK293-Sample-1  4.1 ± 0.3  44 ± 3.3   go     Wu et al.  2011  HEK293-Sample-2  4.1  12.3   go     Shin et al.  2008  T-Cell-Sample-1  1 ± 1  29 ± 5   go    ";}
else if (sostituzione == "F396Y") {presence_in_dataset = "    Author  Year  Type  -DGJ  +DGJ  Link    Benjamin et al.  2016  HEK-293  111.2 ± 4.0  116.4 ± 3.3   go     Lukas et al.  2013  HEK293H  87.6 ± 14.2  93.8 ± 15.7   go    ";}
else if (sostituzione == "F50C") {presence_in_dataset = "    Author  Year  Type  -DGJ  +DGJ  Link    Wu et al.  2011  HEK293-Sample-1  0  0   go     Benjamin et al.  2009  Lymphoblasts  0  0   go    ";}
else if (sostituzione == "G104V") {presence_in_dataset = "    Author  Year  Type  -DGJ  +DGJ  Link    Benjamin et al.  2016  HEK-293  20.9 ± 0.8  33.6 ± 1.1   go    ";}
else if (sostituzione == "G128E") {presence_in_dataset = "    Author  Year  Type  -DGJ  +DGJ  Link    Benjamin et al.  2016  HEK-293  45.2 ± 1.8  58.5 ± 1.7   go     Wu et al.  2011  HEK293-Sample-1  56.9 ± 11.2  85.8 ± 19.4   go     Benjamin et al.  2009  Lymphoblasts  0  0   go     Shin et al.  2008  T-Cell-Sample-1  3 ± 1  4 ± 0   go     Shin et al.  2008  T-Cell-Sample-2  2 ± 2  2 ± 1   go    ";}
else if (sostituzione == "G132A") {presence_in_dataset = "    Author  Year  Type  -DGJ  +DGJ  Link    Benjamin et al.  2016  HEK-293  NR  2.5 ± 0.1   go    ";}
else if (sostituzione == "G132E") {presence_in_dataset = "    Author  Year  Type  -DGJ  +DGJ  Link    Lukas et al.  2013  HEK293H  0  0   go    ";}
else if (sostituzione == "G132R") {presence_in_dataset = "    Author  Year  Type  -DGJ  +DGJ  Link    Lukas et al.  2013  HEK293H  0  0   go     Shin et al.  2008  T-Cell-Sample-1  1 ± 1  2 ± 0   go     Shin et al.  2007  T-Cell-Sample-1  1.3 ± 1.1  2.1 ± 0.4   go    ";}
else if (sostituzione == "G138E") {presence_in_dataset = "    Author  Year  Type  -DGJ  +DGJ  Link    Wu et al.  2011  HEK293-Sample-1  0  0   go     Benjamin et al.  2009  Lymphoblasts  0  0   go    ";}
else if (sostituzione == "G138R") {presence_in_dataset = "    Author  Year  Type  -DGJ  +DGJ  Link    Lukas et al.  2013  HEK293H  0  0   go    ";}
else if (sostituzione == "G144D") {presence_in_dataset = "    Author  Year  Type  -DGJ  +DGJ  Link    Benjamin et al.  2016  HEK-293  50.2 ± 2.2  76.5 ± 1.7   go    ";}
else if (sostituzione == "G144V") {presence_in_dataset = "    Author  Year  Type  -DGJ  +DGJ  Link    Benjamin et al.  2016  HEK-293  0.8 ± 0.1  9.2 ± 1.2   go     Wu et al.  2011  HEK293-Sample-1  0  19.5 ± 2.6   go     Benjamin et al.  2009  Lymphoblasts  0.1 ± 0.1  20   go    ";}
else if (sostituzione == "G147R") {presence_in_dataset = "    Author  Year  Type  -DGJ  +DGJ  Link    Lukas et al.  2013  HEK293H  0  0   go    ";}
else if (sostituzione == "G163V") {presence_in_dataset = "    Author  Year  Type  -DGJ  +DGJ  Link    Benjamin et al.  2016  HEK-293  1.4 ± 0.1  2.9 ± 0.1   go    ";}
else if (sostituzione == "G171D") {presence_in_dataset = "    Author  Year  Type  -DGJ  +DGJ  Link    Wu et al.  2011  HEK293-Sample-1  0  0   go     Wu et al.  2011  HEK293-Sample-2  0  0   go     Benjamin et al.  2009  Lymphoblasts  0  0   go    ";}
else if (sostituzione == "G171R") {presence_in_dataset = "    Author  Year  Type  -DGJ  +DGJ  Link    Benjamin et al.  2016  HEK-293  4.9 ± 0.2  6.4 ± 0.5   go    ";}
else if (sostituzione == "G183A") {presence_in_dataset = "    Author  Year  Type  -DGJ  +DGJ  Link    Benjamin et al.  2016  HEK-293  22.4 ± 1.9  56.4 ± 2.7   go     Filoni et al.  2010  COS-1  34  65.5   go     Filoni et al.  2010  Lymphocytes  46  92   go    ";}
else if (sostituzione == "G183D") {presence_in_dataset = "    Author  Year  Type  -DGJ  +DGJ  Link    Benjamin et al.  2016  HEK-293  0.7 ± 0.1  19.1 ± 1.0   go     Wu et al.  2011  HEK293-Sample-1  0  75 ± 4.2   go     Benjamin et al.  2009  Lymphoblasts  4 ± 2  23   go    ";}
else if (sostituzione == "G183S") {presence_in_dataset = "    Author  Year  Type  -DGJ  +DGJ  Link    Wu et al.  2011  HEK293-Sample-1  5.9 ± 0.5  84.9 ± 12.5   go     Benjamin et al.  2009  Lymphoblasts  0  0   go    ";}
else if (sostituzione == "G183V") {presence_in_dataset = "    Author  Year  Type  -DGJ  +DGJ  Link    Benjamin et al.  2016  HEK-293  NR  2.5 ± 0.1   go     Lukas et al.  2013  HEK293H  0  66.7 ± 2.1   go    ";}
else if (sostituzione == "G195V") {presence_in_dataset = "    Author  Year  Type  -DGJ  +DGJ  Link    Benjamin et al.  2016  HEK-293  25.8 ± 1.3  48.2 ± 1.5   go    ";}
else if (sostituzione == "G258R") {presence_in_dataset = "    Author  Year  Type  -DGJ  +DGJ  Link    Benjamin et al.  2016  HEK-293  32.6 ± 2.1  78.1 ± 5.8   go    ";}
else if (sostituzione == "G258V") {presence_in_dataset = "    Author  Year  Type  -DGJ  +DGJ  Link    Benjamin et al.  2016  HEK-293  7.7 ± 0.3  39.8 ± 1.7   go     Shimotori et al.  2007  COS-7  1.9  10.2   go    ";}
else if (sostituzione == "G260A") {presence_in_dataset = "    Author  Year  Type  -DGJ  +DGJ  Link    Benjamin et al.  2016  HEK-293  7.5 ± 0.6  37.4 ± 3.1   go     Shimotori et al.  2007  COS-7  10.8  59.2   go    ";}
else if (sostituzione == "G260E") {presence_in_dataset = "    Author  Year  Type  -DGJ  +DGJ  Link    Benjamin et al.  2016  HEK-293  10.6 ± 0.6  41.1 ± 1.3   go    ";}
else if (sostituzione == "G261D") {presence_in_dataset = "    Author  Year  Type  -DGJ  +DGJ  Link    Benjamin et al.  2016  HEK-293  NR  2.1 ± 0.2   go     Shin et al.  2008  T-Cell-Sample-1  7 ± 7  5 ± 4   go    ";}
else if (sostituzione == "G261V") {presence_in_dataset = "    Author  Year  Type  -DGJ  +DGJ  Link    Lukas et al.  2013  HEK293H  0.2 ± 1.4  3.5 ± 1.1   go    ";}
else if (sostituzione == "G271C") {presence_in_dataset = "    Author  Year  Type  -DGJ  +DGJ  Link    Benjamin et al.  2016  HEK-293  NR  0.5 ± 0.0   go     Wu et al.  2011  HEK293-Sample-1  0  0   go     Wu et al.  2011  HEK293-Sample-2  0  0   go     Shin et al.  2008  T-Cell-Sample-1  3 ± 1  4 ± 1   go     Shin et al.  2008  T-Cell-Sample-2  1 ± 1  2 ± 1   go    ";}
else if (sostituzione == "G271D") {presence_in_dataset = "    Author  Year  Type  -DGJ  +DGJ  Link    Benjamin et al.  2016  HEK-293  1.5 ± 0.1  32.2 ± 2   go    ";}
else if (sostituzione == "G271S") {presence_in_dataset = "    Author  Year  Type  -DGJ  +DGJ  Link    Benjamin et al.  2016  HEK-293  3.8 ± 0.1  31.9 ± 1.3   go    ";}
else if (sostituzione == "G271V") {presence_in_dataset = "    Author  Year  Type  -DGJ  +DGJ  Link    Wu et al.  2011  HEK293-Sample-1  0  0   go     Benjamin et al.  2009  Lymphoblasts  0  0   go    ";}
else if (sostituzione == "G325D") {presence_in_dataset = "    Author  Year  Type  -DGJ  +DGJ  Link    Benjamin et al.  2016  HEK-293  NR  1.0 ± 0.1   go     Wu et al.  2011  HEK293-Sample-1  0  26.2 ± 7.2   go     Benjamin et al.  2009  Lymphoblasts  3 ± 0.4  6   go    ";}
else if (sostituzione == "G325R") {presence_in_dataset = "    Author  Year  Type  -DGJ  +DGJ  Link    Benjamin et al.  2016  HEK-293  2.6 ± 0.1  26.6 ± 1.4   go    ";}
else if (sostituzione == "G325S") {presence_in_dataset = "    Author  Year  Type  -DGJ  +DGJ  Link    Benjamin et al.  2016  HEK-293  24.7 ± 1.1  62.5 ± 2.0   go     Lukas et al.  2013  HEK293H  25.6 ± 9.6  55.4 ± 14.4   go    ";}
else if (sostituzione == "G328A") {presence_in_dataset = "    Author  Year  Type  -DGJ  +DGJ  Link    Benjamin et al.  2016  HEK-293  6.9 ± 0.3  28.7 ± 1.0   go     Lukas et al.  2013  HEK293H  6.2 ± 1  30 ± 6.4   go     Wu et al.  2011  HEK293-Sample-1  3.9 ± 0.1  79 ± 5.7   go     Wu et al.  2011  HEK293-Sample-2  3.9  22.1   go     Benjamin et al.  2009  Lymphoblasts  9 ± 1  50   go     Shin et al.  2008  T-Cell-Sample-1  2 ± 1  54 ± 13   go    ";}
else if (sostituzione == "G328R") {presence_in_dataset = "    Author  Year  Type  -DGJ  +DGJ  Link    Lukas et al.  2016  HEK293H  0  0   go     Shimotori et al.  2007  COS-7  0.2  0.2   go    ";}
else if (sostituzione == "G334E") {presence_in_dataset = "    Author  Year  Type  -DGJ  +DGJ  Link    Benjamin et al.  2016  HEK-293  86.7 ± 3.4  105.5 ± 3.7   go    ";}
else if (sostituzione == "G35E") {presence_in_dataset = "    Author  Year  Type  -DGJ  +DGJ  Link    Lukas et al.  2016  HEK293H  38 ± 7.8  70.8 ± 14.6   go    ";}
else if (sostituzione == "G35R") {presence_in_dataset = "    Author  Year  Type  -DGJ  +DGJ  Link    Benjamin et al.  2016  HEK-293  19.2 ± 1.0  66.1 ± 3.4   go     Lukas et al.  2013  HEK293H  17.9 ± 2.1  72.3 ± 4.7   go    ";}
else if (sostituzione == "G360C") {presence_in_dataset = "    Author  Year  Type  -DGJ  +DGJ  Link    Benjamin et al.  2016  HEK-293  8.7 ± 0.5  16.8 ± 0.7   go     Lukas et al.  2013  HEK293H  11.9 ± 0.5  26.5 ± 2.7   go    ";}
else if (sostituzione == "G360D") {presence_in_dataset = "    Author  Year  Type  -DGJ  +DGJ  Link    Benjamin et al.  2016  HEK-293  1.0 ± 0.1  7.2 ± 0.4   go    ";}
else if (sostituzione == "G360S") {presence_in_dataset = "    Author  Year  Type  -DGJ  +DGJ  Link    Benjamin et al.  2016  HEK-293  10.0 ± 0.6  22.1 ± 0.9   go     Lukas et al.  2016  HEK293H  0  0.4 ± 0.2   go    ";}
else if (sostituzione == "G361A") {presence_in_dataset = "    Author  Year  Type  -DGJ  +DGJ  Link    Benjamin et al.  2016  HEK-293  7.2 ± 0.3  30.0 ± 1.3   go    ";}
else if (sostituzione == "G361E") {presence_in_dataset = "    Author  Year  Type  -DGJ  +DGJ  Link    Benjamin et al.  2016  HEK-293  2.6 ± 0.1  13.4 ± 1   go    ";}
else if (sostituzione == "G361R") {presence_in_dataset = "    Author  Year  Type  -DGJ  +DGJ  Link    Benjamin et al.  2016  HEK-293  0.5 ± 0.0  3.0 ± 0.1   go     Lukas et al.  2013  HEK293H  0  3.7 ± 1.1   go    ";}
else if (sostituzione == "G373D") {presence_in_dataset = "    Author  Year  Type  -DGJ  +DGJ  Link    Lukas et al.  2013  HEK293H  0  0   go    ";}
else if (sostituzione == "G373S") {presence_in_dataset = "    Author  Year  Type  -DGJ  +DGJ  Link    Benjamin et al.  2016  HEK-293  4.8 ± 0.3  15.7 ± 0.8   go    ";}
else if (sostituzione == "G375A") {presence_in_dataset = "    Author  Year  Type  -DGJ  +DGJ  Link    Lukas et al.  2016  HEK293H  44.1 ± 10.1  48.8 ± 12.6   go    ";}
else if (sostituzione == "G395A") {presence_in_dataset = "    Author  Year  Type  -DGJ  +DGJ  Link    Benjamin et al.  2016  HEK-293  24.4 ± 1.3  30.7 ± 1.0   go     Lukas et al.  2013  HEK293H  20.1 ± 1.7  23.1 ± 2   go    ";}
else if (sostituzione == "G395E") {presence_in_dataset = "    Author  Year  Type  -DGJ  +DGJ  Link    Benjamin et al.  2016  HEK-293  11.9 ± 1.3  19.7 ± 2.2   go    ";}
else if (sostituzione == "G411D") {presence_in_dataset = "    Author  Year  Type  -DGJ  +DGJ  Link    Benjamin et al.  2016  HEK-293  1.1 ± 0.1  9.6 ± 0.4   go    ";}
else if (sostituzione == "G43S") {presence_in_dataset = "    Author  Year  Type  -DGJ  +DGJ  Link    Lukas et al.  2013  HEK293H  0  0   go    ";}
else if (sostituzione == "G80D") {presence_in_dataset = "    Author  Year  Type  -DGJ  +DGJ  Link    Lukas et al.  2016  HEK293H  29.3 ± 5.7  30.4 ± 5.4   go    ";}
else if (sostituzione == "G85D") {presence_in_dataset = "    Author  Year  Type  -DGJ  +DGJ  Link    Benjamin et al.  2016  HEK-293  2.7 ± 0.1  14.3 ± 0.9   go    ";}
else if (sostituzione == "G85M") {presence_in_dataset = "    Author  Year  Type  -DGJ  +DGJ  Link    Benjamin et al.  2016  HEK-293  7.9 ± 0.5  11.8 ± 0.7   go    ";}
else if (sostituzione == "G85S") {presence_in_dataset = "    Author  Year  Type  -DGJ  +DGJ  Link    Benjamin et al.  2016  HEK-293  12.4 ± 0.8  19.1 ± 0.8   go    ";}
else if (sostituzione == "H125L") {presence_in_dataset = "    Author  Year  Type  -DGJ  +DGJ  Link    Benjamin et al.  2016  HEK-293  1.4 ± 0.1  4.7 ± 0.4   go    ";}
else if (sostituzione == "H125P") {presence_in_dataset = "    Author  Year  Type  -DGJ  +DGJ  Link    Lukas et al.  2013  HEK293H  0  0   go    ";}
else if (sostituzione == "H225D") {presence_in_dataset = "    Author  Year  Type  -DGJ  +DGJ  Link    Benjamin et al.  2016  HEK-293  43.8 ± 2.6  110.6 ± 7.2   go     Lukas et al.  2013  HEK293H  32.2 ± 8.1  60.5 ± 19.1   go    ";}
else if (sostituzione == "H225R") {presence_in_dataset = "    Author  Year  Type  -DGJ  +DGJ  Link    Benjamin et al.  2016  HEK-293  NR  2.0 ± 0.1   go     Lukas et al.  2013  HEK293H  0  3 ± 0.8   go     Wu et al.  2011  HEK293-Sample-1  0  15.1 ± 1   go     Wu et al.  2011  HEK293-Sample-2  0  0   go     Shin et al.  2008  T-Cell-Sample-1  5 ± 3  5 ± 1   go    ";}
else if (sostituzione == "H406R") {presence_in_dataset = "    Author  Year  Type  -DGJ  +DGJ  Link    Benjamin et al.  2016  HEK-293  74.2 ± 1.6  73.2 ± 1.7   go    ";}
else if (sostituzione == "H46P") {presence_in_dataset = "    Author  Year  Type  -DGJ  +DGJ  Link    Benjamin et al.  2016  HEK-293  31.0 ± 1.2  106.9 ± 4.4   go     Lukas et al.  2013  HEK293H  40.1 ± 1.2  98.8 ± 2.1   go    ";}
else if (sostituzione == "H46R") {presence_in_dataset = "    Author  Year  Type  -DGJ  +DGJ  Link    Wu et al.  2011  HEK293-Sample-1  0  0   go     Benjamin et al.  2009  Lymphoblasts  0  0   go    ";}
else if (sostituzione == "H46Y") {presence_in_dataset = "    Author  Year  Type  -DGJ  +DGJ  Link    Wu et al.  2011  HEK293-Sample-1  0  0   go     Benjamin et al.  2009  Lymphoblasts  0  0   go    ";}
else if (sostituzione == "I117S") {presence_in_dataset = "    Author  Year  Type  -DGJ  +DGJ  Link    Benjamin et al.  2016  HEK-293  0.7 ± 0.0  1.5 ± 0.1   go    ";}
else if (sostituzione == "I154T") {presence_in_dataset = "    Author  Year  Type  -DGJ  +DGJ  Link    Lukas et al.  2016  HEK293H  98 ± 16.4  108 ± 18.6   go    ";}
else if (sostituzione == "I198T") {presence_in_dataset = "    Author  Year  Type  -DGJ  +DGJ  Link    Benjamin et al.  2016  HEK-293  64.7 ± 3.5  95.5 ± 4.8   go     Lukas et al.  2016  HEK293H  38.7 ± 3.1  50.4 ± 3.2   go    ";}
else if (sostituzione == "I219M") {presence_in_dataset = "    Author  Year  Type  -DGJ  +DGJ  Link    Lukas et al.  2016  HEK293H  15.2 ± 2.2  56.5 ± 9.4   go    ";}
else if (sostituzione == "I219N") {presence_in_dataset = "    Author  Year  Type  -DGJ  +DGJ  Link    Benjamin et al.  2016  HEK-293  0.6 ± 0.1  12.9 ± 1.1   go    ";}
else if (sostituzione == "I219T") {presence_in_dataset = "    Author  Year  Type  -DGJ  +DGJ  Link    Benjamin et al.  2016  HEK-293  55.8 ± 2.9  93.6 ± 3.7   go     Lukas et al.  2013  HEK293H  53.3 ± 2.2  85.3 ± 10   go    ";}
else if (sostituzione == "I232T") {presence_in_dataset = "    Author  Year  Type  -DGJ  +DGJ  Link    Benjamin et al.  2016  HEK-293  15.0 ± 1.6  85.0 ± 4.1   go     Lukas et al.  2013  HEK293H  11.5 ± 2.8  61.6 ± 7.2   go    ";}
else if (sostituzione == "I239T") {presence_in_dataset = "    Author  Year  Type  -DGJ  +DGJ  Link    Benjamin et al.  2016  HEK-293  37.7 ± 1.9  92.8 ± 3.9   go    ";}
else if (sostituzione == "I242F") {presence_in_dataset = "    Author  Year  Type  -DGJ  +DGJ  Link    Benjamin et al.  2016  HEK-293  1.1 ± 0.1  9.0 ± 0.8   go    ";}
else if (sostituzione == "I242N") {presence_in_dataset = "    Author  Year  Type  -DGJ  +DGJ  Link    Benjamin et al.  2016  HEK-293  7.6 ± 0.3  67.4 ± 4.2   go     Lukas et al.  2013  HEK293H  3.1 ± 1.4  49.8 ± 13.9   go    ";}
else if (sostituzione == "I242V") {presence_in_dataset = "    Author  Year  Type  -DGJ  +DGJ  Link    Lukas et al.  2016  HEK293H  70.6 ± 15.7  89.4 ± 21.2   go    ";}
else if (sostituzione == "I253S") {presence_in_dataset = "    Author  Year  Type  -DGJ  +DGJ  Link    Benjamin et al.  2016  HEK-293  3.3 ± 0.2  31.2 ± 1.4   go     Lukas et al.  2013  HEK293H  4.4 ± 1.6  53.4 ± 7.1   go    ";}
else if (sostituzione == "I253T") {presence_in_dataset = "    Author  Year  Type  -DGJ  +DGJ  Link    Benjamin et al.  2016  HEK-293  38.9 ± 3.0  80.2 ± 5.9   go     Lukas et al.  2016  HEK293H  73 ± 4.8  115.8 ± 6.9   go    ";}
else if (sostituzione == "I270M") {presence_in_dataset = "    Author  Year  Type  -DGJ  +DGJ  Link    Lukas et al.  2013  HEK293H  2.3 ± 1.7  33.7 ± 16.8   go    ";}
else if (sostituzione == "I270T") {presence_in_dataset = "    Author  Year  Type  -DGJ  +DGJ  Link    Benjamin et al.  2016  HEK-293  6.3 ± 0.5  42.8 ± 3.0   go    ";}
else if (sostituzione == "I289F") {presence_in_dataset = "    Author  Year  Type  -DGJ  +DGJ  Link    Benjamin et al.  2016  HEK-293  NR  0.8 ± 0.1   go     Wu et al.  2011  HEK293-Sample-1  0  14.3 ± 1.4   go     Benjamin et al.  2009  Lymphoblasts  4 ± 1  20   go    ";}
else if (sostituzione == "I289S") {presence_in_dataset = "    Author  Year  Type  -DGJ  +DGJ  Link    Benjamin et al.  2016  HEK-293  NR  3.0 ± 0.2   go    ";}
else if (sostituzione == "I289V") {presence_in_dataset = "    Author  Year  Type  -DGJ  +DGJ  Link    Lukas et al.  2016  HEK293H  79.9 ± 6.4  95 ± 4.3   go    ";}
else if (sostituzione == "I303N") {presence_in_dataset = "    Author  Year  Type  -DGJ  +DGJ  Link    Benjamin et al.  2016  HEK-293  NR  6.2 ± 0.3   go    ";}
else if (sostituzione == "I317N") {presence_in_dataset = "    Author  Year  Type  -DGJ  +DGJ  Link    Benjamin et al.  2016  HEK-293  NR  2.9 ± 0.2   go    ";}
else if (sostituzione == "I317S") {presence_in_dataset = "    Author  Year  Type  -DGJ  +DGJ  Link    Benjamin et al.  2016  HEK-293  NR  0.8 ± 0   go     Lukas et al.  2016  HEK293H  0  2.7 ± 0.7   go    ";}
else if (sostituzione == "I317T") {presence_in_dataset = "    Author  Year  Type  -DGJ  +DGJ  Link    Benjamin et al.  2016  HEK-293  6.5 ± 0.6  23.6 ± 1.0   go    ";}
else if (sostituzione == "I319F") {presence_in_dataset = "    Author  Year  Type  -DGJ  +DGJ  Link    Benjamin et al.  2016  HEK-293  2.6 ± 0.1  19.6 ± 0.7   go    ";}
else if (sostituzione == "I319T") {presence_in_dataset = "    Author  Year  Type  -DGJ  +DGJ  Link    Benjamin et al.  2016  HEK-293  10.3 ± 0.6  28.0 ± 1.2   go     Lukas et al.  2013  HEK293H  20.2 ± 3.8  58.3 ± 12.6   go    ";}
else if (sostituzione == "I354K") {presence_in_dataset = "    Author  Year  Type  -DGJ  +DGJ  Link    Benjamin et al.  2016  HEK-293  1.7 ± 0.1  21.6 ± 0.9   go    ";}
else if (sostituzione == "I359T") {presence_in_dataset = "    Author  Year  Type  -DGJ  +DGJ  Link    Benjamin et al.  2016  HEK-293  33.7 ± 1.7  63.9 ± 2.0   go    ";}
else if (sostituzione == "I384N") {presence_in_dataset = "    Author  Year  Type  -DGJ  +DGJ  Link    Benjamin et al.  2016  HEK-293  NR  0.8 ± 0.0   go     Lukas et al.  2013  HEK293H  0  0   go    ";}
else if (sostituzione == "I407V") {presence_in_dataset = "    Author  Year  Type  -DGJ  +DGJ  Link    Benjamin et al.  2016  HEK-293  85.6 ± 2.8  97.7 ± 2.3   go    ";}
else if (sostituzione == "I64F") {presence_in_dataset = "    Author  Year  Type  -DGJ  +DGJ  Link    Lukas et al.  2016  HEK293H  0  0   go    ";}
else if (sostituzione == "I91N") {presence_in_dataset = "    Author  Year  Type  -DGJ  +DGJ  Link    Lukas et al.  2016  HEK293H  0  0   go    ";}
else if (sostituzione == "I91T") {presence_in_dataset = "    Author  Year  Type  -DGJ  +DGJ  Link    Benjamin et al.  2016  HEK-293  0.9 ± 0.1  12.6 ± 0.5   go     Lukas et al.  2016  HEK293H  0.7 ± 0.3  7 ± 1   go     Wu et al.  2011  HEK293-Sample-1  0  5.1 ± 0.8   go     Benjamin et al.  2009  Lymphoblasts  8 ± 3  31   go     Park et al.  2009  COS-7  12 ± 0.69  35.7 ± 1.48   go     Ishii et al.  2007  Fibroblasts  6.9  22.6   go    ";}
else if (sostituzione == "K130R") {presence_in_dataset = "    Author  Year  Type  -DGJ  +DGJ  Link    Benjamin et al.  2016  HEK-293  63.0 ± 2.7  73.7 ± 3.9   go    ";}
else if (sostituzione == "K140T") {presence_in_dataset = "    Author  Year  Type  -DGJ  +DGJ  Link    Benjamin et al.  2016  HEK-293  51.4 ± 2.1  89.8 ± 2.7   go    ";}
else if (sostituzione == "K185E") {presence_in_dataset = "    Author  Year  Type  -DGJ  +DGJ  Link    Benjamin et al.  2016  HEK-293  39.6 ± 3.1  71.3 ± 2.9   go    ";}
else if (sostituzione == "K213M") {presence_in_dataset = "    Author  Year  Type  -DGJ  +DGJ  Link    Benjamin et al.  2016  HEK-293  43.2 ± 1.4  55.9 ± 1.8   go     Lukas et al.  2013  HEK293H  83.4 ± 29.6  82.5 ± 15.5   go    ";}
else if (sostituzione == "K213R") {presence_in_dataset = "    Author  Year  Type  -DGJ  +DGJ  Link    Lukas et al.  2016  HEK293H  68.1 ± 8.5  65.3 ± 11.4   go    ";}
else if (sostituzione == "K308N") {presence_in_dataset = "    Author  Year  Type  -DGJ  +DGJ  Link    Benjamin et al.  2016  HEK-293  3.7 ± 0.2  17.6 ± 0.8   go     Shimotori et al.  2007  COS-7  4.6  46.9   go    ";}
else if (sostituzione == "K391T") {presence_in_dataset = "    Author  Year  Type  -DGJ  +DGJ  Link    Benjamin et al.  2016  HEK-293  30.1 ± 1.4  47.4 ± 1.5   go    ";}
else if (sostituzione == "L120P") {presence_in_dataset = "    Author  Year  Type  -DGJ  +DGJ  Link    Benjamin et al.  2016  HEK-293  NR  0.5 ± 0.0   go    ";}
else if (sostituzione == "L120S") {presence_in_dataset = "    Author  Year  Type  -DGJ  +DGJ  Link    Benjamin et al.  2016  HEK-293  NR  2.4 ± 0.1   go    ";}
else if (sostituzione == "L120V") {presence_in_dataset = "    Author  Year  Type  -DGJ  +DGJ  Link    Benjamin et al.  2016  HEK-293  66.8 ± 3.1  74.7 ± 2.4   go     Lukas et al.  2013  HEK293H  50.1 ± 5  62 ± 2.6   go    ";}
else if (sostituzione == "L129P") {presence_in_dataset = "    Author  Year  Type  -DGJ  +DGJ  Link    Lukas et al.  2013  HEK293H  0  0   go    ";}
else if (sostituzione == "L131P") {presence_in_dataset = "    Author  Year  Type  -DGJ  +DGJ  Link    Lukas et al.  2013  HEK293H  0  0   go     Wu et al.  2011  HEK293-Sample-1  0  0   go     Benjamin et al.  2009  Lymphoblasts  0  0   go    ";}
else if (sostituzione == "L14P") {presence_in_dataset = "    Author  Year  Type  -DGJ  +DGJ  Link    Benjamin et al.  2016  HEK-293  NR  0.5 ± 0.1   go    ";}
else if (sostituzione == "L166G") {presence_in_dataset = "    Author  Year  Type  -DGJ  +DGJ  Link    Benjamin et al.  2016  HEK-293  10.2 ± 0.6  33.0 ± 1.6   go    ";}
else if (sostituzione == "L166V") {presence_in_dataset = "    Author  Year  Type  -DGJ  +DGJ  Link    Benjamin et al.  2016  HEK-293  3.8 ± 0.2  20.9 ± 1.1   go    ";}
else if (sostituzione == "L167Q") {presence_in_dataset = "    Author  Year  Type  -DGJ  +DGJ  Link    Benjamin et al.  2016  HEK-293  NR  0.6 ± 0.1   go     Lukas et al.  2016  HEK293H  0  0.7 ± 0.3   go    ";}
else if (sostituzione == "L180F") {presence_in_dataset = "    Author  Year  Type  -DGJ  +DGJ  Link    Lukas et al.  2016  HEK293H  32.4 ± 9.3  80.7 ± 15.6   go    ";}
else if (sostituzione == "L191P") {presence_in_dataset = "    Author  Year  Type  -DGJ  +DGJ  Link    Lukas et al.  2013  HEK293H  0  0   go    ";}
else if (sostituzione == "L191Q") {presence_in_dataset = "    Author  Year  Type  -DGJ  +DGJ  Link    Benjamin et al.  2016  HEK-293  0.6 ± 0.0  9.0 ± 1.2   go    ";}
else if (sostituzione == "L206P") {presence_in_dataset = "    Author  Year  Type  -DGJ  +DGJ  Link    Benjamin et al.  2016  HEK-293  NR  0.7 ± 0.1   go    ";}
else if (sostituzione == "L21F") {presence_in_dataset = "    Author  Year  Type  -DGJ  +DGJ  Link    Benjamin et al.  2016  HEK-293  71.4 ± 2.9  75.9 ± 3.0   go    ";}
else if (sostituzione == "L21P") {presence_in_dataset = "    Author  Year  Type  -DGJ  +DGJ  Link    Benjamin et al.  2016  HEK-293  1.1 ± 0.1  1.6 ± 0.1   go     Lukas et al.  2016  HEK293H  0.6 ± 0.1  1.7 ± 0.3   go    ";}
else if (sostituzione == "L21R") {presence_in_dataset = "    Author  Year  Type  -DGJ  +DGJ  Link    Benjamin et al.  2016  HEK-293  1.2 ± 0.1  1.8 ± 0.1   go    ";}
else if (sostituzione == "L243F") {presence_in_dataset = "    Author  Year  Type  -DGJ  +DGJ  Link    Benjamin et al.  2016  HEK-293  7.9 ± 0.3  42.3 ± 1.4   go     Lukas et al.  2016  HEK293H  11.4 ± 2  70.8 ± 10.5   go    ";}
else if (sostituzione == "L243W") {presence_in_dataset = "    Author  Year  Type  -DGJ  +DGJ  Link    Benjamin et al.  2016  HEK-293  0.6 ± 0.1  14.3 ± 0.9   go    ";}
else if (sostituzione == "L268S") {presence_in_dataset = "    Author  Year  Type  -DGJ  +DGJ  Link    Benjamin et al.  2016  HEK-293  NR  2.8 ± 0.1   go     Lukas et al.  2013  HEK293H  0  10.8 ± 0.7   go    ";}
else if (sostituzione == "L275F") {presence_in_dataset = "    Author  Year  Type  -DGJ  +DGJ  Link    Lukas et al.  2013  HEK293H  0  0   go    ";}
else if (sostituzione == "L275H") {presence_in_dataset = "    Author  Year  Type  -DGJ  +DGJ  Link    Lukas et al.  2013  HEK293H  0  0   go    ";}
else if (sostituzione == "L294S") {presence_in_dataset = "    Author  Year  Type  -DGJ  +DGJ  Link    Benjamin et al.  2016  HEK-293  NR  4.9 ± 0.1   go     Lukas et al.  2013  HEK293H  0  12.4 ± 1.7   go    ";}
else if (sostituzione == "L300F") {presence_in_dataset = "    Author  Year  Type  -DGJ  +DGJ  Link    Benjamin et al.  2016  HEK-293  14.8 ± 0.9  50.5 ± 1.4   go     Andreotti et al.  2011  COS-7  8.7 ± 1.5  50.84 ± 9.4   go    ";}
else if (sostituzione == "L300P") {presence_in_dataset = "    Author  Year  Type  -DGJ  +DGJ  Link    Benjamin et al.  2016  HEK-293  3.7 ± 0.1  37.9 ± 1.2   go     Wu et al.  2011  HEK293-Sample-1  2.9 ± 0.9  62.9 ± 9   go     Wu et al.  2011  HEK293-Sample-2  2.9  25.6   go     Benjamin et al.  2009  Lymphoblasts  4 ± 1  63   go     Benjamin et al.  2009  Fibroblasts  8 ± 3  57.1   go     Shin et al.  2008  T-Cell-Sample-1  2 ± 3  72 ± 25   go     Shin et al.  2007  T-Cell-Sample-1  2 ± 2.7  72.1 ± 25.3   go    ";}
else if (sostituzione == "L310F") {presence_in_dataset = "    Author  Year  Type  -DGJ  +DGJ  Link    Benjamin et al.  2016  HEK-293  0.8 ± 0.1  11.6 ± 1.2   go     Lukas et al.  2013  HEK293H  0  4.1 ± 1.1   go     Andreotti et al.  2011  COS-7  4 ± 1.4  35.4 ± 9.5   go    ";}
else if (sostituzione == "L310R") {presence_in_dataset = "    Author  Year  Type  -DGJ  +DGJ  Link    Benjamin et al.  2016  HEK-293  NR  0.7 ± 0.0   go    ";}
else if (sostituzione == "L311F") {presence_in_dataset = "    Author  Year  Type  -DGJ  +DGJ  Link    Benjamin et al.  2016  HEK-293  NR  2 ± 0.1   go    ";}
else if (sostituzione == "L311V") {presence_in_dataset = "    Author  Year  Type  -DGJ  +DGJ  Link    Benjamin et al.  2016  HEK-293  2 ± 0.1  18 ± 0.8   go     Lukas et al.  2013  HEK293H  1.9 ± 0.4  40.1 ± 4.9   go    ";}
else if (sostituzione == "L32P") {presence_in_dataset = "    Author  Year  Type  -DGJ  +DGJ  Link    Benjamin et al.  2016  HEK-293  7.3 ± 0.4  36.3 ± 2.0   go     Giugliani et al.  2013  HEK-293  7.3 ± 0.4  36.3 ± 2   go    ";}
else if (sostituzione == "L344P") {presence_in_dataset = "    Author  Year  Type  -DGJ  +DGJ  Link    Lukas et al.  2013  HEK293H  0  0   go    ";}
else if (sostituzione == "L36F") {presence_in_dataset = "    Author  Year  Type  -DGJ  +DGJ  Link    Benjamin et al.  2016  HEK-293  4.0 ± 0.3  35.9 ± 1.7   go    ";}
else if (sostituzione == "L36S") {presence_in_dataset = "    Author  Year  Type  -DGJ  +DGJ  Link    Benjamin et al.  2016  HEK-293  32.5 ± 1.6  76.9 ± 3.8   go     Lukas et al.  2013  HEK293H  0  0   go    ";}
else if (sostituzione == "L36W") {presence_in_dataset = "    Author  Year  Type  -DGJ  +DGJ  Link    Benjamin et al.  2016  HEK-293  0.7 ± 0.1  16.6 ± 2.1   go     Lukas et al.  2016  HEK293H  2.3 ± 0.6  22.3 ± 1.5   go    ";}
else if (sostituzione == "L372P") {presence_in_dataset = "    Author  Year  Type  -DGJ  +DGJ  Link    Benjamin et al.  2016  HEK-293  1.2 ± 0.1  2.6 ± 0.1   go     Lukas et al.  2013  HEK293H  0  2.6 ± 0.7   go    ";}
else if (sostituzione == "L372Q") {presence_in_dataset = "    Author  Year  Type  -DGJ  +DGJ  Link    Benjamin et al.  2016  HEK-293  0.8 ± 0.1  1.6 ± 0.1   go    ";}
else if (sostituzione == "L372R") {presence_in_dataset = "    Author  Year  Type  -DGJ  +DGJ  Link    Lukas et al.  2013  HEK293H  0  0   go    ";}
else if (sostituzione == "L3P") {presence_in_dataset = "    Author  Year  Type  -DGJ  +DGJ  Link    Benjamin et al.  2016  HEK-293  71.9 ± 4.5  92.2 ± 4.5   go     Lukas et al.  2016  HEK293H  117.7 ± 13.7  129.4 ± 12.6   go    ";}
else if (sostituzione == "L3V") {presence_in_dataset = "    Author  Year  Type  -DGJ  +DGJ  Link    Lukas et al.  2016  HEK293H  81.5 ± 9.2  88 ± 12.4   go    ";}
else if (sostituzione == "L403S") {presence_in_dataset = "    Author  Year  Type  -DGJ  +DGJ  Link    Benjamin et al.  2016  HEK-293  14.0 ± 0.5  19.0 ± 0.5   go     Shimotori et al.  2007  COS-7  22.7  38.2   go    ";}
else if (sostituzione == "L414S") {presence_in_dataset = "    Author  Year  Type  -DGJ  +DGJ  Link    Benjamin et al.  2016  HEK-293  NR  1.2 ± 0.1   go    ";}
else if (sostituzione == "L415F") {presence_in_dataset = "    Author  Year  Type  -DGJ  +DGJ  Link    Benjamin et al.  2016  HEK-293  91.8 ± 5.1  103.3 ± 7.2   go     Lukas et al.  2013  HEK293H  83.2 ± 5.9  99.5 ± 12.2   go    ";}
else if (sostituzione == "L415P") {presence_in_dataset = "    Author  Year  Type  -DGJ  +DGJ  Link    Lukas et al.  2013  HEK293H  0  0   go     Wu et al.  2011  HEK293-Sample-1  0  0   go     Wu et al.  2011  HEK293-Sample-2  0  0   go     Shin et al.  2008  T-Cell-Sample-1  7 ± 6  6 ± 3   go    ";}
else if (sostituzione == "L45P") {presence_in_dataset = "    Author  Year  Type  -DGJ  +DGJ  Link    Lukas et al.  2016  HEK293H  0  0   go    ";}
else if (sostituzione == "L45R") {presence_in_dataset = "    Author  Year  Type  -DGJ  +DGJ  Link    Benjamin et al.  2016  HEK-293  0.6 ± 0.1  0.6 ± 0.1   go    ";}
else if (sostituzione == "L54F") {presence_in_dataset = "    Author  Year  Type  -DGJ  +DGJ  Link    Benjamin et al.  2016  HEK-293  5 ± 0.2  41.4 ± 1.4   go    ";}
else if (sostituzione == "L54P") {presence_in_dataset = "    Author  Year  Type  -DGJ  +DGJ  Link    Benjamin et al.  2016  HEK-293  NR  11.2 ± 0.5   go    ";}
else if (sostituzione == "L68F") {presence_in_dataset = "    Author  Year  Type  -DGJ  +DGJ  Link    Benjamin et al.  2016  HEK-293  NR  0.8 ± 0.1   go     Lukas et al.  2013  HEK293H  0  4.5 ± 1.8   go    ";}
else if (sostituzione == "L89F") {presence_in_dataset = "    Author  Year  Type  -DGJ  +DGJ  Link    Benjamin et al.  2016  HEK-293  18.9 ± 0.8  37.8 ± 1.1   go    ";}
else if (sostituzione == "L89H") {presence_in_dataset = "    Author  Year  Type  -DGJ  +DGJ  Link    Lukas et al.  2013  HEK293H  0  0   go    ";}
else if (sostituzione == "L89R") {presence_in_dataset = "    Author  Year  Type  -DGJ  +DGJ  Link    Wu et al.  2011  HEK293-Sample-1  0  0   go     Benjamin et al.  2009  Lymphoblasts  0  0   go    ";}
else if (sostituzione == "M187I") {presence_in_dataset = "    Author  Year  Type  -DGJ  +DGJ  Link    Benjamin et al.  2016  HEK-293  5.1 ± 0.2  30.7 ± 1.1   go     Lukas et al.  2016  HEK293H  3.1 ± 0.6  31.2 ± 4.5   go    ";}
else if (sostituzione == "M187T") {presence_in_dataset = "    Author  Year  Type  -DGJ  +DGJ  Link    Benjamin et al.  2016  HEK-293  9.8 ± 1.0  27.4 ± 1.8   go     Lukas et al.  2013  HEK293H  0  0   go    ";}
else if (sostituzione == "M187V") {presence_in_dataset = "    Author  Year  Type  -DGJ  +DGJ  Link    Benjamin et al.  2016  HEK-293  20.1 ± 1.5  45.7 ± 2.8   go     Lukas et al.  2016  HEK293H  22.8 ± 5  67 ± 7.8   go    ";}
else if (sostituzione == "M1I") {presence_in_dataset = "    Author  Year  Type  -DGJ  +DGJ  Link    Benjamin et al.  2016  HEK-293  0.5 ± 0.0  0.6 ± 0.1   go    ";}
else if (sostituzione == "M1L") {presence_in_dataset = "    Author  Year  Type  -DGJ  +DGJ  Link    Benjamin et al.  2016  HEK-293  0.6 ± 0.1  0.8 ± 0.1   go     Giugliani et al.  2013  HEK-293  0  0   go    ";}
else if (sostituzione == "M1R") {presence_in_dataset = "    Author  Year  Type  -DGJ  +DGJ  Link    Benjamin et al.  2016  HEK-293  NR  0.6 ± 0.1   go    ";}
else if (sostituzione == "M1T") {presence_in_dataset = "    Author  Year  Type  -DGJ  +DGJ  Link    Benjamin et al.  2016  HEK-293  0.5 ± 0.0  0.7 ± 0.1   go    ";}
else if (sostituzione == "M1V") {presence_in_dataset = "    Author  Year  Type  -DGJ  +DGJ  Link    Benjamin et al.  2016  HEK-293  NR  0.5 ± 0.1   go    ";}
else if (sostituzione == "M267T") {presence_in_dataset = "    Author  Year  Type  -DGJ  +DGJ  Link    Benjamin et al.  2016  HEK-293  28.8 ± 0.9  45.3 ± 1.7   go     Lukas et al.  2013  HEK293H  27.5 ± 4.4  30.5 ± 1.8   go    ";}
else if (sostituzione == "M284T") {presence_in_dataset = "    Author  Year  Type  -DGJ  +DGJ  Link    Benjamin et al.  2016  HEK-293  1.7 ± 0.1  14.3 ± 0.6   go    ";}
else if (sostituzione == "M284V") {presence_in_dataset = "    Author  Year  Type  -DGJ  +DGJ  Link    Benjamin et al.  2016  HEK-293  25.2 ± 1.6  63.1 ± 2.4   go    ";}
else if (sostituzione == "M290I") {presence_in_dataset = "    Author  Year  Type  -DGJ  +DGJ  Link    Benjamin et al.  2016  HEK-293  68.0 ± 3.4  114.8 ± 6.3   go     Lukas et al.  2013  HEK293H  39 ± 1.8  70.5 ± 7.2   go    ";}
else if (sostituzione == "M290L") {presence_in_dataset = "    Author  Year  Type  -DGJ  +DGJ  Link    Benjamin et al.  2016  HEK-293  58.6 ± 3.2  111.4 ± 7.7   go     Lukas et al.  2013  HEK293H  18.5 ± 8.2  22.4 ± 9.6   go    ";}
else if (sostituzione == "M296I") {presence_in_dataset = "    Author  Year  Type  -DGJ  +DGJ  Link    Benjamin et al.  2016  HEK-293  15.7 ± 0.7  63.2 ± 4.2   go     Wu et al.  2011  HEK293-Sample-1  19.5 ± 3.5  118 ± 5   go     Benjamin et al.  2009  Lymphoblasts  17 ± 5  83   go     Ishii et al.  2007  Lymphoblasts  11.9  62.6   go    ";}
else if (sostituzione == "M296L") {presence_in_dataset = "    Author  Year  Type  -DGJ  +DGJ  Link    Benjamin et al.  2016  HEK-293  14.0 ± 0.8  65.4 ± 4.3   go    ";}
else if (sostituzione == "M296T") {presence_in_dataset = "    Author  Year  Type  -DGJ  +DGJ  Link    Benjamin et al.  2016  HEK-293  1.6 ± 0.1  26.9 ± 2.9   go    ";}
else if (sostituzione == "M296V") {presence_in_dataset = "    Author  Year  Type  -DGJ  +DGJ  Link    Benjamin et al.  2016  HEK-293  11.3 ± 0.6  55.4 ± 4.4   go     Wu et al.  2011  HEK293-Sample-1  18.3 ± 6.5  126 ± 14   go     Benjamin et al.  2009  Lymphoblasts  17 ± 2  74   go     Ishii et al.  2007  Fibroblasts  11.5  71.9   go    ";}
else if (sostituzione == "M421V") {presence_in_dataset = "    Author  Year  Type  -DGJ  +DGJ  Link    Benjamin et al.  2016  HEK-293  83.0 ± 3.4  108.6 ± 4.3   go    ";}
else if (sostituzione == "M42K") {presence_in_dataset = "    Author  Year  Type  -DGJ  +DGJ  Link    Benjamin et al.  2016  HEK-293  3.9 ± 0.1  29.7 ± 1.5   go    ";}
else if (sostituzione == "M42L") {presence_in_dataset = "    Author  Year  Type  -DGJ  +DGJ  Link    Benjamin et al.  2016  HEK-293  38.8 ± 2.0  60.2 ± 2.7   go    ";}
else if (sostituzione == "M42R") {presence_in_dataset = "    Author  Year  Type  -DGJ  +DGJ  Link    Benjamin et al.  2016  HEK-293  6.9 ± 0.4  29.5 ± 1.0   go    ";}
else if (sostituzione == "M42T") {presence_in_dataset = "    Author  Year  Type  -DGJ  +DGJ  Link    Benjamin et al.  2016  HEK-293  2.5 ± 0.1  20.3 ± 1.2   go     Lukas et al.  2016  HEK293H  2.9 ± 0.3  21.4 ± 5   go    ";}
else if (sostituzione == "M42V") {presence_in_dataset = "    Author  Year  Type  -DGJ  +DGJ  Link    Benjamin et al.  2016  HEK-293  0.5 ± 0.1  4.3 ± 0.1   go     Lukas et al.  2013  HEK293H  0  11.9 ± 2.9   go     Park et al.  2009  COS-7  7.2 ± 0.22  17.2 ± 1.31   go     Shimotori et al.  2007  COS-7  4.2  22.5   go    ";}
else if (sostituzione == "M51I") {presence_in_dataset = "    Author  Year  Type  -DGJ  +DGJ  Link    Benjamin et al.  2016  HEK-293  22.3 ± 1.1  47.1 ± 1.5   go     Lukas et al.  2013  HEK293H  37.4 ± 5.9  62 ± 13.2   go     Spada et al.  2006  COS-7  36  81   go    ";}
else if (sostituzione == "M51K") {presence_in_dataset = "    Author  Year  Type  -DGJ  +DGJ  Link    Benjamin et al.  2016  HEK-293  6.3 ± 0.5  22.1 ± 1.9   go     Lukas et al.  2013  HEK293H  0  8.7 ± 4.4   go     Wu et al.  2011  HEK293-Sample-1  5.6 ± 0.5  56.1 ± 8.6   go     Wu et al.  2011  HEK293-Sample-2  5.6  25.1   go     Benjamin et al.  2009  Lymphoblasts  11 ± 2  97   go     Shin et al.  2008  T-Cell-Sample-1  6 ± 1  29 ± 2   go    ";}
else if (sostituzione == "M72I") {presence_in_dataset = "    Author  Year  Type  -DGJ  +DGJ  Link    Benjamin et al.  2016  HEK-293  54.7 ± 1.8  77.6 ± 2.6   go    ";}
else if (sostituzione == "M72V") {presence_in_dataset = "    Author  Year  Type  -DGJ  +DGJ  Link    Benjamin et al.  2016  HEK-293  14.5 ± 0.9  73.2 ± 1.3   go    ";}
else if (sostituzione == "M76T") {presence_in_dataset = "    Author  Year  Type  -DGJ  +DGJ  Link    Benjamin et al.  2016  HEK-293  2.7 ± 0.1  12.2 ± 0.7   go     Shimotori et al.  2007  COS-7  2.9  5.9   go    ";}
else if (sostituzione == "M96I") {presence_in_dataset = "    Author  Year  Type  -DGJ  +DGJ  Link    Benjamin et al.  2016  HEK-293  30.3 ± 1.2  58.2 ± 2.5   go    ";}
else if (sostituzione == "N139S") {presence_in_dataset = "    Author  Year  Type  -DGJ  +DGJ  Link    Benjamin et al.  2016  HEK-293  65.5 ± 2.0  79.1 ± 3.2   go     Lukas et al.  2013  HEK293H  147.8 ± 28.3  176.4 ± 36.4   go    ";}
else if (sostituzione == "N139T") {presence_in_dataset = "    Author  Year  Type  -DGJ  +DGJ  Link    Benjamin et al.  2016  HEK-293  71.4 ± 2.2  79.4 ± 2.0   go    ";}
else if (sostituzione == "N215D") {presence_in_dataset = "    Author  Year  Type  -DGJ  +DGJ  Link    Benjamin et al.  2016  HEK-293  43.8 ± 3.0  58.3 ± 3.1   go    ";}
else if (sostituzione == "N215S") {presence_in_dataset = "    Author  Year  Type  -DGJ  +DGJ  Link    Benjamin et al.  2016  HEK-293  15.6 ± 1.0  35.6 ± 1.2   go     Lukas et al.  2013  HEK293H  39.5 ± 1.5  63.9 ± 3.3   go     Wu et al.  2011  HEK293-Sample-1  15.7 ± 2.4  53.2 ± 2.4   go     Wu et al.  2011  HEK293-Sample-2  15.7  40.7   go     Benjamin et al.  2009  Lymphoblasts  3 ± 0.2  100   go     Benjamin et al.  2009  Fibroblasts  19 ± 7  48.5   go     Shin et al.  2008  T-Cell-Sample-1  15 ± 4  79 ± 10   go     Ishii et al.  2007  Fibroblasts  3.7  21.2   go     Spada et al.  2006  COS-7  47  84.13   go    ";}
else if (sostituzione == "N224D") {presence_in_dataset = "    Author  Year  Type  -DGJ  +DGJ  Link    Benjamin et al.  2016  HEK-293  NR  2.7 ± 0.1   go    ";}
else if (sostituzione == "N224S") {presence_in_dataset = "    Author  Year  Type  -DGJ  +DGJ  Link    Benjamin et al.  2016  HEK-293  10.3 ± 1.0  29.7 ± 2.8   go     Lukas et al.  2013  HEK293H  31.1 ± 2.4  82.2 ± 2.2   go    ";}
else if (sostituzione == "N228S") {presence_in_dataset = "    Author  Year  Type  -DGJ  +DGJ  Link    Benjamin et al.  2016  HEK-293  124.5 ± 4.1  169.2 ± 8.2   go     Lukas et al.  2016  HEK293H  59.5 ± 9.8  70.6 ± 13.1   go    ";}
else if (sostituzione == "N249K") {presence_in_dataset = "    Author  Year  Type  -DGJ  +DGJ  Link    Benjamin et al.  2016  HEK-293  17.9 ± 1.6  35.2 ± 1.4   go     Lukas et al.  2016  HEK293H  23.7 ± 1.7  54.6 ± 2.4   go    ";}
else if (sostituzione == "N263S") {presence_in_dataset = "    Author  Year  Type  -DGJ  +DGJ  Link    Benjamin et al.  2016  HEK-293  15.8 ± 0.9  80.5 ± 6.2   go     Wu et al.  2011  HEK293-Sample-1  6.5 ± 1.4  92.3 ± 9.1   go     Benjamin et al.  2009  Lymphoblasts  5 ± 2  63   go    ";}
else if (sostituzione == "N272K") {presence_in_dataset = "    Author  Year  Type  -DGJ  +DGJ  Link    Wu et al.  2011  HEK293-Sample-1  0  0   go     Benjamin et al.  2009  Lymphoblasts  0  0   go     Shin et al.  2008  T-Cell-Sample-1  0  0   go    ";}
else if (sostituzione == "N272S") {presence_in_dataset = "    Author  Year  Type  -DGJ  +DGJ  Link    Lukas et al.  2013  HEK293H  0  0   go    ";}
else if (sostituzione == "N298S") {presence_in_dataset = "    Author  Year  Type  -DGJ  +DGJ  Link    Benjamin et al.  2016  HEK-293  5.0 ± 0.2  41.4 ± 1.5   go    ";}
else if (sostituzione == "N320I") {presence_in_dataset = "    Author  Year  Type  -DGJ  +DGJ  Link    Benjamin et al.  2016  HEK-293  1.4 ± 0.1  17.1 ± 0.5   go     Lukas et al.  2013  HEK293H  2 ± 0.5  31.8 ± 8.9   go    ";}
else if (sostituzione == "N320Y") {presence_in_dataset = "    Author  Year  Type  -DGJ  +DGJ  Link    Benjamin et al.  2016  HEK-293  NR  0.6 ± 0.1   go     Lukas et al.  2016  HEK293H  0  0   go     Wu et al.  2011  HEK293-Sample-1  0  29.6 ± 3.5   go     Benjamin et al.  2009  Lymphoblasts  2 ± 0.7  9   go    ";}
else if (sostituzione == "N34K") {presence_in_dataset = "    Author  Year  Type  -DGJ  +DGJ  Link    Benjamin et al.  2016  HEK-293  NR  6.6 ± 0.6   go    ";}
else if (sostituzione == "N34S") {presence_in_dataset = "    Author  Year  Type  -DGJ  +DGJ  Link    Benjamin et al.  2016  HEK-293  0.6 ± 0.1  16.7 ± 0.6   go     Wu et al.  2011  HEK293-Sample-1  0  0   go     Benjamin et al.  2009  Lymphoblasts  8 ± 3  60   go    ";}
else if (sostituzione == "N53D") {presence_in_dataset = "    Author  Year  Type  -DGJ  +DGJ  Link    Benjamin et al.  2016  HEK-293  42.2 ± 1.9  79.4 ± 2.0   go    ";}
else if (sostituzione == "N53L") {presence_in_dataset = "    Author  Year  Type  -DGJ  +DGJ  Link    Benjamin et al.  2016  HEK-293  18.5 ± 0.9  48.6 ± 1.5   go    ";}
else if (sostituzione == "P146S") {presence_in_dataset = "    Author  Year  Type  -DGJ  +DGJ  Link    Benjamin et al.  2016  HEK-293  41.9 ± 1.9  64.1 ± 3.7   go    ";}
else if (sostituzione == "P205L") {presence_in_dataset = "    Author  Year  Type  -DGJ  +DGJ  Link    Benjamin et al.  2016  HEK-293  0.8 ± 0.1  4.7 ± 0.7   go    ";}
else if (sostituzione == "P205R") {presence_in_dataset = "    Author  Year  Type  -DGJ  +DGJ  Link    Wu et al.  2011  HEK293-Sample-1  0  0   go     Benjamin et al.  2009  Lymphoblasts  1 ± 0.4  3   go    ";}
else if (sostituzione == "P205S") {presence_in_dataset = "    Author  Year  Type  -DGJ  +DGJ  Link    Benjamin et al.  2016  HEK-293  35.3 ± 2.2  93.2 ± 5.5   go    ";}
else if (sostituzione == "P205T") {presence_in_dataset = "    Author  Year  Type  -DGJ  +DGJ  Link    Benjamin et al.  2016  HEK-293  14.4 ± 0.9  48.8 ± 2.2   go     Giugliani et al.  2013  HEK-293  18.2 ± 2.8  62.6 4.1   go     Wu et al.  2011  HEK293-Sample-1  18.2 ± 2.8  132 ± 21   go     Wu et al.  2011  HEK293-Sample-2  18.2  2.5   go     Benjamin et al.  2009  Lymphoblasts  6 ± 1  74   go     Shin et al.  2008  T-Cell-Sample-1  37  108   go     Shimotori et al.  2007  COS-7  8.5  150   go    ";}
else if (sostituzione == "P210L") {presence_in_dataset = "    Author  Year  Type  -DGJ  +DGJ  Link    Benjamin et al.  2016  HEK-293  27.1 ± 1  60.5 ± 1.7   go    ";}
else if (sostituzione == "P210S") {presence_in_dataset = "    Author  Year  Type  -DGJ  +DGJ  Link    Benjamin et al.  2016  HEK-293  75.2 ± 2  113.3 ± 2.7   go    ";}
else if (sostituzione == "P214L") {presence_in_dataset = "    Author  Year  Type  -DGJ  +DGJ  Link    Benjamin et al.  2016  HEK-293  33 ± 1.3  91.6 ± 1.6   go     Lukas et al.  2016  HEK293H  19.4 ± 1.4  64.1 ± 9.8   go    ";}
else if (sostituzione == "P214S") {presence_in_dataset = "    Author  Year  Type  -DGJ  +DGJ  Link    Benjamin et al.  2016  HEK-293  22.4 ± 1.1  82.5 ± 3.8   go    ";}
else if (sostituzione == "P259L") {presence_in_dataset = "    Author  Year  Type  -DGJ  +DGJ  Link    Benjamin et al.  2016  HEK-293  10.9 ± 0.6  44.0 ± 1.8   go    ";}
else if (sostituzione == "P259R") {presence_in_dataset = "    Author  Year  Type  -DGJ  +DGJ  Link    Benjamin et al.  2016  HEK-293  23.3 ± 2.3  60.3 ± 3.8   go     Lukas et al.  2016  HEK293H  20.5 ± 2.6  40 ± 4.5   go     Giugliani et al.  2013  HEK-293  28.1 ± 4.6  67.8 ± 9.5   go     Wu et al.  2011  HEK293-Sample-1  28.1 ± 4.6  132 ± 10   go     Wu et al.  2011  HEK293-Sample-2  28.1  67   go     Shin et al.  2008  T-Cell-Sample-1  4 ± 2  154 ± 77   go     Shin et al.  2008  T-Cell-Sample-2  4 ± 2  138 ± 16   go    ";}
else if (sostituzione == "P265L") {presence_in_dataset = "    Author  Year  Type  -DGJ  +DGJ  Link    Benjamin et al.  2016  HEK-293  13.9 ± 0.7  74.8 ± 2.9   go    ";}
else if (sostituzione == "P265R") {presence_in_dataset = "    Author  Year  Type  -DGJ  +DGJ  Link    Benjamin et al.  2016  HEK-293  NR  1.8 ± 0.1   go    ";}
else if (sostituzione == "P265S") {presence_in_dataset = "    Author  Year  Type  -DGJ  +DGJ  Link    Benjamin et al.  2016  HEK-293  1.0 ± 0.1  3.9 ± 0.2   go    ";}
else if (sostituzione == "P293A") {presence_in_dataset = "    Author  Year  Type  -DGJ  +DGJ  Link    Benjamin et al.  2016  HEK-293  NR  0.7 ± 0.1   go    ";}
else if (sostituzione == "P293S") {presence_in_dataset = "    Author  Year  Type  -DGJ  +DGJ  Link    Benjamin et al.  2016  HEK-293  NR  0.5 ± 0.0   go    ";}
else if (sostituzione == "P293T") {presence_in_dataset = "    Author  Year  Type  -DGJ  +DGJ  Link    Benjamin et al.  2016  HEK-293  0.7 ± 0.1  13.3 ± 1.1   go    ";}
else if (sostituzione == "P323R") {presence_in_dataset = "    Author  Year  Type  -DGJ  +DGJ  Link    Lukas et al.  2016  HEK293H  62.7 ± 4.4  63.7 ± 3.1   go    ";}
else if (sostituzione == "P343L") {presence_in_dataset = "    Author  Year  Type  -DGJ  +DGJ  Link    Benjamin et al.  2016  HEK-293  36.6 ± 0.9  49.6 ± 1.5   go    ";}
else if (sostituzione == "P362L") {presence_in_dataset = "    Author  Year  Type  -DGJ  +DGJ  Link    Benjamin et al.  2016  HEK-293  2.8 ± 0.1  19.6 ± 0.8   go    ";}
else if (sostituzione == "P362T") {presence_in_dataset = "    Author  Year  Type  -DGJ  +DGJ  Link    Benjamin et al.  2016  HEK-293  32 ± 1.7  70.5 ± 4.5   go    ";}
else if (sostituzione == "P389A") {presence_in_dataset = "    Author  Year  Type  -DGJ  +DGJ  Link    Benjamin et al.  2016  HEK-293  2.7 ± 0.1  4.0 ± 0.1   go    ";}
else if (sostituzione == "P389L") {presence_in_dataset = "    Author  Year  Type  -DGJ  +DGJ  Link    Benjamin et al.  2016  HEK-293  NR  0.6 ± 0   go     Lukas et al.  2013  HEK293H  0  0   go    ";}
else if (sostituzione == "P409A") {presence_in_dataset = "    Author  Year  Type  -DGJ  +DGJ  Link    Benjamin et al.  2016  HEK-293  3.1 ± 0.2  28.9 ± 1.3   go     Wu et al.  2011  HEK293-Sample-1  2.8 ± 0.5  42 ± 7.8   go     Benjamin et al.  2009  Lymphoblasts  3 ± 1  26   go    ";}
else if (sostituzione == "P409S") {presence_in_dataset = "    Author  Year  Type  -DGJ  +DGJ  Link    Benjamin et al.  2016  HEK-293  2.4 ± 0.1  23.0 ± 0.8   go    ";}
else if (sostituzione == "P409T") {presence_in_dataset = "    Author  Year  Type  -DGJ  +DGJ  Link    Benjamin et al.  2016  HEK-293  2.7 ± 0.2  24.7 ± 1.6   go    ";}
else if (sostituzione == "P40A") {presence_in_dataset = "    Author  Year  Type  -DGJ  +DGJ  Link    Benjamin et al.  2016  HEK-293  NR  2.1 ± 0.1   go    ";}
else if (sostituzione == "P40S") {presence_in_dataset = "    Author  Year  Type  -DGJ  +DGJ  Link    Benjamin et al.  2016  HEK-293  NR  1.0 ± 0.1   go     Lukas et al.  2016  HEK293H  0  1.4 ± 0.5   go     Wu et al.  2011  HEK293-Sample-1  0  16.7 ± 2.7   go     Benjamin et al.  2009  Lymphoblasts  2 ± 0.5  17   go    ";}
else if (sostituzione == "P60L") {presence_in_dataset = "    Author  Year  Type  -DGJ  +DGJ  Link    Benjamin et al.  2016  HEK-293  21.7 ± 1.1  61.0 ± 2.4   go     Lukas et al.  2016  HEK293H  15.6 ± 0.2  33.1 ± 1   go    ";}
else if (sostituzione == "P60S") {presence_in_dataset = "    Author  Year  Type  -DGJ  +DGJ  Link    Benjamin et al.  2016  HEK-293  35.1 ± 1.7  78.1 ± 2   go    ";}
else if (sostituzione == "P60T") {presence_in_dataset = "    Author  Year  Type  -DGJ  +DGJ  Link    Benjamin et al.  2016  HEK-293  25.3 ± 1.3  77.7 ± 1.9   go    ";}
else if (sostituzione == "Q107L") {presence_in_dataset = "    Author  Year  Type  -DGJ  +DGJ  Link    Benjamin et al.  2016  HEK-293  98.4 ± 4.3  110.0 ± 2.7   go    ";}
else if (sostituzione == "Q250P") {presence_in_dataset = "    Author  Year  Type  -DGJ  +DGJ  Link    Benjamin et al.  2016  HEK-293  24.8 ± 1.3  58.7 ± 2.9   go    ";}
else if (sostituzione == "Q279E") {presence_in_dataset = "    Author  Year  Type  -DGJ  +DGJ  Link    Benjamin et al.  2016  HEK-293  16.7 ± 1.0  51.7 ± 2.2   go     Wu et al.  2011  HEK293-Sample-1  16.6 ± 2.5  104 ± 25   go     Benjamin et al.  2009  Lymphoblasts  7 ± 3  63   go     Ishii et al.  2007  Lymphoblasts  3.6  45   go    ";}
else if (sostituzione == "Q279H") {presence_in_dataset = "    Author  Year  Type  -DGJ  +DGJ  Link    Benjamin et al.  2016  HEK-293  NR  0.5 ± 0.1   go    ";}
else if (sostituzione == "Q280H") {presence_in_dataset = "    Author  Year  Type  -DGJ  +DGJ  Link    Benjamin et al.  2016  HEK-293  7.3 ± 0.4  39.2 ± 2.1   go    ";}
else if (sostituzione == "Q280K") {presence_in_dataset = "    Author  Year  Type  -DGJ  +DGJ  Link    Benjamin et al.  2016  HEK-293  27.7 ± 1.4  48.5 ± 1.9   go     Andreotti et al.  2011  COS-7  19.8 ± 3.7  79.9 ± 31.3   go    ";}
else if (sostituzione == "Q283P") {presence_in_dataset = "    Author  Year  Type  -DGJ  +DGJ  Link    Lukas et al.  2013  HEK293H  0  0   go    ";}
else if (sostituzione == "Q312H") {presence_in_dataset = "    Author  Year  Type  -DGJ  +DGJ  Link    Benjamin et al.  2016  HEK-293  6.1 ± 0.7  23.1 ± 1.7   go    ";}
else if (sostituzione == "Q312R") {presence_in_dataset = "    Author  Year  Type  -DGJ  +DGJ  Link    Benjamin et al.  2016  HEK-293  18.5 ± 1.3  31.9 ± 1.4   go     Shimotori et al.  2007  COS-7  10.1  43.5   go    ";}
else if (sostituzione == "Q321E") {presence_in_dataset = "    Author  Year  Type  -DGJ  +DGJ  Link    Benjamin et al.  2016  HEK-293  NR  2.9 ± 0.1   go    ";}
else if (sostituzione == "Q321H") {presence_in_dataset = "    Author  Year  Type  -DGJ  +DGJ  Link    Benjamin et al.  2016  HEK-293  1.9 ± 0.1  19.8 ± 0.9   go     Lukas et al.  2013  HEK293H  3.3 ± 1.9  25.3 ± 7.1   go    ";}
else if (sostituzione == "Q321L") {presence_in_dataset = "    Author  Year  Type  -DGJ  +DGJ  Link    Benjamin et al.  2016  HEK-293  2.9 ± 0.2  24.6 ± 1.4   go    ";}
else if (sostituzione == "Q321R") {presence_in_dataset = "    Author  Year  Type  -DGJ  +DGJ  Link    Benjamin et al.  2016  HEK-293  22.3 ± 1.3  66.1 ± 1.9   go    ";}
else if (sostituzione == "Q327E") {presence_in_dataset = "    Author  Year  Type  -DGJ  +DGJ  Link    Benjamin et al.  2016  HEK-293  22.9 ± 1.1  50.4 ± 1.6   go     Lukas et al.  2013  HEK293H  21.5 ± 2.8  80.9 ± 10   go    ";}
else if (sostituzione == "Q327K") {presence_in_dataset = "    Author  Year  Type  -DGJ  +DGJ  Link    Lukas et al.  2013  HEK293H  0  0   go    ";}
else if (sostituzione == "Q327L") {presence_in_dataset = "    Author  Year  Type  -DGJ  +DGJ  Link    Lukas et al.  2016  HEK293H  0.6 ± 0.1  20 ± 3   go    ";}
else if (sostituzione == "Q327R") {presence_in_dataset = "    Author  Year  Type  -DGJ  +DGJ  Link    Lukas et al.  2016  HEK293H  0  3.9 ± 0.1   go    ";}
else if (sostituzione == "Q330R") {presence_in_dataset = "    Author  Year  Type  -DGJ  +DGJ  Link    Lukas et al.  2016  HEK293H  50.9 ± 5.4  62.8 ± 11.9   go    ";}
else if (sostituzione == "Q333R ") {presence_in_dataset = "    Author  Year  Type  -DGJ  +DGJ  Link    Benjamin et al.  2016  HEK-293  93.5 ± 2.5  113.1 ± 3.2   go    ";}
else if (sostituzione == "Q386P") {presence_in_dataset = "    Author  Year  Type  -DGJ  +DGJ  Link    Lukas et al.  2013  HEK293H  0  0   go    ";}
else if (sostituzione == "Q416P") {presence_in_dataset = "    Author  Year  Type  -DGJ  +DGJ  Link    Lukas et al.  2013  HEK293H  0  0   go    ";}
else if (sostituzione == "Q57L") {presence_in_dataset = "    Author  Year  Type  -DGJ  +DGJ  Link    Benjamin et al.  2016  HEK-293  71.6 ± 2.0  93.3 ± 2.7   go    ";}
else if (sostituzione == "R100K") {presence_in_dataset = "    Author  Year  Type  -DGJ  +DGJ  Link    Wu et al.  2011  HEK293-Sample-1  0  0   go     Benjamin et al.  2009  Lymphoblasts  0.5 ± 0.1  2   go    ";}
else if (sostituzione == "R100T") {presence_in_dataset = "    Author  Year  Type  -DGJ  +DGJ  Link    Lukas et al.  2013  HEK293H  0  0   go     Wu et al.  2011  HEK293-Sample-1  0  0   go     Benjamin et al.  2009  Lymphoblasts  0  0   go    ";}
else if (sostituzione == "R112C") {presence_in_dataset = "    Author  Year  Type  -DGJ  +DGJ  Link    Lukas et al.  2013  HEK293H  0  0   go     Wu et al.  2011  HEK293-Sample-1  0  3.1 ± 0.6   go     Wu et al.  2011  HEK293-Sample-2  0  0   go     Benjamin et al.  2009  Lymphoblasts  2 ± 0.9  17   go     Park et al.  2009  COS-7  4.7 ± 0.13  9.3 ± 0.6   go     Shin et al.  2008  T-Cell-Sample-1  6 ± 5  23 ± 18   go     Shin et al.  2008  T-Cell-Sample-2  8 ± 4  49 ± 10   go     Shin et al.  2007  T-Cell-Sample-1  7.8 ± 3.9  49 ± 9.7   go    ";}
else if (sostituzione == "R112G") {presence_in_dataset = "    Author  Year  Type  -DGJ  +DGJ  Link    Benjamin et al.  2016  HEK-293  NR  3.5 ± 0.2   go    ";}
else if (sostituzione == "R112H") {presence_in_dataset = "    Author  Year  Type  -DGJ  +DGJ  Link    Benjamin et al.  2016  HEK-293  2.6 ± 0.1  17.4 ± 0.8   go     Lukas et al.  2013  HEK293H  1.6 ± 0.6  19.4 ± 2.3   go     Giugliani et al.  2013  HEK-293  1.5 ± 0.5  8.2 ± 1.9   go     Wu et al.  2011  HEK293-Sample-1  0  19 ± 1.3   go     Benjamin et al.  2009  Lymphoblasts  19 ± 4  54   go     Shin et al.  2008  T-Cell-Sample-1  3 ± 1  51 ± 21   go     Shin et al.  2008  T-Cell-Sample-2  8 ± 3  73 ± 2   go     Shin et al.  2008  T-Cell-Sample-3  3 ± 2  60 ± 17   go     Shimotori et al.  2007  COS-7  0.6  65.5   go     Shin et al.  2007  T-Cell-Sample-1  3 ± 1.5  59.9 ± 17.2   go     Ishii et al.  2007  Fibroblasts  5.2  31.9   go    ";}
else if (sostituzione == "R112S") {presence_in_dataset = "    Author  Year  Type  -DGJ  +DGJ  Link    Benjamin et al.  2016  HEK-293  NR  2.8 ± 0.2   go    ";}
else if (sostituzione == "R118C") {presence_in_dataset = "    Author  Year  Type  -DGJ  +DGJ  Link    Benjamin et al.  2016  HEK-293  24.0 ± 1.3  29.5 ± 0.7   go     Lukas et al.  2013  HEK293H  20 ± 1.3  23.7 ± 2.9   go     Spada et al.  2006  COS-7  29  31.9   go    ";}
else if (sostituzione == "R118G") {presence_in_dataset = "    Author  Year  Type  -DGJ  +DGJ  Link    Lukas et al.  2013  HEK293H  37.6 ± 4.1  44 ± 5   go    ";}
else if (sostituzione == "R118H") {presence_in_dataset = "    Author  Year  Type  -DGJ  +DGJ  Link    Lukas et al.  2013  HEK293H  67.4 ± 3.4  69.9 ± 3.1   go    ";}
else if (sostituzione == "R118L") {presence_in_dataset = "    Author  Year  Type  -DGJ  +DGJ  Link    Lukas et al.  2013  HEK293H  51.7 ± 3.8  51.4 ± 4   go    ";}
else if (sostituzione == "R118P") {presence_in_dataset = "    Author  Year  Type  -DGJ  +DGJ  Link    Lukas et al.  2013  HEK293H  49 ± 4.9  47.7 ± 2   go    ";}
else if (sostituzione == "R118S") {presence_in_dataset = "    Author  Year  Type  -DGJ  +DGJ  Link    Lukas et al.  2013  HEK293H  76.2 ± 9.5  70.9 ± 2.6   go    ";}
else if (sostituzione == "R196S") {presence_in_dataset = "    Author  Year  Type  -DGJ  +DGJ  Link    Lukas et al.  2016  HEK293H  42.1 ± 2.5  67.8 ± 6.6   go    ";}
else if (sostituzione == "R220P") {presence_in_dataset = "    Author  Year  Type  -DGJ  +DGJ  Link    Benjamin et al.  2016  HEK-293  30.9 ± 1.8  63.5 ± 2.2   go    ";}
else if (sostituzione == "R220Q") {presence_in_dataset = "    Author  Year  Type  -DGJ  +DGJ  Link    Benjamin et al.  2016  HEK-293  45.2 ± 1.4  61.7 ± 0.8   go     Lukas et al.  2013  HEK293H  104.5 ± 11.3  144 ± 11.9   go    ";}
else if (sostituzione == "R227P") {presence_in_dataset = "    Author  Year  Type  -DGJ  +DGJ  Link    Lukas et al.  2016  HEK293H  0  0   go    ";}
else if (sostituzione == "R227Q") {presence_in_dataset = "    Author  Year  Type  -DGJ  +DGJ  Link    Lukas et al.  2013  HEK293H  0  0   go     Wu et al.  2011  HEK293-Sample-1  0  0   go     Wu et al.  2011  HEK293-Sample-2  0  0   go     Benjamin et al.  2009  Lymphoblasts  0  0   go     Shin et al.  2008  T-Cell-Sample-1  4 ± 5  3 ± 4   go    ";}
else if (sostituzione == "R252T") {presence_in_dataset = "    Author  Year  Type  -DGJ  +DGJ  Link    Benjamin et al.  2016  HEK-293  74.8 ± 2.5  79.1 ± 2.1   go     Lukas et al.  2013  HEK293H  117 ± 10  134.3 ± 22.5   go    ";}
else if (sostituzione == "R301G") {presence_in_dataset = "    Author  Year  Type  -DGJ  +DGJ  Link    Benjamin et al.  2016  HEK-293  19.1 ± 1.2  64.7 ± 2.1   go     Lukas et al.  2013  HEK293H  19.3 ± 4.1  56.5 ± 3.4   go    ";}
else if (sostituzione == "R301L") {presence_in_dataset = "    Author  Year  Type  -DGJ  +DGJ  Link    Benjamin et al.  2016  HEK-293  4.3 ± 0.2  36.1 ± 0.7   go    ";}
else if (sostituzione == "R301P") {presence_in_dataset = "    Author  Year  Type  -DGJ  +DGJ  Link    Benjamin et al.  2016  HEK-293  NR  4.2 ± 0.5   go     Lukas et al.  2013  HEK293H  0  5 ± 1.3   go     Andreotti et al.  2011  COS-7  4.2 ± 0.7  15.7 ± 1.9   go    ";}
else if (sostituzione == "R301Q") {presence_in_dataset = "    Author  Year  Type  -DGJ  +DGJ  Link    Benjamin et al.  2016  HEK-293  5.5 ± 0.2  44.5 ± 1.0   go     Lukas et al.  2013  HEK293H  8.5 ± 1.8  48 ± 2.6   go     Wu et al.  2011  HEK293-Sample-1  5.6 ± 0.3  83.7 ± 1.9   go     Wu et al.  2011  HEK293-Sample-2  5.6  49.5   go     Benjamin et al.  2009  Lymphoblasts  5 ± 0.5  54   go     Benjamin et al.  2009  Fibroblasts  20 ± 8  80   go     Shin et al.  2008  T-Cell-Sample-1  11 ± 6  87 ± 11   go     Shin et al.  2008  T-Cell-Sample-2  10 ± 3  94 ± 42   go     Shin et al.  2008  T-Cell-Sample-3  7 ± 2  80 ± 27   go     Shin et al.  2007  T-Cell-Sample-1  6.6 ± 1.8  79.8 ± 27   go     Ishii et al.  2007  Fibroblasts  6.3  26.3   go     Ishii et al.  2007  Lymphoblasts  4  48   go    ";}
else if (sostituzione == "R342L") {presence_in_dataset = "    Author  Year  Type  -DGJ  +DGJ  Link    Lukas et al.  2013  HEK293H  0  0   go    ";}
else if (sostituzione == "R342P") {presence_in_dataset = "    Author  Year  Type  -DGJ  +DGJ  Link    Lukas et al.  2016  HEK293H  0  0   go    ";}
else if (sostituzione == "R342Q") {presence_in_dataset = "    Author  Year  Type  -DGJ  +DGJ  Link    Benjamin et al.  2016  HEK-293  NR  0.9 ± 0.1   go     Lukas et al.  2013  HEK293H  0  0   go     Wu et al.  2011  HEK293-Sample-1  0  4.4 ± 0.4   go     Benjamin et al.  2009  Lymphoblasts  3 ± 0.4  6   go    ";}
else if (sostituzione == "R356G") {presence_in_dataset = "    Author  Year  Type  -DGJ  +DGJ  Link    Benjamin et al.  2016  HEK-293  15.4 ± 0.7  36.2 ± 1.2   go    ";}
else if (sostituzione == "R356P") {presence_in_dataset = "    Author  Year  Type  -DGJ  +DGJ  Link    Lukas et al.  2016  HEK293H  2.1 ± 0.6  6.7 ± 2.3   go    ";}
else if (sostituzione == "R356Q") {presence_in_dataset = "    Author  Year  Type  -DGJ  +DGJ  Link    Benjamin et al.  2016  HEK-293  36.1 ± 1.5  75.1 ± 3.6   go     Lukas et al.  2013  HEK293H  89.1 ± 5  99.4 ± 4.3   go    ";}
else if (sostituzione == "R356W") {presence_in_dataset = "    Author  Year  Type  -DGJ  +DGJ  Link    Benjamin et al.  2016  HEK-293  11.0 ± 0.7  49.1 ± 2.6   go     Lukas et al.  2013  HEK293H  16.9 ± 2.3  62.7 ± 2.9   go     Wu et al.  2011  HEK293-Sample-1  7.6 ± 1.4  75.6 ± 11.6   go     Benjamin et al.  2009  Lymphoblasts  4 ± 1  43   go     Shin et al.  2008  T-Cell-Sample-1  1  1   go     Shin et al.  2008  T-Cell-Sample-2  0 ± 0  0 ± 1   go     Shin et al.  2007  T-Cell-Sample-1  0.01 ± 0.02  0.43 ± 0.85   go    ";}
else if (sostituzione == "R363C") {presence_in_dataset = "    Author  Year  Type  -DGJ  +DGJ  Link    Benjamin et al.  2016  HEK-293  11.9 ± 0.6  36.0 ± 1.6   go     Wu et al.  2011  HEK293-Sample-1  7.5 ± 0.7  42 ± 3.9   go     Benjamin et al.  2009  Lymphoblasts  8 ± 2  57   go    ";}
else if (sostituzione == "R363H") {presence_in_dataset = "    Author  Year  Type  -DGJ  +DGJ  Link    Benjamin et al.  2016  HEK-293  20.0 ± 0.8  50.5 ± 1.6   go     Lukas et al.  2013  HEK293H  31.9 ± 2.9  57.9 ± 5.5   go     Wu et al.  2011  HEK293-Sample-1  24.1 ± 6.8  73.4 ± 5.3   go     Benjamin et al.  2009  Lymphoblasts  12 ± 2  106   go    ";}
else if (sostituzione == "R363P") {presence_in_dataset = "    Author  Year  Type  -DGJ  +DGJ  Link    Benjamin et al.  2016  HEK-293  NR  0.8 ± 0.0   go    ";}
else if (sostituzione == "R392S") {presence_in_dataset = "    Author  Year  Type  -DGJ  +DGJ  Link    Lukas et al.  2016  HEK293H  44.3 ± 1.6  46.2 ± 1.7   go    ";}
else if (sostituzione == "R49C") {presence_in_dataset = "    Author  Year  Type  -DGJ  +DGJ  Link    Benjamin et al.  2016  HEK-293  NR  2.7 ± 0.2   go     Lukas et al.  2013  HEK293H  0  5.1 ± 0.5   go     Shin et al.  2008  T-Cell-Sample-1  3 ± 4  11 ± 8   go    ";}
else if (sostituzione == "R49G") {presence_in_dataset = "    Author  Year  Type  -DGJ  +DGJ  Link    Lukas et al.  2013  HEK293H  0  0   go    ";}
else if (sostituzione == "R49L") {presence_in_dataset = "    Author  Year  Type  -DGJ  +DGJ  Link    Benjamin et al.  2016  HEK-293  NR  0.9 ± 0.1   go     Wu et al.  2011  HEK293-Sample-1  0  1.2 ± 0.3   go     Benjamin et al.  2009  Lymphoblasts  1 ± 0.3  9   go    ";}
else if (sostituzione == "S102L") {presence_in_dataset = "    Author  Year  Type  -DGJ  +DGJ  Link    Benjamin et al.  2016  HEK-293  19.9 ± 1  62.8 ± 2.2   go     Lukas et al.  2013  HEK293H  71.6 ± 4.4  78.9 ± 8.3   go    ";}
else if (sostituzione == "S126C") {presence_in_dataset = "    Author  Year  Type  -DGJ  +DGJ  Link    Lukas et al.  2013  HEK293H  1.1 ± 0.4  2.7 ± 0.2   go    ";}
else if (sostituzione == "S126G") {presence_in_dataset = "    Author  Year  Type  -DGJ  +DGJ  Link    Benjamin et al.  2016  HEK-293  83.7 ± 2.4  113.9 ± 3.2   go     Lukas et al.  2013  HEK293H  51.3 ± 9.6  67.4 ± 11.9   go    ";}
else if (sostituzione == "S126I") {presence_in_dataset = "    Author  Year  Type  -DGJ  +DGJ  Link    Lukas et al.  2013  HEK293H  40.5 ± 8.1  43.2 ± 7.7   go    ";}
else if (sostituzione == "S126N") {presence_in_dataset = "    Author  Year  Type  -DGJ  +DGJ  Link    Lukas et al.  2013  HEK293H  14.2 ± 2.4  17 ± 2.3   go    ";}
else if (sostituzione == "S126R") {presence_in_dataset = "    Author  Year  Type  -DGJ  +DGJ  Link    Lukas et al.  2013  HEK293H  19.7 ± 1.2  23.5 ± 2.3   go    ";}
else if (sostituzione == "S126T") {presence_in_dataset = "    Author  Year  Type  -DGJ  +DGJ  Link    Lukas et al.  2013  HEK293H  48.9 ± 11.4  42.8 ± 7.9   go    ";}
else if (sostituzione == "S148N") {presence_in_dataset = "    Author  Year  Type  -DGJ  +DGJ  Link    Benjamin et al.  2016  HEK-293  NR  0.7 ± 0.0   go     Wu et al.  2011  HEK293-Sample-1  0  11.9 ± 1.3   go     Benjamin et al.  2009  Lymphoblasts  2 ± 0.6  6   go     Shimotori et al.  2007  COS-7  0.1  1.3   go    ";}
else if (sostituzione == "S148R") {presence_in_dataset = "    Author  Year  Type  -DGJ  +DGJ  Link    Wu et al.  2011  HEK293-Sample-1  0  0   go     Benjamin et al.  2009  Lymphoblasts  0  0   go    ";}
else if (sostituzione == "S201F") {presence_in_dataset = "    Author  Year  Type  -DGJ  +DGJ  Link    Benjamin et al.  2016  HEK-293  2.4 ± 0.1  30.8 ± 1.8   go     Shin et al.  2008  T-Cell-Sample-1  9  82   go    ";}
else if (sostituzione == "S201Y") {presence_in_dataset = "    Author  Year  Type  -DGJ  +DGJ  Link    Benjamin et al.  2016  HEK-293  6.5 ± 0.3  40.2 ± 1.7   go    ";}
else if (sostituzione == "S235C") {presence_in_dataset = "    Author  Year  Type  -DGJ  +DGJ  Link    Benjamin et al.  2016  HEK-293  NR  0.7 ± 0.1   go     Wu et al.  2011  HEK293-Sample-1  0  3.1 ± 0.5   go     Benjamin et al.  2009  Lymphoblasts  4 ± 0.1  17   go    ";}
else if (sostituzione == "S235F") {presence_in_dataset = "    Author  Year  Type  -DGJ  +DGJ  Link    Shimotori et al.  2007  COS-7  3.2  8.1   go    ";}
else if (sostituzione == "S238N") {presence_in_dataset = "    Author  Year  Type  -DGJ  +DGJ  Link    Benjamin et al.  2016  HEK-293  37.1 ± 1.8  96.4 ± 2.0   go     Lukas et al.  2013  HEK293H  36 ± 6.7  94.3 ± 24.5   go    ";}
else if (sostituzione == "S238R") {presence_in_dataset = "    Author  Year  Type  -DGJ  +DGJ  Link    Benjamin et al.  2016  HEK-293  55.3 ± 2.7  58.0 ± 2.5   go    ";}
else if (sostituzione == "S247C") {presence_in_dataset = "    Author  Year  Type  -DGJ  +DGJ  Link    Benjamin et al.  2016  HEK-293  28.0 ± 1.1  51.5 ± 2.0   go    ";}
else if (sostituzione == "S247P") {presence_in_dataset = "    Author  Year  Type  -DGJ  +DGJ  Link    Benjamin et al.  2016  HEK-293  NR  1.3 ± 0.1   go     Lukas et al.  2016  HEK293H  0  5.8 ± 1.5   go    ";}
else if (sostituzione == "S276G") {presence_in_dataset = "    Author  Year  Type  -DGJ  +DGJ  Link    Benjamin et al.  2016  HEK-293  NR  2.0 ± 0.1   go     Lukas et al.  2016  HEK293H  0  5.6 ± 1.4   go     Wu et al.  2011  HEK293-Sample-1  0  12.7 ± 2.5   go     Wu et al.  2011  HEK293-Sample-2  0  3.7   go     Benjamin et al.  2009  Lymphoblasts  5 ± 2  23   go     Shin et al.  2008  T-Cell-Sample-1  2 ± 4  14 ± 6   go     Shin et al.  2008  T-Cell-Sample-2  1 ± 1  12 ± 2   go    ";}
else if (sostituzione == "S276N") {presence_in_dataset = "    Author  Year  Type  -DGJ  +DGJ  Link    Benjamin et al.  2016  HEK-293  2.3 ± 0.1  9.3 ± 0.9   go    ";}
else if (sostituzione == "S297C") {presence_in_dataset = "    Author  Year  Type  -DGJ  +DGJ  Link    Lukas et al.  2013  HEK293H  0  3.8 ± 0.7   go    ";}
else if (sostituzione == "S297F") {presence_in_dataset = "    Author  Year  Type  -DGJ  +DGJ  Link    Park et al.  2009  COS-7  5.2 ± 0.41  5.9 ± 0.45   go    ";}
else if (sostituzione == "S304N") {presence_in_dataset = "    Author  Year  Type  -DGJ  +DGJ  Link    Benjamin et al.  2016  HEK-293  94.1 ± 1.1  121.8 ± 2.2   go    ";}
else if (sostituzione == "S304T") {presence_in_dataset = "    Author  Year  Type  -DGJ  +DGJ  Link    Benjamin et al.  2016  HEK-293  76.4 ± 1.6  116.9 ± 3.3   go    ";}
else if (sostituzione == "S345P") {presence_in_dataset = "    Author  Year  Type  -DGJ  +DGJ  Link    Benjamin et al.  2016  HEK-293  NR  3.7 ± 0.2   go     Lukas et al.  2013  HEK293H  0  13.3 ± 3.8   go    ";}
else if (sostituzione == "S405R") {presence_in_dataset = "    Author  Year  Type  -DGJ  +DGJ  Link    Benjamin et al.  2016  HEK-293  52.5 ± 3.7  59.6 ± 5.1   go     Lukas et al.  2013  HEK293H  91 ± 10.2  92.7 ± 11.1   go    ";}
else if (sostituzione == "S65A") {presence_in_dataset = "    Author  Year  Type  -DGJ  +DGJ  Link    Ishii et al.  2000  COS-1  24.44  44   go    ";}
else if (sostituzione == "S65I") {presence_in_dataset = "    Author  Year  Type  -DGJ  +DGJ  Link    Lukas et al.  2013  HEK293H  0  11.1 ± 1.2   go    ";}
else if (sostituzione == "S65T") {presence_in_dataset = "    Author  Year  Type  -DGJ  +DGJ  Link    Ishii et al.  2000  COS-1  8.94  34   go    ";}
else if (sostituzione == "T141I") {presence_in_dataset = "    Author  Year  Type  -DGJ  +DGJ  Link    Lukas et al.  2013  HEK293H  0  0   go    ";}
else if (sostituzione == "T194I") {presence_in_dataset = "    Author  Year  Type  -DGJ  +DGJ  Link    Benjamin et al.  2016  HEK-293  2.3 ± 0.1  19.1 ± 1.2   go    ";}
else if (sostituzione == "T282A") {presence_in_dataset = "    Author  Year  Type  -DGJ  +DGJ  Link    Benjamin et al.  2016  HEK-293  NR  4.2 ± 0.2   go     Shimotori et al.  2007  COS-7  0.2  5.9   go    ";}
else if (sostituzione == "T282I") {presence_in_dataset = "    Author  Year  Type  -DGJ  +DGJ  Link    Benjamin et al.  2016  HEK-293  5.2 ± 0.2  23.7 ± 0.6   go     Lukas et al.  2013  HEK293H  5 ± 0.5  47.7 ± 0.7   go    ";}
else if (sostituzione == "T385A") {presence_in_dataset = "    Author  Year  Type  -DGJ  +DGJ  Link    Benjamin et al.  2016  HEK-293  57.3 ± 2.1  73.5 ± 2.4   go     Lukas et al.  2013  HEK293H  45 ± 3.7  48.9 ± 7.5   go    ";}
else if (sostituzione == "T410A") {presence_in_dataset = "    Author  Year  Type  -DGJ  +DGJ  Link    Benjamin et al.  2016  HEK-293  1.0 ± 0.1  11.2 ± 0.4   go     Andreotti et al.  2011  COS-7  4.7 ± 0.3  16.7 ± 1   go    ";}
else if (sostituzione == "T410I") {presence_in_dataset = "    Author  Year  Type  -DGJ  +DGJ  Link    Benjamin et al.  2016  HEK-293  0.4 ± 0.1  12.2 ± 0.8   go     Lukas et al.  2013  HEK293H  2.3 ± 0.8  16.1 ± 3.5   go    ";}
else if (sostituzione == "T410P") {presence_in_dataset = "    Author  Year  Type  -DGJ  +DGJ  Link    Shimotori et al.  2007  COS-7  0.2  0.2   go    ";}
else if (sostituzione == "T412N") {presence_in_dataset = "    Author  Year  Type  -DGJ  +DGJ  Link    Benjamin et al.  2016  HEK-293  1.0 ± 0.1  14.2 ± 0.8   go    ";}
else if (sostituzione == "T41I") {presence_in_dataset = "    Author  Year  Type  -DGJ  +DGJ  Link    Benjamin et al.  2016  HEK-293  68.7 ± 3.1  116.7 ± 7.1   go     Wu et al.  2011  HEK293-Sample-1  43.1 ± 9  70.4 ± 11.1   go     Wu et al.  2011  HEK293-Sample-2  43.1  58.4   go     Benjamin et al.  2009  Lymphoblasts  52 ± 1  129   go     Shin et al.  2008  T-Cell-Sample-1  53 ± 48  156 ± 93   go     Shin et al.  2008  T-Cell-Sample-2  61 ± 33  175 ± 52   go    ";}
else if (sostituzione == "V164G") {presence_in_dataset = "    Author  Year  Type  -DGJ  +DGJ  Link    Benjamin et al.  2016  HEK-293  1.7 ± 0.1  3.2 ± 0.1   go     Lukas et al.  2016  HEK293H  1.4 ± 0.1  2.8 ± 0.5   go    ";}
else if (sostituzione == "V164L") {presence_in_dataset = "    Author  Year  Type  -DGJ  +DGJ  Link    Lukas et al.  2016  HEK293H  43.1 ± 1  47.8 ± 1.1   go    ";}
else if (sostituzione == "V199G") {presence_in_dataset = "    Author  Year  Type  -DGJ  +DGJ  Link    Benjamin et al.  2016  HEK-293  26.0 ± 1.9  62.7 ± 3.8   go    ";}
else if (sostituzione == "V199M") {presence_in_dataset = "    Author  Year  Type  -DGJ  +DGJ  Link    Benjamin et al.  2016  HEK-293  59.7 ± 3.8  108.4 ± 3.9   go    ";}
else if (sostituzione == "V254A") {presence_in_dataset = "    Author  Year  Type  -DGJ  +DGJ  Link    Lukas et al.  2016  HEK293H  26.4 ± 2.4  39.3 ± 2.2   go    ";}
else if (sostituzione == "V269A") {presence_in_dataset = "    Author  Year  Type  -DGJ  +DGJ  Link    Benjamin et al.  2016  HEK-293  NR  7.8 ± 0.7   go     Lukas et al.  2013  HEK293H  9 ± 1.4  45 ± 4.3   go    ";}
else if (sostituzione == "V269G") {presence_in_dataset = "    Author  Year  Type  -DGJ  +DGJ  Link    Lukas et al.  2016  HEK293H  0  0   go    ";}
else if (sostituzione == "V269M") {presence_in_dataset = "    Author  Year  Type  -DGJ  +DGJ  Link    Benjamin et al.  2016  HEK-293  4.4 ± 0.2  25.9 ± 1.2   go     Lukas et al.  2013  HEK293H  0  17.3 ± 1.4   go     Andreotti et al.  2011  COS-7  7.5 ± 0.5  18.5 ± 1.2   go    ";}
else if (sostituzione == "V316A") {presence_in_dataset = "    Author  Year  Type  -DGJ  +DGJ  Link    Lukas et al.  2016  HEK293H  49.1 ± 5.5  58.3 ± 7.7   go    ";}
else if (sostituzione == "V316E") {presence_in_dataset = "    Author  Year  Type  -DGJ  +DGJ  Link    Benjamin et al.  2016  HEK-293  NR  0.8 ± 0.1   go     Wu et al.  2011  HEK293-Sample-1  0  1.8 ± 0.3   go     Benjamin et al.  2009  Lymphoblasts  0  3   go    ";}
else if (sostituzione == "V316G") {presence_in_dataset = "    Author  Year  Type  -DGJ  +DGJ  Link    Benjamin et al.  2016  HEK-293  0.7 ± 0.1  3.8 ± 0.1   go     Lukas et al.  2013  HEK293H  0  0   go    ";}
else if (sostituzione == "V316I") {presence_in_dataset = "    Author  Year  Type  -DGJ  +DGJ  Link    Benjamin et al.  2016  HEK-293  92.1 ± 3.6  126.1 ± 5.5   go     Lukas et al.  2013  HEK293H  65.6 ± 22.3  68.3 ± 21.8   go    ";}
else if (sostituzione == "V339E") {presence_in_dataset = "    Author  Year  Type  -DGJ  +DGJ  Link    Benjamin et al.  2016  HEK-293  11.5 ± 0.6  19.6 ± 0.6   go    ";}
else if (sostituzione == "V339G") {presence_in_dataset = "    Author  Year  Type  -DGJ  +DGJ  Link    Benjamin et al.  2016  HEK-293  NR  1.5 ± 0.1   go    ";}
else if (sostituzione == "V390M") {presence_in_dataset = "    Author  Year  Type  -DGJ  +DGJ  Link    Lukas et al.  2013  HEK293H  0  0   go    ";}
else if (sostituzione == "W162C") {presence_in_dataset = "    Author  Year  Type  -DGJ  +DGJ  Link    Benjamin et al.  2016  HEK-293  0.5 ± 0.1  NR   go    ";}
else if (sostituzione == "W162G") {presence_in_dataset = "    Author  Year  Type  -DGJ  +DGJ  Link    Benjamin et al.  2016  HEK-293  0.8 ± 0.1  5.9 ± 0.4   go     Lukas et al.  2013  HEK293H  0  5.2 ± 1.2   go    ";}
else if (sostituzione == "W162R") {presence_in_dataset = "    Author  Year  Type  -DGJ  +DGJ  Link    Benjamin et al.  2016  HEK-293  NR  1.1 ± 0.1   go     Wu et al.  2011  HEK293-Sample-1  0  0   go     Benjamin et al.  2009  Lymphoblasts  0  0   go    ";}
else if (sostituzione == "W204C") {presence_in_dataset = "    Author  Year  Type  -DGJ  +DGJ  Link    Benjamin et al.  2016  HEK-293  NR  1.1 ± 0.1   go     Lukas et al.  2013  HEK293H  0  4.4 ± 0.9   go    ";}
else if (sostituzione == "W204R") {presence_in_dataset = "    Author  Year  Type  -DGJ  +DGJ  Link    Lukas et al.  2016  HEK293H  0  0   go    ";}
else if (sostituzione == "W226R") {presence_in_dataset = "    Author  Year  Type  -DGJ  +DGJ  Link    Wu et al.  2011  HEK293-Sample-1  0  0   go     Benjamin et al.  2009  Lymphoblasts  0  0   go    ";}
else if (sostituzione == "W236C") {presence_in_dataset = "    Author  Year  Type  -DGJ  +DGJ  Link    Lukas et al.  2013  HEK293H  0  0   go    ";}
else if (sostituzione == "W236R") {presence_in_dataset = "    Author  Year  Type  -DGJ  +DGJ  Link    Shin et al.  2008  T-Cell-Sample-1  1 ± 0  2 ± 1   go    ";}
else if (sostituzione == "W245G") {presence_in_dataset = "    Author  Year  Type  -DGJ  +DGJ  Link    Benjamin et al.  2016  HEK-293  44.1 ± 1.6  63 ± 1.4   go    ";}
else if (sostituzione == "W24C") {presence_in_dataset = "    Author  Year  Type  -DGJ  +DGJ  Link    Benjamin et al.  2016  HEK-293  19.9 ± 1.2  35.0 ± 1.3   go     Lukas et al.  2013  HEK293H  45.6 ± 11.2  89.8 ± 21   go    ";}
else if (sostituzione == "W24G") {presence_in_dataset = "    Author  Year  Type  -DGJ  +DGJ  Link    Benjamin et al.  2016  HEK-293  22.1 ± 1.1  41 ± 1.1   go    ";}
else if (sostituzione == "W24R") {presence_in_dataset = "    Author  Year  Type  -DGJ  +DGJ  Link    Benjamin et al.  2016  HEK-293  52.6 ± 2.3  63.4 ± 1.7   go    ";}
else if (sostituzione == "W262C") {presence_in_dataset = "    Author  Year  Type  -DGJ  +DGJ  Link    Lukas et al.  2013  HEK293H  0  0   go    ";}
else if (sostituzione == "W262L") {presence_in_dataset = "    Author  Year  Type  -DGJ  +DGJ  Link    Benjamin et al.  2016  HEK-293  NR  2.5 ± 0.1   go    ";}
else if (sostituzione == "W262R") {presence_in_dataset = "    Author  Year  Type  -DGJ  +DGJ  Link    Lukas et al.  2016  HEK293H  0  0   go    ";}
else if (sostituzione == "W277C") {presence_in_dataset = "    Author  Year  Type  -DGJ  +DGJ  Link    Benjamin et al.  2016  HEK-293  28.5 ± 1.4  53.7 ± 1.5   go    ";}
else if (sostituzione == "W277G") {presence_in_dataset = "    Author  Year  Type  -DGJ  +DGJ  Link    Benjamin et al.  2016  HEK-293  50.3 ± 2.2  84.9 ± 2.8   go    ";}
else if (sostituzione == "W287C") {presence_in_dataset = "    Author  Year  Type  -DGJ  +DGJ  Link    Wu et al.  2011  HEK293-Sample-1  0  16.4 ± 1.1   go     Benjamin et al.  2009  Lymphoblasts  0.6 ± 0.3  3   go     Shin et al.  2008  T-Cell-Sample-1  3 ± 1  3 ± 1   go     Shin et al.  2008  T-Cell-Sample-2  3 ± 2  3 ± 1   go    ";}
else if (sostituzione == "W349R") {presence_in_dataset = "    Author  Year  Type  -DGJ  +DGJ  Link    Benjamin et al.  2016  HEK-293  NR  0.8 ± 0.1   go     Lukas et al.  2013  HEK293H  1.6 ± 1.4  8.9 ± 3.9   go    ";}
else if (sostituzione == "W349S") {presence_in_dataset = "    Author  Year  Type  -DGJ  +DGJ  Link    Benjamin et al.  2016  HEK-293  16.5 ± 0.7  31.4 ± 1.7   go    ";}
else if (sostituzione == "W399S") {presence_in_dataset = "    Author  Year  Type  -DGJ  +DGJ  Link    Lukas et al.  2016  HEK293H  53 ± 4.3  51.5 ± 5.1   go    ";}
else if (sostituzione == "W81C") {presence_in_dataset = "    Author  Year  Type  -DGJ  +DGJ  Link    Benjamin et al.  2016  HEK-293  NR  0.7 ± 0.0   go    ";}
else if (sostituzione == "W81S") {presence_in_dataset = "    Author  Year  Type  -DGJ  +DGJ  Link    Benjamin et al.  2016  HEK-293  NR  0.6 ± 0.1   go    ";}
else if (sostituzione == "W95C") {presence_in_dataset = "    Author  Year  Type  -DGJ  +DGJ  Link    Lukas et al.  2013  HEK293H  0  0   go    ";}
else if (sostituzione == "W95L") {presence_in_dataset = "    Author  Year  Type  -DGJ  +DGJ  Link    Lukas et al.  2013  HEK293H  0  0   go    ";}
else if (sostituzione == "W95S") {presence_in_dataset = "    Author  Year  Type  -DGJ  +DGJ  Link    Wu et al.  2011  HEK293-Sample-1  0  2.1 ± 0.4   go     Benjamin et al.  2009  Lymphoblasts  1 ± 0.4  26   go    ";}
else if (sostituzione == "Y123C") {presence_in_dataset = "    Author  Year  Type  -DGJ  +DGJ  Link    Benjamin et al.  2016  HEK-293  8.9 ± 0.4  15.1 ± 0.5   go    ";}
else if (sostituzione == "Y152C") {presence_in_dataset = "    Author  Year  Type  -DGJ  +DGJ  Link    Benjamin et al.  2016  HEK-293  16.7 ± 0.6  31.5 ± 1.4   go    ";}
else if (sostituzione == "Y184C") {presence_in_dataset = "    Author  Year  Type  -DGJ  +DGJ  Link    Benjamin et al.  2016  HEK-293  1.7 ± 0.1  7.2 ± 0.8   go    ";}
else if (sostituzione == "Y184N") {presence_in_dataset = "    Author  Year  Type  -DGJ  +DGJ  Link    Benjamin et al.  2016  HEK-293  2.3 ± 0.2  9.7 ± 1   go    ";}
else if (sostituzione == "Y200C") {presence_in_dataset = "    Author  Year  Type  -DGJ  +DGJ  Link    Benjamin et al.  2016  HEK-293  1.3 ± 0.1  14.9 ± 0.9   go    ";}
else if (sostituzione == "Y207C") {presence_in_dataset = "    Author  Year  Type  -DGJ  +DGJ  Link    Wu et al.  2011  HEK293-Sample-1  0  2.7 ± 0.8   go     Benjamin et al.  2009  Lymphoblasts  5 ± 1  11   go    ";}
else if (sostituzione == "Y207H") {presence_in_dataset = "    Author  Year  Type  -DGJ  +DGJ  Link    Benjamin et al.  2016  HEK-293  34.4 ± 2.0  65.5 ± 3.2   go    ";}
else if (sostituzione == "Y207S") {presence_in_dataset = "    Author  Year  Type  -DGJ  +DGJ  Link    Benjamin et al.  2016  HEK-293  2.2 ± 0.1  7.3 ± 0.8   go     Wu et al.  2011  HEK293-Sample-1  1.1 ± 0.4  12.8 ± 1.9   go     Benjamin et al.  2009  Lymphoblasts  4 ± 0.6  9   go     Shin et al.  2008  T-Cell-Sample-1  4 ± 4  15 ± 7   go    ";}
else if (sostituzione == "Y216C") {presence_in_dataset = "    Author  Year  Type  -DGJ  +DGJ  Link    Benjamin et al.  2016  HEK-293  2.0 ± 0.1  20.7 ± 0.8   go     Filoni et al.  2010  COS-1  9.5  41   go     Filoni et al.  2010  Lymphocytes  2.6  9   go    ";}
else if (sostituzione == "Y216D") {presence_in_dataset = "    Author  Year  Type  -DGJ  +DGJ  Link    Benjamin et al.  2016  HEK-293  1.6 ± 0.1  13.3 ± 0.7   go    ";}
else if (sostituzione == "Y86D") {presence_in_dataset = "    Author  Year  Type  -DGJ  +DGJ  Link    Lukas et al.  2013  HEK293H  0  0   go    ";}
else if (sostituzione == "Y86H") {presence_in_dataset = "    Author  Year  Type  -DGJ  +DGJ  Link    Lukas et al.  2016  HEK293H  0  0.7 ± 0.1   go    ";}
else if (sostituzione == "Y88D") {presence_in_dataset = "    Author  Year  Type  -DGJ  +DGJ  Link    Benjamin et al.  2016  HEK-293  NR  1.0 ± 0.0   go    ";}

else {presence_in_dataset = "No";}
					
	
	
	
	
	


var tipo_test;

if (sostituzione == "A108T") {tipo_test ="Yes";}
else if (sostituzione == "A121T") {tipo_test ="Yes";}
else if (sostituzione == "A135V") {tipo_test ="Yes";}
else if (sostituzione == "A13P") {tipo_test ="Yes";}
else if (sostituzione == "A13T") {tipo_test ="Yes";}
else if (sostituzione == "A143P") {tipo_test ="Yes";}
else if (sostituzione == "A143T") {tipo_test ="Yes";}
else if (sostituzione == "A156D") {tipo_test ="Yes";}
else if (sostituzione == "A156T") {tipo_test ="Yes";}
else if (sostituzione == "A156V") {tipo_test ="Yes";}
else if (sostituzione == "A15E") {tipo_test ="Yes";}
else if (sostituzione == "A15G") {tipo_test ="Yes";}
else if (sostituzione == "A15P") {tipo_test ="Yes";}
else if (sostituzione == "A15T") {tipo_test ="Yes";}
else if (sostituzione == "A20D") {tipo_test ="Yes";}
else if (sostituzione == "A20P") {tipo_test ="Yes";}
else if (sostituzione == "A20V") {tipo_test ="Yes";}
else if (sostituzione == "A230T") {tipo_test ="Yes";}
else if (sostituzione == "A257D") {tipo_test ="Yes";}
else if (sostituzione == "A257G") {tipo_test ="Yes";}
else if (sostituzione == "A257P") {tipo_test ="Yes";}
else if (sostituzione == "A285D") {tipo_test ="Yes";}
else if (sostituzione == "A288D") {tipo_test ="Yes";}
else if (sostituzione == "A288P") {tipo_test ="Yes";}
else if (sostituzione == "A291T") {tipo_test ="Yes";}
else if (sostituzione == "A292T") {tipo_test ="Yes";}
else if (sostituzione == "A307T") {tipo_test ="Yes";}
else if (sostituzione == "A309P") {tipo_test ="Yes";}
else if (sostituzione == "A309V") {tipo_test ="Yes";}
else if (sostituzione == "A31V") {tipo_test ="Yes";}
else if (sostituzione == "A348P") {tipo_test ="Yes";}
else if (sostituzione == "A352D") {tipo_test ="Yes";}
else if (sostituzione == "A352G") {tipo_test ="Yes";}
else if (sostituzione == "A352V") {tipo_test ="Yes";}
else if (sostituzione == "A368T") {tipo_test ="Yes";}
else if (sostituzione == "A377D") {tipo_test ="Yes";}
else if (sostituzione == "A37T") {tipo_test ="Yes";}
else if (sostituzione == "A37V") {tipo_test ="Yes";}
else if (sostituzione == "A73E") {tipo_test ="Yes";}
else if (sostituzione == "A73V") {tipo_test ="Yes";}
else if (sostituzione == "A97P") {tipo_test ="Yes";}
else if (sostituzione == "A97V") {tipo_test ="Yes";}
else if (sostituzione == "C142R") {tipo_test ="Yes";}
else if (sostituzione == "C142W") {tipo_test ="Yes";}
else if (sostituzione == "C172G") {tipo_test ="Yes";}
else if (sostituzione == "C172Y") {tipo_test ="Yes";}
else if (sostituzione == "C174G") {tipo_test ="Yes";}
else if (sostituzione == "C174R") {tipo_test ="Yes";}
else if (sostituzione == "C202Y") {tipo_test ="Yes";}
else if (sostituzione == "C378R") {tipo_test ="Yes";}
else if (sostituzione == "C52G") {tipo_test ="Yes";}
else if (sostituzione == "C52W") {tipo_test ="Yes";}
else if (sostituzione == "C52Y") {tipo_test ="Yes";}
else if (sostituzione == "C56F") {tipo_test ="Yes";}
else if (sostituzione == "C56Y") {tipo_test ="Yes";}
else if (sostituzione == "C63Y") {tipo_test ="Yes";}
else if (sostituzione == "C94S") {tipo_test ="Yes";}
else if (sostituzione == "C94Y") {tipo_test ="Yes";}
else if (sostituzione == "D109G") {tipo_test ="Yes";}
else if (sostituzione == "D136E") {tipo_test ="Yes";}
else if (sostituzione == "D136H") {tipo_test ="Yes";}
else if (sostituzione == "D155H") {tipo_test ="Yes";}
else if (sostituzione == "D165G") {tipo_test ="Yes";}
else if (sostituzione == "D165H") {tipo_test ="Yes";}
else if (sostituzione == "D165V") {tipo_test ="Yes";}
else if (sostituzione == "D165Y") {tipo_test ="Yes";}
else if (sostituzione == "D170N") {tipo_test ="Yes";}
else if (sostituzione == "D170V") {tipo_test ="Yes";}
else if (sostituzione == "D175E") {tipo_test ="Yes";}
else if (sostituzione == "D175N") {tipo_test ="Yes";}
else if (sostituzione == "D231G") {tipo_test ="Yes";}
else if (sostituzione == "D231N") {tipo_test ="Yes";}
else if (sostituzione == "D231V") {tipo_test ="Yes";}
else if (sostituzione == "D234E") {tipo_test ="Yes";}
else if (sostituzione == "D244H") {tipo_test ="Yes";}
else if (sostituzione == "D244N") {tipo_test ="Yes";}
else if (sostituzione == "D264A") {tipo_test ="Yes";}
else if (sostituzione == "D264E") {tipo_test ="Yes";}
else if (sostituzione == "D264G") {tipo_test ="Yes";}
else if (sostituzione == "D264H") {tipo_test ="Yes";}
else if (sostituzione == "D264N") {tipo_test ="Yes";}
else if (sostituzione == "D264V") {tipo_test ="Yes";}
else if (sostituzione == "D264Y") {tipo_test ="Yes";}
else if (sostituzione == "D266N") {tipo_test ="Yes";}
else if (sostituzione == "D266V") {tipo_test ="Yes";}
else if (sostituzione == "D299E") {tipo_test ="Yes";}
else if (sostituzione == "D313G") {tipo_test ="Yes";}
else if (sostituzione == "D313N") {tipo_test ="Yes";}
else if (sostituzione == "D313Y") {tipo_test ="Yes";}
else if (sostituzione == "D315N") {tipo_test ="Yes";}
else if (sostituzione == "D322E") {tipo_test ="Yes";}
else if (sostituzione == "D322N") {tipo_test ="Yes";}
else if (sostituzione == "D33G") {tipo_test ="Yes";}
else if (sostituzione == "D33Y") {tipo_test ="Yes";}
else if (sostituzione == "D55V") {tipo_test ="Yes";}
else if (sostituzione == "D83N") {tipo_test ="Yes";}
else if (sostituzione == "D92N") {tipo_test ="Yes";}
else if (sostituzione == "D92Y") {tipo_test ="Yes";}
else if (sostituzione == "D93E") {tipo_test ="Yes";}
else if (sostituzione == "D93V") {tipo_test ="Yes";}
else if (sostituzione == "D93Y") {tipo_test ="Yes";}
else if (sostituzione == "E103Q") {tipo_test ="Yes";}
else if (sostituzione == "E203D") {tipo_test ="Yes";}
else if (sostituzione == "E203V") {tipo_test ="Yes";}
else if (sostituzione == "E338K") {tipo_test ="Yes";}
else if (sostituzione == "E341D") {tipo_test ="Yes";}
else if (sostituzione == "E341K") {tipo_test ="Yes";}
else if (sostituzione == "E358A") {tipo_test ="Yes";}
else if (sostituzione == "E358D") {tipo_test ="Yes";}
else if (sostituzione == "E358G") {tipo_test ="Yes";}
else if (sostituzione == "E358K") {tipo_test ="Yes";}
else if (sostituzione == "E398A") {tipo_test ="Yes";}
else if (sostituzione == "E398K") {tipo_test ="Yes";}
else if (sostituzione == "E418G") {tipo_test ="Yes";}
else if (sostituzione == "E48D") {tipo_test ="Yes";}
else if (sostituzione == "E48K") {tipo_test ="Yes";}
else if (sostituzione == "E48Q") {tipo_test ="Yes";}
else if (sostituzione == "E59K") {tipo_test ="Yes";}
else if (sostituzione == "E66D") {tipo_test ="Yes";}
else if (sostituzione == "E66G") {tipo_test ="Yes";}
else if (sostituzione == "E66K") {tipo_test ="Yes";}
else if (sostituzione == "E66Q") {tipo_test ="Yes";}
else if (sostituzione == "E71G") {tipo_test ="Yes";}
else if (sostituzione == "F113I") {tipo_test ="Yes";}
else if (sostituzione == "F113L") {tipo_test ="Yes";}
else if (sostituzione == "F113S") {tipo_test ="Yes";}
else if (sostituzione == "F169S") {tipo_test ="Yes";}
else if (sostituzione == "F18S") {tipo_test ="Yes";}
else if (sostituzione == "F229L") {tipo_test ="Yes";}
else if (sostituzione == "F273L") {tipo_test ="Yes";}
else if (sostituzione == "F295C") {tipo_test ="Yes";}
else if (sostituzione == "F396Y") {tipo_test ="Yes";}
else if (sostituzione == "F50C") {tipo_test ="Yes";}
else if (sostituzione == "G104V") {tipo_test ="Yes";}
else if (sostituzione == "G128E") {tipo_test ="Yes";}
else if (sostituzione == "G132A") {tipo_test ="Yes";}
else if (sostituzione == "G132E") {tipo_test ="Yes";}
else if (sostituzione == "G132R") {tipo_test ="Yes";}
else if (sostituzione == "G138E") {tipo_test ="Yes";}
else if (sostituzione == "G138R") {tipo_test ="Yes";}
else if (sostituzione == "G144D") {tipo_test ="Yes";}
else if (sostituzione == "G144V") {tipo_test ="Yes";}
else if (sostituzione == "G147R") {tipo_test ="Yes";}
else if (sostituzione == "G163V") {tipo_test ="Yes";}
else if (sostituzione == "G171D") {tipo_test ="Yes";}
else if (sostituzione == "G171R") {tipo_test ="Yes";}
else if (sostituzione == "G183A") {tipo_test ="Yes";}
else if (sostituzione == "G183D") {tipo_test ="Yes";}
else if (sostituzione == "G183S") {tipo_test ="Yes";}
else if (sostituzione == "G183V") {tipo_test ="Yes";}
else if (sostituzione == "G195V") {tipo_test ="Yes";}
else if (sostituzione == "G258R") {tipo_test ="Yes";}
else if (sostituzione == "G258V") {tipo_test ="Yes";}
else if (sostituzione == "G260A") {tipo_test ="Yes";}
else if (sostituzione == "G260E") {tipo_test ="Yes";}
else if (sostituzione == "G261D") {tipo_test ="Yes";}
else if (sostituzione == "G261V") {tipo_test ="Yes";}
else if (sostituzione == "G271C") {tipo_test ="Yes";}
else if (sostituzione == "G271D") {tipo_test ="Yes";}
else if (sostituzione == "G271S") {tipo_test ="Yes";}
else if (sostituzione == "G271V") {tipo_test ="Yes";}
else if (sostituzione == "G325D") {tipo_test ="Yes";}
else if (sostituzione == "G325R") {tipo_test ="Yes";}
else if (sostituzione == "G325S") {tipo_test ="Yes";}
else if (sostituzione == "G328A") {tipo_test ="Yes";}
else if (sostituzione == "G328R") {tipo_test ="Yes";}
else if (sostituzione == "G334E") {tipo_test ="Yes";}
else if (sostituzione == "G35E") {tipo_test ="Yes";}
else if (sostituzione == "G35R") {tipo_test ="Yes";}
else if (sostituzione == "G360C") {tipo_test ="Yes";}
else if (sostituzione == "G360D") {tipo_test ="Yes";}
else if (sostituzione == "G360S") {tipo_test ="Yes";}
else if (sostituzione == "G361A") {tipo_test ="Yes";}
else if (sostituzione == "G361E") {tipo_test ="Yes";}
else if (sostituzione == "G361R") {tipo_test ="Yes";}
else if (sostituzione == "G373D") {tipo_test ="Yes";}
else if (sostituzione == "G373S") {tipo_test ="Yes";}
else if (sostituzione == "G375A") {tipo_test ="Yes";}
else if (sostituzione == "G395A") {tipo_test ="Yes";}
else if (sostituzione == "G395E") {tipo_test ="Yes";}
else if (sostituzione == "G411D") {tipo_test ="Yes";}
else if (sostituzione == "G43S") {tipo_test ="Yes";}
else if (sostituzione == "G80D") {tipo_test ="Yes";}
else if (sostituzione == "G85D") {tipo_test ="Yes";}
else if (sostituzione == "G85M") {tipo_test ="Yes";}
else if (sostituzione == "G85S") {tipo_test ="Yes";}
else if (sostituzione == "H125L") {tipo_test ="Yes";}
else if (sostituzione == "H125P") {tipo_test ="Yes";}
else if (sostituzione == "H225D") {tipo_test ="Yes";}
else if (sostituzione == "H225R") {tipo_test ="Yes";}
else if (sostituzione == "H406R") {tipo_test ="Yes";}
else if (sostituzione == "H46P") {tipo_test ="Yes";}
else if (sostituzione == "H46R") {tipo_test ="Yes";}
else if (sostituzione == "H46Y") {tipo_test ="Yes";}
else if (sostituzione == "I117S") {tipo_test ="Yes";}
else if (sostituzione == "I154T") {tipo_test ="Yes";}
else if (sostituzione == "I198T") {tipo_test ="Yes";}
else if (sostituzione == "I219M") {tipo_test ="Yes";}
else if (sostituzione == "I219N") {tipo_test ="Yes";}
else if (sostituzione == "I219T") {tipo_test ="Yes";}
else if (sostituzione == "I232T") {tipo_test ="Yes";}
else if (sostituzione == "I239T") {tipo_test ="Yes";}
else if (sostituzione == "I242F") {tipo_test ="Yes";}
else if (sostituzione == "I242N") {tipo_test ="Yes";}
else if (sostituzione == "I242V") {tipo_test ="Yes";}
else if (sostituzione == "I253S") {tipo_test ="Yes";}
else if (sostituzione == "I253T") {tipo_test ="Yes";}
else if (sostituzione == "I270M") {tipo_test ="Yes";}
else if (sostituzione == "I270T") {tipo_test ="Yes";}
else if (sostituzione == "I289F") {tipo_test ="Yes";}
else if (sostituzione == "I289S") {tipo_test ="Yes";}
else if (sostituzione == "I289V") {tipo_test ="Yes";}
else if (sostituzione == "I303N") {tipo_test ="Yes";}
else if (sostituzione == "I317N") {tipo_test ="Yes";}
else if (sostituzione == "I317S") {tipo_test ="Yes";}
else if (sostituzione == "I317T") {tipo_test ="Yes";}
else if (sostituzione == "I319F") {tipo_test ="Yes";}
else if (sostituzione == "I319T") {tipo_test ="Yes";}
else if (sostituzione == "I354K") {tipo_test ="Yes";}
else if (sostituzione == "I359T") {tipo_test ="Yes";}
else if (sostituzione == "I384N") {tipo_test ="Yes";}
else if (sostituzione == "I407V") {tipo_test ="Yes";}
else if (sostituzione == "I64F") {tipo_test ="Yes";}
else if (sostituzione == "I91N") {tipo_test ="Yes";}
else if (sostituzione == "I91T") {tipo_test ="Yes";}
else if (sostituzione == "K130R") {tipo_test ="Yes";}
else if (sostituzione == "K140T") {tipo_test ="Yes";}
else if (sostituzione == "K185E") {tipo_test ="Yes";}
else if (sostituzione == "K213M") {tipo_test ="Yes";}
else if (sostituzione == "K213R") {tipo_test ="Yes";}
else if (sostituzione == "K308N") {tipo_test ="Yes";}
else if (sostituzione == "K391T") {tipo_test ="Yes";}
else if (sostituzione == "L120P") {tipo_test ="Yes";}
else if (sostituzione == "L120S") {tipo_test ="Yes";}
else if (sostituzione == "L120V") {tipo_test ="Yes";}
else if (sostituzione == "L129P") {tipo_test ="Yes";}
else if (sostituzione == "L131P") {tipo_test ="Yes";}
else if (sostituzione == "L14P") {tipo_test ="Yes";}
else if (sostituzione == "L166G") {tipo_test ="Yes";}
else if (sostituzione == "L166V") {tipo_test ="Yes";}
else if (sostituzione == "L167Q") {tipo_test ="Yes";}
else if (sostituzione == "L180F") {tipo_test ="Yes";}
else if (sostituzione == "L191P") {tipo_test ="Yes";}
else if (sostituzione == "L191Q") {tipo_test ="Yes";}
else if (sostituzione == "L206P") {tipo_test ="Yes";}
else if (sostituzione == "L21F") {tipo_test ="Yes";}
else if (sostituzione == "L21P") {tipo_test ="Yes";}
else if (sostituzione == "L21R") {tipo_test ="Yes";}
else if (sostituzione == "L243F") {tipo_test ="Yes";}
else if (sostituzione == "L243W") {tipo_test ="Yes";}
else if (sostituzione == "L268S") {tipo_test ="Yes";}
else if (sostituzione == "L275F") {tipo_test ="Yes";}
else if (sostituzione == "L275H") {tipo_test ="Yes";}
else if (sostituzione == "L294S") {tipo_test ="Yes";}
else if (sostituzione == "L300F") {tipo_test ="Yes";}
else if (sostituzione == "L300P") {tipo_test ="Yes";}
else if (sostituzione == "L310F") {tipo_test ="Yes";}
else if (sostituzione == "L310R") {tipo_test ="Yes";}
else if (sostituzione == "L311F") {tipo_test ="Yes";}
else if (sostituzione == "L311V") {tipo_test ="Yes";}
else if (sostituzione == "L32P") {tipo_test ="Yes";}
else if (sostituzione == "L344P") {tipo_test ="Yes";}
else if (sostituzione == "L36F") {tipo_test ="Yes";}
else if (sostituzione == "L36S") {tipo_test ="Yes";}
else if (sostituzione == "L36W") {tipo_test ="Yes";}
else if (sostituzione == "L372P") {tipo_test ="Yes";}
else if (sostituzione == "L372Q") {tipo_test ="Yes";}
else if (sostituzione == "L372R") {tipo_test ="Yes";}
else if (sostituzione == "L3P") {tipo_test ="Yes";}
else if (sostituzione == "L3V") {tipo_test ="Yes";}
else if (sostituzione == "L403S") {tipo_test ="Yes";}
else if (sostituzione == "L414S") {tipo_test ="Yes";}
else if (sostituzione == "L415F") {tipo_test ="Yes";}
else if (sostituzione == "L415P") {tipo_test ="Yes";}
else if (sostituzione == "L45P") {tipo_test ="Yes";}
else if (sostituzione == "L45R") {tipo_test ="Yes";}
else if (sostituzione == "L54F") {tipo_test ="Yes";}
else if (sostituzione == "L54P") {tipo_test ="Yes";}
else if (sostituzione == "L68F") {tipo_test ="Yes";}
else if (sostituzione == "L89F") {tipo_test ="Yes";}
else if (sostituzione == "L89H") {tipo_test ="Yes";}
else if (sostituzione == "L89R") {tipo_test ="Yes";}
else if (sostituzione == "M187I") {tipo_test ="Yes";}
else if (sostituzione == "M187T") {tipo_test ="Yes";}
else if (sostituzione == "M187V") {tipo_test ="Yes";}
else if (sostituzione == "M1I") {tipo_test ="Yes";}
else if (sostituzione == "M1L") {tipo_test ="Yes";}
else if (sostituzione == "M1R") {tipo_test ="Yes";}
else if (sostituzione == "M1T") {tipo_test ="Yes";}
else if (sostituzione == "M1V") {tipo_test ="Yes";}
else if (sostituzione == "M267T") {tipo_test ="Yes";}
else if (sostituzione == "M284T") {tipo_test ="Yes";}
else if (sostituzione == "M284V") {tipo_test ="Yes";}
else if (sostituzione == "M290I") {tipo_test ="Yes";}
else if (sostituzione == "M290L") {tipo_test ="Yes";}
else if (sostituzione == "M296I") {tipo_test ="Yes";}
else if (sostituzione == "M296L") {tipo_test ="Yes";}
else if (sostituzione == "M296T") {tipo_test ="Yes";}
else if (sostituzione == "M296V") {tipo_test ="Yes";}
else if (sostituzione == "M421V") {tipo_test ="Yes";}
else if (sostituzione == "M42K") {tipo_test ="Yes";}
else if (sostituzione == "M42L") {tipo_test ="Yes";}
else if (sostituzione == "M42R") {tipo_test ="Yes";}
else if (sostituzione == "M42T") {tipo_test ="Yes";}
else if (sostituzione == "M42V") {tipo_test ="Yes";}
else if (sostituzione == "M51I") {tipo_test ="Yes";}
else if (sostituzione == "M51K") {tipo_test ="Yes";}
else if (sostituzione == "M72I") {tipo_test ="Yes";}
else if (sostituzione == "M72V") {tipo_test ="Yes";}
else if (sostituzione == "M76T") {tipo_test ="Yes";}
else if (sostituzione == "M96I") {tipo_test ="Yes";}
else if (sostituzione == "N139S") {tipo_test ="Yes";}
else if (sostituzione == "N139T") {tipo_test ="Yes";}
else if (sostituzione == "N215D") {tipo_test ="Yes";}
else if (sostituzione == "N215S") {tipo_test ="Yes";}
else if (sostituzione == "N224D") {tipo_test ="Yes";}
else if (sostituzione == "N224S") {tipo_test ="Yes";}
else if (sostituzione == "N228S") {tipo_test ="Yes";}
else if (sostituzione == "N249K") {tipo_test ="Yes";}
else if (sostituzione == "N263S") {tipo_test ="Yes";}
else if (sostituzione == "N272K") {tipo_test ="Yes";}
else if (sostituzione == "N272S") {tipo_test ="Yes";}
else if (sostituzione == "N298S") {tipo_test ="Yes";}
else if (sostituzione == "N320I") {tipo_test ="Yes";}
else if (sostituzione == "N320Y") {tipo_test ="Yes";}
else if (sostituzione == "N34K") {tipo_test ="Yes";}
else if (sostituzione == "N34S") {tipo_test ="Yes";}
else if (sostituzione == "N53D") {tipo_test ="Yes";}
else if (sostituzione == "N53L") {tipo_test ="Yes";}
else if (sostituzione == "P146S") {tipo_test ="Yes";}
else if (sostituzione == "P205L") {tipo_test ="Yes";}
else if (sostituzione == "P205R") {tipo_test ="Yes";}
else if (sostituzione == "P205S") {tipo_test ="Yes";}
else if (sostituzione == "P205T") {tipo_test ="Yes";}
else if (sostituzione == "P210L") {tipo_test ="Yes";}
else if (sostituzione == "P210S") {tipo_test ="Yes";}
else if (sostituzione == "P214L") {tipo_test ="Yes";}
else if (sostituzione == "P214S") {tipo_test ="Yes";}
else if (sostituzione == "P259L") {tipo_test ="Yes";}
else if (sostituzione == "P259R") {tipo_test ="Yes";}
else if (sostituzione == "P265L") {tipo_test ="Yes";}
else if (sostituzione == "P265R") {tipo_test ="Yes";}
else if (sostituzione == "P265S") {tipo_test ="Yes";}
else if (sostituzione == "P293A") {tipo_test ="Yes";}
else if (sostituzione == "P293S") {tipo_test ="Yes";}
else if (sostituzione == "P293T") {tipo_test ="Yes";}
else if (sostituzione == "P323R") {tipo_test ="Yes";}
else if (sostituzione == "P343L") {tipo_test ="Yes";}
else if (sostituzione == "P362L") {tipo_test ="Yes";}
else if (sostituzione == "P362T") {tipo_test ="Yes";}
else if (sostituzione == "P389A") {tipo_test ="Yes";}
else if (sostituzione == "P389L") {tipo_test ="Yes";}
else if (sostituzione == "P409A") {tipo_test ="Yes";}
else if (sostituzione == "P409S") {tipo_test ="Yes";}
else if (sostituzione == "P409T") {tipo_test ="Yes";}
else if (sostituzione == "P40A") {tipo_test ="Yes";}
else if (sostituzione == "P40S") {tipo_test ="Yes";}
else if (sostituzione == "P60L") {tipo_test ="Yes";}
else if (sostituzione == "P60S") {tipo_test ="Yes";}
else if (sostituzione == "P60T") {tipo_test ="Yes";}
else if (sostituzione == "Q107L") {tipo_test ="Yes";}
else if (sostituzione == "Q250P") {tipo_test ="Yes";}
else if (sostituzione == "Q279E") {tipo_test ="Yes";}
else if (sostituzione == "Q279H") {tipo_test ="Yes";}
else if (sostituzione == "Q280H") {tipo_test ="Yes";}
else if (sostituzione == "Q280K") {tipo_test ="Yes";}
else if (sostituzione == "Q283P") {tipo_test ="Yes";}
else if (sostituzione == "Q312H") {tipo_test ="Yes";}
else if (sostituzione == "Q312R") {tipo_test ="Yes";}
else if (sostituzione == "Q321E") {tipo_test ="Yes";}
else if (sostituzione == "Q321H") {tipo_test ="Yes";}
else if (sostituzione == "Q321L") {tipo_test ="Yes";}
else if (sostituzione == "Q321R") {tipo_test ="Yes";}
else if (sostituzione == "Q327E") {tipo_test ="Yes";}
else if (sostituzione == "Q327K") {tipo_test ="Yes";}
else if (sostituzione == "Q327L") {tipo_test ="Yes";}
else if (sostituzione == "Q327R") {tipo_test ="Yes";}
else if (sostituzione == "Q330R") {tipo_test ="Yes";}
else if (sostituzione == "Q333R ") {tipo_test ="Yes";}
else if (sostituzione == "Q386P") {tipo_test ="Yes";}
else if (sostituzione == "Q416P") {tipo_test ="Yes";}
else if (sostituzione == "Q57L") {tipo_test ="Yes";}
else if (sostituzione == "R100K") {tipo_test ="Yes";}
else if (sostituzione == "R100T") {tipo_test ="Yes";}
else if (sostituzione == "R112C") {tipo_test ="Yes";}
else if (sostituzione == "R112G") {tipo_test ="Yes";}
else if (sostituzione == "R112H") {tipo_test ="Yes";}
else if (sostituzione == "R112S") {tipo_test ="Yes";}
else if (sostituzione == "R118C") {tipo_test ="Yes";}
else if (sostituzione == "R118G") {tipo_test ="Yes";}
else if (sostituzione == "R118H") {tipo_test ="Yes";}
else if (sostituzione == "R118L") {tipo_test ="Yes";}
else if (sostituzione == "R118P") {tipo_test ="Yes";}
else if (sostituzione == "R118S") {tipo_test ="Yes";}
else if (sostituzione == "R196S") {tipo_test ="Yes";}
else if (sostituzione == "R220P") {tipo_test ="Yes";}
else if (sostituzione == "R220Q") {tipo_test ="Yes";}
else if (sostituzione == "R227P") {tipo_test ="Yes";}
else if (sostituzione == "R227Q") {tipo_test ="Yes";}
else if (sostituzione == "R252T") {tipo_test ="Yes";}
else if (sostituzione == "R301G") {tipo_test ="Yes";}
else if (sostituzione == "R301L") {tipo_test ="Yes";}
else if (sostituzione == "R301P") {tipo_test ="Yes";}
else if (sostituzione == "R301Q") {tipo_test ="Yes";}
else if (sostituzione == "R342L") {tipo_test ="Yes";}
else if (sostituzione == "R342P") {tipo_test ="Yes";}
else if (sostituzione == "R342Q") {tipo_test ="Yes";}
else if (sostituzione == "R356G") {tipo_test ="Yes";}
else if (sostituzione == "R356P") {tipo_test ="Yes";}
else if (sostituzione == "R356Q") {tipo_test ="Yes";}
else if (sostituzione == "R356W") {tipo_test ="Yes";}
else if (sostituzione == "R363C") {tipo_test ="Yes";}
else if (sostituzione == "R363H") {tipo_test ="Yes";}
else if (sostituzione == "R363P") {tipo_test ="Yes";}
else if (sostituzione == "R392S") {tipo_test ="Yes";}
else if (sostituzione == "R49C") {tipo_test ="Yes";}
else if (sostituzione == "R49G") {tipo_test ="Yes";}
else if (sostituzione == "R49L") {tipo_test ="Yes";}
else if (sostituzione == "S102L") {tipo_test ="Yes";}
else if (sostituzione == "S126C") {tipo_test ="Yes";}
else if (sostituzione == "S126G") {tipo_test ="Yes";}
else if (sostituzione == "S126I") {tipo_test ="Yes";}
else if (sostituzione == "S126N") {tipo_test ="Yes";}
else if (sostituzione == "S126R") {tipo_test ="Yes";}
else if (sostituzione == "S126T") {tipo_test ="Yes";}
else if (sostituzione == "S148N") {tipo_test ="Yes";}
else if (sostituzione == "S148R") {tipo_test ="Yes";}
else if (sostituzione == "S201F") {tipo_test ="Yes";}
else if (sostituzione == "S201Y") {tipo_test ="Yes";}
else if (sostituzione == "S235C") {tipo_test ="Yes";}
else if (sostituzione == "S235F") {tipo_test ="Yes";}
else if (sostituzione == "S238N") {tipo_test ="Yes";}
else if (sostituzione == "S238R") {tipo_test ="Yes";}
else if (sostituzione == "S247C") {tipo_test ="Yes";}
else if (sostituzione == "S247P") {tipo_test ="Yes";}
else if (sostituzione == "S276G") {tipo_test ="Yes";}
else if (sostituzione == "S276N") {tipo_test ="Yes";}
else if (sostituzione == "S297C") {tipo_test ="Yes";}
else if (sostituzione == "S297F") {tipo_test ="Yes";}
else if (sostituzione == "S304N") {tipo_test ="Yes";}
else if (sostituzione == "S304T") {tipo_test ="Yes";}
else if (sostituzione == "S345P") {tipo_test ="Yes";}
else if (sostituzione == "S405R") {tipo_test ="Yes";}
else if (sostituzione == "S65A") {tipo_test ="Yes";}
else if (sostituzione == "S65I") {tipo_test ="Yes";}
else if (sostituzione == "S65T") {tipo_test ="Yes";}
else if (sostituzione == "T141I") {tipo_test ="Yes";}
else if (sostituzione == "T194I") {tipo_test ="Yes";}
else if (sostituzione == "T282A") {tipo_test ="Yes";}
else if (sostituzione == "T282I") {tipo_test ="Yes";}
else if (sostituzione == "T385A") {tipo_test ="Yes";}
else if (sostituzione == "T410A") {tipo_test ="Yes";}
else if (sostituzione == "T410I") {tipo_test ="Yes";}
else if (sostituzione == "T410P") {tipo_test ="Yes";}
else if (sostituzione == "T412N") {tipo_test ="Yes";}
else if (sostituzione == "T41I") {tipo_test ="Yes";}
else if (sostituzione == "V164G") {tipo_test ="Yes";}
else if (sostituzione == "V164L") {tipo_test ="Yes";}
else if (sostituzione == "V199G") {tipo_test ="Yes";}
else if (sostituzione == "V199M") {tipo_test ="Yes";}
else if (sostituzione == "V254A") {tipo_test ="Yes";}
else if (sostituzione == "V269A") {tipo_test ="Yes";}
else if (sostituzione == "V269G") {tipo_test ="Yes";}
else if (sostituzione == "V269M") {tipo_test ="Yes";}
else if (sostituzione == "V316A") {tipo_test ="Yes";}
else if (sostituzione == "V316E") {tipo_test ="Yes";}
else if (sostituzione == "V316G") {tipo_test ="Yes";}
else if (sostituzione == "V316I") {tipo_test ="Yes";}
else if (sostituzione == "V339E") {tipo_test ="Yes";}
else if (sostituzione == "V339G") {tipo_test ="Yes";}
else if (sostituzione == "V390M") {tipo_test ="Yes";}
else if (sostituzione == "W162C") {tipo_test ="Yes";}
else if (sostituzione == "W162G") {tipo_test ="Yes";}
else if (sostituzione == "W162R") {tipo_test ="Yes";}
else if (sostituzione == "W204C") {tipo_test ="Yes";}
else if (sostituzione == "W204R") {tipo_test ="Yes";}
else if (sostituzione == "W226R") {tipo_test ="Yes";}
else if (sostituzione == "W236C") {tipo_test ="Yes";}
else if (sostituzione == "W236R") {tipo_test ="Yes";}
else if (sostituzione == "W245G") {tipo_test ="Yes";}
else if (sostituzione == "W24C") {tipo_test ="Yes";}
else if (sostituzione == "W24G") {tipo_test ="Yes";}
else if (sostituzione == "W24R") {tipo_test ="Yes";}
else if (sostituzione == "W262C") {tipo_test ="Yes";}
else if (sostituzione == "W262L") {tipo_test ="Yes";}
else if (sostituzione == "W262R") {tipo_test ="Yes";}
else if (sostituzione == "W277C") {tipo_test ="Yes";}
else if (sostituzione == "W277G") {tipo_test ="Yes";}
else if (sostituzione == "W287C") {tipo_test ="Yes";}
else if (sostituzione == "W349R") {tipo_test ="Yes";}
else if (sostituzione == "W349S") {tipo_test ="Yes";}
else if (sostituzione == "W399S") {tipo_test ="Yes";}
else if (sostituzione == "W81C") {tipo_test ="Yes";}
else if (sostituzione == "W81S") {tipo_test ="Yes";}
else if (sostituzione == "W95C") {tipo_test ="Yes";}
else if (sostituzione == "W95L") {tipo_test ="Yes";}
else if (sostituzione == "W95S") {tipo_test ="Yes";}
else if (sostituzione == "Y123C") {tipo_test ="Yes";}
else if (sostituzione == "Y152C") {tipo_test ="Yes";}
else if (sostituzione == "Y184C") {tipo_test ="Yes";}
else if (sostituzione == "Y184N") {tipo_test ="Yes";}
else if (sostituzione == "Y200C") {tipo_test ="Yes";}
else if (sostituzione == "Y207C") {tipo_test ="Yes";}
else if (sostituzione == "Y207H") {tipo_test ="Yes";}
else if (sostituzione == "Y207S") {tipo_test ="Yes";}
else if (sostituzione == "Y216C") {tipo_test ="Yes";}
else if (sostituzione == "Y216D") {tipo_test ="Yes";}
else if (sostituzione == "Y86D") {tipo_test ="Yes";}
else if (sostituzione == "Y86H") {tipo_test ="Yes";}
else if (sostituzione == "Y88D") {tipo_test ="Yes";}


else {tipo_test = "No";}


var galafold;

if (sostituzione == "A108T") {galafold ="Responsive";}
else if (sostituzione == "A121T") {galafold ="Responsive";}
else if (sostituzione == "A135V") {galafold ="Responsive";}
else if (sostituzione == "A13P") {galafold ="Responsive";}
else if (sostituzione == "A13T") {galafold ="Responsive";}
else if (sostituzione == "A143P") {galafold ="Non Responsive";}
else if (sostituzione == "A143T") {galafold ="Responsive";}
else if (sostituzione == "A156D") {galafold ="Non Responsive";}
else if (sostituzione == "A156T") {galafold ="Responsive";}
else if (sostituzione == "A156V") {galafold ="Responsive";}
else if (sostituzione == "A15E") {galafold ="Not Reported";}
else if (sostituzione == "A15G") {galafold ="Responsive";}
else if (sostituzione == "A15P") {galafold ="Non Responsive";}
else if (sostituzione == "A15T") {galafold ="Responsive";}
else if (sostituzione == "A20D") {galafold ="Responsive";}
else if (sostituzione == "A20P") {galafold ="Responsive";}
else if (sostituzione == "A20V") {galafold ="Non Responsive";}
else if (sostituzione == "A230T") {galafold ="Non Responsive";}
else if (sostituzione == "A257D") {galafold ="Non Responsive";}
else if (sostituzione == "A257G") {galafold ="Responsive";}
else if (sostituzione == "A257P") {galafold ="Responsive";}
else if (sostituzione == "A285D") {galafold ="Non Responsive";}
else if (sostituzione == "A288D") {galafold ="Non Responsive";}
else if (sostituzione == "A288P") {galafold ="Responsive";}
else if (sostituzione == "A291T") {galafold ="Responsive";}
else if (sostituzione == "A292T") {galafold ="Non Responsive";}
else if (sostituzione == "A307T") {galafold ="Responsive";}
else if (sostituzione == "A309P") {galafold ="Responsive";}
else if (sostituzione == "A309V") {galafold ="Not Reported";}
else if (sostituzione == "A31V") {galafold ="Non Responsive";}
else if (sostituzione == "A348P") {galafold ="Non Responsive";}
else if (sostituzione == "A352D") {galafold ="Non Responsive";}
else if (sostituzione == "A352G") {galafold ="Not Reported";}
else if (sostituzione == "A352V") {galafold ="Responsive";}
else if (sostituzione == "A368T") {galafold ="Responsive";}
else if (sostituzione == "A377D") {galafold ="Non Responsive";}
else if (sostituzione == "A37T") {galafold ="Responsive";}
else if (sostituzione == "A37V") {galafold ="Responsive";}
else if (sostituzione == "A73E") {galafold ="Non Responsive";}
else if (sostituzione == "A73V") {galafold ="Responsive";}
else if (sostituzione == "A97P") {galafold ="Responsive";}
else if (sostituzione == "A97V") {galafold ="Responsive";}
else if (sostituzione == "C142R") {galafold ="Non Responsive";}
else if (sostituzione == "C142W") {galafold ="Non Responsive";}
else if (sostituzione == "C172G") {galafold ="Non Responsive";}
else if (sostituzione == "C172Y") {galafold ="Non Responsive";}
else if (sostituzione == "C174G") {galafold ="Responsive";}
else if (sostituzione == "C174R") {galafold ="Responsive";}
else if (sostituzione == "C202Y") {galafold ="Non Responsive";}
else if (sostituzione == "C378R") {galafold ="Non Responsive";}
else if (sostituzione == "C52G") {galafold ="Non Responsive";}
else if (sostituzione == "C52W") {galafold ="Non Responsive";}
else if (sostituzione == "C52Y") {galafold ="Non Responsive";}
else if (sostituzione == "C56F") {galafold ="Responsive";}
else if (sostituzione == "C56Y") {galafold ="Responsive";}
else if (sostituzione == "C63Y") {galafold ="Non Responsive";}
else if (sostituzione == "C94S") {galafold ="Non Responsive";}
else if (sostituzione == "C94Y") {galafold ="Non Responsive";}
else if (sostituzione == "D109G") {galafold ="Responsive";}
else if (sostituzione == "D136E") {galafold ="Responsive";}
else if (sostituzione == "D136H") {galafold ="Non Responsive";}
else if (sostituzione == "D155H") {galafold ="Non Responsive";}
else if (sostituzione == "D165G") {galafold ="Responsive";}
else if (sostituzione == "D165H") {galafold ="Responsive";}
else if (sostituzione == "D165V") {galafold ="Non Responsive";}
else if (sostituzione == "D165Y") {galafold ="Non Responsive";}
else if (sostituzione == "D170N") {galafold ="Non Responsive";}
else if (sostituzione == "D170V") {galafold ="Non Responsive";}
else if (sostituzione == "D175E") {galafold ="Responsive";}
else if (sostituzione == "D175N") {galafold ="Not Reported";}
else if (sostituzione == "D231G") {galafold ="Non Responsive";}
else if (sostituzione == "D231N") {galafold ="Non Responsive";}
else if (sostituzione == "D231V") {galafold ="Non Responsive";}
else if (sostituzione == "D234E") {galafold ="Non Responsive";}
else if (sostituzione == "D244H") {galafold ="Responsive";}
else if (sostituzione == "D244N") {galafold ="Responsive";}
else if (sostituzione == "D264A") {galafold ="Non Responsive";}
else if (sostituzione == "D264E") {galafold ="Not Reported";}
else if (sostituzione == "D264G") {galafold ="Not Reported";}
else if (sostituzione == "D264H") {galafold ="Not Reported";}
else if (sostituzione == "D264N") {galafold ="Not Reported";}
else if (sostituzione == "D264V") {galafold ="Non Responsive";}
else if (sostituzione == "D264Y") {galafold ="Responsive";}
else if (sostituzione == "D266N") {galafold ="Non Responsive";}
else if (sostituzione == "D266V") {galafold ="Non Responsive";}
else if (sostituzione == "D299E") {galafold ="Responsive";}
else if (sostituzione == "D313G") {galafold ="Responsive";}
else if (sostituzione == "D313N") {galafold ="Not Reported";}
else if (sostituzione == "D313Y") {galafold ="Responsive";}
else if (sostituzione == "D315N") {galafold ="Not Reported";}
else if (sostituzione == "D322E") {galafold ="Responsive";}
else if (sostituzione == "D322N") {galafold ="Responsive";}
else if (sostituzione == "D33G") {galafold ="Responsive";}
else if (sostituzione == "D33Y") {galafold ="Responsive";}
else if (sostituzione == "D55V") {galafold ="Responsive";}
else if (sostituzione == "D83N") {galafold ="Responsive";}
else if (sostituzione == "D92N") {galafold ="Non Responsive";}
else if (sostituzione == "D92Y") {galafold ="Non Responsive";}
else if (sostituzione == "D93E") {galafold ="Non Responsive";}
else if (sostituzione == "D93V") {galafold ="Non Responsive";}
else if (sostituzione == "D93Y") {galafold ="Non Responsive";}
else if (sostituzione == "E103Q") {galafold ="Non Responsive";}
else if (sostituzione == "E203D") {galafold ="Responsive";}
else if (sostituzione == "E203V") {galafold ="Responsive";}
else if (sostituzione == "E338K") {galafold ="Responsive";}
else if (sostituzione == "E341D") {galafold ="Non Responsive";}
else if (sostituzione == "E341K") {galafold ="Non Responsive";}
else if (sostituzione == "E358A") {galafold ="Responsive";}
else if (sostituzione == "E358D") {galafold ="Responsive";}
else if (sostituzione == "E358G") {galafold ="Responsive";}
else if (sostituzione == "E358K") {galafold ="Non Responsive";}
else if (sostituzione == "E398A") {galafold ="Not Reported";}
else if (sostituzione == "E398K") {galafold ="Responsive";}
else if (sostituzione == "E418G") {galafold ="Responsive";}
else if (sostituzione == "E48D") {galafold ="Non Responsive";}
else if (sostituzione == "E48K") {galafold ="Non Responsive";}
else if (sostituzione == "E48Q") {galafold ="Responsive";}
else if (sostituzione == "E59K") {galafold ="Responsive";}
else if (sostituzione == "E66D") {galafold ="Not Reported";}
else if (sostituzione == "E66G") {galafold ="Responsive";}
else if (sostituzione == "E66K") {galafold ="Responsive";}
else if (sostituzione == "E66Q") {galafold ="Non Responsive";}
else if (sostituzione == "E71G") {galafold ="Not Reported";}
else if (sostituzione == "F113I") {galafold ="Not Reported";}
else if (sostituzione == "F113L") {galafold ="Responsive";}
else if (sostituzione == "F113S") {galafold ="Non Responsive";}
else if (sostituzione == "F169S") {galafold ="Responsive";}
else if (sostituzione == "F18S") {galafold ="Non Responsive";}
else if (sostituzione == "F229L") {galafold ="Responsive";}
else if (sostituzione == "F273L") {galafold ="Non Responsive";}
else if (sostituzione == "F295C") {galafold ="Responsive";}
else if (sostituzione == "F396Y") {galafold ="Non Responsive";}
else if (sostituzione == "F50C") {galafold ="Non Responsive";}
else if (sostituzione == "G104V") {galafold ="Responsive";}
else if (sostituzione == "G128E") {galafold ="Responsive";}
else if (sostituzione == "G132A") {galafold ="Non Responsive";}
else if (sostituzione == "G132E") {galafold ="Non Responsive";}
else if (sostituzione == "G132R") {galafold ="Non Responsive";}
else if (sostituzione == "G138E") {galafold ="Non Responsive";}
else if (sostituzione == "G138R") {galafold ="Non Responsive";}
else if (sostituzione == "G144D") {galafold ="Responsive";}
else if (sostituzione == "G144V") {galafold ="Responsive";}
else if (sostituzione == "G147R") {galafold ="Non Responsive";}
else if (sostituzione == "G163V") {galafold ="Non Responsive";}
else if (sostituzione == "G171D") {galafold ="Non Responsive";}
else if (sostituzione == "G171R") {galafold ="Non Responsive";}
else if (sostituzione == "G183A") {galafold ="Responsive";}
else if (sostituzione == "G183D") {galafold ="Responsive";}
else if (sostituzione == "G183S") {galafold ="Not Reported";}
else if (sostituzione == "G183V") {galafold ="Non Responsive";}
else if (sostituzione == "G195V") {galafold ="Responsive";}
else if (sostituzione == "G258R") {galafold ="Responsive";}
else if (sostituzione == "G258V") {galafold ="Responsive";}
else if (sostituzione == "G260A") {galafold ="Responsive";}
else if (sostituzione == "G260E") {galafold ="Responsive";}
else if (sostituzione == "G261D") {galafold ="Non Responsive";}
else if (sostituzione == "G261V") {galafold ="Non Responsive";}
else if (sostituzione == "G271C") {galafold ="Non Responsive";}
else if (sostituzione == "G271D") {galafold ="Responsive";}
else if (sostituzione == "G271S") {galafold ="Responsive";}
else if (sostituzione == "G271V") {galafold ="Non Responsive";}
else if (sostituzione == "G325D") {galafold ="Non Responsive";}
else if (sostituzione == "G325R") {galafold ="Responsive";}
else if (sostituzione == "G325S") {galafold ="Responsive";}
else if (sostituzione == "G328A") {galafold ="Responsive";}
else if (sostituzione == "G328R") {galafold ="Non Responsive";}
else if (sostituzione == "G334E") {galafold ="Responsive";}
else if (sostituzione == "G35E") {galafold ="Not Reported";}
else if (sostituzione == "G35R") {galafold ="Responsive";}
else if (sostituzione == "G360C") {galafold ="Responsive";}
else if (sostituzione == "G360D") {galafold ="Responsive";}
else if (sostituzione == "G360S") {galafold ="Responsive";}
else if (sostituzione == "G361A") {galafold ="Responsive";}
else if (sostituzione == "G361E") {galafold ="Responsive";}
else if (sostituzione == "G361R") {galafold ="Non Responsive";}
else if (sostituzione == "G373D") {galafold ="Non Responsive";}
else if (sostituzione == "G373S") {galafold ="Responsive";}
else if (sostituzione == "G375A") {galafold ="Not Reported";}
else if (sostituzione == "G395A") {galafold ="Responsive";}
else if (sostituzione == "G395E") {galafold ="Responsive";}
else if (sostituzione == "G411D") {galafold ="Responsive";}
else if (sostituzione == "G43S") {galafold ="Non Responsive";}
else if (sostituzione == "G80D") {galafold ="Not Reported";}
else if (sostituzione == "G85D") {galafold ="Responsive";}
else if (sostituzione == "G85M") {galafold ="Responsive";}
else if (sostituzione == "G85S") {galafold ="Responsive";}
else if (sostituzione == "H125L") {galafold ="Responsive";}
else if (sostituzione == "H125P") {galafold ="Non Responsive";}
else if (sostituzione == "H225D") {galafold ="Responsive";}
else if (sostituzione == "H225R") {galafold ="Non Responsive";}
else if (sostituzione == "H406R") {galafold ="Non Responsive";}
else if (sostituzione == "H46P") {galafold ="Responsive";}
else if (sostituzione == "H46R") {galafold ="Non Responsive";}
else if (sostituzione == "H46Y") {galafold ="Non Responsive";}
else if (sostituzione == "I117S") {galafold ="Non Responsive";}
else if (sostituzione == "I154T") {galafold ="Not Reported";}
else if (sostituzione == "I198T") {galafold ="Responsive";}
else if (sostituzione == "I219M") {galafold ="Not Reported";}
else if (sostituzione == "I219N") {galafold ="Responsive";}
else if (sostituzione == "I219T") {galafold ="Responsive";}
else if (sostituzione == "I232T") {galafold ="Responsive";}
else if (sostituzione == "I239T") {galafold ="Responsive";}
else if (sostituzione == "I242F") {galafold ="Responsive";}
else if (sostituzione == "I242N") {galafold ="Responsive";}
else if (sostituzione == "I242V") {galafold ="Not Reported";}
else if (sostituzione == "I253S") {galafold ="Responsive";}
else if (sostituzione == "I253T") {galafold ="Responsive";}
else if (sostituzione == "I270M") {galafold ="Not Reported";}
else if (sostituzione == "I270T") {galafold ="Responsive";}
else if (sostituzione == "I289F") {galafold ="Non Responsive";}
else if (sostituzione == "I289S") {galafold ="Responsive";}
else if (sostituzione == "I289V") {galafold ="Not Reported";}
else if (sostituzione == "I303N") {galafold ="Responsive";}
else if (sostituzione == "I317N") {galafold ="Non Responsive";}
else if (sostituzione == "I317S") {galafold ="Non Responsive";}
else if (sostituzione == "I317T") {galafold ="Responsive";}
else if (sostituzione == "I319F") {galafold ="Responsive";}
else if (sostituzione == "I319T") {galafold ="Responsive";}
else if (sostituzione == "I354K") {galafold ="Responsive";}
else if (sostituzione == "I359T") {galafold ="Responsive";}
else if (sostituzione == "I384N") {galafold ="Non Responsive";}
else if (sostituzione == "I407V") {galafold ="Non Responsive";}
else if (sostituzione == "I64F") {galafold ="Not Reported";}
else if (sostituzione == "I91N") {galafold ="Not Reported";}
else if (sostituzione == "I91T") {galafold ="Responsive";}
else if (sostituzione == "K130R") {galafold ="Non Responsive";}
else if (sostituzione == "K140T") {galafold ="Responsive";}
else if (sostituzione == "K185E") {galafold ="Responsive";}
else if (sostituzione == "K213M") {galafold ="Responsive";}
else if (sostituzione == "K213R") {galafold ="Not Reported";}
else if (sostituzione == "K308N") {galafold ="Responsive";}
else if (sostituzione == "K391T") {galafold ="Responsive";}
else if (sostituzione == "L120P") {galafold ="Non Responsive";}
else if (sostituzione == "L120S") {galafold ="Non Responsive";}
else if (sostituzione == "L120V") {galafold ="Non Responsive";}
else if (sostituzione == "L129P") {galafold ="Non Responsive";}
else if (sostituzione == "L131P") {galafold ="Non Responsive";}
else if (sostituzione == "L14P") {galafold ="Non Responsive";}
else if (sostituzione == "L166G") {galafold ="Responsive";}
else if (sostituzione == "L166V") {galafold ="Responsive";}
else if (sostituzione == "L167Q") {galafold ="Non Responsive";}
else if (sostituzione == "L180F") {galafold ="Not Reported";}
else if (sostituzione == "L191P") {galafold ="Non Responsive";}
else if (sostituzione == "L191Q") {galafold ="Responsive";}
else if (sostituzione == "L206P") {galafold ="Non Responsive";}
else if (sostituzione == "L21F") {galafold ="Non Responsive";}
else if (sostituzione == "L21P") {galafold ="Non Responsive";}
else if (sostituzione == "L21R") {galafold ="Non Responsive";}
else if (sostituzione == "L243F") {galafold ="Responsive";}
else if (sostituzione == "L243W") {galafold ="Responsive";}
else if (sostituzione == "L268S") {galafold ="Non Responsive";}
else if (sostituzione == "L275F") {galafold ="Non Responsive";}
else if (sostituzione == "L275H") {galafold ="Not Reported";}
else if (sostituzione == "L294S") {galafold ="Responsive";}
else if (sostituzione == "L300F") {galafold ="Responsive";}
else if (sostituzione == "L300P") {galafold ="Responsive";}
else if (sostituzione == "L310F") {galafold ="Responsive";}
else if (sostituzione == "L310R") {galafold ="Non Responsive";}
else if (sostituzione == "L311F") {galafold ="Non Responsive";}
else if (sostituzione == "L311V") {galafold ="Responsive";}
else if (sostituzione == "L32P") {galafold ="Responsive";}
else if (sostituzione == "L344P") {galafold ="Non Responsive";}
else if (sostituzione == "L36F") {galafold ="Responsive";}
else if (sostituzione == "L36S") {galafold ="Responsive";}
else if (sostituzione == "L36W") {galafold ="Responsive";}
else if (sostituzione == "L372P") {galafold ="Non Responsive";}
else if (sostituzione == "L372Q") {galafold ="Non Responsive";}
else if (sostituzione == "L372R") {galafold ="Non Responsive";}
else if (sostituzione == "L3P") {galafold ="Responsive";}
else if (sostituzione == "L3V") {galafold ="Not Reported";}
else if (sostituzione == "L403S") {galafold ="Responsive";}
else if (sostituzione == "L414S") {galafold ="Non Responsive";}
else if (sostituzione == "L415F") {galafold ="Non Responsive";}
else if (sostituzione == "L415P") {galafold ="Non Responsive";}
else if (sostituzione == "L45P") {galafold ="Non Responsive";}
else if (sostituzione == "L45R") {galafold ="Non Responsive";}
else if (sostituzione == "L54F") {galafold ="Responsive";}
else if (sostituzione == "L54P") {galafold ="Responsive";}
else if (sostituzione == "L68F") {galafold ="Non Responsive";}
else if (sostituzione == "L89F") {galafold ="Responsive";}
else if (sostituzione == "L89H") {galafold ="Not Reported";}
else if (sostituzione == "L89R") {galafold ="Non Responsive";}
else if (sostituzione == "M187I") {galafold ="Responsive";}
else if (sostituzione == "M187T") {galafold ="Responsive";}
else if (sostituzione == "M187V") {galafold ="Responsive";}
else if (sostituzione == "M1I") {galafold ="Non Responsive";}
else if (sostituzione == "M1L") {galafold ="Non Responsive";}
else if (sostituzione == "M1R") {galafold ="Non Responsive";}
else if (sostituzione == "M1T") {galafold ="Non Responsive";}
else if (sostituzione == "M1V") {galafold ="Non Responsive";}
else if (sostituzione == "M267T") {galafold ="Responsive";}
else if (sostituzione == "M284T") {galafold ="Responsive";}
else if (sostituzione == "M284V") {galafold ="Responsive";}
else if (sostituzione == "M290I") {galafold ="Responsive";}
else if (sostituzione == "M290L") {galafold ="Responsive";}
else if (sostituzione == "M296I") {galafold ="Responsive";}
else if (sostituzione == "M296L") {galafold ="Responsive";}
else if (sostituzione == "M296T") {galafold ="Responsive";}
else if (sostituzione == "M296V") {galafold ="Responsive";}
else if (sostituzione == "M421V") {galafold ="Responsive";}
else if (sostituzione == "M42K") {galafold ="Responsive";}
else if (sostituzione == "M42L") {galafold ="Responsive";}
else if (sostituzione == "M42R") {galafold ="Responsive";}
else if (sostituzione == "M42T") {galafold ="Responsive";}
else if (sostituzione == "M42V") {galafold ="Responsive";}
else if (sostituzione == "M51I") {galafold ="Responsive";}
else if (sostituzione == "M51K") {galafold ="Responsive";}
else if (sostituzione == "M72I") {galafold ="Responsive";}
else if (sostituzione == "M72V") {galafold ="Responsive";}
else if (sostituzione == "M76T") {galafold ="Responsive";}
else if (sostituzione == "M96I") {galafold ="Responsive";}
else if (sostituzione == "N139S") {galafold ="Responsive";}
else if (sostituzione == "N139T") {galafold ="Non Responsive";}
else if (sostituzione == "N215D") {galafold ="Responsive";}
else if (sostituzione == "N215S") {galafold ="Responsive";}
else if (sostituzione == "N224D") {galafold ="Non Responsive";}
else if (sostituzione == "N224S") {galafold ="Responsive";}
else if (sostituzione == "N228S") {galafold ="Responsive";}
else if (sostituzione == "N249K") {galafold ="Responsive";}
else if (sostituzione == "N263S") {galafold ="Responsive";}
else if (sostituzione == "N272K") {galafold ="Non Responsive";}
else if (sostituzione == "N272S") {galafold ="Non Responsive";}
else if (sostituzione == "N298S") {galafold ="Responsive";}
else if (sostituzione == "N320I") {galafold ="Responsive";}
else if (sostituzione == "N320Y") {galafold ="Non Responsive";}
else if (sostituzione == "N34K") {galafold ="Responsive";}
else if (sostituzione == "N34S") {galafold ="Responsive";}
else if (sostituzione == "N53D") {galafold ="Responsive";}
else if (sostituzione == "N53L") {galafold ="Responsive";}
else if (sostituzione == "P146S") {galafold ="Responsive";}
else if (sostituzione == "P205L") {galafold ="Responsive";}
else if (sostituzione == "P205R") {galafold ="Non Responsive";}
else if (sostituzione == "P205S") {galafold ="Responsive";}
else if (sostituzione == "P205T") {galafold ="Responsive";}
else if (sostituzione == "P210L") {galafold ="Responsive";}
else if (sostituzione == "P210S") {galafold ="Responsive";}
else if (sostituzione == "P214L") {galafold ="Responsive";}
else if (sostituzione == "P214S") {galafold ="Responsive";}
else if (sostituzione == "P259L") {galafold ="Responsive";}
else if (sostituzione == "P259R") {galafold ="Responsive";}
else if (sostituzione == "P265L") {galafold ="Responsive";}
else if (sostituzione == "P265R") {galafold ="Non Responsive";}
else if (sostituzione == "P265S") {galafold ="Non Responsive";}
else if (sostituzione == "P293A") {galafold ="Non Responsive";}
else if (sostituzione == "P293S") {galafold ="Non Responsive";}
else if (sostituzione == "P293T") {galafold ="Responsive";}
else if (sostituzione == "P323R") {galafold ="Not Reported";}
else if (sostituzione == "P343L") {galafold ="Responsive";}
else if (sostituzione == "P362L") {galafold ="Responsive";}
else if (sostituzione == "P362T") {galafold ="Responsive";}
else if (sostituzione == "P389A") {galafold ="Non Responsive";}
else if (sostituzione == "P389L") {galafold ="Non Responsive";}
else if (sostituzione == "P409A") {galafold ="Responsive";}
else if (sostituzione == "P409S") {galafold ="Responsive";}
else if (sostituzione == "P409T") {galafold ="Responsive";}
else if (sostituzione == "P40A") {galafold ="Non Responsive";}
else if (sostituzione == "P40S") {galafold ="Non Responsive";}
else if (sostituzione == "P60L") {galafold ="Responsive";}
else if (sostituzione == "P60S") {galafold ="Responsive";}
else if (sostituzione == "P60T") {galafold ="Responsive";}
else if (sostituzione == "Q107L") {galafold ="Non Responsive";}
else if (sostituzione == "Q250P") {galafold ="Responsive";}
else if (sostituzione == "Q279E") {galafold ="Responsive";}
else if (sostituzione == "Q279H") {galafold ="Non Responsive";}
else if (sostituzione == "Q280H") {galafold ="Responsive";}
else if (sostituzione == "Q280K") {galafold ="Responsive";}
else if (sostituzione == "Q283P") {galafold ="Non Responsive";}
else if (sostituzione == "Q312H") {galafold ="Responsive";}
else if (sostituzione == "Q312R") {galafold ="Responsive";}
else if (sostituzione == "Q321E") {galafold ="Non Responsive";}
else if (sostituzione == "Q321H") {galafold ="Responsive";}
else if (sostituzione == "Q321L") {galafold ="Responsive";}
else if (sostituzione == "Q321R") {galafold ="Responsive";}
else if (sostituzione == "Q327E") {galafold ="Responsive";}
else if (sostituzione == "Q327K") {galafold ="Non Responsive";}
else if (sostituzione == "Q327L") {galafold ="Not Reported";}
else if (sostituzione == "Q327R") {galafold ="Not Reported";}
else if (sostituzione == "Q330R") {galafold ="Not Reported";}
else if (sostituzione == "Q333R ") {galafold ="Non Responsive";}
else if (sostituzione == "Q386P") {galafold ="Non Responsive";}
else if (sostituzione == "Q416P") {galafold ="Not Reported";}
else if (sostituzione == "Q57L") {galafold ="Responsive";}
else if (sostituzione == "R100K") {galafold ="Non Responsive";}
else if (sostituzione == "R100T") {galafold ="Non Responsive";}
else if (sostituzione == "R112C") {galafold ="Non Responsive";}
else if (sostituzione == "R112G") {galafold ="Responsive";}
else if (sostituzione == "R112H") {galafold ="Responsive";}
else if (sostituzione == "R112S") {galafold ="Non Responsive";}
else if (sostituzione == "R118C") {galafold ="Responsive";}
else if (sostituzione == "R118G") {galafold ="Not Reported";}
else if (sostituzione == "R118H") {galafold ="Not Reported";}
else if (sostituzione == "R118L") {galafold ="Not Reported";}
else if (sostituzione == "R118P") {galafold ="Not Reported";}
else if (sostituzione == "R118S") {galafold ="Not Reported";}
else if (sostituzione == "R196S") {galafold ="Not Reported";}
else if (sostituzione == "R220P") {galafold ="Responsive";}
else if (sostituzione == "R220Q") {galafold ="Responsive";}
else if (sostituzione == "R227P") {galafold ="Non Responsive";}
else if (sostituzione == "R227Q") {galafold ="Non Responsive";}
else if (sostituzione == "R252T") {galafold ="Non Responsive";}
else if (sostituzione == "R301G") {galafold ="Responsive";}
else if (sostituzione == "R301L") {galafold ="Responsive";}
else if (sostituzione == "R301P") {galafold ="Responsive";}
else if (sostituzione == "R301Q") {galafold ="Responsive";}
else if (sostituzione == "R342L") {galafold ="Non Responsive";}
else if (sostituzione == "R342P") {galafold ="Non Responsive";}
else if (sostituzione == "R342Q") {galafold ="Non Responsive";}
else if (sostituzione == "R356G") {galafold ="Responsive";}
else if (sostituzione == "R356P") {galafold ="Not Reported";}
else if (sostituzione == "R356Q") {galafold ="Responsive";}
else if (sostituzione == "R356W") {galafold ="Responsive";}
else if (sostituzione == "R363C") {galafold ="Responsive";}
else if (sostituzione == "R363H") {galafold ="Responsive";}
else if (sostituzione == "R363P") {galafold ="Non Responsive";}
else if (sostituzione == "R392S") {galafold ="Not Reported";}
else if (sostituzione == "R49C") {galafold ="Non Responsive";}
else if (sostituzione == "R49G") {galafold ="Non Responsive";}
else if (sostituzione == "R49L") {galafold ="Non Responsive";}
else if (sostituzione == "S102L") {galafold ="Responsive";}
else if (sostituzione == "S126C") {galafold ="Not Reported";}
else if (sostituzione == "S126G") {galafold ="Responsive";}
else if (sostituzione == "S126I") {galafold ="Not Reported";}
else if (sostituzione == "S126N") {galafold ="Not Reported";}
else if (sostituzione == "S126R") {galafold ="Not Reported";}
else if (sostituzione == "S126T") {galafold ="Not Reported";}
else if (sostituzione == "S148N") {galafold ="Non Responsive";}
else if (sostituzione == "S148R") {galafold ="Non Responsive";}
else if (sostituzione == "S201F") {galafold ="Responsive";}
else if (sostituzione == "S201Y") {galafold ="Responsive";}
else if (sostituzione == "S235C") {galafold ="Non Responsive";}
else if (sostituzione == "S235F") {galafold ="Non Responsive";}
else if (sostituzione == "S238N") {galafold ="Responsive";}
else if (sostituzione == "S238R") {galafold ="Non Responsive";}
else if (sostituzione == "S247C") {galafold ="Responsive";}
else if (sostituzione == "S247P") {galafold ="Non Responsive";}
else if (sostituzione == "S276G") {galafold ="Non Responsive";}
else if (sostituzione == "S276N") {galafold ="Responsive";}
else if (sostituzione == "S297C") {galafold ="Non Responsive";}
else if (sostituzione == "S297F") {galafold ="Non Responsive";}
else if (sostituzione == "S304N") {galafold ="Responsive";}
else if (sostituzione == "S304T") {galafold ="Responsive";}
else if (sostituzione == "S345P") {galafold ="Responsive";}
else if (sostituzione == "S405R") {galafold ="Non Responsive";}
else if (sostituzione == "S65A") {galafold ="Not Reported";}
else if (sostituzione == "S65I") {galafold ="Not Reported";}
else if (sostituzione == "S65T") {galafold ="Not Reported";}
else if (sostituzione == "T141I") {galafold ="Non Responsive";}
else if (sostituzione == "T194I") {galafold ="Responsive";}
else if (sostituzione == "T282A") {galafold ="Responsive";}
else if (sostituzione == "T282I") {galafold ="Responsive";}
else if (sostituzione == "T385A") {galafold ="Responsive";}
else if (sostituzione == "T410A") {galafold ="Responsive";}
else if (sostituzione == "T410I") {galafold ="Responsive";}
else if (sostituzione == "T410P") {galafold ="Non Responsive";}
else if (sostituzione == "T412N") {galafold ="Responsive";}
else if (sostituzione == "T41I") {galafold ="Responsive";}
else if (sostituzione == "V164G") {galafold ="Non Responsive";}
else if (sostituzione == "V164L") {galafold ="Not Reported";}
else if (sostituzione == "V199G") {galafold ="Responsive";}
else if (sostituzione == "V199M") {galafold ="Responsive";}
else if (sostituzione == "V254A") {galafold ="Not Reported";}
else if (sostituzione == "V269A") {galafold ="Responsive";}
else if (sostituzione == "V269G") {galafold ="Not Reported";}
else if (sostituzione == "V269M") {galafold ="Responsive";}
else if (sostituzione == "V316A") {galafold ="Not Reported";}
else if (sostituzione == "V316E") {galafold ="Non Responsive";}
else if (sostituzione == "V316G") {galafold ="Responsive";}
else if (sostituzione == "V316I") {galafold ="Responsive";}
else if (sostituzione == "V339E") {galafold ="Responsive";}
else if (sostituzione == "V339G") {galafold ="Non Responsive";}
else if (sostituzione == "V390M") {galafold ="Not Reported";}
else if (sostituzione == "W162C") {galafold ="Non Responsive";}
else if (sostituzione == "W162G") {galafold ="Responsive";}
else if (sostituzione == "W162R") {galafold ="Non Responsive";}
else if (sostituzione == "W204C") {galafold ="Non Responsive";}
else if (sostituzione == "W204R") {galafold ="Not Reported";}
else if (sostituzione == "W226R") {galafold ="Non Responsive";}
else if (sostituzione == "W236C") {galafold ="Non Responsive";}
else if (sostituzione == "W236R") {galafold ="Non Responsive";}
else if (sostituzione == "W245G") {galafold ="Responsive";}
else if (sostituzione == "W24C") {galafold ="Responsive";}
else if (sostituzione == "W24G") {galafold ="Responsive";}
else if (sostituzione == "W24R") {galafold ="Responsive";}
else if (sostituzione == "W262C") {galafold ="Non Responsive";}
else if (sostituzione == "W262L") {galafold ="Non Responsive";}
else if (sostituzione == "W262R") {galafold ="Not Reported";}
else if (sostituzione == "W277C") {galafold ="Responsive";}
else if (sostituzione == "W277G") {galafold ="Responsive";}
else if (sostituzione == "W287C") {galafold ="Non Responsive";}
else if (sostituzione == "W349R") {galafold ="Non Responsive";}
else if (sostituzione == "W349S") {galafold ="Responsive";}
else if (sostituzione == "W399S") {galafold ="Not Reported";}
else if (sostituzione == "W81C") {galafold ="Non Responsive";}
else if (sostituzione == "W81S") {galafold ="Non Responsive";}
else if (sostituzione == "W95C") {galafold ="Not Reported";}
else if (sostituzione == "W95L") {galafold ="Non Responsive";}
else if (sostituzione == "W95S") {galafold ="Non Responsive";}
else if (sostituzione == "Y123C") {galafold ="Responsive";}
else if (sostituzione == "Y152C") {galafold ="Responsive";}
else if (sostituzione == "Y184C") {galafold ="Responsive";}
else if (sostituzione == "Y184N") {galafold ="Responsive";}
else if (sostituzione == "Y200C") {galafold ="Responsive";}
else if (sostituzione == "Y207C") {galafold ="Non Responsive";}
else if (sostituzione == "Y207H") {galafold ="Responsive";}
else if (sostituzione == "Y207S") {galafold ="Responsive";}
else if (sostituzione == "Y216C") {galafold ="Responsive";}
else if (sostituzione == "Y216D") {galafold ="Responsive";}
else if (sostituzione == "Y86D") {galafold ="Non Responsive";}
else if (sostituzione == "Y86H") {galafold ="Non Responsive";}
else if (sostituzione == "Y88D") {galafold ="Non Responsive";}

else {galafold = "Not Reported";}


var ponti_disolfuro;
if (posizione_amminoacidica == "52") {ponti_disolfuro = "Yes";}
else if (posizione_amminoacidica == "94") {ponti_disolfuro = "Yes";}
else if (posizione_amminoacidica == "56") {ponti_disolfuro = "Yes";}
else if (posizione_amminoacidica == "63") {ponti_disolfuro = "Yes";}
else if (posizione_amminoacidica == "142") {ponti_disolfuro = "Yes";}
else if (posizione_amminoacidica == "172") {ponti_disolfuro = "Yes";}
else if (posizione_amminoacidica == "202") {ponti_disolfuro = "Yes";}
else if (posizione_amminoacidica == "223") {ponti_disolfuro = "Yes";}
else if (posizione_amminoacidica == "378") {ponti_disolfuro = "Yes";}
else if (posizione_amminoacidica == "382") {ponti_disolfuro = "Yes";}
else {ponti_disolfuro = "No";}


var sito_attivo;

if (posizione_amminoacidica == "47") {sito_attivo = "Yes";}
else if (posizione_amminoacidica == "92") {sito_attivo = "Yes";}
else if (posizione_amminoacidica == "93") {sito_attivo = "Yes";}
else if (posizione_amminoacidica == "134") {sito_attivo = "Yes";}
else if (posizione_amminoacidica == "142") {sito_attivo = "Yes";}
else if (posizione_amminoacidica == "168") {sito_attivo = "Yes";}
else if (posizione_amminoacidica == "170") {sito_attivo = "Yes";}
else if (posizione_amminoacidica == "203") {sito_attivo = "Yes";}
else if (posizione_amminoacidica == "206") {sito_attivo = "Yes";}
else if (posizione_amminoacidica == "207") {sito_attivo = "Yes";}
else if (posizione_amminoacidica == "227") {sito_attivo = "Yes";}
else if (posizione_amminoacidica == "230") {sito_attivo = "Yes";}
else if (posizione_amminoacidica == "231") {sito_attivo = "Yes";}
else if (posizione_amminoacidica == "267") {sito_attivo = "Yes";}
else {sito_attivo = "No";}


var valore_pssm;
if (sostituzione == "N336H") {valore_pssm = "2";}
 if (sostituzione == "S197P") {valore_pssm = "2";}
 if (sostituzione == "L166Y") {valore_pssm = "2";}
 if (sostituzione == "D182E") {valore_pssm = "2";}
 if (sostituzione == "D313N") {valore_pssm = "2";}
 if (sostituzione == "S364H") {valore_pssm = "2";}
 if (sostituzione == "N249H") {valore_pssm = "2";}
 if (sostituzione == "N298C") {valore_pssm = "2";}
 if (sostituzione == "T41P") {valore_pssm = "2";}
 if (sostituzione == "I354F") {valore_pssm = "2";}
 if (sostituzione == "N278E") {valore_pssm = "2";}
 if (sostituzione == "D255P") {valore_pssm = "2";}
 if (sostituzione == "G346N") {valore_pssm = "2";}
 if (sostituzione == "Q283H") {valore_pssm = "2";}
 if (sostituzione == "Y151H") {valore_pssm = "2";}
 if (sostituzione == "L75R") {valore_pssm = "2";}
 if (sostituzione == "Y152E") {valore_pssm = "2";}
 if (sostituzione == "A230G") {valore_pssm = "2";}
 if (sostituzione == "W277F") {valore_pssm = "1";}
 if (sostituzione == "Q119F") {valore_pssm = "1";}
 if (sostituzione == "M353F") {valore_pssm = "1";}
 if (sostituzione == "L189Q") {valore_pssm = "1";}
 if (sostituzione == "L347G") {valore_pssm = "1";}
 if (sostituzione == "D315E") {valore_pssm = "1";}
 if (sostituzione == "K393H") {valore_pssm = "1";}
 if (sostituzione == "P27H") {valore_pssm = "1";}
 if (sostituzione == "N122D") {valore_pssm = "1";}
 if (sostituzione == "V199F") {valore_pssm = "1";}
 if (sostituzione == "R118K") {valore_pssm = "1";}
 if (sostituzione == "Q119Y") {valore_pssm = "1";}
 if (sostituzione == "K240M") {valore_pssm = "1";}
 if (sostituzione == "N278D") {valore_pssm = "1";}
 if (sostituzione == "T41Q") {valore_pssm = "1";}
 if (sostituzione == "L310I") {valore_pssm = "1";}
 if (sostituzione == "L166M") {valore_pssm = "1";}
 if (sostituzione == "L415F") {valore_pssm = "1";}
 if (sostituzione == "L75A") {valore_pssm = "1";}
 if (sostituzione == "D182Q") {valore_pssm = "1";}
 if (sostituzione == "Q280Y") {valore_pssm = "1";}
 if (sostituzione == "L347N") {valore_pssm = "1";}
 if (sostituzione == "T246F") {valore_pssm = "1";}
 if (sostituzione == "S23F") {valore_pssm = "1";}
 if (sostituzione == "D153E") {valore_pssm = "1";}
 if (sostituzione == "L331V") {valore_pssm = "1";}
 if (sostituzione == "Y222I") {valore_pssm = "1";}
 if (sostituzione == "Q330H") {valore_pssm = "1";}
 if (sostituzione == "M353V") {valore_pssm = "1";}
 if (sostituzione == "Q119H") {valore_pssm = "1";}
 if (sostituzione == "E251D") {valore_pssm = "1";}
 if (sostituzione == "A108P") {valore_pssm = "1";}
 if (sostituzione == "R17C") {valore_pssm = "1";}
 if (sostituzione == "L89I") {valore_pssm = "1";}
 if (sostituzione == "L177K") {valore_pssm = "1";}
 if (sostituzione == "D255R") {valore_pssm = "1";}
 if (sostituzione == "S247V") {valore_pssm = "1";}
 if (sostituzione == "R363C") {valore_pssm = "1";}
 if (sostituzione == "S364P") {valore_pssm = "1";}
 if (sostituzione == "L206A") {valore_pssm = "1";}
 if (sostituzione == "K82R") {valore_pssm = "1";}
 if (sostituzione == "L189K") {valore_pssm = "1";}
 if (sostituzione == "M208E") {valore_pssm = "1";}
 if (sostituzione == "Q221R") {valore_pssm = "1";}
 if (sostituzione == "A230D") {valore_pssm = "1";}
 if (sostituzione == "Q330R") {valore_pssm = "1";}
 if (sostituzione == "M284F") {valore_pssm = "1";}
 if (sostituzione == "M208F") {valore_pssm = "1";}
 if (sostituzione == "K326V") {valore_pssm = "1";}
 if (sostituzione == "I154Q") {valore_pssm = "1";}
 if (sostituzione == "P210G") {valore_pssm = "1";}
 if (sostituzione == "Y222H") {valore_pssm = "1";}
 if (sostituzione == "L189D") {valore_pssm = "1";}
 if (sostituzione == "V281R") {valore_pssm = "1";}
 if (sostituzione == "S364R") {valore_pssm = "1";}
 if (sostituzione == "P98H") {valore_pssm = "1";}
 if (sostituzione == "G261H") {valore_pssm = "1";}
 if (sostituzione == "L347K") {valore_pssm = "1";}
 if (sostituzione == "L177P") {valore_pssm = "1";}
 if (sostituzione == "R332K") {valore_pssm = "1";}
 if (sostituzione == "D61K") {valore_pssm = "1";}
 if (sostituzione == "A307T") {valore_pssm = "1";}
 if (sostituzione == "A135E") {valore_pssm = "1";}
 if (sostituzione == "G183R") {valore_pssm = "1";}
 if (sostituzione == "L275M") {valore_pssm = "1";}
 if (sostituzione == "N336E") {valore_pssm = "1";}
 if (sostituzione == "L75K") {valore_pssm = "1";}
 if (sostituzione == "K326I") {valore_pssm = "1";}
 if (sostituzione == "A352V") {valore_pssm = "1";}
 if (sostituzione == "D255Q") {valore_pssm = "1";}
 if (sostituzione == "N278T") {valore_pssm = "1";}
 if (sostituzione == "D161E") {valore_pssm = "1";}
 if (sostituzione == "Q330K") {valore_pssm = "1";}
 if (sostituzione == "N336Q") {valore_pssm = "1";}
 if (sostituzione == "S62N") {valore_pssm = "1";}
 if (sostituzione == "Y200F") {valore_pssm = "1";}
 if (sostituzione == "N122K") {valore_pssm = "1";}
 if (sostituzione == "V269E") {valore_pssm = "1";}
 if (sostituzione == "V254Q") {valore_pssm = "1";}
 if (sostituzione == "D255S") {valore_pssm = "1";}
 if (sostituzione == "A13E") {valore_pssm = "1";}
 if (sostituzione == "R252P") {valore_pssm = "1";}
 if (sostituzione == "D255E") {valore_pssm = "1";}
 if (sostituzione == "Q279E") {valore_pssm = "1";}
 if (sostituzione == "F169Y") {valore_pssm = "1";}
 if (sostituzione == "R252K") {valore_pssm = "1";}
 if (sostituzione == "M353L") {valore_pssm = "1";}
 if (sostituzione == "D33E") {valore_pssm = "0";}
 if (sostituzione == "L75T") {valore_pssm = "0";}
 if (sostituzione == "L131F") {valore_pssm = "0";}
 if (sostituzione == "L45N") {valore_pssm = "0";}
 if (sostituzione == "L54I") {valore_pssm = "0";}
 if (sostituzione == "K140Y") {valore_pssm = "0";}
 if (sostituzione == "S176T") {valore_pssm = "0";}
 if (sostituzione == "D182K") {valore_pssm = "0";}
 if (sostituzione == "L189R") {valore_pssm = "0";}
 if (sostituzione == "A37G") {valore_pssm = "0";}
 if (sostituzione == "K140M") {valore_pssm = "0";}
 if (sostituzione == "L189A") {valore_pssm = "0";}
 if (sostituzione == "Y222V") {valore_pssm = "0";}
 if (sostituzione == "A291K") {valore_pssm = "0";}
 if (sostituzione == "N336T") {valore_pssm = "0";}
 if (sostituzione == "V137A") {valore_pssm = "0";}
 if (sostituzione == "D233Q") {valore_pssm = "0";}
 if (sostituzione == "E74D") {valore_pssm = "0";}
 if (sostituzione == "A135S") {valore_pssm = "0";}
 if (sostituzione == "F337I") {valore_pssm = "0";}
 if (sostituzione == "Q330C") {valore_pssm = "0";}
 if (sostituzione == "W349H") {valore_pssm = "0";}
 if (sostituzione == "A230K") {valore_pssm = "0";}
 if (sostituzione == "A29V") {valore_pssm = "0";}
 if (sostituzione == "K67Q") {valore_pssm = "0";}
 if (sostituzione == "Q119A") {valore_pssm = "0";}
 if (sostituzione == "L177G") {valore_pssm = "0";}
 if (sostituzione == "R363F") {valore_pssm = "0";}
 if (sostituzione == "S405T") {valore_pssm = "0";}
 if (sostituzione == "L8T") {valore_pssm = "0";}
 if (sostituzione == "Y152V") {valore_pssm = "0";}
 if (sostituzione == "A230P") {valore_pssm = "0";}
 if (sostituzione == "V256Y") {valore_pssm = "0";}
 if (sostituzione == "S23P") {valore_pssm = "0";}
 if (sostituzione == "L166F") {valore_pssm = "0";}
 if (sostituzione == "R193K") {valore_pssm = "0";}
 if (sostituzione == "A381G") {valore_pssm = "0";}
 if (sostituzione == "I26V") {valore_pssm = "0";}
 if (sostituzione == "K314P") {valore_pssm = "0";}
 if (sostituzione == "F337Y") {valore_pssm = "0";}
 if (sostituzione == "S188H") {valore_pssm = "0";}
 if (sostituzione == "Q212P") {valore_pssm = "0";}
 if (sostituzione == "I354L") {valore_pssm = "0";}
 if (sostituzione == "A84L") {valore_pssm = "0";}
 if (sostituzione == "M208V") {valore_pssm = "0";}
 if (sostituzione == "L300I") {valore_pssm = "0";}
 if (sostituzione == "L89V") {valore_pssm = "0";}
 if (sostituzione == "K140Q") {valore_pssm = "0";}
 if (sostituzione == "F145Y") {valore_pssm = "0";}
 if (sostituzione == "K240L") {valore_pssm = "0";}
 if (sostituzione == "L331I") {valore_pssm = "0";}
 if (sostituzione == "S371R") {valore_pssm = "0";}
 if (sostituzione == "R118P") {valore_pssm = "0";}
 if (sostituzione == "N336D") {valore_pssm = "0";}
 if (sostituzione == "L347E") {valore_pssm = "0";}
 if (sostituzione == "P27Y") {valore_pssm = "0";}
 if (sostituzione == "F295I") {valore_pssm = "0";}
 if (sostituzione == "Q333R") {valore_pssm = "0";}
 if (sostituzione == "A368Q") {valore_pssm = "0";}
 if (sostituzione == "C90N") {valore_pssm = "0";}
 if (sostituzione == "P98Y") {valore_pssm = "0";}
 if (sostituzione == "N122E") {valore_pssm = "0";}
 if (sostituzione == "N298T") {valore_pssm = "0";}
 if (sostituzione == "A368S") {valore_pssm = "0";}
 if (sostituzione == "R220A") {valore_pssm = "0";}
 if (sostituzione == "M51R") {valore_pssm = "0";}
 if (sostituzione == "S62Q") {valore_pssm = "0";}
 if (sostituzione == "R252V") {valore_pssm = "0";}
 if (sostituzione == "L310V") {valore_pssm = "0";}
 if (sostituzione == "L347Q") {valore_pssm = "0";}
 if (sostituzione == "D175T") {valore_pssm = "0";}
 if (sostituzione == "R363Y") {valore_pssm = "0";}
 if (sostituzione == "F229Y") {valore_pssm = "0";}
 if (sostituzione == "S247T") {valore_pssm = "0";}
 if (sostituzione == "W349R") {valore_pssm = "0";}
 if (sostituzione == "K140F") {valore_pssm = "0";}
 if (sostituzione == "Q221E") {valore_pssm = "0";}
 if (sostituzione == "T366H") {valore_pssm = "0";}
 if (sostituzione == "E79D") {valore_pssm = "0";}
 if (sostituzione == "L177V") {valore_pssm = "0";}
 if (sostituzione == "L180V") {valore_pssm = "0";}
 if (sostituzione == "R220K") {valore_pssm = "0";}
 if (sostituzione == "V254A") {valore_pssm = "0";}
 if (sostituzione == "A307N") {valore_pssm = "0";}
 if (sostituzione == "Q333K") {valore_pssm = "0";}
 if (sostituzione == "S78Q") {valore_pssm = "0";}
 if (sostituzione == "K240T") {valore_pssm = "0";}
 if (sostituzione == "S297G") {valore_pssm = "0";}
 if (sostituzione == "L310F") {valore_pssm = "0";}
 if (sostituzione == "K326T") {valore_pssm = "0";}
 if (sostituzione == "Q333E") {valore_pssm = "0";}
 if (sostituzione == "A230S") {valore_pssm = "0";}
 if (sostituzione == "K374R") {valore_pssm = "0";}
 if (sostituzione == "L189E") {valore_pssm = "0";}
 if (sostituzione == "N249K") {valore_pssm = "0";}
 if (sostituzione == "A307E") {valore_pssm = "0";}
 if (sostituzione == "Y329R") {valore_pssm = "0";}
 if (sostituzione == "M208Q") {valore_pssm = "0";}
 if (sostituzione == "L14I") {valore_pssm = "0";}
 if (sostituzione == "D61R") {valore_pssm = "0";}
 if (sostituzione == "I270V") {valore_pssm = "0";}
 if (sostituzione == "S371K") {valore_pssm = "0";}
 if (sostituzione == "S176Q") {valore_pssm = "0";}
 if (sostituzione == "K237D") {valore_pssm = "0";}
 if (sostituzione == "L177R") {valore_pssm = "0";}
 if (sostituzione == "W277T") {valore_pssm = "0";}
 if (sostituzione == "Q212E") {valore_pssm = "0";}
 if (sostituzione == "V390E") {valore_pssm = "0";}
 if (sostituzione == "Q416R") {valore_pssm = "0";}
 if (sostituzione == "E59D") {valore_pssm = "0";}
 if (sostituzione == "H302T") {valore_pssm = "0";}
 if (sostituzione == "F337L") {valore_pssm = "0";}
 if (sostituzione == "R17V") {valore_pssm = "0";}
 if (sostituzione == "N179R") {valore_pssm = "0";}
 if (sostituzione == "D255K") {valore_pssm = "0";}
 if (sostituzione == "F149L") {valore_pssm = "0";}
 if (sostituzione == "K185P") {valore_pssm = "0";}
 if (sostituzione == "I407V") {valore_pssm = "0";}
 if (sostituzione == "Q107V") {valore_pssm = "0";}
 if (sostituzione == "N278S") {valore_pssm = "0";}
 if (sostituzione == "L300V") {valore_pssm = "0";}
 if (sostituzione == "Q333S") {valore_pssm = "0";}
 if (sostituzione == "E341A") {valore_pssm = "0";}
 if (sostituzione == "S62H") {valore_pssm = "0";}
 if (sostituzione == "E103N") {valore_pssm = "0";}
 if (sostituzione == "L180P") {valore_pssm = "0";}
 if (sostituzione == "A181E") {valore_pssm = "0";}
 if (sostituzione == "D161S") {valore_pssm = "0";}
 if (sostituzione == "N249E") {valore_pssm = "0";}
 if (sostituzione == "V269I") {valore_pssm = "0";}
 if (sostituzione == "T282S") {valore_pssm = "0";}
 if (sostituzione == "G361M") {valore_pssm = "0";}
 if (sostituzione == "A143M") {valore_pssm = "0";}
 if (sostituzione == "L166H") {valore_pssm = "0";}
 if (sostituzione == "L177I") {valore_pssm = "0";}
 if (sostituzione == "L189N") {valore_pssm = "0";}
 if (sostituzione == "D255A") {valore_pssm = "0";}
 if (sostituzione == "G346D") {valore_pssm = "0";}
 if (sostituzione == "I239V") {valore_pssm = "0";}
 if (sostituzione == "D315A") {valore_pssm = "0";}
 if (sostituzione == "G334K") {valore_pssm = "0";}
 if (sostituzione == "M353I") {valore_pssm = "0";}
 if (sostituzione == "F149Y") {valore_pssm = "0";}
 if (sostituzione == "L177Q") {valore_pssm = "0";}
 if (sostituzione == "Q212H") {valore_pssm = "0";}
 if (sostituzione == "S247F") {valore_pssm = "0";}
 if (sostituzione == "Q330L") {valore_pssm = "0";}
 if (sostituzione == "A29S") {valore_pssm = "0";}
 if (sostituzione == "M72T") {valore_pssm = "0";}
 if (sostituzione == "S176P") {valore_pssm = "0";}
 if (sostituzione == "L206D") {valore_pssm = "0";}
 if (sostituzione == "A309D") {valore_pssm = "0";}
 if (sostituzione == "Q333Y") {valore_pssm = "0";}
 if (sostituzione == "S78E") {valore_pssm = "0";}
 if (sostituzione == "L129F") {valore_pssm = "0";}
 if (sostituzione == "V256F") {valore_pssm = "0";}
 if (sostituzione == "W277Y") {valore_pssm = "0";}
 if (sostituzione == "D315L") {valore_pssm = "0";}
 if (sostituzione == "S401L") {valore_pssm = "0";}
 if (sostituzione == "L54V") {valore_pssm = "0";}
 if (sostituzione == "V137M") {valore_pssm = "0";}
 if (sostituzione == "Q357R") {valore_pssm = "0";}
 if (sostituzione == "R17T") {valore_pssm = "0";}
 if (sostituzione == "D175E") {valore_pssm = "0";}
 if (sostituzione == "L206V") {valore_pssm = "0";}
 if (sostituzione == "Q221T") {valore_pssm = "0";}
 if (sostituzione == "D233F") {valore_pssm = "0";}
 if (sostituzione == "K326Q") {valore_pssm = "0";}
 if (sostituzione == "E48N") {valore_pssm = "0";}
 if (sostituzione == "S176G") {valore_pssm = "0";}
 if (sostituzione == "N249Y") {valore_pssm = "0";}
 if (sostituzione == "W340F") {valore_pssm = "0";}
 if (sostituzione == "L347D") {valore_pssm = "0";}
 if (sostituzione == "I354W") {valore_pssm = "0";}
 if (sostituzione == "wild") {valore_pssm = "0";}
 if (sostituzione == "R220G") {valore_pssm = "0";}
 if (sostituzione == "R49H") {valore_pssm = "0";}
 if (sostituzione == "S62A") {valore_pssm = "0";}
 if (sostituzione == "I219V") {valore_pssm = "0";}
 if (sostituzione == "R252I") {valore_pssm = "0";}
 if (sostituzione == "M290F") {valore_pssm = "0";}
 if (sostituzione == "L347A") {valore_pssm = "0";}
 if (sostituzione == "G360D") {valore_pssm = "0";}
 if (sostituzione == "G11V") {valore_pssm = "0";}
 if (sostituzione == "R220S") {valore_pssm = "0";}
 if (sostituzione == "S62E") {valore_pssm = "0";}
 if (sostituzione == "Q99G") {valore_pssm = "0";}
 if (sostituzione == "W277E") {valore_pssm = "0";}
 if (sostituzione == "N278Q") {valore_pssm = "0";}
 if (sostituzione == "L347H") {valore_pssm = "0";}
 if (sostituzione == "V22F") {valore_pssm = "0";}
 if (sostituzione == "N228T") {valore_pssm = "0";}
 if (sostituzione == "A348D") {valore_pssm = "0";}
 if (sostituzione == "V369I") {valore_pssm = "0";}
 if (sostituzione == "K67R") {valore_pssm = "0";}
 if (sostituzione == "L189H") {valore_pssm = "0";}
 if (sostituzione == "S241N") {valore_pssm = "0";}
 if (sostituzione == "G346Q") {valore_pssm = "0";}
 if (sostituzione == "S62K") {valore_pssm = "0";}
 if (sostituzione == "L75V") {valore_pssm = "0";}
 if (sostituzione == "A135T") {valore_pssm = "0";}
 if (sostituzione == "R220M") {valore_pssm = "0";}
 if (sostituzione == "E341S") {valore_pssm = "0";}
 if (sostituzione == "Q2K") {valore_pssm = "0";}
 if (sostituzione == "S62V") {valore_pssm = "0";}
 if (sostituzione == "A97E") {valore_pssm = "0";}
 if (sostituzione == "Q111K") {valore_pssm = "0";}
 if (sostituzione == "I219L") {valore_pssm = "0";}
 if (sostituzione == "R220Q") {valore_pssm = "0";}
 if (sostituzione == "Q221S") {valore_pssm = "0";}
 if (sostituzione == "M290L") {valore_pssm = "0";}
 if (sostituzione == "D313H") {valore_pssm = "0";}
 if (sostituzione == "E87N") {valore_pssm = "0";}
 if (sostituzione == "I239M") {valore_pssm = "0";}
 if (sostituzione == "D255T") {valore_pssm = "0";}
 if (sostituzione == "E79N") {valore_pssm = "0";}
 if (sostituzione == "R252Q") {valore_pssm = "0";}
 if (sostituzione == "E341T") {valore_pssm = "0";}
 if (sostituzione == "A37L") {valore_pssm = "0";}
 if (sostituzione == "S62T") {valore_pssm = "0";}
 if (sostituzione == "E87Q") {valore_pssm = "0";}
 if (sostituzione == "Q119S") {valore_pssm = "0";}
 if (sostituzione == "Q280K") {valore_pssm = "0";}
 if (sostituzione == "S62R") {valore_pssm = "0";}
 if (sostituzione == "K67E") {valore_pssm = "0";}
 if (sostituzione == "D175K") {valore_pssm = "0";}
 if (sostituzione == "N179T") {valore_pssm = "0";}
 if (sostituzione == "R252E") {valore_pssm = "0";}
 if (sostituzione == "N278K") {valore_pssm = "0";}
 if (sostituzione == "D153Q") {valore_pssm = "0";}
 if (sostituzione == "A230E") {valore_pssm = "0";}
 if (sostituzione == "D233E") {valore_pssm = "0";}
 if (sostituzione == "I253L") {valore_pssm = "0";}
 if (sostituzione == "L286M") {valore_pssm = "0";}
 if (sostituzione == "L331F") {valore_pssm = "0";}
 if (sostituzione == "V369L") {valore_pssm = "0";}
 if (sostituzione == "D255H") {valore_pssm = "0";}
 if (sostituzione == "M296I") {valore_pssm = "0";}
 if (sostituzione == "A307D") {valore_pssm = "0";}
 if (sostituzione == "E7D") {valore_pssm = "0";}
 if (sostituzione == "S65N") {valore_pssm = "0";}
 if (sostituzione == "Q111T") {valore_pssm = "0";}
 if (sostituzione == "E251A") {valore_pssm = "0";}
 if (sostituzione == "F295L") {valore_pssm = "0";}
 if (sostituzione == "A307S") {valore_pssm = "0";}
 if (sostituzione == "N336S") {valore_pssm = "0";}
 if (sostituzione == "P27L") {valore_pssm = "0";}
 if (sostituzione == "R220N") {valore_pssm = "0";}
 if (sostituzione == "N278A") {valore_pssm = "0";}
 if (sostituzione == "A309S") {valore_pssm = "0";}
 if (sostituzione == "N336G") {valore_pssm = "0";}
 if (sostituzione == "L347R") {valore_pssm = "0";}
 if (sostituzione == "S364K") {valore_pssm = "0";}
 if (sostituzione == "D33Q") {valore_pssm = "0";}
 if (sostituzione == "D61E") {valore_pssm = "0";}
 if (sostituzione == "E103Q") {valore_pssm = "0";}
 if (sostituzione == "K140E") {valore_pssm = "0";}
 if (sostituzione == "D182R") {valore_pssm = "0";}
 if (sostituzione == "R193A") {valore_pssm = "0";}
 if (sostituzione == "L286I") {valore_pssm = "0";}
 if (sostituzione == "I303M") {valore_pssm = "0";}
 if (sostituzione == "E341K") {valore_pssm = "0";}
 if (sostituzione == "A20F") {valore_pssm = "0";}
 if (sostituzione == "M208I") {valore_pssm = "0";}
 if (sostituzione == "I219G") {valore_pssm = "0";}
 if (sostituzione == "Q221D") {valore_pssm = "0";}
 if (sostituzione == "D233K") {valore_pssm = "0";}
 if (sostituzione == "T246N") {valore_pssm = "0";}
 if (sostituzione == "H302S") {valore_pssm = "0";}
 if (sostituzione == "Q333T") {valore_pssm = "0";}
 if (sostituzione == "L14V") {valore_pssm = "0";}
 if (sostituzione == "G171N") {valore_pssm = "0";}
 if (sostituzione == "Q280E") {valore_pssm = "0";}
 if (sostituzione == "A309E") {valore_pssm = "0";}
 if (sostituzione == "Q333D") {valore_pssm = "0";}
 if (sostituzione == "N336K") {valore_pssm = "0";}
 if (sostituzione == "Q119W") {valore_pssm = "0";}
 if (sostituzione == "S188A") {valore_pssm = "0";}
 if (sostituzione == "L206E") {valore_pssm = "0";}
 if (sostituzione == "T282A") {valore_pssm = "0";}
 if (sostituzione == "N298A") {valore_pssm = "0";}
 if (sostituzione == "R332H") {valore_pssm = "0";}
 if (sostituzione == "Q333N") {valore_pssm = "0";}
 if (sostituzione == "M96I") {valore_pssm = "0";}
 if (sostituzione == "D182H") {valore_pssm = "0";}
 if (sostituzione == "M208L") {valore_pssm = "0";}
 if (sostituzione == "A285S") {valore_pssm = "0";}
 if (sostituzione == "K67T") {valore_pssm = "0";}
 if (sostituzione == "L68V") {valore_pssm = "0";}
 if (sostituzione == "G150D") {valore_pssm = "0";}
 if (sostituzione == "D182N") {valore_pssm = "0";}
 if (sostituzione == "S247I") {valore_pssm = "0";}
 if (sostituzione == "G346H") {valore_pssm = "0";}
 if (sostituzione == "L8V") {valore_pssm = "0";}
 if (sostituzione == "L21T") {valore_pssm = "0";}
 if (sostituzione == "L68I") {valore_pssm = "0";}
 if (sostituzione == "E87K") {valore_pssm = "0";}
 if (sostituzione == "N179S") {valore_pssm = "0";}
 if (sostituzione == "H302D") {valore_pssm = "0";}
 if (sostituzione == "R404K") {valore_pssm = "0";}
 if (sostituzione == "S23A") {valore_pssm = "0";}
 if (sostituzione == "L166V") {valore_pssm = "0";}
 if (sostituzione == "L189V") {valore_pssm = "0";}
 if (sostituzione == "E251K") {valore_pssm = "0";}
 if (sostituzione == "W277A") {valore_pssm = "0";}
 if (sostituzione == "D55N") {valore_pssm = "0";}
 if (sostituzione == "S78K") {valore_pssm = "0";}
 if (sostituzione == "L120V") {valore_pssm = "0";}
 if (sostituzione == "N139K") {valore_pssm = "0";}
 if (sostituzione == "A181P") {valore_pssm = "0";}
 if (sostituzione == "V199I") {valore_pssm = "0";}
 if (sostituzione == "L206H") {valore_pssm = "0";}
 if (sostituzione == "I354Y") {valore_pssm = "0";}
 if (sostituzione == "V22I") {valore_pssm = "0";}
 if (sostituzione == "K67S") {valore_pssm = "0";}
 if (sostituzione == "L75Q") {valore_pssm = "0";}
 if (sostituzione == "A143G") {valore_pssm = "0";}
 if (sostituzione == "A181Q") {valore_pssm = "0";}
 if (sostituzione == "M208K") {valore_pssm = "0";}
 if (sostituzione == "Q221Y") {valore_pssm = "0";}
 if (sostituzione == "V254T") {valore_pssm = "0";}
 if (sostituzione == "W81L") {valore_pssm = "0";}
 if (sostituzione == "S148T") {valore_pssm = "0";}
 if (sostituzione == "L206T") {valore_pssm = "0";}
 if (sostituzione == "I219A") {valore_pssm = "0";}
 if (sostituzione == "F229T") {valore_pssm = "0";}
 if (sostituzione == "N278H") {valore_pssm = "0";}
 if (sostituzione == "I289V") {valore_pssm = "0";}
 if (sostituzione == "W349V") {valore_pssm = "0";}
 if (sostituzione == "I354R") {valore_pssm = "0";}
 if (sostituzione == "N5S") {valore_pssm = "0";}
 if (sostituzione == "A31P") {valore_pssm = "0";}
 if (sostituzione == "E74K") {valore_pssm = "0";}
 if (sostituzione == "L75S") {valore_pssm = "0";}
 if (sostituzione == "L75E") {valore_pssm = "0";}
 if (sostituzione == "K82A") {valore_pssm = "0";}
 if (sostituzione == "E87V") {valore_pssm = "0";}
 if (sostituzione == "S102A") {valore_pssm = "0";}
 if (sostituzione == "E103K") {valore_pssm = "0";}
 if (sostituzione == "R112K") {valore_pssm = "0";}
 if (sostituzione == "L206K") {valore_pssm = "0";}
 if (sostituzione == "M208H") {valore_pssm = "0";}
 if (sostituzione == "W209G") {valore_pssm = "0";}
 if (sostituzione == "Q280S") {valore_pssm = "0";}
 if (sostituzione == "A31E") {valore_pssm = "0";}
 if (sostituzione == "Q99E") {valore_pssm = "0";}
 if (sostituzione == "D175S") {valore_pssm = "0";}
 if (sostituzione == "L206I") {valore_pssm = "0";}
 if (sostituzione == "K237P") {valore_pssm = "0";}
 if (sostituzione == "V269L") {valore_pssm = "0";}
 if (sostituzione == "N272H") {valore_pssm = "0";}
 if (sostituzione == "Y329K") {valore_pssm = "0";}
 if (sostituzione == "G334E") {valore_pssm = "0";}
 if (sostituzione == "N336R") {valore_pssm = "0";}
 if (sostituzione == "P6R") {valore_pssm = "0";}
 if (sostituzione == "Q57R") {valore_pssm = "0";}
 if (sostituzione == "L189T") {valore_pssm = "0";}
 if (sostituzione == "S276T") {valore_pssm = "0";}
 if (sostituzione == "L347S") {valore_pssm = "0";}
 if (sostituzione == "L347T") {valore_pssm = "0";}
 if (sostituzione == "Q57E") {valore_pssm = "0";}
 if (sostituzione == "L75H") {valore_pssm = "0";}
 if (sostituzione == "A97G") {valore_pssm = "0";}
 if (sostituzione == "Q221K") {valore_pssm = "0";}
 if (sostituzione == "K237E") {valore_pssm = "0";}
 if (sostituzione == "N249A") {valore_pssm = "0";}
 if (sostituzione == "W277D") {valore_pssm = "0";}
 if (sostituzione == "A309Q") {valore_pssm = "0";}
 if (sostituzione == "K240V") {valore_pssm = "0";}
 if (sostituzione == "M290V") {valore_pssm = "0";}
 if (sostituzione == "D313E") {valore_pssm = "0";}
 if (sostituzione == "A348R") {valore_pssm = "0";}
 if (sostituzione == "R392V") {valore_pssm = "0";}
 if (sostituzione == "L189S") {valore_pssm = "0";}
 if (sostituzione == "H225S") {valore_pssm = "0";}
 if (sostituzione == "E251S") {valore_pssm = "0";}
 if (sostituzione == "V269M") {valore_pssm = "0";}
 if (sostituzione == "G334D") {valore_pssm = "0";}
 if (sostituzione == "A350V") {valore_pssm = "0";}
 if (sostituzione == "S364Q") {valore_pssm = "0";}
 if (sostituzione == "D61H") {valore_pssm = "0";}
 if (sostituzione == "E87T") {valore_pssm = "0";}
 if (sostituzione == "E103T") {valore_pssm = "0";}
 if (sostituzione == "A143S") {valore_pssm = "0";}
 if (sostituzione == "Y151K") {valore_pssm = "0";}
 if (sostituzione == "L206S") {valore_pssm = "0";}
 if (sostituzione == "Q221A") {valore_pssm = "0";}
 if (sostituzione == "A288T") {valore_pssm = "0";}
 if (sostituzione == "H302A") {valore_pssm = "0";}
 if (sostituzione == "F337V") {valore_pssm = "0";}
 if (sostituzione == "G346S") {valore_pssm = "0";}
 if (sostituzione == "A352I") {valore_pssm = "0";}
 if (sostituzione == "I354V") {valore_pssm = "0";}
 if (sostituzione == "R118A") {valore_pssm = "0";}
 if (sostituzione == "N179Q") {valore_pssm = "0";}
 if (sostituzione == "L243I") {valore_pssm = "0";}
 if (sostituzione == "N249S") {valore_pssm = "0";}
 if (sostituzione == "A309K") {valore_pssm = "0";}
 if (sostituzione == "D313K") {valore_pssm = "0";}
 if (sostituzione == "I319V") {valore_pssm = "0";}
 if (sostituzione == "L347V") {valore_pssm = "0";}
 if (sostituzione == "I367T") {valore_pssm = "0";}
 if (sostituzione == "S401V") {valore_pssm = "0";}
 if (sostituzione == "R402T") {valore_pssm = "0";}
 if (sostituzione == "S62I") {valore_pssm = "0";}
 if (sostituzione == "S62F") {valore_pssm = "0";}
 if (sostituzione == "M208R") {valore_pssm = "0";}
 if (sostituzione == "K240I") {valore_pssm = "0";}
 if (sostituzione == "D313A") {valore_pssm = "0";}
 if (sostituzione == "Q330A") {valore_pssm = "0";}
 if (sostituzione == "W349S") {valore_pssm = "0";}
 if (sostituzione == "I359M") {valore_pssm = "0";}
 if (sostituzione == "V376L") {valore_pssm = "0";}
 if (sostituzione == "D25Q") {valore_pssm = "0";}
 if (sostituzione == "D109N") {valore_pssm = "0";}
 if (sostituzione == "L166I") {valore_pssm = "0";}
 if (sostituzione == "A181Y") {valore_pssm = "0";}
 if (sostituzione == "F211Y") {valore_pssm = "0";}
 if (sostituzione == "V254G") {valore_pssm = "0";}
 if (sostituzione == "W277N") {valore_pssm = "0";}
 if (sostituzione == "W349K") {valore_pssm = "0";}
 if (sostituzione == "L8I") {valore_pssm = "0";}
 if (sostituzione == "I154M") {valore_pssm = "0";}
 if (sostituzione == "F169L") {valore_pssm = "0";}
 if (sostituzione == "S176A") {valore_pssm = "0";}
 if (sostituzione == "E203S") {valore_pssm = "0";}
 if (sostituzione == "N336L") {valore_pssm = "0";}
 if (sostituzione == "S364E") {valore_pssm = "0";}
 if (sostituzione == "A368E") {valore_pssm = "0";}
 if (sostituzione == "T410S") {valore_pssm = "0";}
 if (sostituzione == "S65K") {valore_pssm = "0";}
 if (sostituzione == "Y173F") {valore_pssm = "0";}
 if (sostituzione == "L177E") {valore_pssm = "0";}
 if (sostituzione == "A230H") {valore_pssm = "0";}
 if (sostituzione == "N249Q") {valore_pssm = "0";}
 if (sostituzione == "N249R") {valore_pssm = "0";}
 if (sostituzione == "I253M") {valore_pssm = "0";}
 if (sostituzione == "N278R") {valore_pssm = "0";}
 if (sostituzione == "V281K") {valore_pssm = "0";}
 if (sostituzione == "K314E") {valore_pssm = "0";}
 if (sostituzione == "L32F") {valore_pssm = "0";}
 if (sostituzione == "L206R") {valore_pssm = "0";}
 if (sostituzione == "M208T") {valore_pssm = "0";}
 if (sostituzione == "Y222M") {valore_pssm = "0";}
 if (sostituzione == "L243V") {valore_pssm = "0";}
 if (sostituzione == "L243Y") {valore_pssm = "0";}
 if (sostituzione == "D244N") {valore_pssm = "0";}
 if (sostituzione == "D313T") {valore_pssm = "0";}
 if (sostituzione == "A368T") {valore_pssm = "0";}
 if (sostituzione == "L415I") {valore_pssm = "0";}
 if (sostituzione == "D61Q") {valore_pssm = "0";}
 if (sostituzione == "L206F") {valore_pssm = "0";}
 if (sostituzione == "A230T") {valore_pssm = "0";}
 if (sostituzione == "K237S") {valore_pssm = "0";}
 if (sostituzione == "K308H") {valore_pssm = "0";}
 if (sostituzione == "A318K") {valore_pssm = "0";}
 if (sostituzione == "A352T") {valore_pssm = "0";}
 if (sostituzione == "I384V") {valore_pssm = "0";}
 if (sostituzione == "P27A") {valore_pssm = "0";}
 if (sostituzione == "S62G") {valore_pssm = "0";}
 if (sostituzione == "S62P") {valore_pssm = "0";}
 if (sostituzione == "S62C") {valore_pssm = "0";}
 if (sostituzione == "E79T") {valore_pssm = "0";}
 if (sostituzione == "V137S") {valore_pssm = "0";}
 if (sostituzione == "L206Q") {valore_pssm = "0";}
 if (sostituzione == "A230Q") {valore_pssm = "0";}
 if (sostituzione == "S235N") {valore_pssm = "0";}
 if (sostituzione == "D255V") {valore_pssm = "0";}
 if (sostituzione == "G346E") {valore_pssm = "0";}
 if (sostituzione == "L415V") {valore_pssm = "0";}
 if (sostituzione == "V22T") {valore_pssm = "0";}
 if (sostituzione == "T41E") {valore_pssm = "0";}
 if (sostituzione == "N53D") {valore_pssm = "0";}
 if (sostituzione == "S102T") {valore_pssm = "0";}
 if (sostituzione == "A143Q") {valore_pssm = "0";}
 if (sostituzione == "N215P") {valore_pssm = "0";}
 if (sostituzione == "E218F") {valore_pssm = "0";}
 if (sostituzione == "A230R") {valore_pssm = "0";}
 if (sostituzione == "T246A") {valore_pssm = "0";}
 if (sostituzione == "F273G") {valore_pssm = "0";}
 if (sostituzione == "K308M") {valore_pssm = "0";}
 if (sostituzione == "L75I") {valore_pssm = "0";}
 if (sostituzione == "A84M") {valore_pssm = "0";}
 if (sostituzione == "G150N") {valore_pssm = "0";}
 if (sostituzione == "N179E") {valore_pssm = "0";}
 if (sostituzione == "N249L") {valore_pssm = "0";}
 if (sostituzione == "I359V") {valore_pssm = "0";}
 if (sostituzione == "V376I") {valore_pssm = "0";}
 if (sostituzione == "H46S") {valore_pssm = "0";}
 if (sostituzione == "A97K") {valore_pssm = "0";}
 if (sostituzione == "R118G") {valore_pssm = "0";}
 if (sostituzione == "S126A") {valore_pssm = "0";}
 if (sostituzione == "D313S") {valore_pssm = "0";}
 if (sostituzione == "Q333H") {valore_pssm = "0";}
 if (sostituzione == "D335N") {valore_pssm = "0";}
 if (sostituzione == "A368K") {valore_pssm = "0";}
 if (sostituzione == "S401I") {valore_pssm = "0";}
 if (sostituzione == "S401T") {valore_pssm = "0";}
 if (sostituzione == "Q57V") {valore_pssm = "-1";}
 if (sostituzione == "S62D") {valore_pssm = "-1";}
 if (sostituzione == "N179I") {valore_pssm = "-1";}
 if (sostituzione == "D233Y") {valore_pssm = "-1";}
 if (sostituzione == "L415Y") {valore_pssm = "-1";}
 if (sostituzione == "D61V") {valore_pssm = "-1";}
 if (sostituzione == "D61T") {valore_pssm = "-1";}
 if (sostituzione == "Q107I") {valore_pssm = "-1";}
 if (sostituzione == "Q119V") {valore_pssm = "-1";}
 if (sostituzione == "A181K") {valore_pssm = "-1";}
 if (sostituzione == "P210N") {valore_pssm = "-1";}
 if (sostituzione == "K237R") {valore_pssm = "-1";}
 if (sostituzione == "K308I") {valore_pssm = "-1";}
 if (sostituzione == "R332S") {valore_pssm = "-1";}
 if (sostituzione == "R332Q") {valore_pssm = "-1";}
 if (sostituzione == "S364T") {valore_pssm = "-1";}
 if (sostituzione == "I367V") {valore_pssm = "-1";}
 if (sostituzione == "A84V") {valore_pssm = "-1";}
 if (sostituzione == "L177H") {valore_pssm = "-1";}
 if (sostituzione == "L206C") {valore_pssm = "-1";}
 if (sostituzione == "F211L") {valore_pssm = "-1";}
 if (sostituzione == "Q212K") {valore_pssm = "-1";}
 if (sostituzione == "C174N") {valore_pssm = "-1";}
 if (sostituzione == "S176N") {valore_pssm = "-1";}
 if (sostituzione == "V199A") {valore_pssm = "-1";}
 if (sostituzione == "Q212D") {valore_pssm = "-1";}
 if (sostituzione == "N228V") {valore_pssm = "-1";}
 if (sostituzione == "Q280H") {valore_pssm = "-1";}
 if (sostituzione == "D313R") {valore_pssm = "-1";}
 if (sostituzione == "A13Q") {valore_pssm = "-1";}
 if (sostituzione == "N122Q") {valore_pssm = "-1";}
 if (sostituzione == "D153V") {valore_pssm = "-1";}
 if (sostituzione == "L180I") {valore_pssm = "-1";}
 if (sostituzione == "S197N") {valore_pssm = "-1";}
 if (sostituzione == "D233G") {valore_pssm = "-1";}
 if (sostituzione == "W277V") {valore_pssm = "-1";}
 if (sostituzione == "L311V") {valore_pssm = "-1";}
 if (sostituzione == "F337Q") {valore_pssm = "-1";}
 if (sostituzione == "L21A") {valore_pssm = "-1";}
 if (sostituzione == "D33Y") {valore_pssm = "-1";}
 if (sostituzione == "L45D") {valore_pssm = "-1";}
 if (sostituzione == "P98K") {valore_pssm = "-1";}
 if (sostituzione == "L131I") {valore_pssm = "-1";}
 if (sostituzione == "A230N") {valore_pssm = "-1";}
 if (sostituzione == "S247H") {valore_pssm = "-1";}
 if (sostituzione == "W277H") {valore_pssm = "-1";}
 if (sostituzione == "D315Q") {valore_pssm = "-1";}
 if (sostituzione == "T41K") {valore_pssm = "-1";}
 if (sostituzione == "M96A") {valore_pssm = "-1";}
 if (sostituzione == "K213N") {valore_pssm = "-1";}
 if (sostituzione == "K213E") {valore_pssm = "-1";}
 if (sostituzione == "D313Q") {valore_pssm = "-1";}
 if (sostituzione == "G346K") {valore_pssm = "-1";}
 if (sostituzione == "I359T") {valore_pssm = "-1";}
 if (sostituzione == "Q111E") {valore_pssm = "-1";}
 if (sostituzione == "Q157K") {valore_pssm = "-1";}
 if (sostituzione == "L180R") {valore_pssm = "-1";}
 if (sostituzione == "L189I") {valore_pssm = "-1";}
 if (sostituzione == "Q221H") {valore_pssm = "-1";}
 if (sostituzione == "D315K") {valore_pssm = "-1";}
 if (sostituzione == "V316M") {valore_pssm = "-1";}
 if (sostituzione == "L331A") {valore_pssm = "-1";}
 if (sostituzione == "L425I") {valore_pssm = "-1";}
 if (sostituzione == "L428I") {valore_pssm = "-1";}
 if (sostituzione == "L429I") {valore_pssm = "-1";}
 if (sostituzione == "P27F") {valore_pssm = "-1";}
 if (sostituzione == "S78A") {valore_pssm = "-1";}
 if (sostituzione == "N122R") {valore_pssm = "-1";}
 if (sostituzione == "G183E") {valore_pssm = "-1";}
 if (sostituzione == "L206G") {valore_pssm = "-1";}
 if (sostituzione == "Q280F") {valore_pssm = "-1";}
 if (sostituzione == "E341M") {valore_pssm = "-1";}
 if (sostituzione == "A368H") {valore_pssm = "-1";}
 if (sostituzione == "V137L") {valore_pssm = "-1";}
 if (sostituzione == "L177T") {valore_pssm = "-1";}
 if (sostituzione == "N179G") {valore_pssm = "-1";}
 if (sostituzione == "R193S") {valore_pssm = "-1";}
 if (sostituzione == "W245Y") {valore_pssm = "-1";}
 if (sostituzione == "N249F") {valore_pssm = "-1";}
 if (sostituzione == "Q312S") {valore_pssm = "-1";}
 if (sostituzione == "N139T") {valore_pssm = "-1";}
 if (sostituzione == "F145M") {valore_pssm = "-1";}
 if (sostituzione == "Y152Q") {valore_pssm = "-1";}
 if (sostituzione == "P214V") {valore_pssm = "-1";}
 if (sostituzione == "S235T") {valore_pssm = "-1";}
 if (sostituzione == "N249T") {valore_pssm = "-1";}
 if (sostituzione == "W262F") {valore_pssm = "-1";}
 if (sostituzione == "H302Q") {valore_pssm = "-1";}
 if (sostituzione == "A309N") {valore_pssm = "-1";}
 if (sostituzione == "Q312T") {valore_pssm = "-1";}
 if (sostituzione == "V339I") {valore_pssm = "-1";}
 if (sostituzione == "L425V") {valore_pssm = "-1";}
 if (sostituzione == "L428V") {valore_pssm = "-1";}
 if (sostituzione == "L429V") {valore_pssm = "-1";}
 if (sostituzione == "M76I") {valore_pssm = "-1";}
 if (sostituzione == "A135G") {valore_pssm = "-1";}
 if (sostituzione == "R220E") {valore_pssm = "-1";}
 if (sostituzione == "T246V") {valore_pssm = "-1";}
 if (sostituzione == "S247A") {valore_pssm = "-1";}
 if (sostituzione == "F295V") {valore_pssm = "-1";}
 if (sostituzione == "Q330E") {valore_pssm = "-1";}
 if (sostituzione == "F383I") {valore_pssm = "-1";}
 if (sostituzione == "S102P") {valore_pssm = "-1";}
 if (sostituzione == "Q119T") {valore_pssm = "-1";}
 if (sostituzione == "R193T") {valore_pssm = "-1";}
 if (sostituzione == "L206P") {valore_pssm = "-1";}
 if (sostituzione == "M208Y") {valore_pssm = "-1";}
 if (sostituzione == "T246H") {valore_pssm = "-1";}
 if (sostituzione == "S247K") {valore_pssm = "-1";}
 if (sostituzione == "S247E") {valore_pssm = "-1";}
 if (sostituzione == "Q330V") {valore_pssm = "-1";}
 if (sostituzione == "N336V") {valore_pssm = "-1";}
 if (sostituzione == "L3I") {valore_pssm = "-1";}
 if (sostituzione == "L10I") {valore_pssm = "-1";}
 if (sostituzione == "A13K") {valore_pssm = "-1";}
 if (sostituzione == "L21V") {valore_pssm = "-1";}
 if (sostituzione == "S62L") {valore_pssm = "-1";}
 if (sostituzione == "V77A") {valore_pssm = "-1";}
 if (sostituzione == "A84I") {valore_pssm = "-1";}
 if (sostituzione == "K140H") {valore_pssm = "-1";}
 if (sostituzione == "A307H") {valore_pssm = "-1";}
 if (sostituzione == "L347I") {valore_pssm = "-1";}
 if (sostituzione == "L387I") {valore_pssm = "-1";}
 if (sostituzione == "L388I") {valore_pssm = "-1";}
 if (sostituzione == "L394I") {valore_pssm = "-1";}
 if (sostituzione == "L403I") {valore_pssm = "-1";}
 if (sostituzione == "L414I") {valore_pssm = "-1";}
 if (sostituzione == "L417I") {valore_pssm = "-1";}
 if (sostituzione == "Q107E") {valore_pssm = "-1";}
 if (sostituzione == "Q119E") {valore_pssm = "-1";}
 if (sostituzione == "L120M") {valore_pssm = "-1";}
 if (sostituzione == "A135Q") {valore_pssm = "-1";}
 if (sostituzione == "D182T") {valore_pssm = "-1";}
 if (sostituzione == "N228I") {valore_pssm = "-1";}
 if (sostituzione == "N249V") {valore_pssm = "-1";}
 if (sostituzione == "D255N") {valore_pssm = "-1";}
 if (sostituzione == "K314R") {valore_pssm = "-1";}
 if (sostituzione == "D315V") {valore_pssm = "-1";}
 if (sostituzione == "N336Y") {valore_pssm = "-1";}
 if (sostituzione == "G346T") {valore_pssm = "-1";}
 if (sostituzione == "D61F") {valore_pssm = "-1";}
 if (sostituzione == "S62Y") {valore_pssm = "-1";}
 if (sostituzione == "E79K") {valore_pssm = "-1";}
 if (sostituzione == "R220T") {valore_pssm = "-1";}
 if (sostituzione == "M290A") {valore_pssm = "-1";}
 if (sostituzione == "A291S") {valore_pssm = "-1";}
 if (sostituzione == "A370S") {valore_pssm = "-1";}
 if (sostituzione == "S424T") {valore_pssm = "-1";}
 if (sostituzione == "L3V") {valore_pssm = "-1";}
 if (sostituzione == "L10V") {valore_pssm = "-1";}
 if (sostituzione == "Q111S") {valore_pssm = "-1";}
 if (sostituzione == "A135K") {valore_pssm = "-1";}
 if (sostituzione == "D175N") {valore_pssm = "-1";}
 if (sostituzione == "F211T") {valore_pssm = "-1";}
 if (sostituzione == "F248M") {valore_pssm = "-1";}
 if (sostituzione == "N278V") {valore_pssm = "-1";}
 if (sostituzione == "A285T") {valore_pssm = "-1";}
 if (sostituzione == "A307K") {valore_pssm = "-1";}
 if (sostituzione == "Y329T") {valore_pssm = "-1";}
 if (sostituzione == "L387V") {valore_pssm = "-1";}
 if (sostituzione == "L388V") {valore_pssm = "-1";}
 if (sostituzione == "L394V") {valore_pssm = "-1";}
 if (sostituzione == "L403V") {valore_pssm = "-1";}
 if (sostituzione == "L414V") {valore_pssm = "-1";}
 if (sostituzione == "L417V") {valore_pssm = "-1";}
 if (sostituzione == "Q57K") {valore_pssm = "-1";}
 if (sostituzione == "D61I") {valore_pssm = "-1";}
 if (sostituzione == "L106V") {valore_pssm = "-1";}
 if (sostituzione == "L177A") {valore_pssm = "-1";}
 if (sostituzione == "M208A") {valore_pssm = "-1";}
 if (sostituzione == "E218W") {valore_pssm = "-1";}
 if (sostituzione == "W277L") {valore_pssm = "-1";}
 if (sostituzione == "R342G") {valore_pssm = "-1";}
 if (sostituzione == "A368R") {valore_pssm = "-1";}
 if (sostituzione == "Q119K") {valore_pssm = "-1";}
 if (sostituzione == "S176E") {valore_pssm = "-1";}
 if (sostituzione == "E203Q") {valore_pssm = "-1";}
 if (sostituzione == "S304D") {valore_pssm = "-1";}
 if (sostituzione == "M353Y") {valore_pssm = "-1";}
 if (sostituzione == "S371Q") {valore_pssm = "-1";}
 if (sostituzione == "L16A") {valore_pssm = "-1";}
 if (sostituzione == "P98G") {valore_pssm = "-1";}
 if (sostituzione == "Y152H") {valore_pssm = "-1";}
 if (sostituzione == "A181M") {valore_pssm = "-1";}
 if (sostituzione == "K213D") {valore_pssm = "-1";}
 if (sostituzione == "Y222F") {valore_pssm = "-1";}
 if (sostituzione == "I232V") {valore_pssm = "-1";}
 if (sostituzione == "K237N") {valore_pssm = "-1";}
 if (sostituzione == "S247R") {valore_pssm = "-1";}
 if (sostituzione == "F248V") {valore_pssm = "-1";}
 if (sostituzione == "I253V") {valore_pssm = "-1";}
 if (sostituzione == "P305Q") {valore_pssm = "-1";}
 if (sostituzione == "L311I") {valore_pssm = "-1";}
 if (sostituzione == "S364A") {valore_pssm = "-1";}
 if (sostituzione == "S371T") {valore_pssm = "-1";}
 if (sostituzione == "P27V") {valore_pssm = "-1";}
 if (sostituzione == "K67M") {valore_pssm = "-1";}
 if (sostituzione == "K130H") {valore_pssm = "-1";}
 if (sostituzione == "N139E") {valore_pssm = "-1";}
 if (sostituzione == "S176K") {valore_pssm = "-1";}
 if (sostituzione == "D182S") {valore_pssm = "-1";}
 if (sostituzione == "S197T") {valore_pssm = "-1";}
 if (sostituzione == "L206M") {valore_pssm = "-1";}
 if (sostituzione == "Y207F") {valore_pssm = "-1";}
 if (sostituzione == "N215I") {valore_pssm = "-1";}
 if (sostituzione == "R220H") {valore_pssm = "-1";}
 if (sostituzione == "T246Y") {valore_pssm = "-1";}
 if (sostituzione == "A307Q") {valore_pssm = "-1";}
 if (sostituzione == "K314S") {valore_pssm = "-1";}
 if (sostituzione == "N336A") {valore_pssm = "-1";}
 if (sostituzione == "G346R") {valore_pssm = "-1";}
 if (sostituzione == "I354M") {valore_pssm = "-1";}
 if (sostituzione == "V390I") {valore_pssm = "-1";}
 if (sostituzione == "Q416K") {valore_pssm = "-1";}
 if (sostituzione == "A29I") {valore_pssm = "-1";}
 if (sostituzione == "M51G") {valore_pssm = "-1";}
 if (sostituzione == "Q57H") {valore_pssm = "-1";}
 if (sostituzione == "K67N") {valore_pssm = "-1";}
 if (sostituzione == "P98F") {valore_pssm = "-1";}
 if (sostituzione == "P98S") {valore_pssm = "-1";}
 if (sostituzione == "N122H") {valore_pssm = "-1";}
 if (sostituzione == "Y152K") {valore_pssm = "-1";}
 if (sostituzione == "M208S") {valore_pssm = "-1";}
 if (sostituzione == "Q221G") {valore_pssm = "-1";}
 if (sostituzione == "L243A") {valore_pssm = "-1";}
 if (sostituzione == "S247Q") {valore_pssm = "-1";}
 if (sostituzione == "F248T") {valore_pssm = "-1";}
 if (sostituzione == "I253W") {valore_pssm = "-1";}
 if (sostituzione == "L324I") {valore_pssm = "-1";}
 if (sostituzione == "E341L") {valore_pssm = "-1";}
 if (sostituzione == "L16V") {valore_pssm = "-1";}
 if (sostituzione == "R30Y") {valore_pssm = "-1";}
 if (sostituzione == "D61S") {valore_pssm = "-1";}
 if (sostituzione == "L75C") {valore_pssm = "-1";}
 if (sostituzione == "K140L") {valore_pssm = "-1";}
 if (sostituzione == "S176H") {valore_pssm = "-1";}
 if (sostituzione == "A230V") {valore_pssm = "-1";}
 if (sostituzione == "V254E") {valore_pssm = "-1";}
 if (sostituzione == "A307V") {valore_pssm = "-1";}
 if (sostituzione == "A348K") {valore_pssm = "-1";}
 if (sostituzione == "K393R") {valore_pssm = "-1";}
 if (sostituzione == "V413I") {valore_pssm = "-1";}
 if (sostituzione == "M70R") {valore_pssm = "-1";}
 if (sostituzione == "L75M") {valore_pssm = "-1";}
 if (sostituzione == "P98L") {valore_pssm = "-1";}
 if (sostituzione == "E103S") {valore_pssm = "-1";}
 if (sostituzione == "R118E") {valore_pssm = "-1";}
 if (sostituzione == "N179K") {valore_pssm = "-1";}
 if (sostituzione == "L189M") {valore_pssm = "-1";}
 if (sostituzione == "M208C") {valore_pssm = "-1";}
 if (sostituzione == "N215T") {valore_pssm = "-1";}
 if (sostituzione == "S247L") {valore_pssm = "-1";}
 if (sostituzione == "D255G") {valore_pssm = "-1";}
 if (sostituzione == "L286V") {valore_pssm = "-1";}
 if (sostituzione == "S371E") {valore_pssm = "-1";}
 if (sostituzione == "S401A") {valore_pssm = "-1";}
 if (sostituzione == "R17I") {valore_pssm = "-1";}
 if (sostituzione == "A29T") {valore_pssm = "-1";}
 if (sostituzione == "H46A") {valore_pssm = "-1";}
 if (sostituzione == "H46N") {valore_pssm = "-1";}
 if (sostituzione == "D61A") {valore_pssm = "-1";}
 if (sostituzione == "L75F") {valore_pssm = "-1";}
 if (sostituzione == "E87D") {valore_pssm = "-1";}
 if (sostituzione == "L106F") {valore_pssm = "-1";}
 if (sostituzione == "Y222T") {valore_pssm = "-1";}
 if (sostituzione == "S247C") {valore_pssm = "-1";}
 if (sostituzione == "D255I") {valore_pssm = "-1";}
 if (sostituzione == "M290I") {valore_pssm = "-1";}
 if (sostituzione == "D335S") {valore_pssm = "-1";}
 if (sostituzione == "L347F") {valore_pssm = "-1";}
 if (sostituzione == "T41H") {valore_pssm = "-1";}
 if (sostituzione == "D61P") {valore_pssm = "-1";}
 if (sostituzione == "I64V") {valore_pssm = "-1";}
 if (sostituzione == "Q107Y") {valore_pssm = "-1";}
 if (sostituzione == "A135H") {valore_pssm = "-1";}
 if (sostituzione == "E178R") {valore_pssm = "-1";}
 if (sostituzione == "L180S") {valore_pssm = "-1";}
 if (sostituzione == "M208D") {valore_pssm = "-1";}
 if (sostituzione == "R220V") {valore_pssm = "-1";}
 if (sostituzione == "D234C") {valore_pssm = "-1";}
 if (sostituzione == "A257Q") {valore_pssm = "-1";}
 if (sostituzione == "I289L") {valore_pssm = "-1";}
 if (sostituzione == "D315H") {valore_pssm = "-1";}
 if (sostituzione == "N336F") {valore_pssm = "-1";}
 if (sostituzione == "L8M") {valore_pssm = "-1";}
 if (sostituzione == "A13H") {valore_pssm = "-1";}
 if (sostituzione == "T41R") {valore_pssm = "-1";}
 if (sostituzione == "L45S") {valore_pssm = "-1";}
 if (sostituzione == "M51A") {valore_pssm = "-1";}
 if (sostituzione == "Q57D") {valore_pssm = "-1";}
 if (sostituzione == "Y173H") {valore_pssm = "-1";}
 if (sostituzione == "L177F") {valore_pssm = "-1";}
 if (sostituzione == "Y216F") {valore_pssm = "-1";}
 if (sostituzione == "D315I") {valore_pssm = "-1";}
 if (sostituzione == "Q330I") {valore_pssm = "-1";}
 if (sostituzione == "N336I") {valore_pssm = "-1";}
 if (sostituzione == "S401F") {valore_pssm = "-1";}
 if (sostituzione == "G11I") {valore_pssm = "-1";}
 if (sostituzione == "A37V") {valore_pssm = "-1";}
 if (sostituzione == "S62M") {valore_pssm = "-1";}
 if (sostituzione == "Q99N") {valore_pssm = "-1";}
 if (sostituzione == "R118Q") {valore_pssm = "-1";}
 if (sostituzione == "Q119I") {valore_pssm = "-1";}
 if (sostituzione == "Q119R") {valore_pssm = "-1";}
 if (sostituzione == "N139R") {valore_pssm = "-1";}
 if (sostituzione == "L167V") {valore_pssm = "-1";}
 if (sostituzione == "L177S") {valore_pssm = "-1";}
 if (sostituzione == "L189G") {valore_pssm = "-1";}
 if (sostituzione == "S197K") {valore_pssm = "-1";}
 if (sostituzione == "K240F") {valore_pssm = "-1";}
 if (sostituzione == "N249I") {valore_pssm = "-1";}
 if (sostituzione == "I367S") {valore_pssm = "-1";}
 if (sostituzione == "A368V") {valore_pssm = "-1";}
 if (sostituzione == "S401M") {valore_pssm = "-1";}
 if (sostituzione == "Q2R") {valore_pssm = "-1";}
 if (sostituzione == "R4L") {valore_pssm = "-1";}
 if (sostituzione == "N5T") {valore_pssm = "-1";}
 if (sostituzione == "L21I") {valore_pssm = "-1";}
 if (sostituzione == "L75G") {valore_pssm = "-1";}
 if (sostituzione == "S102D") {valore_pssm = "-1";}
 if (sostituzione == "V164I") {valore_pssm = "-1";}
 if (sostituzione == "L189F") {valore_pssm = "-1";}
 if (sostituzione == "M208P") {valore_pssm = "-1";}
 if (sostituzione == "T246I") {valore_pssm = "-1";}
 if (sostituzione == "V254K") {valore_pssm = "-1";}
 if (sostituzione == "G261Y") {valore_pssm = "-1";}
 if (sostituzione == "W277I") {valore_pssm = "-1";}
 if (sostituzione == "M296V") {valore_pssm = "-1";}
 if (sostituzione == "L372I") {valore_pssm = "-1";}
 if (sostituzione == "S405A") {valore_pssm = "-1";}
 if (sostituzione == "L415M") {valore_pssm = "-1";}
 if (sostituzione == "S23T") {valore_pssm = "-1";}
 if (sostituzione == "F69I") {valore_pssm = "-1";}
 if (sostituzione == "I91L") {valore_pssm = "-1";}
 if (sostituzione == "A97S") {valore_pssm = "-1";}
 if (sostituzione == "V137I") {valore_pssm = "-1";}
 if (sostituzione == "S197E") {valore_pssm = "-1";}
 if (sostituzione == "T246K") {valore_pssm = "-1";}
 if (sostituzione == "T246E") {valore_pssm = "-1";}
 if (sostituzione == "D255F") {valore_pssm = "-1";}
 if (sostituzione == "V256I") {valore_pssm = "-1";}
 if (sostituzione == "E341V") {valore_pssm = "-1";}
 if (sostituzione == "L8F") {valore_pssm = "-1";}
 if (sostituzione == "A13S") {valore_pssm = "-1";}
 if (sostituzione == "L16I") {valore_pssm = "-1";}
 if (sostituzione == "I26L") {valore_pssm = "-1";}
 if (sostituzione == "E59H") {valore_pssm = "-1";}
 if (sostituzione == "K140R") {valore_pssm = "-1";}
 if (sostituzione == "Y151R") {valore_pssm = "-1";}
 if (sostituzione == "Y152I") {valore_pssm = "-1";}
 if (sostituzione == "I154V") {valore_pssm = "-1";}
 if (sostituzione == "S176V") {valore_pssm = "-1";}
 if (sostituzione == "L177M") {valore_pssm = "-1";}
 if (sostituzione == "L206Y") {valore_pssm = "-1";}
 if (sostituzione == "E251Q") {valore_pssm = "-1";}
 if (sostituzione == "S371H") {valore_pssm = "-1";}
 if (sostituzione == "S405V") {valore_pssm = "-1";}
 if (sostituzione == "Q57S") {valore_pssm = "-1";}
 if (sostituzione == "E59K") {valore_pssm = "-1";}
 if (sostituzione == "S78N") {valore_pssm = "-1";}
 if (sostituzione == "R105N") {valore_pssm = "-1";}
 if (sostituzione == "Q111D") {valore_pssm = "-1";}
 if (sostituzione == "D153K") {valore_pssm = "-1";}
 if (sostituzione == "R196H") {valore_pssm = "-1";}
 if (sostituzione == "N228M") {valore_pssm = "-1";}
 if (sostituzione == "D255C") {valore_pssm = "-1";}
 if (sostituzione == "L275V") {valore_pssm = "-1";}
 if (sostituzione == "W277K") {valore_pssm = "-1";}
 if (sostituzione == "D315T") {valore_pssm = "-1";}
 if (sostituzione == "W340Y") {valore_pssm = "-1";}
 if (sostituzione == "L347M") {valore_pssm = "-1";}
 if (sostituzione == "R363H") {valore_pssm = "-1";}
 if (sostituzione == "L372V") {valore_pssm = "-1";}
 if (sostituzione == "G144K") {valore_pssm = "-1";}
 if (sostituzione == "H186R") {valore_pssm = "-1";}
 if (sostituzione == "K213R") {valore_pssm = "-1";}
 if (sostituzione == "N215Q") {valore_pssm = "-1";}
 if (sostituzione == "D233H") {valore_pssm = "-1";}
 if (sostituzione == "R252H") {valore_pssm = "-1";}
 if (sostituzione == "K314A") {valore_pssm = "-1";}
 if (sostituzione == "D315R") {valore_pssm = "-1";}
 if (sostituzione == "D335G") {valore_pssm = "-1";}
 if (sostituzione == "Q2E") {valore_pssm = "-1";}
 if (sostituzione == "H9R") {valore_pssm = "-1";}
 if (sostituzione == "A13V") {valore_pssm = "-1";}
 if (sostituzione == "S23Y") {valore_pssm = "-1";}
 if (sostituzione == "W24T") {valore_pssm = "-1";}
 if (sostituzione == "L54F") {valore_pssm = "-1";}
 if (sostituzione == "Y222L") {valore_pssm = "-1";}
 if (sostituzione == "E251H") {valore_pssm = "-1";}
 if (sostituzione == "R252T") {valore_pssm = "-1";}
 if (sostituzione == "L275I") {valore_pssm = "-1";}
 if (sostituzione == "V281Q") {valore_pssm = "-1";}
 if (sostituzione == "D313G") {valore_pssm = "-1";}
 if (sostituzione == "S364N") {valore_pssm = "-1";}
 if (sostituzione == "H406R") {valore_pssm = "-1";}
 if (sostituzione == "F18P") {valore_pssm = "-1";}
 if (sostituzione == "D175R") {valore_pssm = "-1";}
 if (sostituzione == "L189P") {valore_pssm = "-1";}
 if (sostituzione == "L206N") {valore_pssm = "-1";}
 if (sostituzione == "F211W") {valore_pssm = "-1";}
 if (sostituzione == "Q212G") {valore_pssm = "-1";}
 if (sostituzione == "T246R") {valore_pssm = "-1";}
 if (sostituzione == "A291R") {valore_pssm = "-1";}
 if (sostituzione == "S364Y") {valore_pssm = "-1";}
 if (sostituzione == "D25E") {valore_pssm = "-1";}
 if (sostituzione == "D61N") {valore_pssm = "-1";}
 if (sostituzione == "M96V") {valore_pssm = "-1";}
 if (sostituzione == "I133M") {valore_pssm = "-1";}
 if (sostituzione == "K140V") {valore_pssm = "-1";}
 if (sostituzione == "G163K") {valore_pssm = "-1";}
 if (sostituzione == "S176R") {valore_pssm = "-1";}
 if (sostituzione == "S197A") {valore_pssm = "-1";}
 if (sostituzione == "S197H") {valore_pssm = "-1";}
 if (sostituzione == "F229S") {valore_pssm = "-1";}
 if (sostituzione == "K237G") {valore_pssm = "-1";}
 if (sostituzione == "V254S") {valore_pssm = "-1";}
 if (sostituzione == "L268M") {valore_pssm = "-1";}
 if (sostituzione == "K326L") {valore_pssm = "-1";}
 if (sostituzione == "S345A") {valore_pssm = "-1";}
 if (sostituzione == "Q57T") {valore_pssm = "-1";}
 if (sostituzione == "D61G") {valore_pssm = "-1";}
 if (sostituzione == "D175Q") {valore_pssm = "-1";}
 if (sostituzione == "A190V") {valore_pssm = "-1";}
 if (sostituzione == "V199Y") {valore_pssm = "-1";}
 if (sostituzione == "S235A") {valore_pssm = "-1";}
 if (sostituzione == "W245K") {valore_pssm = "-1";}
 if (sostituzione == "T246S") {valore_pssm = "-1";}
 if (sostituzione == "A291N") {valore_pssm = "-1";}
 if (sostituzione == "A307R") {valore_pssm = "-1";}
 if (sostituzione == "V316L") {valore_pssm = "-1";}
 if (sostituzione == "E338Q") {valore_pssm = "-1";}
 if (sostituzione == "K426R") {valore_pssm = "-1";}
 if (sostituzione == "S23V") {valore_pssm = "-1";}
 if (sostituzione == "P27I") {valore_pssm = "-1";}
 if (sostituzione == "E59Q") {valore_pssm = "-1";}
 if (sostituzione == "S65T") {valore_pssm = "-1";}
 if (sostituzione == "W81M") {valore_pssm = "-1";}
 if (sostituzione == "I154L") {valore_pssm = "-1";}
 if (sostituzione == "D161K") {valore_pssm = "-1";}
 if (sostituzione == "C174S") {valore_pssm = "-1";}
 if (sostituzione == "W209R") {valore_pssm = "-1";}
 if (sostituzione == "D233A") {valore_pssm = "-1";}
 if (sostituzione == "W277Q") {valore_pssm = "-1";}
 if (sostituzione == "P305K") {valore_pssm = "-1";}
 if (sostituzione == "A309H") {valore_pssm = "-1";}
 if (sostituzione == "Q330T") {valore_pssm = "-1";}
 if (sostituzione == "M353T") {valore_pssm = "-1";}
 if (sostituzione == "L425M") {valore_pssm = "-1";}
 if (sostituzione == "L428M") {valore_pssm = "-1";}
 if (sostituzione == "L429M") {valore_pssm = "-1";}
 if (sostituzione == "L32V") {valore_pssm = "-1";}
 if (sostituzione == "D61C") {valore_pssm = "-1";}
 if (sostituzione == "S65H") {valore_pssm = "-1";}
 if (sostituzione == "K67H") {valore_pssm = "-1";}
 if (sostituzione == "L120I") {valore_pssm = "-1";}
 if (sostituzione == "L131V") {valore_pssm = "-1";}
 if (sostituzione == "A135V") {valore_pssm = "-1";}
 if (sostituzione == "D153A") {valore_pssm = "-1";}
 if (sostituzione == "I154N") {valore_pssm = "-1";}
 if (sostituzione == "D161Q") {valore_pssm = "-1";}
 if (sostituzione == "D175H") {valore_pssm = "-1";}
 if (sostituzione == "H186K") {valore_pssm = "-1";}
 if (sostituzione == "D233R") {valore_pssm = "-1";}
 if (sostituzione == "E251T") {valore_pssm = "-1";}
 if (sostituzione == "N278P") {valore_pssm = "-1";}
 if (sostituzione == "I303L") {valore_pssm = "-1";}
 if (sostituzione == "Q306E") {valore_pssm = "-1";}
 if (sostituzione == "K308L") {valore_pssm = "-1";}
 if (sostituzione == "K308F") {valore_pssm = "-1";}
 if (sostituzione == "A309T") {valore_pssm = "-1";}
 if (sostituzione == "L311Y") {valore_pssm = "-1";}
 if (sostituzione == "A381S") {valore_pssm = "-1";}
 if (sostituzione == "L45H") {valore_pssm = "-1";}
 if (sostituzione == "L75P") {valore_pssm = "-1";}
 if (sostituzione == "L106M") {valore_pssm = "-1";}
 if (sostituzione == "C174A") {valore_pssm = "-1";}
 if (sostituzione == "A181G") {valore_pssm = "-1";}
 if (sostituzione == "D182A") {valore_pssm = "-1";}
 if (sostituzione == "G183K") {valore_pssm = "-1";}
 if (sostituzione == "L189C") {valore_pssm = "-1";}
 if (sostituzione == "K240R") {valore_pssm = "-1";}
 if (sostituzione == "S247Y") {valore_pssm = "-1";}
 if (sostituzione == "F248L") {valore_pssm = "-1";}
 if (sostituzione == "F248R") {valore_pssm = "-1";}
 if (sostituzione == "G334T") {valore_pssm = "-1";}
 if (sostituzione == "S364V") {valore_pssm = "-1";}
 if (sostituzione == "S424A") {valore_pssm = "-1";}
 if (sostituzione == "L429H") {valore_pssm = "-1";}
 if (sostituzione == "N5H") {valore_pssm = "-1";}
 if (sostituzione == "L8S") {valore_pssm = "-1";}
 if (sostituzione == "L14M") {valore_pssm = "-1";}
 if (sostituzione == "D33K") {valore_pssm = "-1";}
 if (sostituzione == "S78H") {valore_pssm = "-1";}
 if (sostituzione == "V124I") {valore_pssm = "-1";}
 if (sostituzione == "A135R") {valore_pssm = "-1";}
 if (sostituzione == "I154E") {valore_pssm = "-1";}
 if (sostituzione == "L166T") {valore_pssm = "-1";}
 if (sostituzione == "E251R") {valore_pssm = "-1";}
 if (sostituzione == "N278G") {valore_pssm = "-1";}
 if (sostituzione == "S304K") {valore_pssm = "-1";}
 if (sostituzione == "A309L") {valore_pssm = "-1";}
 if (sostituzione == "L310M") {valore_pssm = "-1";}
 if (sostituzione == "K326R") {valore_pssm = "-1";}
 if (sostituzione == "Q330M") {valore_pssm = "-1";}
 if (sostituzione == "E341Q") {valore_pssm = "-1";}
 if (sostituzione == "L347P") {valore_pssm = "-1";}
 if (sostituzione == "A13R") {valore_pssm = "-1";}
 if (sostituzione == "L14F") {valore_pssm = "-1";}
 if (sostituzione == "P98V") {valore_pssm = "-1";}
 if (sostituzione == "D161T") {valore_pssm = "-1";}
 if (sostituzione == "N179H") {valore_pssm = "-1";}
 if (sostituzione == "A181H") {valore_pssm = "-1";}
 if (sostituzione == "S247P") {valore_pssm = "-1";}
 if (sostituzione == "E251N") {valore_pssm = "-1";}
 if (sostituzione == "V254H") {valore_pssm = "-1";}
 if (sostituzione == "Q280A") {valore_pssm = "-1";}
 if (sostituzione == "Q280R") {valore_pssm = "-1";}
 if (sostituzione == "D315S") {valore_pssm = "-1";}
 if (sostituzione == "S405K") {valore_pssm = "-1";}
 if (sostituzione == "D33H") {valore_pssm = "-1";}
 if (sostituzione == "L45V") {valore_pssm = "-1";}
 if (sostituzione == "D61L") {valore_pssm = "-1";}
 if (sostituzione == "N122T") {valore_pssm = "-1";}
 if (sostituzione == "I133V") {valore_pssm = "-1";}
 if (sostituzione == "S197Q") {valore_pssm = "-1";}
 if (sostituzione == "M208G") {valore_pssm = "-1";}
 if (sostituzione == "T246Q") {valore_pssm = "-1";}
 if (sostituzione == "V254R") {valore_pssm = "-1";}
 if (sostituzione == "I270P") {valore_pssm = "-1";}
 if (sostituzione == "N298S") {valore_pssm = "-1";}
 if (sostituzione == "F337M") {valore_pssm = "-1";}
 if (sostituzione == "L347C") {valore_pssm = "-1";}
 if (sostituzione == "S371A") {valore_pssm = "-1";}
 if (sostituzione == "S401K") {valore_pssm = "-1";}
 if (sostituzione == "S401H") {valore_pssm = "-1";}
 if (sostituzione == "S405E") {valore_pssm = "-1";}
 if (sostituzione == "Q416E") {valore_pssm = "-1";}
 if (sostituzione == "L425F") {valore_pssm = "-1";}
 if (sostituzione == "L428F") {valore_pssm = "-1";}
 if (sostituzione == "L429F") {valore_pssm = "-1";}
 if (sostituzione == "R30I") {valore_pssm = "-1";}
 if (sostituzione == "E59V") {valore_pssm = "-1";}
 if (sostituzione == "E59R") {valore_pssm = "-1";}
 if (sostituzione == "S78T") {valore_pssm = "-1";}
 if (sostituzione == "S78R") {valore_pssm = "-1";}
 if (sostituzione == "Q107M") {valore_pssm = "-1";}
 if (sostituzione == "Q119L") {valore_pssm = "-1";}
 if (sostituzione == "V137T") {valore_pssm = "-1";}
 if (sostituzione == "F149I") {valore_pssm = "-1";}
 if (sostituzione == "L180T") {valore_pssm = "-1";}
 if (sostituzione == "R193N") {valore_pssm = "-1";}
 if (sostituzione == "Q221V") {valore_pssm = "-1";}
 if (sostituzione == "A292S") {valore_pssm = "-1";}
 if (sostituzione == "A309V") {valore_pssm = "-1";}
 if (sostituzione == "K326E") {valore_pssm = "-1";}
 if (sostituzione == "L331M") {valore_pssm = "-1";}
 if (sostituzione == "R332E") {valore_pssm = "-1";}
 if (sostituzione == "G334Y") {valore_pssm = "-1";}
 if (sostituzione == "L3M") {valore_pssm = "-1";}
 if (sostituzione == "L10M") {valore_pssm = "-1";}
 if (sostituzione == "A13T") {valore_pssm = "-1";}
 if (sostituzione == "S23H") {valore_pssm = "-1";}
 if (sostituzione == "L32I") {valore_pssm = "-1";}
 if (sostituzione == "R38L") {valore_pssm = "-1";}
 if (sostituzione == "D61Y") {valore_pssm = "-1";}
 if (sostituzione == "N122S") {valore_pssm = "-1";}
 if (sostituzione == "K127L") {valore_pssm = "-1";}
 if (sostituzione == "L166Q") {valore_pssm = "-1";}
 if (sostituzione == "I242V") {valore_pssm = "-1";}
 if (sostituzione == "S247M") {valore_pssm = "-1";}
 if (sostituzione == "N249D") {valore_pssm = "-1";}
 if (sostituzione == "V254I") {valore_pssm = "-1";}
 if (sostituzione == "I303V") {valore_pssm = "-1";}
 if (sostituzione == "Q333A") {valore_pssm = "-1";}
 if (sostituzione == "V351L") {valore_pssm = "-1";}
 if (sostituzione == "L387M") {valore_pssm = "-1";}
 if (sostituzione == "L388M") {valore_pssm = "-1";}
 if (sostituzione == "V390Q") {valore_pssm = "-1";}
 if (sostituzione == "L394M") {valore_pssm = "-1";}
 if (sostituzione == "Y397F") {valore_pssm = "-1";}
 if (sostituzione == "S401E") {valore_pssm = "-1";}
 if (sostituzione == "L403M") {valore_pssm = "-1";}
 if (sostituzione == "L414M") {valore_pssm = "-1";}
 if (sostituzione == "L417M") {valore_pssm = "-1";}
 if (sostituzione == "M421L") {valore_pssm = "-1";}
 if (sostituzione == "M423L") {valore_pssm = "-1";}
 if (sostituzione == "T41S") {valore_pssm = "-1";}
 if (sostituzione == "C90V") {valore_pssm = "-1";}
 if (sostituzione == "K127M") {valore_pssm = "-1";}
 if (sostituzione == "K140I") {valore_pssm = "-1";}
 if (sostituzione == "L180F") {valore_pssm = "-1";}
 if (sostituzione == "W209A") {valore_pssm = "-1";}
 if (sostituzione == "A257S") {valore_pssm = "-1";}
 if (sostituzione == "N298V") {valore_pssm = "-1";}
 if (sostituzione == "K391R") {valore_pssm = "-1";}
 if (sostituzione == "L54M") {valore_pssm = "-1";}
 if (sostituzione == "Q57I") {valore_pssm = "-1";}
 if (sostituzione == "L75N") {valore_pssm = "-1";}
 if (sostituzione == "Q119C") {valore_pssm = "-1";}
 if (sostituzione == "S126N") {valore_pssm = "-1";}
 if (sostituzione == "K127R") {valore_pssm = "-1";}
 if (sostituzione == "Y152R") {valore_pssm = "-1";}
 if (sostituzione == "L166R") {valore_pssm = "-1";}
 if (sostituzione == "L191M") {valore_pssm = "-1";}
 if (sostituzione == "P210H") {valore_pssm = "-1";}
 if (sostituzione == "E218L") {valore_pssm = "-1";}
 if (sostituzione == "F248K") {valore_pssm = "-1";}
 if (sostituzione == "W277S") {valore_pssm = "-1";}
 if (sostituzione == "N278I") {valore_pssm = "-1";}
 if (sostituzione == "A309R") {valore_pssm = "-1";}
 if (sostituzione == "Q330F") {valore_pssm = "-1";}
 if (sostituzione == "E341R") {valore_pssm = "-1";}
 if (sostituzione == "A381V") {valore_pssm = "-1";}
 if (sostituzione == "K393Q") {valore_pssm = "-1";}
 if (sostituzione == "M421I") {valore_pssm = "-1";}
 if (sostituzione == "M423I") {valore_pssm = "-1";}
 if (sostituzione == "A37I") {valore_pssm = "-1";}
 if (sostituzione == "Y152F") {valore_pssm = "-1";}
 if (sostituzione == "L166K") {valore_pssm = "-1";}
 if (sostituzione == "D255Y") {valore_pssm = "-1";}
 if (sostituzione == "V269Q") {valore_pssm = "-1";}
 if (sostituzione == "W277R") {valore_pssm = "-1";}
 if (sostituzione == "F383V") {valore_pssm = "-1";}
 if (sostituzione == "S401Q") {valore_pssm = "-1";}
 if (sostituzione == "D427E") {valore_pssm = "-1";}
 if (sostituzione == "L3F") {valore_pssm = "-1";}
 if (sostituzione == "L10F") {valore_pssm = "-1";}
 if (sostituzione == "L19I") {valore_pssm = "-1";}
 if (sostituzione == "L45I") {valore_pssm = "-1";}
 if (sostituzione == "M51Y") {valore_pssm = "-1";}
 if (sostituzione == "S65E") {valore_pssm = "-1";}
 if (sostituzione == "Q119M") {valore_pssm = "-1";}
 if (sostituzione == "A143V") {valore_pssm = "-1";}
 if (sostituzione == "M208N") {valore_pssm = "-1";}
 if (sostituzione == "Q221N") {valore_pssm = "-1";}
 if (sostituzione == "H225L") {valore_pssm = "-1";}
 if (sostituzione == "F248E") {valore_pssm = "-1";}
 if (sostituzione == "D255L") {valore_pssm = "-1";}
 if (sostituzione == "H302E") {valore_pssm = "-1";}
 if (sostituzione == "D335K") {valore_pssm = "-1";}
 if (sostituzione == "L387F") {valore_pssm = "-1";}
 if (sostituzione == "L388F") {valore_pssm = "-1";}
 if (sostituzione == "K393E") {valore_pssm = "-1";}
 if (sostituzione == "L394F") {valore_pssm = "-1";}
 if (sostituzione == "L403F") {valore_pssm = "-1";}
 if (sostituzione == "L414F") {valore_pssm = "-1";}
 if (sostituzione == "L417F") {valore_pssm = "-1";}
 if (sostituzione == "A13D") {valore_pssm = "-1";}
 if (sostituzione == "R38P") {valore_pssm = "-1";}
 if (sostituzione == "D55E") {valore_pssm = "-1";}
 if (sostituzione == "L75Y") {valore_pssm = "-1";}
 if (sostituzione == "A84F") {valore_pssm = "-1";}
 if (sostituzione == "L89M") {valore_pssm = "-1";}
 if (sostituzione == "L89F") {valore_pssm = "-1";}
 if (sostituzione == "I91M") {valore_pssm = "-1";}
 if (sostituzione == "E103H") {valore_pssm = "-1";}
 if (sostituzione == "F149V") {valore_pssm = "-1";}
 if (sostituzione == "L189Y") {valore_pssm = "-1";}
 if (sostituzione == "E203D") {valore_pssm = "-1";}
 if (sostituzione == "K240Q") {valore_pssm = "-1";}
 if (sostituzione == "Q250N") {valore_pssm = "-1";}
 if (sostituzione == "S405H") {valore_pssm = "-1";}
 if (sostituzione == "M421V") {valore_pssm = "-1";}
 if (sostituzione == "M423V") {valore_pssm = "-1";}
 if (sostituzione == "R17K") {valore_pssm = "-1";}
 if (sostituzione == "G28T") {valore_pssm = "-1";}
 if (sostituzione == "I91V") {valore_pssm = "-1";}
 if (sostituzione == "L106I") {valore_pssm = "-1";}
 if (sostituzione == "D153H") {valore_pssm = "-1";}
 if (sostituzione == "Q157E") {valore_pssm = "-1";}
 if (sostituzione == "R193E") {valore_pssm = "-1";}
 if (sostituzione == "W236F") {valore_pssm = "-1";}
 if (sostituzione == "K237Q") {valore_pssm = "-1";}
 if (sostituzione == "L243F") {valore_pssm = "-1";}
 if (sostituzione == "D244S") {valore_pssm = "-1";}
 if (sostituzione == "S247G") {valore_pssm = "-1";}
 if (sostituzione == "D315F") {valore_pssm = "-1";}
 if (sostituzione == "K326M") {valore_pssm = "-1";}
 if (sostituzione == "N336M") {valore_pssm = "-1";}
 if (sostituzione == "V351I") {valore_pssm = "-1";}
 if (sostituzione == "A368G") {valore_pssm = "-1";}
 if (sostituzione == "A368N") {valore_pssm = "-1";}
 if (sostituzione == "R402K") {valore_pssm = "-1";}
 if (sostituzione == "S405Q") {valore_pssm = "-1";}
 if (sostituzione == "N5K") {valore_pssm = "-1";}
 if (sostituzione == "N5E") {valore_pssm = "-1";}
 if (sostituzione == "L19V") {valore_pssm = "-1";}
 if (sostituzione == "R38Q") {valore_pssm = "-1";}
 if (sostituzione == "S65R") {valore_pssm = "-1";}
 if (sostituzione == "S126D") {valore_pssm = "-1";}
 if (sostituzione == "K185Q") {valore_pssm = "-1";}
 if (sostituzione == "A230C") {valore_pssm = "-1";}
 if (sostituzione == "T246L") {valore_pssm = "-1";}
 if (sostituzione == "N249C") {valore_pssm = "-1";}
 if (sostituzione == "R252A") {valore_pssm = "-1";}
 if (sostituzione == "D255M") {valore_pssm = "-1";}
 if (sostituzione == "Q330S") {valore_pssm = "-1";}
 if (sostituzione == "Q333F") {valore_pssm = "-1";}
 if (sostituzione == "N336P") {valore_pssm = "-1";}
 if (sostituzione == "R356K") {valore_pssm = "-1";}
 if (sostituzione == "S401C") {valore_pssm = "-1";}
 if (sostituzione == "S401R") {valore_pssm = "-1";}
 if (sostituzione == "E87H") {valore_pssm = "-1";}
 if (sostituzione == "E87R") {valore_pssm = "-1";}
 if (sostituzione == "P98E") {valore_pssm = "-1";}
 if (sostituzione == "R118H") {valore_pssm = "-1";}
 if (sostituzione == "I154K") {valore_pssm = "-1";}
 if (sostituzione == "L180K") {valore_pssm = "-1";}
 if (sostituzione == "I354H") {valore_pssm = "-1";}
 if (sostituzione == "R392I") {valore_pssm = "-1";}
 if (sostituzione == "W44F") {valore_pssm = "-1";}
 if (sostituzione == "E59T") {valore_pssm = "-1";}
 if (sostituzione == "P98R") {valore_pssm = "-1";}
 if (sostituzione == "A135D") {valore_pssm = "-1";}
 if (sostituzione == "Y152D") {valore_pssm = "-1";}
 if (sostituzione == "L177C") {valore_pssm = "-1";}
 if (sostituzione == "D182V") {valore_pssm = "-1";}
 if (sostituzione == "R193Q") {valore_pssm = "-1";}
 if (sostituzione == "E203N") {valore_pssm = "-1";}
 if (sostituzione == "N249G") {valore_pssm = "-1";}
 if (sostituzione == "E251V") {valore_pssm = "-1";}
 if (sostituzione == "Q280T") {valore_pssm = "-1";}
 if (sostituzione == "A291E") {valore_pssm = "-1";}
 if (sostituzione == "N336C") {valore_pssm = "-1";}
 if (sostituzione == "F337H") {valore_pssm = "-1";}
 if (sostituzione == "G346A") {valore_pssm = "-1";}
 if (sostituzione == "L347Y") {valore_pssm = "-1";}
 if (sostituzione == "Q357K") {valore_pssm = "-1";}
 if (sostituzione == "V369M") {valore_pssm = "-1";}
 if (sostituzione == "L45T") {valore_pssm = "-1";}
 if (sostituzione == "Q57F") {valore_pssm = "-1";}
 if (sostituzione == "S65Q") {valore_pssm = "-1";}
 if (sostituzione == "E103R") {valore_pssm = "-1";}
 if (sostituzione == "Q119G") {valore_pssm = "-1";}
 if (sostituzione == "D161H") {valore_pssm = "-1";}
 if (sostituzione == "D175V") {valore_pssm = "-1";}
 if (sostituzione == "N179V") {valore_pssm = "-1";}
 if (sostituzione == "P210A") {valore_pssm = "-1";}
 if (sostituzione == "P210K") {valore_pssm = "-1";}
 if (sostituzione == "T246C") {valore_pssm = "-1";}
 if (sostituzione == "I253F") {valore_pssm = "-1";}
 if (sostituzione == "N278C") {valore_pssm = "-1";}
 if (sostituzione == "A288S") {valore_pssm = "-1";}
 if (sostituzione == "P305D") {valore_pssm = "-1";}
 if (sostituzione == "G328A") {valore_pssm = "-1";}
 if (sostituzione == "Y329H") {valore_pssm = "-1";}
 if (sostituzione == "Q333V") {valore_pssm = "-1";}
 if (sostituzione == "E341H") {valore_pssm = "-1";}
 if (sostituzione == "A348E") {valore_pssm = "-1";}
 if (sostituzione == "Q111R") {valore_pssm = "-1";}
 if (sostituzione == "L180M") {valore_pssm = "-1";}
 if (sostituzione == "A181V") {valore_pssm = "-1";}
 if (sostituzione == "S197V") {valore_pssm = "-1";}
 if (sostituzione == "F211V") {valore_pssm = "-1";}
 if (sostituzione == "S247N") {valore_pssm = "-1";}
 if (sostituzione == "A318S") {valore_pssm = "-1";}
 if (sostituzione == "A318Q") {valore_pssm = "-1";}
 if (sostituzione == "L331T") {valore_pssm = "-1";}
 if (sostituzione == "I359L") {valore_pssm = "-1";}
 if (sostituzione == "A368C") {valore_pssm = "-1";}
 if (sostituzione == "A377V") {valore_pssm = "-1";}
 if (sostituzione == "Q422E") {valore_pssm = "-1";}
 if (sostituzione == "S23K") {valore_pssm = "-1";}
 if (sostituzione == "L54T") {valore_pssm = "-1";}
 if (sostituzione == "Q57A") {valore_pssm = "-1";}
 if (sostituzione == "M96S") {valore_pssm = "-1";}
 if (sostituzione == "P98T") {valore_pssm = "-1";}
 if (sostituzione == "D101S") {valore_pssm = "-1";}
 if (sostituzione == "R118S") {valore_pssm = "-1";}
 if (sostituzione == "F169E") {valore_pssm = "-1";}
 if (sostituzione == "L177N") {valore_pssm = "-1";}
 if (sostituzione == "N249P") {valore_pssm = "-1";}
 if (sostituzione == "R252S") {valore_pssm = "-1";}
 if (sostituzione == "A257G") {valore_pssm = "-1";}
 if (sostituzione == "L324V") {valore_pssm = "-1";}
 if (sostituzione == "M353C") {valore_pssm = "-1";}
 if (sostituzione == "N355S") {valore_pssm = "-1";}
 if (sostituzione == "G360E") {valore_pssm = "-1";}
 if (sostituzione == "R363K") {valore_pssm = "-1";}
 if (sostituzione == "K374Q") {valore_pssm = "-1";}
 if (sostituzione == "L429K") {valore_pssm = "-1";}
 if (sostituzione == "L429E") {valore_pssm = "-1";}
 if (sostituzione == "D25S") {valore_pssm = "-1";}
 if (sostituzione == "T41A") {valore_pssm = "-1";}
 if (sostituzione == "T41V") {valore_pssm = "-1";}
 if (sostituzione == "M51K") {valore_pssm = "-1";}
 if (sostituzione == "E59S") {valore_pssm = "-1";}
 if (sostituzione == "K82Q") {valore_pssm = "-1";}
 if (sostituzione == "D83E") {valore_pssm = "-1";}
 if (sostituzione == "Q107L") {valore_pssm = "-1";}
 if (sostituzione == "L180H") {valore_pssm = "-1";}
 if (sostituzione == "D182P") {valore_pssm = "-1";}
 if (sostituzione == "S188G") {valore_pssm = "-1";}
 if (sostituzione == "S197R") {valore_pssm = "-1";}
 if (sostituzione == "I219M") {valore_pssm = "-1";}
 if (sostituzione == "I219F") {valore_pssm = "-1";}
 if (sostituzione == "V256A") {valore_pssm = "-1";}
 if (sostituzione == "G258R") {valore_pssm = "-1";}
 if (sostituzione == "A291Q") {valore_pssm = "-1";}
 if (sostituzione == "D313V") {valore_pssm = "-1";}
 if (sostituzione == "E341I") {valore_pssm = "-1";}
 if (sostituzione == "N5Q") {valore_pssm = "-1";}
 if (sostituzione == "L8K") {valore_pssm = "-1";}
 if (sostituzione == "S23E") {valore_pssm = "-1";}
 if (sostituzione == "L45E") {valore_pssm = "-1";}
 if (sostituzione == "R49K") {valore_pssm = "-1";}
 if (sostituzione == "D61M") {valore_pssm = "-1";}
 if (sostituzione == "M76L") {valore_pssm = "-1";}
 if (sostituzione == "C90I") {valore_pssm = "-1";}
 if (sostituzione == "P98Q") {valore_pssm = "-1";}
 if (sostituzione == "R118T") {valore_pssm = "-1";}
 if (sostituzione == "L129I") {valore_pssm = "-1";}
 if (sostituzione == "L167I") {valore_pssm = "-1";}
 if (sostituzione == "R252L") {valore_pssm = "-1";}
 if (sostituzione == "Y329A") {valore_pssm = "-1";}
 if (sostituzione == "D335E") {valore_pssm = "-1";}
 if (sostituzione == "W349T") {valore_pssm = "-1";}
 if (sostituzione == "L372M") {valore_pssm = "-1";}
 if (sostituzione == "I407L") {valore_pssm = "-1";}
 if (sostituzione == "L8A") {valore_pssm = "-1";}
 if (sostituzione == "Q111A") {valore_pssm = "-1";}
 if (sostituzione == "A143T") {valore_pssm = "-1";}
 if (sostituzione == "D153R") {valore_pssm = "-1";}
 if (sostituzione == "L166E") {valore_pssm = "-1";}
 if (sostituzione == "D175A") {valore_pssm = "-1";}
 if (sostituzione == "A181R") {valore_pssm = "-1";}
 if (sostituzione == "E218D") {valore_pssm = "-1";}
 if (sostituzione == "H225D") {valore_pssm = "-1";}
 if (sostituzione == "K237H") {valore_pssm = "-1";}
 if (sostituzione == "I242N") {valore_pssm = "-1";}
 if (sostituzione == "T246G") {valore_pssm = "-1";}
 if (sostituzione == "D315M") {valore_pssm = "-1";}
 if (sostituzione == "V316I") {valore_pssm = "-1";}
 if (sostituzione == "K326A") {valore_pssm = "-1";}
 if (sostituzione == "V369F") {valore_pssm = "-1";}
 if (sostituzione == "A381T") {valore_pssm = "-1";}
 if (sostituzione == "R392K") {valore_pssm = "-1";}
 if (sostituzione == "S405R") {valore_pssm = "-1";}
 if (sostituzione == "S424E") {valore_pssm = "-1";}
 if (sostituzione == "L429R") {valore_pssm = "-1";}
 if (sostituzione == "M1L") {valore_pssm = "-1";}
 if (sostituzione == "L16M") {valore_pssm = "-1";}
 if (sostituzione == "L21M") {valore_pssm = "-1";}
 if (sostituzione == "Q57P") {valore_pssm = "-1";}
 if (sostituzione == "E79Q") {valore_pssm = "-1";}
 if (sostituzione == "A108W") {valore_pssm = "-1";}
 if (sostituzione == "Y152T") {valore_pssm = "-1";}
 if (sostituzione == "I154H") {valore_pssm = "-1";}
 if (sostituzione == "S188T") {valore_pssm = "-1";}
 if (sostituzione == "W245F") {valore_pssm = "-1";}
 if (sostituzione == "T246P") {valore_pssm = "-1";}
 if (sostituzione == "F248I") {valore_pssm = "-1";}
 if (sostituzione == "N249M") {valore_pssm = "-1";}
 if (sostituzione == "Q306M") {valore_pssm = "-1";}
 if (sostituzione == "K326F") {valore_pssm = "-1";}
 if (sostituzione == "Y329S") {valore_pssm = "-1";}
 if (sostituzione == "W349Q") {valore_pssm = "-1";}
 if (sostituzione == "W349E") {valore_pssm = "-1";}
 if (sostituzione == "R363V") {valore_pssm = "-1";}
 if (sostituzione == "S364F") {valore_pssm = "-1";}
 if (sostituzione == "Y365F") {valore_pssm = "-1";}
 if (sostituzione == "A381K") {valore_pssm = "-1";}
 if (sostituzione == "M1I") {valore_pssm = "-1";}
 if (sostituzione == "R30N") {valore_pssm = "-1";}
 if (sostituzione == "E59P") {valore_pssm = "-1";}
 if (sostituzione == "E59I") {valore_pssm = "-1";}
 if (sostituzione == "E79H") {valore_pssm = "-1";}
 if (sostituzione == "K82L") {valore_pssm = "-1";}
 if (sostituzione == "L129V") {valore_pssm = "-1";}
 if (sostituzione == "I154R") {valore_pssm = "-1";}
 if (sostituzione == "L180E") {valore_pssm = "-1";}
 if (sostituzione == "P210S") {valore_pssm = "-1";}
 if (sostituzione == "P210E") {valore_pssm = "-1";}
 if (sostituzione == "T217Y") {valore_pssm = "-1";}
 if (sostituzione == "A230I") {valore_pssm = "-1";}
 if (sostituzione == "K240E") {valore_pssm = "-1";}
 if (sostituzione == "I242R") {valore_pssm = "-1";}
 if (sostituzione == "N278F") {valore_pssm = "-1";}
 if (sostituzione == "K326S") {valore_pssm = "-1";}
 if (sostituzione == "G334R") {valore_pssm = "-1";}
 if (sostituzione == "V390K") {valore_pssm = "-1";}
 if (sostituzione == "S424K") {valore_pssm = "-1";}
 if (sostituzione == "R4K") {valore_pssm = "-1";}
 if (sostituzione == "L8C") {valore_pssm = "-1";}
 if (sostituzione == "A29K") {valore_pssm = "-1";}
 if (sostituzione == "E59F") {valore_pssm = "-1";}
 if (sostituzione == "L75D") {valore_pssm = "-1";}
 if (sostituzione == "P98I") {valore_pssm = "-1";}
 if (sostituzione == "S126T") {valore_pssm = "-1";}
 if (sostituzione == "L177Y") {valore_pssm = "-1";}
 if (sostituzione == "F211I") {valore_pssm = "-1";}
 if (sostituzione == "Q221F") {valore_pssm = "-1";}
 if (sostituzione == "M296L") {valore_pssm = "-1";}
 if (sostituzione == "A307G") {valore_pssm = "-1";}
 if (sostituzione == "E358Q") {valore_pssm = "-1";}
 if (sostituzione == "G361V") {valore_pssm = "-1";}
 if (sostituzione == "S364D") {valore_pssm = "-1";}
 if (sostituzione == "A370V") {valore_pssm = "-1";}
 if (sostituzione == "A377S") {valore_pssm = "-1";}
 if (sostituzione == "Q416H") {valore_pssm = "-1";}
 if (sostituzione == "M1V") {valore_pssm = "-1";}
 if (sostituzione == "L21F") {valore_pssm = "-1";}
 if (sostituzione == "A29E") {valore_pssm = "-1";}
 if (sostituzione == "D33A") {valore_pssm = "-1";}
 if (sostituzione == "D33R") {valore_pssm = "-1";}
 if (sostituzione == "F50Y") {valore_pssm = "-1";}
 if (sostituzione == "E59N") {valore_pssm = "-1";}
 if (sostituzione == "I117V") {valore_pssm = "-1";}
 if (sostituzione == "A135N") {valore_pssm = "-1";}
 if (sostituzione == "Q157N") {valore_pssm = "-1";}
 if (sostituzione == "L177D") {valore_pssm = "-1";}
 if (sostituzione == "P210T") {valore_pssm = "-1";}
 if (sostituzione == "I219T") {valore_pssm = "-1";}
 if (sostituzione == "R220I") {valore_pssm = "-1";}
 if (sostituzione == "A230F") {valore_pssm = "-1";}
 if (sostituzione == "K240S") {valore_pssm = "-1";}
 if (sostituzione == "K240H") {valore_pssm = "-1";}
 if (sostituzione == "T246M") {valore_pssm = "-1";}
 if (sostituzione == "S247D") {valore_pssm = "-1";}
 if (sostituzione == "N298K") {valore_pssm = "-1";}
 if (sostituzione == "N298H") {valore_pssm = "-1";}
 if (sostituzione == "G334Q") {valore_pssm = "-1";}
 if (sostituzione == "L372F") {valore_pssm = "-1";}
 if (sostituzione == "Q386E") {valore_pssm = "-1";}
 if (sostituzione == "L16F") {valore_pssm = "-1";}
 if (sostituzione == "S23I") {valore_pssm = "-1";}
 if (sostituzione == "P27M") {valore_pssm = "-1";}
 if (sostituzione == "G28A") {valore_pssm = "-1";}
 if (sostituzione == "S65A") {valore_pssm = "-1";}
 if (sostituzione == "R105S") {valore_pssm = "-1";}
 if (sostituzione == "L166A") {valore_pssm = "-1";}
 if (sostituzione == "L180A") {valore_pssm = "-1";}
 if (sostituzione == "I198V") {valore_pssm = "-1";}
 if (sostituzione == "V199L") {valore_pssm = "-1";}
 if (sostituzione == "Q212R") {valore_pssm = "-1";}
 if (sostituzione == "K237T") {valore_pssm = "-1";}
 if (sostituzione == "D244E") {valore_pssm = "-1";}
 if (sostituzione == "L268I") {valore_pssm = "-1";}
 if (sostituzione == "V269K") {valore_pssm = "-1";}
 if (sostituzione == "L300M") {valore_pssm = "-1";}
 if (sostituzione == "Q330Y") {valore_pssm = "-1";}
 if (sostituzione == "R342K") {valore_pssm = "-1";}
 if (sostituzione == "M353A") {valore_pssm = "-1";}
 if (sostituzione == "S364G") {valore_pssm = "-1";}
 if (sostituzione == "V376M") {valore_pssm = "-1";}
 if (sostituzione == "S405N") {valore_pssm = "-1";}
 if (sostituzione == "N419H") {valore_pssm = "-1";}
 if (sostituzione == "A31V") {valore_pssm = "-1";}
 if (sostituzione == "L45K") {valore_pssm = "-1";}
 if (sostituzione == "M70L") {valore_pssm = "-1";}
 if (sostituzione == "R112T") {valore_pssm = "-1";}
 if (sostituzione == "A143K") {valore_pssm = "-1";}
 if (sostituzione == "A156V") {valore_pssm = "-1";}
 if (sostituzione == "L191I") {valore_pssm = "-1";}
 if (sostituzione == "R220D") {valore_pssm = "-1";}
 if (sostituzione == "K240A") {valore_pssm = "-1";}
 if (sostituzione == "S241T") {valore_pssm = "-1";}
 if (sostituzione == "T246D") {valore_pssm = "-1";}
 if (sostituzione == "E251G") {valore_pssm = "-1";}
 if (sostituzione == "K308V") {valore_pssm = "-1";}
 if (sostituzione == "A368P") {valore_pssm = "-1";}
 if (sostituzione == "A368D") {valore_pssm = "-1";}
 if (sostituzione == "S424H") {valore_pssm = "-1";}
 if (sostituzione == "L429T") {valore_pssm = "-1";}
 if (sostituzione == "N5R") {valore_pssm = "-1";}
 if (sostituzione == "L8H") {valore_pssm = "-1";}
 if (sostituzione == "L54H") {valore_pssm = "-1";}
 if (sostituzione == "E59A") {valore_pssm = "-1";}
 if (sostituzione == "K82E") {valore_pssm = "-1";}
 if (sostituzione == "N139S") {valore_pssm = "-1";}
 if (sostituzione == "D161R") {valore_pssm = "-1";}
 if (sostituzione == "F169H") {valore_pssm = "-1";}
 if (sostituzione == "A181S") {valore_pssm = "-1";}
 if (sostituzione == "T194M") {valore_pssm = "-1";}
 if (sostituzione == "R252M") {valore_pssm = "-1";}
 if (sostituzione == "R252F") {valore_pssm = "-1";}
 if (sostituzione == "V256N") {valore_pssm = "-1";}
 if (sostituzione == "L268V") {valore_pssm = "-1";}
 if (sostituzione == "L300F") {valore_pssm = "-1";}
 if (sostituzione == "H302K") {valore_pssm = "-1";}
 if (sostituzione == "K326H") {valore_pssm = "-1";}
 if (sostituzione == "M353H") {valore_pssm = "-1";}
 if (sostituzione == "I354T") {valore_pssm = "-1";}
 if (sostituzione == "A370T") {valore_pssm = "-1";}
 if (sostituzione == "S405C") {valore_pssm = "-1";}
 if (sostituzione == "L415H") {valore_pssm = "-1";}
 if (sostituzione == "E7Q") {valore_pssm = "-1";}
 if (sostituzione == "L8R") {valore_pssm = "-1";}
 if (sostituzione == "L8E") {valore_pssm = "-1";}
 if (sostituzione == "P27T") {valore_pssm = "-1";}
 if (sostituzione == "Q57C") {valore_pssm = "-1";}
 if (sostituzione == "A97Q") {valore_pssm = "-1";}
 if (sostituzione == "P98A") {valore_pssm = "-1";}
 if (sostituzione == "Q119N") {valore_pssm = "-1";}
 if (sostituzione == "S176C") {valore_pssm = "-1";}
 if (sostituzione == "L191V") {valore_pssm = "-1";}
 if (sostituzione == "V256H") {valore_pssm = "-1";}
 if (sostituzione == "A291T") {valore_pssm = "-1";}
 if (sostituzione == "A291H") {valore_pssm = "-1";}
 if (sostituzione == "G328P") {valore_pssm = "-1";}
 if (sostituzione == "G334H") {valore_pssm = "-1";}
 if (sostituzione == "K374E") {valore_pssm = "-1";}
 if (sostituzione == "A381H") {valore_pssm = "-1";}
 if (sostituzione == "Q57N") {valore_pssm = "-1";}
 if (sostituzione == "E71N") {valore_pssm = "-1";}
 if (sostituzione == "D101N") {valore_pssm = "-1";}
 if (sostituzione == "V124L") {valore_pssm = "-1";}
 if (sostituzione == "A143E") {valore_pssm = "-1";}
 if (sostituzione == "R193H") {valore_pssm = "-1";}
 if (sostituzione == "M267F") {valore_pssm = "-1";}
 if (sostituzione == "F273P") {valore_pssm = "-1";}
 if (sostituzione == "L275F") {valore_pssm = "-1";}
 if (sostituzione == "A307I") {valore_pssm = "-1";}
 if (sostituzione == "K314Q") {valore_pssm = "-1";}
 if (sostituzione == "V339L") {valore_pssm = "-1";}
 if (sostituzione == "I354K") {valore_pssm = "-1";}
 if (sostituzione == "S424Q") {valore_pssm = "-1";}
 if (sostituzione == "N5D") {valore_pssm = "-1";}
 if (sostituzione == "S23Q") {valore_pssm = "-1";}
 if (sostituzione == "A29C") {valore_pssm = "-1";}
 if (sostituzione == "L36I") {valore_pssm = "-1";}
 if (sostituzione == "A37S") {valore_pssm = "-1";}
 if (sostituzione == "W81F") {valore_pssm = "-1";}
 if (sostituzione == "Q99S") {valore_pssm = "-1";}
 if (sostituzione == "S102E") {valore_pssm = "-1";}
 if (sostituzione == "K140T") {valore_pssm = "-1";}
 if (sostituzione == "D161A") {valore_pssm = "-1";}
 if (sostituzione == "L166C") {valore_pssm = "-1";}
 if (sostituzione == "L180Q") {valore_pssm = "-1";}
 if (sostituzione == "D233T") {valore_pssm = "-1";}
 if (sostituzione == "W277C") {valore_pssm = "-1";}
 if (sostituzione == "L294I") {valore_pssm = "-1";}
 if (sostituzione == "S297T") {valore_pssm = "-1";}
 if (sostituzione == "N298E") {valore_pssm = "-1";}
 if (sostituzione == "A307C") {valore_pssm = "-1";}
 if (sostituzione == "L344I") {valore_pssm = "-1";}
 if (sostituzione == "E358K") {valore_pssm = "-1";}
 if (sostituzione == "S371N") {valore_pssm = "-1";}
 if (sostituzione == "T410A") {valore_pssm = "-1";}
 if (sostituzione == "N5A") {valore_pssm = "-1";}
 if (sostituzione == "L8Q") {valore_pssm = "-1";}
 if (sostituzione == "A15V") {valore_pssm = "-1";}
 if (sostituzione == "R17S") {valore_pssm = "-1";}
 if (sostituzione == "P27K") {valore_pssm = "-1";}
 if (sostituzione == "P27E") {valore_pssm = "-1";}
 if (sostituzione == "L32S") {valore_pssm = "-1";}
 if (sostituzione == "D55H") {valore_pssm = "-1";}
 if (sostituzione == "S65V") {valore_pssm = "-1";}
 if (sostituzione == "A143H") {valore_pssm = "-1";}
 if (sostituzione == "N179A") {valore_pssm = "-1";}
 if (sostituzione == "P210V") {valore_pssm = "-1";}
 if (sostituzione == "P210Q") {valore_pssm = "-1";}
 if (sostituzione == "R220C") {valore_pssm = "-1";}
 if (sostituzione == "W277M") {valore_pssm = "-1";}
 if (sostituzione == "Q280V") {valore_pssm = "-1";}
 if (sostituzione == "H302V") {valore_pssm = "-1";}
 if (sostituzione == "Q306D") {valore_pssm = "-1";}
 if (sostituzione == "Q2H") {valore_pssm = "-1";}
 if (sostituzione == "P27Q") {valore_pssm = "-1";}
 if (sostituzione == "A29H") {valore_pssm = "-1";}
 if (sostituzione == "Q57G") {valore_pssm = "-1";}
 if (sostituzione == "K67A") {valore_pssm = "-1";}
 if (sostituzione == "E87S") {valore_pssm = "-1";}
 if (sostituzione == "V137K") {valore_pssm = "-1";}
 if (sostituzione == "F145R") {valore_pssm = "-1";}
 if (sostituzione == "F149M") {valore_pssm = "-1";}
 if (sostituzione == "S176I") {valore_pssm = "-1";}
 if (sostituzione == "P205G") {valore_pssm = "-1";}
 if (sostituzione == "P210R") {valore_pssm = "-1";}
 if (sostituzione == "E251P") {valore_pssm = "-1";}
 if (sostituzione == "V254C") {valore_pssm = "-1";}
 if (sostituzione == "Q279K") {valore_pssm = "-1";}
 if (sostituzione == "S297A") {valore_pssm = "-1";}
 if (sostituzione == "G346V") {valore_pssm = "-1";}
 if (sostituzione == "S364C") {valore_pssm = "-1";}
 if (sostituzione == "V376F") {valore_pssm = "-1";}
 if (sostituzione == "N379H") {valore_pssm = "-1";}
 if (sostituzione == "A381E") {valore_pssm = "-1";}
 if (sostituzione == "I384L") {valore_pssm = "-1";}
 if (sostituzione == "V390T") {valore_pssm = "-1";}
 if (sostituzione == "N408H") {valore_pssm = "-1";}
 if (sostituzione == "A31K") {valore_pssm = "-1";}
 if (sostituzione == "L36V") {valore_pssm = "-1";}
 if (sostituzione == "E59G") {valore_pssm = "-1";}
 if (sostituzione == "E74Q") {valore_pssm = "-1";}
 if (sostituzione == "E79L") {valore_pssm = "-1";}
 if (sostituzione == "R118V") {valore_pssm = "-1";}
 if (sostituzione == "V137C") {valore_pssm = "-1";}
 if (sostituzione == "D153T") {valore_pssm = "-1";}
 if (sostituzione == "A181T") {valore_pssm = "-1";}
 if (sostituzione == "G183Q") {valore_pssm = "-1";}
 if (sostituzione == "N215H") {valore_pssm = "-1";}
 if (sostituzione == "R220P") {valore_pssm = "-1";}
 if (sostituzione == "L294V") {valore_pssm = "-1";}
 if (sostituzione == "F295M") {valore_pssm = "-1";}
 if (sostituzione == "Q306S") {valore_pssm = "-1";}
 if (sostituzione == "L311F") {valore_pssm = "-1";}
 if (sostituzione == "Y329F") {valore_pssm = "-1";}
 if (sostituzione == "L344V") {valore_pssm = "-1";}
 if (sostituzione == "L429C") {valore_pssm = "-1";}
 if (sostituzione == "L429Q") {valore_pssm = "-1";}
 if (sostituzione == "A13G") {valore_pssm = "-1";}
 if (sostituzione == "R17A") {valore_pssm = "-1";}
 if (sostituzione == "A37F") {valore_pssm = "-1";}
 if (sostituzione == "A37T") {valore_pssm = "-1";}
 if (sostituzione == "L45F") {valore_pssm = "-1";}
 if (sostituzione == "L45Q") {valore_pssm = "-1";}
 if (sostituzione == "K67V") {valore_pssm = "-1";}
 if (sostituzione == "M70T") {valore_pssm = "-1";}
 if (sostituzione == "W81V") {valore_pssm = "-1";}
 if (sostituzione == "K82H") {valore_pssm = "-1";}
 if (sostituzione == "A135C") {valore_pssm = "-1";}
 if (sostituzione == "W236Y") {valore_pssm = "-1";}
 if (sostituzione == "V269F") {valore_pssm = "-1";}
 if (sostituzione == "T282V") {valore_pssm = "-1";}
 if (sostituzione == "L310T") {valore_pssm = "-1";}
 if (sostituzione == "I317V") {valore_pssm = "-1";}
 if (sostituzione == "Q330N") {valore_pssm = "-1";}
 if (sostituzione == "Q357E") {valore_pssm = "-1";}
 if (sostituzione == "S371V") {valore_pssm = "-1";}
 if (sostituzione == "A20V") {valore_pssm = "-1";}
 if (sostituzione == "D25K") {valore_pssm = "-1";}
 if (sostituzione == "P27R") {valore_pssm = "-1";}
 if (sostituzione == "A29Q") {valore_pssm = "-1";}
 if (sostituzione == "T39K") {valore_pssm = "-1";}
 if (sostituzione == "L45R") {valore_pssm = "-1";}
 if (sostituzione == "L54K") {valore_pssm = "-1";}
 if (sostituzione == "E79S") {valore_pssm = "-1";}
 if (sostituzione == "Q99K") {valore_pssm = "-1";}
 if (sostituzione == "E103D") {valore_pssm = "-1";}
 if (sostituzione == "D109K") {valore_pssm = "-1";}
 if (sostituzione == "Q119P") {valore_pssm = "-1";}
 if (sostituzione == "S176D") {valore_pssm = "-1";}
 if (sostituzione == "D233S") {valore_pssm = "-1";}
 if (sostituzione == "R252D") {valore_pssm = "-1";}
 if (sostituzione == "V269T") {valore_pssm = "-1";}
 if (sostituzione == "N298Q") {valore_pssm = "-1";}
 if (sostituzione == "W349F") {valore_pssm = "-1";}
 if (sostituzione == "T410V") {valore_pssm = "-1";}
 if (sostituzione == "G11A") {valore_pssm = "-1";}
 if (sostituzione == "S23R") {valore_pssm = "-1";}
 if (sostituzione == "A31S") {valore_pssm = "-1";}
 if (sostituzione == "L45M") {valore_pssm = "-1";}
 if (sostituzione == "L54C") {valore_pssm = "-1";}
 if (sostituzione == "Q57L") {valore_pssm = "-1";}
 if (sostituzione == "Q57Y") {valore_pssm = "-1";}
 if (sostituzione == "W81I") {valore_pssm = "-1";}
 if (sostituzione == "A84T") {valore_pssm = "-1";}
 if (sostituzione == "Y151F") {valore_pssm = "-1";}
 if (sostituzione == "F169V") {valore_pssm = "-1";}
 if (sostituzione == "K240C") {valore_pssm = "-1";}
 if (sostituzione == "L243M") {valore_pssm = "-1";}
 if (sostituzione == "N278Y") {valore_pssm = "-1";}
 if (sostituzione == "A291V") {valore_pssm = "-1";}
 if (sostituzione == "S304T") {valore_pssm = "-1";}
 if (sostituzione == "A307P") {valore_pssm = "-1";}
 if (sostituzione == "R332T") {valore_pssm = "-1";}
 if (sostituzione == "S345T") {valore_pssm = "-1";}
 if (sostituzione == "E358H") {valore_pssm = "-1";}
 if (sostituzione == "G361L") {valore_pssm = "-1";}
 if (sostituzione == "G361I") {valore_pssm = "-1";}
 if (sostituzione == "K393Y") {valore_pssm = "-1";}
 if (sostituzione == "S405I") {valore_pssm = "-1";}
 if (sostituzione == "Q422K") {valore_pssm = "-1";}
 if (sostituzione == "L32M") {valore_pssm = "-1";}
 if (sostituzione == "A37M") {valore_pssm = "-1";}
 if (sostituzione == "E71Q") {valore_pssm = "-1";}
 if (sostituzione == "E79R") {valore_pssm = "-1";}
 if (sostituzione == "D101Y") {valore_pssm = "-1";}
 if (sostituzione == "Q111H") {valore_pssm = "-1";}
 if (sostituzione == "A135P") {valore_pssm = "-1";}
 if (sostituzione == "Y152A") {valore_pssm = "-1";}
 if (sostituzione == "Q157D") {valore_pssm = "-1";}
 if (sostituzione == "F169I") {valore_pssm = "-1";}
 if (sostituzione == "L180C") {valore_pssm = "-1";}
 if (sostituzione == "R193V") {valore_pssm = "-1";}
 if (sostituzione == "S241H") {valore_pssm = "-1";}
 if (sostituzione == "V281I") {valore_pssm = "-1";}
 if (sostituzione == "L286F") {valore_pssm = "-1";}
 if (sostituzione == "L310Y") {valore_pssm = "-1";}
 if (sostituzione == "S364I") {valore_pssm = "-1";}
 if (sostituzione == "Y397H") {valore_pssm = "-1";}
 if (sostituzione == "A31Q") {valore_pssm = "-1";}
 if (sostituzione == "L54R") {valore_pssm = "-1";}
 if (sostituzione == "L54E") {valore_pssm = "-1";}
 if (sostituzione == "V77K") {valore_pssm = "-1";}
 if (sostituzione == "D109E") {valore_pssm = "-1";}
 if (sostituzione == "V137F") {valore_pssm = "-1";}
 if (sostituzione == "V137Q") {valore_pssm = "-1";}
 if (sostituzione == "N139Q") {valore_pssm = "-1";}
 if (sostituzione == "L166S") {valore_pssm = "-1";}
 if (sostituzione == "D182G") {valore_pssm = "-1";}
 if (sostituzione == "S197D") {valore_pssm = "-1";}
 if (sostituzione == "R220L") {valore_pssm = "-1";}
 if (sostituzione == "F229H") {valore_pssm = "-1";}
 if (sostituzione == "V269H") {valore_pssm = "-1";}
 if (sostituzione == "T282K") {valore_pssm = "-1";}
 if (sostituzione == "N298I") {valore_pssm = "-1";}
 if (sostituzione == "A309I") {valore_pssm = "-1";}
 if (sostituzione == "K326C") {valore_pssm = "-1";}
 if (sostituzione == "Y329N") {valore_pssm = "-1";}
 if (sostituzione == "I367L") {valore_pssm = "-1";}
 if (sostituzione == "A368I") {valore_pssm = "-1";}
 if (sostituzione == "S401N") {valore_pssm = "-1";}
 if (sostituzione == "A15S") {valore_pssm = "-1";}
 if (sostituzione == "W24S") {valore_pssm = "-1";}
 if (sostituzione == "N34P") {valore_pssm = "-1";}
 if (sostituzione == "D55K") {valore_pssm = "-1";}
 if (sostituzione == "K82V") {valore_pssm = "-1";}
 if (sostituzione == "A97R") {valore_pssm = "-1";}
 if (sostituzione == "A108V") {valore_pssm = "-1";}
 if (sostituzione == "V137E") {valore_pssm = "-1";}
 if (sostituzione == "N215V") {valore_pssm = "-1";}
 if (sostituzione == "N215E") {valore_pssm = "-1";}
 if (sostituzione == "W245M") {valore_pssm = "-1";}
 if (sostituzione == "V254P") {valore_pssm = "-1";}
 if (sostituzione == "R301P") {valore_pssm = "-1";}
 if (sostituzione == "D315N") {valore_pssm = "-1";}
 if (sostituzione == "L331C") {valore_pssm = "-1";}
 if (sostituzione == "S345D") {valore_pssm = "-1";}
 if (sostituzione == "M353K") {valore_pssm = "-1";}
 if (sostituzione == "M353R") {valore_pssm = "-1";}
 if (sostituzione == "T410K") {valore_pssm = "-1";}
 if (sostituzione == "K426Q") {valore_pssm = "-1";}
 if (sostituzione == "R4V") {valore_pssm = "-1";}
 if (sostituzione == "P6K") {valore_pssm = "-1";}
 if (sostituzione == "E7K") {valore_pssm = "-1";}
 if (sostituzione == "A13P") {valore_pssm = "-1";}
 if (sostituzione == "L19M") {valore_pssm = "-1";}
 if (sostituzione == "V22L") {valore_pssm = "-1";}
 if (sostituzione == "S23C") {valore_pssm = "-1";}
 if (sostituzione == "T41N") {valore_pssm = "-1";}
 if (sostituzione == "T41D") {valore_pssm = "-1";}
 if (sostituzione == "M96L") {valore_pssm = "-1";}
 if (sostituzione == "M96T") {valore_pssm = "-1";}
 if (sostituzione == "A97H") {valore_pssm = "-1";}
 if (sostituzione == "D101E") {valore_pssm = "-1";}
 if (sostituzione == "L131M") {valore_pssm = "-1";}
 if (sostituzione == "N139H") {valore_pssm = "-1";}
 if (sostituzione == "D161N") {valore_pssm = "-1";}
 if (sostituzione == "D175I") {valore_pssm = "-1";}
 if (sostituzione == "N192K") {valore_pssm = "-1";}
 if (sostituzione == "K213Q") {valore_pssm = "-1";}
 if (sostituzione == "N215K") {valore_pssm = "-1";}
 if (sostituzione == "R220F") {valore_pssm = "-1";}
 if (sostituzione == "V254L") {valore_pssm = "-1";}
 if (sostituzione == "W277G") {valore_pssm = "-1";}
 if (sostituzione == "T282E") {valore_pssm = "-1";}
 if (sostituzione == "N298R") {valore_pssm = "-1";}
 if (sostituzione == "Q333G") {valore_pssm = "-1";}
 if (sostituzione == "R363I") {valore_pssm = "-1";}
 if (sostituzione == "A381Q") {valore_pssm = "-1";}
 if (sostituzione == "V390H") {valore_pssm = "-1";}
 if (sostituzione == "E398Q") {valore_pssm = "-1";}
 if (sostituzione == "R404Q") {valore_pssm = "-1";}
 if (sostituzione == "T410E") {valore_pssm = "-1";}
 if (sostituzione == "E418Q") {valore_pssm = "-1";}
 if (sostituzione == "S23L") {valore_pssm = "-1";}
 if (sostituzione == "A29R") {valore_pssm = "-1";}
 if (sostituzione == "A31H") {valore_pssm = "-1";}
 if (sostituzione == "A37K") {valore_pssm = "-1";}
 if (sostituzione == "Q57M") {valore_pssm = "-1";}
 if (sostituzione == "E58Q") {valore_pssm = "-1";}
 if (sostituzione == "E59C") {valore_pssm = "-1";}
 if (sostituzione == "E87I") {valore_pssm = "-1";}
 if (sostituzione == "A97T") {valore_pssm = "-1";}
 if (sostituzione == "S102K") {valore_pssm = "-1";}
 if (sostituzione == "R105T") {valore_pssm = "-1";}
 if (sostituzione == "A181F") {valore_pssm = "-1";}
 if (sostituzione == "F211H") {valore_pssm = "-1";}
 if (sostituzione == "I219S") {valore_pssm = "-1";}
 if (sostituzione == "G258M") {valore_pssm = "-1";}
 if (sostituzione == "A309G") {valore_pssm = "-1";}
 if (sostituzione == "D313P") {valore_pssm = "-1";}
 if (sostituzione == "D313F") {valore_pssm = "-1";}
 if (sostituzione == "E341C") {valore_pssm = "-1";}
 if (sostituzione == "A381C") {valore_pssm = "-1";}
 if (sostituzione == "K426E") {valore_pssm = "-1";}
 if (sostituzione == "R17L") {valore_pssm = "-1";}
 if (sostituzione == "P27S") {valore_pssm = "-1";}
 if (sostituzione == "D33T") {valore_pssm = "-1";}
 if (sostituzione == "L54A") {valore_pssm = "-1";}
 if (sostituzione == "E58K") {valore_pssm = "-1";}
 if (sostituzione == "K67D") {valore_pssm = "-1";}
 if (sostituzione == "L68M") {valore_pssm = "-1";}
 if (sostituzione == "V77I") {valore_pssm = "-1";}
 if (sostituzione == "L89T") {valore_pssm = "-1";}
 if (sostituzione == "A97V") {valore_pssm = "-1";}
 if (sostituzione == "Q99H") {valore_pssm = "-1";}
 if (sostituzione == "S102V") {valore_pssm = "-1";}
 if (sostituzione == "I117M") {valore_pssm = "-1";}
 if (sostituzione == "N122A") {valore_pssm = "-1";}
 if (sostituzione == "Y152S") {valore_pssm = "-1";}
 if (sostituzione == "D153S") {valore_pssm = "-1";}
 if (sostituzione == "N179D") {valore_pssm = "-1";}
 if (sostituzione == "H186I") {valore_pssm = "-1";}
 if (sostituzione == "T217G") {valore_pssm = "-1";}
 if (sostituzione == "I219K") {valore_pssm = "-1";}
 if (sostituzione == "Q221P") {valore_pssm = "-1";}
 if (sostituzione == "N224T") {valore_pssm = "-1";}
 if (sostituzione == "R252N") {valore_pssm = "-1";}
 if (sostituzione == "N278L") {valore_pssm = "-1";}
 if (sostituzione == "N278M") {valore_pssm = "-1";}
 if (sostituzione == "A285V") {valore_pssm = "-1";}
 if (sostituzione == "Q306T") {valore_pssm = "-1";}
 if (sostituzione == "Y329E") {valore_pssm = "-1";}
 if (sostituzione == "Q333P") {valore_pssm = "-1";}
 if (sostituzione == "A348S") {valore_pssm = "-1";}
 if (sostituzione == "W349Y") {valore_pssm = "-1";}
 if (sostituzione == "I354C") {valore_pssm = "-1";}
 if (sostituzione == "V390A") {valore_pssm = "-1";}
 if (sostituzione == "T420S") {valore_pssm = "-1";}
 if (sostituzione == "S424R") {valore_pssm = "-1";}
 if (sostituzione == "R4I") {valore_pssm = "-1";}
 if (sostituzione == "R17Q") {valore_pssm = "-1";}
 if (sostituzione == "F18L") {valore_pssm = "-1";}
 if (sostituzione == "R30S") {valore_pssm = "-1";}
 if (sostituzione == "N53S") {valore_pssm = "-1";}
 if (sostituzione == "M76V") {valore_pssm = "-1";}
 if (sostituzione == "N122G") {valore_pssm = "-1";}
 if (sostituzione == "L131Y") {valore_pssm = "-1";}
 if (sostituzione == "A143R") {valore_pssm = "-1";}
 if (sostituzione == "G183H") {valore_pssm = "-1";}
 if (sostituzione == "S197G") {valore_pssm = "-1";}
 if (sostituzione == "W209F") {valore_pssm = "-1";}
 if (sostituzione == "Q221I") {valore_pssm = "-1";}
 if (sostituzione == "I239L") {valore_pssm = "-1";}
 if (sostituzione == "E251I") {valore_pssm = "-1";}
 if (sostituzione == "A288V") {valore_pssm = "-1";}
 if (sostituzione == "K308R") {valore_pssm = "-1";}
 if (sostituzione == "D315P") {valore_pssm = "-1";}
 if (sostituzione == "Y329Q") {valore_pssm = "-1";}
 if (sostituzione == "E341G") {valore_pssm = "-1";}
 if (sostituzione == "W349I") {valore_pssm = "-1";}
 if (sostituzione == "A350L") {valore_pssm = "-1";}
 if (sostituzione == "A352S") {valore_pssm = "-1";}
 if (sostituzione == "L415T") {valore_pssm = "-1";}
 if (sostituzione == "M421F") {valore_pssm = "-1";}
 if (sostituzione == "M423F") {valore_pssm = "-1";}
 if (sostituzione == "S424N") {valore_pssm = "-1";}
 if (sostituzione == "L429A") {valore_pssm = "-1";}
 if (sostituzione == "L19F") {valore_pssm = "-1";}
 if (sostituzione == "A31T") {valore_pssm = "-1";}
 if (sostituzione == "A37H") {valore_pssm = "-1";}
 if (sostituzione == "E48A") {valore_pssm = "-1";}
 if (sostituzione == "E58H") {valore_pssm = "-1";}
 if (sostituzione == "P110R") {valore_pssm = "-1";}
 if (sostituzione == "S126E") {valore_pssm = "-1";}
 if (sostituzione == "V137G") {valore_pssm = "-1";}
 if (sostituzione == "V137H") {valore_pssm = "-1";}
 if (sostituzione == "Q157A") {valore_pssm = "-1";}
 if (sostituzione == "D182Y") {valore_pssm = "-1";}
 if (sostituzione == "G195Q") {valore_pssm = "-1";}
 if (sostituzione == "I219C") {valore_pssm = "-1";}
 if (sostituzione == "A230Y") {valore_pssm = "-1";}
 if (sostituzione == "K237A") {valore_pssm = "-1";}
 if (sostituzione == "K240Y") {valore_pssm = "-1";}
 if (sostituzione == "S304E") {valore_pssm = "-1";}
 if (sostituzione == "D315C") {valore_pssm = "-1";}
 if (sostituzione == "E338D") {valore_pssm = "-1";}
 if (sostituzione == "A377T") {valore_pssm = "-1";}
 if (sostituzione == "A13C") {valore_pssm = "-1";}
 if (sostituzione == "D25H") {valore_pssm = "-1";}
 if (sostituzione == "R49F") {valore_pssm = "-1";}
 if (sostituzione == "E59Y") {valore_pssm = "-1";}
 if (sostituzione == "L68F") {valore_pssm = "-1";}
 if (sostituzione == "S78V") {valore_pssm = "-1";}
 if (sostituzione == "K140S") {valore_pssm = "-1";}
 if (sostituzione == "A143I") {valore_pssm = "-1";}
 if (sostituzione == "E203K") {valore_pssm = "-1";}
 if (sostituzione == "A230M") {valore_pssm = "-1";}
 if (sostituzione == "S235H") {valore_pssm = "-1";}
 if (sostituzione == "W245H") {valore_pssm = "-1";}
 if (sostituzione == "F248H") {valore_pssm = "-1";}
 if (sostituzione == "V269R") {valore_pssm = "-1";}
 if (sostituzione == "H302R") {valore_pssm = "-1";}
 if (sostituzione == "D313I") {valore_pssm = "-1";}
 if (sostituzione == "D313C") {valore_pssm = "-1";}
 if (sostituzione == "F337T") {valore_pssm = "-1";}
 if (sostituzione == "E341F") {valore_pssm = "-1";}
 if (sostituzione == "W349A") {valore_pssm = "-1";}
 if (sostituzione == "K374H") {valore_pssm = "-1";}
 if (sostituzione == "F383Y") {valore_pssm = "-1";}
 if (sostituzione == "Q386K") {valore_pssm = "-1";}
 if (sostituzione == "T420V") {valore_pssm = "-1";}
 if (sostituzione == "R17E") {valore_pssm = "-1";}
 if (sostituzione == "R30K") {valore_pssm = "-1";}
 if (sostituzione == "E59L") {valore_pssm = "-1";}
 if (sostituzione == "A84C") {valore_pssm = "-1";}
 if (sostituzione == "N122P") {valore_pssm = "-1";}
 if (sostituzione == "F145H") {valore_pssm = "-1";}
 if (sostituzione == "D182I") {valore_pssm = "-1";}
 if (sostituzione == "E218Y") {valore_pssm = "-1";}
 if (sostituzione == "D233V") {valore_pssm = "-1";}
 if (sostituzione == "D233N") {valore_pssm = "-1";}
 if (sostituzione == "R252Y") {valore_pssm = "-1";}
 if (sostituzione == "R252C") {valore_pssm = "-1";}
 if (sostituzione == "V254M") {valore_pssm = "-1";}
 if (sostituzione == "M284Y") {valore_pssm = "-1";}
 if (sostituzione == "D299Y") {valore_pssm = "-1";}
 if (sostituzione == "K314T") {valore_pssm = "-1";}
 if (sostituzione == "L331Y") {valore_pssm = "-1";}
 if (sostituzione == "E341D") {valore_pssm = "-1";}
 if (sostituzione == "A348Q") {valore_pssm = "-1";}
 if (sostituzione == "M353Q") {valore_pssm = "-1";}
 if (sostituzione == "I354Q") {valore_pssm = "-1";}
 if (sostituzione == "S364M") {valore_pssm = "-1";}
 if (sostituzione == "A381R") {valore_pssm = "-1";}
 if (sostituzione == "V390L") {valore_pssm = "-1";}
 if (sostituzione == "S401G") {valore_pssm = "-1";}
 if (sostituzione == "S401Y") {valore_pssm = "-1";}
 if (sostituzione == "S405G") {valore_pssm = "-1";}
 if (sostituzione == "I407M") {valore_pssm = "-1";}
 if (sostituzione == "G11T") {valore_pssm = "-1";}
 if (sostituzione == "L21S") {valore_pssm = "-1";}
 if (sostituzione == "M51V") {valore_pssm = "-1";}
 if (sostituzione == "M51Q") {valore_pssm = "-1";}
 if (sostituzione == "L54Q") {valore_pssm = "-1";}
 if (sostituzione == "E74R") {valore_pssm = "-1";}
 if (sostituzione == "K82S") {valore_pssm = "-1";}
 if (sostituzione == "S102H") {valore_pssm = "-1";}
 if (sostituzione == "A108S") {valore_pssm = "-1";}
 if (sostituzione == "K140A") {valore_pssm = "-1";}
 if (sostituzione == "D175P") {valore_pssm = "-1";}
 if (sostituzione == "D182F") {valore_pssm = "-1";}
 if (sostituzione == "S188E") {valore_pssm = "-1";}
 if (sostituzione == "E218Q") {valore_pssm = "-1";}
 if (sostituzione == "Q221C") {valore_pssm = "-1";}
 if (sostituzione == "V254F") {valore_pssm = "-1";}
 if (sostituzione == "Q283E") {valore_pssm = "-1";}
 if (sostituzione == "L311M") {valore_pssm = "-1";}
 if (sostituzione == "Q312E") {valore_pssm = "-1";}
 if (sostituzione == "A350I") {valore_pssm = "-1";}
 if (sostituzione == "A352L") {valore_pssm = "-1";}
 if (sostituzione == "V390R") {valore_pssm = "-1";}
 if (sostituzione == "M1H") {valore_pssm = "-1";}
 if (sostituzione == "R17H") {valore_pssm = "-1";}
 if (sostituzione == "S23G") {valore_pssm = "-1";}
 if (sostituzione == "A29G") {valore_pssm = "-1";}
 if (sostituzione == "A29L") {valore_pssm = "-1";}
 if (sostituzione == "E48H") {valore_pssm = "-1";}
 if (sostituzione == "M51H") {valore_pssm = "-1";}
 if (sostituzione == "S65D") {valore_pssm = "-1";}
 if (sostituzione == "M72N") {valore_pssm = "-1";}
 if (sostituzione == "Q119D") {valore_pssm = "-1";}
 if (sostituzione == "L120F") {valore_pssm = "-1";}
 if (sostituzione == "V137R") {valore_pssm = "-1";}
 if (sostituzione == "I154T") {valore_pssm = "-1";}
 if (sostituzione == "S176F") {valore_pssm = "-1";}
 if (sostituzione == "I198M") {valore_pssm = "-1";}
 if (sostituzione == "I219H") {valore_pssm = "-1";}
 if (sostituzione == "F248Y") {valore_pssm = "-1";}
 if (sostituzione == "D315G") {valore_pssm = "-1";}
 if (sostituzione == "A348V") {valore_pssm = "-1";}
 if (sostituzione == "A348H") {valore_pssm = "-1";}
 if (sostituzione == "I354A") {valore_pssm = "-1";}
 if (sostituzione == "E358R") {valore_pssm = "-1";}
 if (sostituzione == "A368M") {valore_pssm = "-1";}
 if (sostituzione == "A370K") {valore_pssm = "-1";}
 if (sostituzione == "V390D") {valore_pssm = "-1";}
 if (sostituzione == "K391Q") {valore_pssm = "-1";}
 if (sostituzione == "Q422R") {valore_pssm = "-1";}
 if (sostituzione == "L8Y") {valore_pssm = "-1";}
 if (sostituzione == "S65G") {valore_pssm = "-1";}
 if (sostituzione == "V124A") {valore_pssm = "-1";}
 if (sostituzione == "A135I") {valore_pssm = "-1";}
 if (sostituzione == "G150E") {valore_pssm = "-1";}
 if (sostituzione == "D182M") {valore_pssm = "-1";}
 if (sostituzione == "K185R") {valore_pssm = "-1";}
 if (sostituzione == "S188K") {valore_pssm = "-1";}
 if (sostituzione == "T194S") {valore_pssm = "-1";}
 if (sostituzione == "S201T") {valore_pssm = "-1";}
 if (sostituzione == "K213H") {valore_pssm = "-1";}
 if (sostituzione == "S238T") {valore_pssm = "-1";}
 if (sostituzione == "V281E") {valore_pssm = "-1";}
 if (sostituzione == "P305A") {valore_pssm = "-1";}
 if (sostituzione == "Q333I") {valore_pssm = "-1";}
 if (sostituzione == "E341N") {valore_pssm = "-1";}
 if (sostituzione == "G346P") {valore_pssm = "-1";}
 if (sostituzione == "M353E") {valore_pssm = "-1";}
 if (sostituzione == "I359F") {valore_pssm = "-1";}
 if (sostituzione == "K391E") {valore_pssm = "-1";}
 if (sostituzione == "R404E") {valore_pssm = "-1";}
 if (sostituzione == "S405P") {valore_pssm = "-1";}
 if (sostituzione == "S424V") {valore_pssm = "-1";}
 if (sostituzione == "L429G") {valore_pssm = "-1";}
 if (sostituzione == "L429P") {valore_pssm = "-1";}
 if (sostituzione == "L429S") {valore_pssm = "-1";}
 if (sostituzione == "N5G") {valore_pssm = "-1";}
 if (sostituzione == "A13I") {valore_pssm = "-1";}
 if (sostituzione == "D33F") {valore_pssm = "-1";}
 if (sostituzione == "D33S") {valore_pssm = "-1";}
 if (sostituzione == "T41I") {valore_pssm = "-1";}
 if (sostituzione == "F69V") {valore_pssm = "-1";}
 if (sostituzione == "S78D") {valore_pssm = "-1";}
 if (sostituzione == "S102Q") {valore_pssm = "-1";}
 if (sostituzione == "Q107H") {valore_pssm = "-1";}
 if (sostituzione == "D136S") {valore_pssm = "-1";}
 if (sostituzione == "S197C") {valore_pssm = "-1";}
 if (sostituzione == "I219E") {valore_pssm = "-1";}
 if (sostituzione == "E251C") {valore_pssm = "-1";}
 if (sostituzione == "V269A") {valore_pssm = "-1";}
 if (sostituzione == "Q279H") {valore_pssm = "-1";}
 if (sostituzione == "K314H") {valore_pssm = "-1";}
 if (sostituzione == "E341P") {valore_pssm = "-1";}
 if (sostituzione == "R363L") {valore_pssm = "-1";}
 if (sostituzione == "A368F") {valore_pssm = "-1";}
 if (sostituzione == "A370E") {valore_pssm = "-1";}
 if (sostituzione == "T385S") {valore_pssm = "-1";}
 if (sostituzione == "T400S") {valore_pssm = "-1";}
 if (sostituzione == "T412S") {valore_pssm = "-1";}
 if (sostituzione == "L14T") {valore_pssm = "-1";}
 if (sostituzione == "A37C") {valore_pssm = "-1";}
 if (sostituzione == "L54Y") {valore_pssm = "-1";}
 if (sostituzione == "D55T") {valore_pssm = "-1";}
 if (sostituzione == "K82T") {valore_pssm = "-1";}
 if (sostituzione == "D83K") {valore_pssm = "-1";}
 if (sostituzione == "M96F") {valore_pssm = "-1";}
 if (sostituzione == "Q107F") {valore_pssm = "-1";}
 if (sostituzione == "R118N") {valore_pssm = "-1";}
 if (sostituzione == "N122V") {valore_pssm = "-1";}
 if (sostituzione == "L129M") {valore_pssm = "-1";}
 if (sostituzione == "D175G") {valore_pssm = "-1";}
 if (sostituzione == "D175F") {valore_pssm = "-1";}
 if (sostituzione == "K185T") {valore_pssm = "-1";}
 if (sostituzione == "N192S") {valore_pssm = "-1";}
 if (sostituzione == "W209K") {valore_pssm = "-1";}
 if (sostituzione == "N215R") {valore_pssm = "-1";}
 if (sostituzione == "N228H") {valore_pssm = "-1";}
 if (sostituzione == "Q330P") {valore_pssm = "-1";}
 if (sostituzione == "I354E") {valore_pssm = "-1";}
 if (sostituzione == "R363Q") {valore_pssm = "-1";}
 if (sostituzione == "S371G") {valore_pssm = "-1";}
 if (sostituzione == "S401P") {valore_pssm = "-1";}
 if (sostituzione == "L429Y") {valore_pssm = "-1";}
 if (sostituzione == "I26M") {valore_pssm = "-1";}
 if (sostituzione == "A29F") {valore_pssm = "-1";}
 if (sostituzione == "E48Q") {valore_pssm = "-1";}
 if (sostituzione == "P98M") {valore_pssm = "-1";}
 if (sostituzione == "Q107K") {valore_pssm = "-1";}
 if (sostituzione == "S126H") {valore_pssm = "-1";}
 if (sostituzione == "Y151E") {valore_pssm = "-1";}
 if (sostituzione == "Y152L") {valore_pssm = "-1";}
 if (sostituzione == "P210D") {valore_pssm = "-1";}
 if (sostituzione == "E218H") {valore_pssm = "-1";}
 if (sostituzione == "I219R") {valore_pssm = "-1";}
 if (sostituzione == "Q221M") {valore_pssm = "-1";}
 if (sostituzione == "T282Q") {valore_pssm = "-1";}
 if (sostituzione == "T282H") {valore_pssm = "-1";}
 if (sostituzione == "H302N") {valore_pssm = "-1";}
 if (sostituzione == "A309C") {valore_pssm = "-1";}
 if (sostituzione == "D313Y") {valore_pssm = "-1";}
 if (sostituzione == "G334S") {valore_pssm = "-1";}
 if (sostituzione == "D335T") {valore_pssm = "-1";}
 if (sostituzione == "D335H") {valore_pssm = "-1";}
 if (sostituzione == "E358D") {valore_pssm = "-1";}
 if (sostituzione == "R363T") {valore_pssm = "-1";}
 if (sostituzione == "S364L") {valore_pssm = "-1";}
 if (sostituzione == "Y365H") {valore_pssm = "-1";}
 if (sostituzione == "V369T") {valore_pssm = "-1";}
 if (sostituzione == "I407F") {valore_pssm = "-1";}
 if (sostituzione == "T410Q") {valore_pssm = "-1";}
 if (sostituzione == "E7H") {valore_pssm = "-1";}
 if (sostituzione == "R17M") {valore_pssm = "-1";}
 if (sostituzione == "L32T") {valore_pssm = "-1";}
 if (sostituzione == "A37E") {valore_pssm = "-1";}
 if (sostituzione == "A84S") {valore_pssm = "-1";}
 if (sostituzione == "A108T") {valore_pssm = "-1";}
 if (sostituzione == "A108K") {valore_pssm = "-1";}
 if (sostituzione == "D136E") {valore_pssm = "-1";}
 if (sostituzione == "H186Q") {valore_pssm = "-1";}
 if (sostituzione == "S197I") {valore_pssm = "-1";}
 if (sostituzione == "F211M") {valore_pssm = "-1";}
 if (sostituzione == "Q212T") {valore_pssm = "-1";}
 if (sostituzione == "E218V") {valore_pssm = "-1";}
 if (sostituzione == "R220Y") {valore_pssm = "-1";}
 if (sostituzione == "Y222A") {valore_pssm = "-1";}
 if (sostituzione == "Y222K") {valore_pssm = "-1";}
 if (sostituzione == "A230L") {valore_pssm = "-1";}
 if (sostituzione == "S235K") {valore_pssm = "-1";}
 if (sostituzione == "E251F") {valore_pssm = "-1";}
 if (sostituzione == "R252G") {valore_pssm = "-1";}
 if (sostituzione == "V254N") {valore_pssm = "-1";}
 if (sostituzione == "M290T") {valore_pssm = "-1";}
 if (sostituzione == "A307F") {valore_pssm = "-1";}
 if (sostituzione == "A352C") {valore_pssm = "-1";}
 if (sostituzione == "V376T") {valore_pssm = "-1";}
 if (sostituzione == "T385V") {valore_pssm = "-1";}
 if (sostituzione == "T400V") {valore_pssm = "-1";}
 if (sostituzione == "R402Q") {valore_pssm = "-1";}
 if (sostituzione == "S405D") {valore_pssm = "-1";}
 if (sostituzione == "T410H") {valore_pssm = "-1";}
 if (sostituzione == "T412V") {valore_pssm = "-1";}
 if (sostituzione == "N419D") {valore_pssm = "-1";}
 if (sostituzione == "Q422H") {valore_pssm = "-1";}
 if (sostituzione == "A13N") {valore_pssm = "-1";}
 if (sostituzione == "D25T") {valore_pssm = "-1";}
 if (sostituzione == "D25R") {valore_pssm = "-1";}
 if (sostituzione == "P27C") {valore_pssm = "-1";}
 if (sostituzione == "N53E") {valore_pssm = "-1";}
 if (sostituzione == "D55Q") {valore_pssm = "-1";}
 if (sostituzione == "D55R") {valore_pssm = "-1";}
 if (sostituzione == "M72V") {valore_pssm = "-1";}
 if (sostituzione == "E74H") {valore_pssm = "-1";}
 if (sostituzione == "C90H") {valore_pssm = "-1";}
 if (sostituzione == "P98C") {valore_pssm = "-1";}
 if (sostituzione == "S126K") {valore_pssm = "-1";}
 if (sostituzione == "Y151Q") {valore_pssm = "-1";}
 if (sostituzione == "D153I") {valore_pssm = "-1";}
 if (sostituzione == "Q157R") {valore_pssm = "-1";}
 if (sostituzione == "L180Y") {valore_pssm = "-1";}
 if (sostituzione == "H186Y") {valore_pssm = "-1";}
 if (sostituzione == "S188V") {valore_pssm = "-1";}
 if (sostituzione == "S188Q") {valore_pssm = "-1";}
 if (sostituzione == "R196K") {valore_pssm = "-1";}
 if (sostituzione == "E203H") {valore_pssm = "-1";}
 if (sostituzione == "W209V") {valore_pssm = "-1";}
 if (sostituzione == "W209H") {valore_pssm = "-1";}
 if (sostituzione == "E218K") {valore_pssm = "-1";}
 if (sostituzione == "S235E") {valore_pssm = "-1";}
 if (sostituzione == "F248Q") {valore_pssm = "-1";}
 if (sostituzione == "Q250E") {valore_pssm = "-1";}
 if (sostituzione == "I253Y") {valore_pssm = "-1";}
 if (sostituzione == "W277P") {valore_pssm = "-1";}
 if (sostituzione == "L310C") {valore_pssm = "-1";}
 if (sostituzione == "K326Y") {valore_pssm = "-1";}
 if (sostituzione == "G360N") {valore_pssm = "-1";}
 if (sostituzione == "I367A") {valore_pssm = "-1";}
 if (sostituzione == "A377G") {valore_pssm = "-1";}
 if (sostituzione == "M1F") {valore_pssm = "-1";}
 if (sostituzione == "M1K") {valore_pssm = "-1";}
 if (sostituzione == "M1E") {valore_pssm = "-1";}
 if (sostituzione == "A20S") {valore_pssm = "-1";}
 if (sostituzione == "A31R") {valore_pssm = "-1";}
 if (sostituzione == "E79V") {valore_pssm = "-1";}
 if (sostituzione == "Q99R") {valore_pssm = "-1";}
 if (sostituzione == "R105K") {valore_pssm = "-1";}
 if (sostituzione == "F149H") {valore_pssm = "-1";}
 if (sostituzione == "P205E") {valore_pssm = "-1";}
 if (sostituzione == "P210F") {valore_pssm = "-1";}
 if (sostituzione == "Q212S") {valore_pssm = "-1";}
 if (sostituzione == "D234E") {valore_pssm = "-1";}
 if (sostituzione == "S241K") {valore_pssm = "-1";}
 if (sostituzione == "S241E") {valore_pssm = "-1";}
 if (sostituzione == "V281H") {valore_pssm = "-1";}
 if (sostituzione == "P305E") {valore_pssm = "-1";}
 if (sostituzione == "A309P") {valore_pssm = "-1";}
 if (sostituzione == "L324M") {valore_pssm = "-1";}
 if (sostituzione == "Q330G") {valore_pssm = "-1";}
 if (sostituzione == "Q333C") {valore_pssm = "-1";}
 if (sostituzione == "A348T") {valore_pssm = "-1";}
 if (sostituzione == "S371D") {valore_pssm = "-1";}
 if (sostituzione == "A377K") {valore_pssm = "-1";}
 if (sostituzione == "F396Y") {valore_pssm = "-1";}
 if (sostituzione == "S424G") {valore_pssm = "-1";}
 if (sostituzione == "S424C") {valore_pssm = "-1";}
 if (sostituzione == "L21C") {valore_pssm = "-1";}
 if (sostituzione == "T41M") {valore_pssm = "-1";}
 if (sostituzione == "E58R") {valore_pssm = "-1";}
 if (sostituzione == "S65C") {valore_pssm = "-1";}
 if (sostituzione == "A84K") {valore_pssm = "-1";}
 if (sostituzione == "A108E") {valore_pssm = "-1";}
 if (sostituzione == "I117L") {valore_pssm = "-1";}
 if (sostituzione == "L167M") {valore_pssm = "-1";}
 if (sostituzione == "D175C") {valore_pssm = "-1";}
 if (sostituzione == "Y207H") {valore_pssm = "-1";}
 if (sostituzione == "Y222C") {valore_pssm = "-1";}
 if (sostituzione == "Y222R") {valore_pssm = "-1";}
 if (sostituzione == "N228S") {valore_pssm = "-1";}
 if (sostituzione == "D244H") {valore_pssm = "-1";}
 if (sostituzione == "V269D") {valore_pssm = "-1";}
 if (sostituzione == "Q357H") {valore_pssm = "-1";}
 if (sostituzione == "G361A") {valore_pssm = "-1";}
 if (sostituzione == "T366S") {valore_pssm = "-1";}
 if (sostituzione == "A368Y") {valore_pssm = "-1";}
 if (sostituzione == "A370G") {valore_pssm = "-1";}
 if (sostituzione == "S371P") {valore_pssm = "-1";}
 if (sostituzione == "Q386R") {valore_pssm = "-1";}
 if (sostituzione == "T410N") {valore_pssm = "-1";}
 if (sostituzione == "L415C") {valore_pssm = "-1";}
 if (sostituzione == "N419K") {valore_pssm = "-1";}
 if (sostituzione == "V22Y") {valore_pssm = "-2";}
 if (sostituzione == "W24F") {valore_pssm = "-2";}
 if (sostituzione == "A29M") {valore_pssm = "-2";}
 if (sostituzione == "R30H") {valore_pssm = "-2";}
 if (sostituzione == "A37Q") {valore_pssm = "-2";}
 if (sostituzione == "A37R") {valore_pssm = "-2";}
 if (sostituzione == "E48K") {valore_pssm = "-2";}
 if (sostituzione == "E74N") {valore_pssm = "-2";}
 if (sostituzione == "P98N") {valore_pssm = "-2";}
 if (sostituzione == "D153N") {valore_pssm = "-2";}
 if (sostituzione == "C174T") {valore_pssm = "-2";}
 if (sostituzione == "S176M") {valore_pssm = "-2";}
 if (sostituzione == "I198L") {valore_pssm = "-2";}
 if (sostituzione == "I219Q") {valore_pssm = "-2";}
 if (sostituzione == "W245V") {valore_pssm = "-2";}
 if (sostituzione == "G258A") {valore_pssm = "-2";}
 if (sostituzione == "Q279R") {valore_pssm = "-2";}
 if (sostituzione == "Q280N") {valore_pssm = "-2";}
 if (sostituzione == "M284I") {valore_pssm = "-2";}
 if (sostituzione == "A285K") {valore_pssm = "-2";}
 if (sostituzione == "N298G") {valore_pssm = "-2";}
 if (sostituzione == "D315Y") {valore_pssm = "-2";}
 if (sostituzione == "A318V") {valore_pssm = "-2";}
 if (sostituzione == "A318R") {valore_pssm = "-2";}
 if (sostituzione == "Q330D") {valore_pssm = "-2";}
 if (sostituzione == "R332A") {valore_pssm = "-2";}
 if (sostituzione == "Q333M") {valore_pssm = "-2";}
 if (sostituzione == "G334A") {valore_pssm = "-2";}
 if (sostituzione == "F337K") {valore_pssm = "-2";}
 if (sostituzione == "F337R") {valore_pssm = "-2";}
 if (sostituzione == "G346C") {valore_pssm = "-2";}
 if (sostituzione == "K393N") {valore_pssm = "-2";}
 if (sostituzione == "R402S") {valore_pssm = "-2";}
 if (sostituzione == "I407T") {valore_pssm = "-2";}
 if (sostituzione == "T410R") {valore_pssm = "-2";}
 if (sostituzione == "M1R") {valore_pssm = "-2";}
 if (sostituzione == "L16T") {valore_pssm = "-2";}
 if (sostituzione == "R38K") {valore_pssm = "-2";}
 if (sostituzione == "N53G") {valore_pssm = "-2";}
 if (sostituzione == "S65P") {valore_pssm = "-2";}
 if (sostituzione == "E87A") {valore_pssm = "-2";}
 if (sostituzione == "M96K") {valore_pssm = "-2";}
 if (sostituzione == "Q111V") {valore_pssm = "-2";}
 if (sostituzione == "R118D") {valore_pssm = "-2";}
 if (sostituzione == "S148A") {valore_pssm = "-2";}
 if (sostituzione == "A181I") {valore_pssm = "-2";}
 if (sostituzione == "V199T") {valore_pssm = "-2";}
 if (sostituzione == "N215S") {valore_pssm = "-2";}
 if (sostituzione == "Y222E") {valore_pssm = "-2";}
 if (sostituzione == "V254Y") {valore_pssm = "-2";}
 if (sostituzione == "V254D") {valore_pssm = "-2";}
 if (sostituzione == "T282R") {valore_pssm = "-2";}
 if (sostituzione == "A318E") {valore_pssm = "-2";}
 if (sostituzione == "P323K") {valore_pssm = "-2";}
 if (sostituzione == "A377C") {valore_pssm = "-2";}
 if (sostituzione == "F383L") {valore_pssm = "-2";}
 if (sostituzione == "R4M") {valore_pssm = "-2";}
 if (sostituzione == "A15T") {valore_pssm = "-2";}
 if (sostituzione == "R17F") {valore_pssm = "-2";}
 if (sostituzione == "S23M") {valore_pssm = "-2";}
 if (sostituzione == "L32H") {valore_pssm = "-2";}
 if (sostituzione == "D33V") {valore_pssm = "-2";}
 if (sostituzione == "D33N") {valore_pssm = "-2";}
 if (sostituzione == "L45A") {valore_pssm = "-2";}
 if (sostituzione == "E59M") {valore_pssm = "-2";}
 if (sostituzione == "A84H") {valore_pssm = "-2";}
 if (sostituzione == "P110T") {valore_pssm = "-2";}
 if (sostituzione == "R112N") {valore_pssm = "-2";}
 if (sostituzione == "A143L") {valore_pssm = "-2";}
 if (sostituzione == "A143C") {valore_pssm = "-2";}
 if (sostituzione == "S176L") {valore_pssm = "-2";}
 if (sostituzione == "V199M") {valore_pssm = "-2";}
 if (sostituzione == "E203T") {valore_pssm = "-2";}
 if (sostituzione == "S241A") {valore_pssm = "-2";}
 if (sostituzione == "A257V") {valore_pssm = "-2";}
 if (sostituzione == "S276A") {valore_pssm = "-2";}
 if (sostituzione == "A285E") {valore_pssm = "-2";}
 if (sostituzione == "S297K") {valore_pssm = "-2";}
 if (sostituzione == "A309F") {valore_pssm = "-2";}
 if (sostituzione == "Q312K") {valore_pssm = "-2";}
 if (sostituzione == "L324F") {valore_pssm = "-2";}
 if (sostituzione == "F337E") {valore_pssm = "-2";}
 if (sostituzione == "G346F") {valore_pssm = "-2";}
 if (sostituzione == "I367M") {valore_pssm = "-2";}
 if (sostituzione == "A370C") {valore_pssm = "-2";}
 if (sostituzione == "A370H") {valore_pssm = "-2";}
 if (sostituzione == "A377E") {valore_pssm = "-2";}
 if (sostituzione == "N419E") {valore_pssm = "-2";}
 if (sostituzione == "L21K") {valore_pssm = "-2";}
 if (sostituzione == "I26F") {valore_pssm = "-2";}
 if (sostituzione == "L36M") {valore_pssm = "-2";}
 if (sostituzione == "H46T") {valore_pssm = "-2";}
 if (sostituzione == "M51F") {valore_pssm = "-2";}
 if (sostituzione == "E103A") {valore_pssm = "-2";}
 if (sostituzione == "A135F") {valore_pssm = "-2";}
 if (sostituzione == "P210C") {valore_pssm = "-2";}
 if (sostituzione == "T217S") {valore_pssm = "-2";}
 if (sostituzione == "E218I") {valore_pssm = "-2";}
 if (sostituzione == "Y222Q") {valore_pssm = "-2";}
 if (sostituzione == "N228K") {valore_pssm = "-2";}
 if (sostituzione == "L268F") {valore_pssm = "-2";}
 if (sostituzione == "L294M") {valore_pssm = "-2";}
 if (sostituzione == "S297H") {valore_pssm = "-2";}
 if (sostituzione == "D299E") {valore_pssm = "-2";}
 if (sostituzione == "Y329V") {valore_pssm = "-2";}
 if (sostituzione == "L344M") {valore_pssm = "-2";}
 if (sostituzione == "G360H") {valore_pssm = "-2";}
 if (sostituzione == "G361F") {valore_pssm = "-2";}
 if (sostituzione == "R363E") {valore_pssm = "-2";}
 if (sostituzione == "I367F") {valore_pssm = "-2";}
 if (sostituzione == "S371C") {valore_pssm = "-2";}
 if (sostituzione == "E398K") {valore_pssm = "-2";}
 if (sostituzione == "S401D") {valore_pssm = "-2";}
 if (sostituzione == "V413L") {valore_pssm = "-2";}
 if (sostituzione == "E418K") {valore_pssm = "-2";}
 if (sostituzione == "T41G") {valore_pssm = "-2";}
 if (sostituzione == "E48D") {valore_pssm = "-2";}
 if (sostituzione == "N53H") {valore_pssm = "-2";}
 if (sostituzione == "E58V") {valore_pssm = "-2";}
 if (sostituzione == "Y88F") {valore_pssm = "-2";}
 if (sostituzione == "Y123F") {valore_pssm = "-2";}
 if (sostituzione == "S126Q") {valore_pssm = "-2";}
 if (sostituzione == "L167F") {valore_pssm = "-2";}
 if (sostituzione == "L191F") {valore_pssm = "-2";}
 if (sostituzione == "W209E") {valore_pssm = "-2";}
 if (sostituzione == "D231E") {valore_pssm = "-2";}
 if (sostituzione == "S235Q") {valore_pssm = "-2";}
 if (sostituzione == "L243H") {valore_pssm = "-2";}
 if (sostituzione == "Q250S") {valore_pssm = "-2";}
 if (sostituzione == "V256L") {valore_pssm = "-2";}
 if (sostituzione == "M284L") {valore_pssm = "-2";}
 if (sostituzione == "S304A") {valore_pssm = "-2";}
 if (sostituzione == "A307M") {valore_pssm = "-2";}
 if (sostituzione == "L331H") {valore_pssm = "-2";}
 if (sostituzione == "N379D") {valore_pssm = "-2";}
 if (sostituzione == "A381P") {valore_pssm = "-2";}
 if (sostituzione == "Q386H") {valore_pssm = "-2";}
 if (sostituzione == "V390S") {valore_pssm = "-2";}
 if (sostituzione == "R402E") {valore_pssm = "-2";}
 if (sostituzione == "N408D") {valore_pssm = "-2";}
 if (sostituzione == "S23N") {valore_pssm = "-2";}
 if (sostituzione == "L54S") {valore_pssm = "-2";}
 if (sostituzione == "S65F") {valore_pssm = "-2";}
 if (sostituzione == "A84R") {valore_pssm = "-2";}
 if (sostituzione == "Q107T") {valore_pssm = "-2";}
 if (sostituzione == "K127S") {valore_pssm = "-2";}
 if (sostituzione == "G150H") {valore_pssm = "-2";}
 if (sostituzione == "D182L") {valore_pssm = "-2";}
 if (sostituzione == "P210I") {valore_pssm = "-2";}
 if (sostituzione == "Q212N") {valore_pssm = "-2";}
 if (sostituzione == "H225T") {valore_pssm = "-2";}
 if (sostituzione == "S241Q") {valore_pssm = "-2";}
 if (sostituzione == "D244K") {valore_pssm = "-2";}
 if (sostituzione == "T282C") {valore_pssm = "-2";}
 if (sostituzione == "A291G") {valore_pssm = "-2";}
 if (sostituzione == "Q306K") {valore_pssm = "-2";}
 if (sostituzione == "L331K") {valore_pssm = "-2";}
 if (sostituzione == "G334N") {valore_pssm = "-2";}
 if (sostituzione == "A352K") {valore_pssm = "-2";}
 if (sostituzione == "M353S") {valore_pssm = "-2";}
 if (sostituzione == "G360K") {valore_pssm = "-2";}
 if (sostituzione == "A370Q") {valore_pssm = "-2";}
 if (sostituzione == "A381I") {valore_pssm = "-2";}
 if (sostituzione == "S405F") {valore_pssm = "-2";}
 if (sostituzione == "N419T") {valore_pssm = "-2";}
 if (sostituzione == "L429D") {valore_pssm = "-2";}
 if (sostituzione == "G11L") {valore_pssm = "-2";}
 if (sostituzione == "A29P") {valore_pssm = "-2";}
 if (sostituzione == "L54P") {valore_pssm = "-2";}
 if (sostituzione == "S65I") {valore_pssm = "-2";}
 if (sostituzione == "L89C") {valore_pssm = "-2";}
 if (sostituzione == "M96E") {valore_pssm = "-2";}
 if (sostituzione == "Y151S") {valore_pssm = "-2";}
 if (sostituzione == "I154F") {valore_pssm = "-2";}
 if (sostituzione == "S176Y") {valore_pssm = "-2";}
 if (sostituzione == "R193G") {valore_pssm = "-2";}
 if (sostituzione == "W209Y") {valore_pssm = "-2";}
 if (sostituzione == "C223G") {valore_pssm = "-2";}
 if (sostituzione == "L243T") {valore_pssm = "-2";}
 if (sostituzione == "D244T") {valore_pssm = "-2";}
 if (sostituzione == "Q280I") {valore_pssm = "-2";}
 if (sostituzione == "I289S") {valore_pssm = "-2";}
 if (sostituzione == "N298D") {valore_pssm = "-2";}
 if (sostituzione == "H302G") {valore_pssm = "-2";}
 if (sostituzione == "A309M") {valore_pssm = "-2";}
 if (sostituzione == "A352M") {valore_pssm = "-2";}
 if (sostituzione == "E358V") {valore_pssm = "-2";}
 if (sostituzione == "T366V") {valore_pssm = "-2";}
 if (sostituzione == "N379K") {valore_pssm = "-2";}
 if (sostituzione == "A381N") {valore_pssm = "-2";}
 if (sostituzione == "R402V") {valore_pssm = "-2";}
 if (sostituzione == "N408K") {valore_pssm = "-2";}
 if (sostituzione == "N419Q") {valore_pssm = "-2";}
 if (sostituzione == "M1T") {valore_pssm = "-2";}
 if (sostituzione == "L8N") {valore_pssm = "-2";}
 if (sostituzione == "R30V") {valore_pssm = "-2";}
 if (sostituzione == "M51E") {valore_pssm = "-2";}
 if (sostituzione == "D55S") {valore_pssm = "-2";}
 if (sostituzione == "A84Q") {valore_pssm = "-2";}
 if (sostituzione == "Y86F") {valore_pssm = "-2";}
 if (sostituzione == "D92E") {valore_pssm = "-2";}
 if (sostituzione == "D93E") {valore_pssm = "-2";}
 if (sostituzione == "M96Q") {valore_pssm = "-2";}
 if (sostituzione == "S102R") {valore_pssm = "-2";}
 if (sostituzione == "Q107R") {valore_pssm = "-2";}
 if (sostituzione == "Q111N") {valore_pssm = "-2";}
 if (sostituzione == "Y134F") {valore_pssm = "-2";}
 if (sostituzione == "V137P") {valore_pssm = "-2";}
 if (sostituzione == "D155E") {valore_pssm = "-2";}
 if (sostituzione == "D165E") {valore_pssm = "-2";}
 if (sostituzione == "F169M") {valore_pssm = "-2";}
 if (sostituzione == "D170E") {valore_pssm = "-2";}
 if (sostituzione == "C174H") {valore_pssm = "-2";}
 if (sostituzione == "A181D") {valore_pssm = "-2";}
 if (sostituzione == "Y184F") {valore_pssm = "-2";}
 if (sostituzione == "Q212A") {valore_pssm = "-2";}
 if (sostituzione == "F229V") {valore_pssm = "-2";}
 if (sostituzione == "W245R") {valore_pssm = "-2";}
 if (sostituzione == "D264E") {valore_pssm = "-2";}
 if (sostituzione == "D266E") {valore_pssm = "-2";}
 if (sostituzione == "Q280D") {valore_pssm = "-2";}
 if (sostituzione == "T282G") {valore_pssm = "-2";}
 if (sostituzione == "M284V") {valore_pssm = "-2";}
 if (sostituzione == "S297E") {valore_pssm = "-2";}
 if (sostituzione == "L300T") {valore_pssm = "-2";}
 if (sostituzione == "P305S") {valore_pssm = "-2";}
 if (sostituzione == "L310A") {valore_pssm = "-2";}
 if (sostituzione == "D322E") {valore_pssm = "-2";}
 if (sostituzione == "W340L") {valore_pssm = "-2";}
 if (sostituzione == "S345E") {valore_pssm = "-2";}
 if (sostituzione == "A352F") {valore_pssm = "-2";}
 if (sostituzione == "A368L") {valore_pssm = "-2";}
 if (sostituzione == "I384M") {valore_pssm = "-2";}
 if (sostituzione == "L8P") {valore_pssm = "-2";}
 if (sostituzione == "L36F") {valore_pssm = "-2";}
 if (sostituzione == "M51I") {valore_pssm = "-2";}
 if (sostituzione == "K67I") {valore_pssm = "-2";}
 if (sostituzione == "S78G") {valore_pssm = "-2";}
 if (sostituzione == "M96H") {valore_pssm = "-2";}
 if (sostituzione == "A108H") {valore_pssm = "-2";}
 if (sostituzione == "G128Q") {valore_pssm = "-2";}
 if (sostituzione == "K130R") {valore_pssm = "-2";}
 if (sostituzione == "E178K") {valore_pssm = "-2";}
 if (sostituzione == "S188R") {valore_pssm = "-2";}
 if (sostituzione == "Q221L") {valore_pssm = "-2";}
 if (sostituzione == "A257T") {valore_pssm = "-2";}
 if (sostituzione == "V269S") {valore_pssm = "-2";}
 if (sostituzione == "L294F") {valore_pssm = "-2";}
 if (sostituzione == "F295Y") {valore_pssm = "-2";}
 if (sostituzione == "A307L") {valore_pssm = "-2";}
 if (sostituzione == "A318T") {valore_pssm = "-2";}
 if (sostituzione == "K326P") {valore_pssm = "-2";}
 if (sostituzione == "K326N") {valore_pssm = "-2";}
 if (sostituzione == "R332N") {valore_pssm = "-2";}
 if (sostituzione == "L344F") {valore_pssm = "-2";}
 if (sostituzione == "G360S") {valore_pssm = "-2";}
 if (sostituzione == "R402H") {valore_pssm = "-2";}
 if (sostituzione == "L415R") {valore_pssm = "-2";}
 if (sostituzione == "N419S") {valore_pssm = "-2";}
 if (sostituzione == "R4Q") {valore_pssm = "-2";}
 if (sostituzione == "L21H") {valore_pssm = "-2";}
 if (sostituzione == "D25N") {valore_pssm = "-2";}
 if (sostituzione == "M51T") {valore_pssm = "-2";}
 if (sostituzione == "A84E") {valore_pssm = "-2";}
 if (sostituzione == "E103V") {valore_pssm = "-2";}
 if (sostituzione == "F159Y") {valore_pssm = "-2";}
 if (sostituzione == "D161V") {valore_pssm = "-2";}
 if (sostituzione == "E178Q") {valore_pssm = "-2";}
 if (sostituzione == "N179C") {valore_pssm = "-2";}
 if (sostituzione == "K185E") {valore_pssm = "-2";}
 if (sostituzione == "H186P") {valore_pssm = "-2";}
 if (sostituzione == "N228E") {valore_pssm = "-2";}
 if (sostituzione == "A257E") {valore_pssm = "-2";}
 if (sostituzione == "A292V") {valore_pssm = "-2";}
 if (sostituzione == "A307Y") {valore_pssm = "-2";}
 if (sostituzione == "D313M") {valore_pssm = "-2";}
 if (sostituzione == "G325A") {valore_pssm = "-2";}
 if (sostituzione == "D335Q") {valore_pssm = "-2";}
 if (sostituzione == "F337C") {valore_pssm = "-2";}
 if (sostituzione == "G346I") {valore_pssm = "-2";}
 if (sostituzione == "G346Y") {valore_pssm = "-2";}
 if (sostituzione == "I354S") {valore_pssm = "-2";}
 if (sostituzione == "G361H") {valore_pssm = "-2";}
 if (sostituzione == "R363A") {valore_pssm = "-2";}
 if (sostituzione == "N379E") {valore_pssm = "-2";}
 if (sostituzione == "I384F") {valore_pssm = "-2";}
 if (sostituzione == "R392Q") {valore_pssm = "-2";}
 if (sostituzione == "K393S") {valore_pssm = "-2";}
 if (sostituzione == "N408E") {valore_pssm = "-2";}
 if (sostituzione == "L425T") {valore_pssm = "-2";}
 if (sostituzione == "L428T") {valore_pssm = "-2";}
 if (sostituzione == "E7N") {valore_pssm = "-2";}
 if (sostituzione == "L14C") {valore_pssm = "-2";}
 if (sostituzione == "A29N") {valore_pssm = "-2";}
 if (sostituzione == "A31G") {valore_pssm = "-2";}
 if (sostituzione == "T41C") {valore_pssm = "-2";}
 if (sostituzione == "E58D") {valore_pssm = "-2";}
 if (sostituzione == "S126V") {valore_pssm = "-2";}
 if (sostituzione == "K140D") {valore_pssm = "-2";}
 if (sostituzione == "A143F") {valore_pssm = "-2";}
 if (sostituzione == "Q157H") {valore_pssm = "-2";}
 if (sostituzione == "G171H") {valore_pssm = "-2";}
 if (sostituzione == "N179F") {valore_pssm = "-2";}
 if (sostituzione == "T194Y") {valore_pssm = "-2";}
 if (sostituzione == "V199H") {valore_pssm = "-2";}
 if (sostituzione == "W209T") {valore_pssm = "-2";}
 if (sostituzione == "F211R") {valore_pssm = "-2";}
 if (sostituzione == "I219Y") {valore_pssm = "-2";}
 if (sostituzione == "N228Q") {valore_pssm = "-2";}
 if (sostituzione == "D233I") {valore_pssm = "-2";}
 if (sostituzione == "Q250K") {valore_pssm = "-2";}
 if (sostituzione == "E251Y") {valore_pssm = "-2";}
 if (sostituzione == "A257K") {valore_pssm = "-2";}
 if (sostituzione == "Q279D") {valore_pssm = "-2";}
 if (sostituzione == "V281T") {valore_pssm = "-2";}
 if (sostituzione == "T282N") {valore_pssm = "-2";}
 if (sostituzione == "Q283K") {valore_pssm = "-2";}
 if (sostituzione == "Q283R") {valore_pssm = "-2";}
 if (sostituzione == "M290Y") {valore_pssm = "-2";}
 if (sostituzione == "H302C") {valore_pssm = "-2";}
 if (sostituzione == "S304Q") {valore_pssm = "-2";}
 if (sostituzione == "R363M") {valore_pssm = "-2";}
 if (sostituzione == "V390M") {valore_pssm = "-2";}
 if (sostituzione == "R404H") {valore_pssm = "-2";}
 if (sostituzione == "L415A") {valore_pssm = "-2";}
 if (sostituzione == "N419R") {valore_pssm = "-2";}
 if (sostituzione == "A13F") {valore_pssm = "-2";}
 if (sostituzione == "L21E") {valore_pssm = "-2";}
 if (sostituzione == "K67P") {valore_pssm = "-2";}
 if (sostituzione == "M72I") {valore_pssm = "-2";}
 if (sostituzione == "Q99T") {valore_pssm = "-2";}
 if (sostituzione == "N122I") {valore_pssm = "-2";}
 if (sostituzione == "K140N") {valore_pssm = "-2";}
 if (sostituzione == "Y152M") {valore_pssm = "-2";}
 if (sostituzione == "I154S") {valore_pssm = "-2";}
 if (sostituzione == "F211K") {valore_pssm = "-2";}
 if (sostituzione == "E218R") {valore_pssm = "-2";}
 if (sostituzione == "K237V") {valore_pssm = "-2";}
 if (sostituzione == "W245E") {valore_pssm = "-2";}
 if (sostituzione == "F248S") {valore_pssm = "-2";}
 if (sostituzione == "I253T") {valore_pssm = "-2";}
 if (sostituzione == "I270L") {valore_pssm = "-2";}
 if (sostituzione == "A285H") {valore_pssm = "-2";}
 if (sostituzione == "L331S") {valore_pssm = "-2";}
 if (sostituzione == "D335R") {valore_pssm = "-2";}
 if (sostituzione == "F337A") {valore_pssm = "-2";}
 if (sostituzione == "A352E") {valore_pssm = "-2";}
 if (sostituzione == "E358T") {valore_pssm = "-2";}
 if (sostituzione == "G361T") {valore_pssm = "-2";}
 if (sostituzione == "I367K") {valore_pssm = "-2";}
 if (sostituzione == "V376A") {valore_pssm = "-2";}
 if (sostituzione == "A377H") {valore_pssm = "-2";}
 if (sostituzione == "W399F") {valore_pssm = "-2";}
 if (sostituzione == "T410C") {valore_pssm = "-2";}
 if (sostituzione == "L429N") {valore_pssm = "-2";}
 if (sostituzione == "M1C") {valore_pssm = "-2";}
 if (sostituzione == "M1Q") {valore_pssm = "-2";}
 if (sostituzione == "L21R") {valore_pssm = "-2";}
 if (sostituzione == "I26T") {valore_pssm = "-2";}
 if (sostituzione == "K82I") {valore_pssm = "-2";}
 if (sostituzione == "M96C") {valore_pssm = "-2";}
 if (sostituzione == "A108Q") {valore_pssm = "-2";}
 if (sostituzione == "Y152P") {valore_pssm = "-2";}
 if (sostituzione == "E203R") {valore_pssm = "-2";}
 if (sostituzione == "W209Q") {valore_pssm = "-2";}
 if (sostituzione == "K240P") {valore_pssm = "-2";}
 if (sostituzione == "F248A") {valore_pssm = "-2";}
 if (sostituzione == "V256T") {valore_pssm = "-2";}
 if (sostituzione == "G261S") {valore_pssm = "-2";}
 if (sostituzione == "N298F") {valore_pssm = "-2";}
 if (sostituzione == "S304H") {valore_pssm = "-2";}
 if (sostituzione == "E341Y") {valore_pssm = "-2";}
 if (sostituzione == "G360A") {valore_pssm = "-2";}
 if (sostituzione == "G360Q") {valore_pssm = "-2";}
 if (sostituzione == "G361Q") {valore_pssm = "-2";}
 if (sostituzione == "V369C") {valore_pssm = "-2";}
 if (sostituzione == "N379T") {valore_pssm = "-2";}
 if (sostituzione == "R392T") {valore_pssm = "-2";}
 if (sostituzione == "N408T") {valore_pssm = "-2";}
 if (sostituzione == "S424D") {valore_pssm = "-2";}
 if (sostituzione == "R4F") {valore_pssm = "-2";}
 if (sostituzione == "N5V") {valore_pssm = "-2";}
 if (sostituzione == "A15G") {valore_pssm = "-2";}
 if (sostituzione == "A15K") {valore_pssm = "-2";}
 if (sostituzione == "A20T") {valore_pssm = "-2";}
 if (sostituzione == "L32Y") {valore_pssm = "-2";}
 if (sostituzione == "L32K") {valore_pssm = "-2";}
 if (sostituzione == "M70V") {valore_pssm = "-2";}
 if (sostituzione == "M70I") {valore_pssm = "-2";}
 if (sostituzione == "M96R") {valore_pssm = "-2";}
 if (sostituzione == "Y152N") {valore_pssm = "-2";}
 if (sostituzione == "G163D") {valore_pssm = "-2";}
 if (sostituzione == "K168R") {valore_pssm = "-2";}
 if (sostituzione == "F211E") {valore_pssm = "-2";}
 if (sostituzione == "N215D") {valore_pssm = "-2";}
 if (sostituzione == "S235V") {valore_pssm = "-2";}
 if (sostituzione == "K240N") {valore_pssm = "-2";}
 if (sostituzione == "N272V") {valore_pssm = "-2";}
 if (sostituzione == "M290C") {valore_pssm = "-2";}
 if (sostituzione == "K308Q") {valore_pssm = "-2";}
 if (sostituzione == "L331R") {valore_pssm = "-2";}
 if (sostituzione == "L331E") {valore_pssm = "-2";}
 if (sostituzione == "E358S") {valore_pssm = "-2";}
 if (sostituzione == "A370R") {valore_pssm = "-2";}
 if (sostituzione == "N379Q") {valore_pssm = "-2";}
 if (sostituzione == "V390F") {valore_pssm = "-2";}
 if (sostituzione == "K393T") {valore_pssm = "-2";}
 if (sostituzione == "S405M") {valore_pssm = "-2";}
 if (sostituzione == "N408Q") {valore_pssm = "-2";}
 if (sostituzione == "M421Q") {valore_pssm = "-2";}
 if (sostituzione == "M423Q") {valore_pssm = "-2";}
 if (sostituzione == "Q2T") {valore_pssm = "-2";}
 if (sostituzione == "L16C") {valore_pssm = "-2";}
 if (sostituzione == "F18Y") {valore_pssm = "-2";}
 if (sostituzione == "W24Y") {valore_pssm = "-2";}
 if (sostituzione == "A31C") {valore_pssm = "-2";}
 if (sostituzione == "K67G") {valore_pssm = "-2";}
 if (sostituzione == "S102G") {valore_pssm = "-2";}
 if (sostituzione == "S102C") {valore_pssm = "-2";}
 if (sostituzione == "S102N") {valore_pssm = "-2";}
 if (sostituzione == "A135M") {valore_pssm = "-2";}
 if (sostituzione == "N139D") {valore_pssm = "-2";}
 if (sostituzione == "L180G") {valore_pssm = "-2";}
 if (sostituzione == "Y200H") {valore_pssm = "-2";}
 if (sostituzione == "S235R") {valore_pssm = "-2";}
 if (sostituzione == "S241R") {valore_pssm = "-2";}
 if (sostituzione == "Q250H") {valore_pssm = "-2";}
 if (sostituzione == "A285Q") {valore_pssm = "-2";}
 if (sostituzione == "S297Q") {valore_pssm = "-2";}
 if (sostituzione == "H302P") {valore_pssm = "-2";}
 if (sostituzione == "L310H") {valore_pssm = "-2";}
 if (sostituzione == "K314V") {valore_pssm = "-2";}
 if (sostituzione == "G361K") {valore_pssm = "-2";}
 if (sostituzione == "R363S") {valore_pssm = "-2";}
 if (sostituzione == "V369A") {valore_pssm = "-2";}
 if (sostituzione == "A377Q") {valore_pssm = "-2";}
 if (sostituzione == "S405L") {valore_pssm = "-2";}
 if (sostituzione == "L3T") {valore_pssm = "-2";}
 if (sostituzione == "N5C") {valore_pssm = "-2";}
 if (sostituzione == "L10T") {valore_pssm = "-2";}
 if (sostituzione == "L21Q") {valore_pssm = "-2";}
 if (sostituzione == "V22M") {valore_pssm = "-2";}
 if (sostituzione == "L32R") {valore_pssm = "-2";}
 if (sostituzione == "L45G") {valore_pssm = "-2";}
 if (sostituzione == "M51L") {valore_pssm = "-2";}
 if (sostituzione == "E58T") {valore_pssm = "-2";}
 if (sostituzione == "S78P") {valore_pssm = "-2";}
 if (sostituzione == "P98D") {valore_pssm = "-2";}
 if (sostituzione == "Q99A") {valore_pssm = "-2";}
 if (sostituzione == "R118I") {valore_pssm = "-2";}
 if (sostituzione == "A135Y") {valore_pssm = "-2";}
 if (sostituzione == "E178S") {valore_pssm = "-2";}
 if (sostituzione == "A181C") {valore_pssm = "-2";}
 if (sostituzione == "S197F") {valore_pssm = "-2";}
 if (sostituzione == "Y216H") {valore_pssm = "-2";}
 if (sostituzione == "N228R") {valore_pssm = "-2";}
 if (sostituzione == "Q250A") {valore_pssm = "-2";}
 if (sostituzione == "E251L") {valore_pssm = "-2";}
 if (sostituzione == "R301K") {valore_pssm = "-2";}
 if (sostituzione == "Q333L") {valore_pssm = "-2";}
 if (sostituzione == "N379S") {valore_pssm = "-2";}
 if (sostituzione == "L387T") {valore_pssm = "-2";}
 if (sostituzione == "L388T") {valore_pssm = "-2";}
 if (sostituzione == "L394T") {valore_pssm = "-2";}
 if (sostituzione == "E398D") {valore_pssm = "-2";}
 if (sostituzione == "W399Y") {valore_pssm = "-2";}
 if (sostituzione == "L403T") {valore_pssm = "-2";}
 if (sostituzione == "N408S") {valore_pssm = "-2";}
 if (sostituzione == "L414T") {valore_pssm = "-2";}
 if (sostituzione == "L415K") {valore_pssm = "-2";}
 if (sostituzione == "L417T") {valore_pssm = "-2";}
 if (sostituzione == "E418D") {valore_pssm = "-2";}
 if (sostituzione == "S424P") {valore_pssm = "-2";}
 if (sostituzione == "D427N") {valore_pssm = "-2";}
 if (sostituzione == "E7R") {valore_pssm = "-2";}
 if (sostituzione == "G11F") {valore_pssm = "-2";}
 if (sostituzione == "A15C") {valore_pssm = "-2";}
 if (sostituzione == "L32E") {valore_pssm = "-2";}
 if (sostituzione == "E74S") {valore_pssm = "-2";}
 if (sostituzione == "E79I") {valore_pssm = "-2";}
 if (sostituzione == "L89A") {valore_pssm = "-2";}
 if (sostituzione == "C90T") {valore_pssm = "-2";}
 if (sostituzione == "V137Y") {valore_pssm = "-2";}
 if (sostituzione == "C174K") {valore_pssm = "-2";}
 if (sostituzione == "C174E") {valore_pssm = "-2";}
 if (sostituzione == "D175L") {valore_pssm = "-2";}
 if (sostituzione == "D182C") {valore_pssm = "-2";}
 if (sostituzione == "N192H") {valore_pssm = "-2";}
 if (sostituzione == "S201A") {valore_pssm = "-2";}
 if (sostituzione == "I219P") {valore_pssm = "-2";}
 if (sostituzione == "D234S") {valore_pssm = "-2";}
 if (sostituzione == "S238A") {valore_pssm = "-2";}
 if (sostituzione == "E251M") {valore_pssm = "-2";}
 if (sostituzione == "I289M") {valore_pssm = "-2";}
 if (sostituzione == "S304R") {valore_pssm = "-2";}
 if (sostituzione == "A350S") {valore_pssm = "-2";}
 if (sostituzione == "V376C") {valore_pssm = "-2";}
 if (sostituzione == "A381F") {valore_pssm = "-2";}
 if (sostituzione == "T410I") {valore_pssm = "-2";}
 if (sostituzione == "G11S") {valore_pssm = "-2";}
 if (sostituzione == "A13M") {valore_pssm = "-2";}
 if (sostituzione == "A15E") {valore_pssm = "-2";}
 if (sostituzione == "A31I") {valore_pssm = "-2";}
 if (sostituzione == "T41Y") {valore_pssm = "-2";}
 if (sostituzione == "L45C") {valore_pssm = "-2";}
 if (sostituzione == "E79A") {valore_pssm = "-2";}
 if (sostituzione == "D109H") {valore_pssm = "-2";}
 if (sostituzione == "S126R") {valore_pssm = "-2";}
 if (sostituzione == "D161G") {valore_pssm = "-2";}
 if (sostituzione == "D161P") {valore_pssm = "-2";}
 if (sostituzione == "L166P") {valore_pssm = "-2";}
 if (sostituzione == "D175Y") {valore_pssm = "-2";}
 if (sostituzione == "R193P") {valore_pssm = "-2";}
 if (sostituzione == "W245Q") {valore_pssm = "-2";}
 if (sostituzione == "Q280M") {valore_pssm = "-2";}
 if (sostituzione == "P305G") {valore_pssm = "-2";}
 if (sostituzione == "Q312R") {valore_pssm = "-2";}
 if (sostituzione == "K326D") {valore_pssm = "-2";}
 if (sostituzione == "S345K") {valore_pssm = "-2";}
 if (sostituzione == "I367E") {valore_pssm = "-2";}
 if (sostituzione == "N379R") {valore_pssm = "-2";}
 if (sostituzione == "N408R") {valore_pssm = "-2";}
 if (sostituzione == "M421T") {valore_pssm = "-2";}
 if (sostituzione == "M423T") {valore_pssm = "-2";}
 if (sostituzione == "R4H") {valore_pssm = "-2";}
 if (sostituzione == "S23D") {valore_pssm = "-2";}
 if (sostituzione == "T41L") {valore_pssm = "-2";}
 if (sostituzione == "D55V") {valore_pssm = "-2";}
 if (sostituzione == "A73V") {valore_pssm = "-2";}
 if (sostituzione == "A121V") {valore_pssm = "-2";}
 if (sostituzione == "I154A") {valore_pssm = "-2";}
 if (sostituzione == "A160V") {valore_pssm = "-2";}
 if (sostituzione == "L166N") {valore_pssm = "-2";}
 if (sostituzione == "C174V") {valore_pssm = "-2";}
 if (sostituzione == "G183A") {valore_pssm = "-2";}
 if (sostituzione == "R193D") {valore_pssm = "-2";}
 if (sostituzione == "F211Q") {valore_pssm = "-2";}
 if (sostituzione == "A285G") {valore_pssm = "-2";}
 if (sostituzione == "A285C") {valore_pssm = "-2";}
 if (sostituzione == "A292T") {valore_pssm = "-2";}
 if (sostituzione == "M296A") {valore_pssm = "-2";}
 if (sostituzione == "H302I") {valore_pssm = "-2";}
 if (sostituzione == "Q306A") {valore_pssm = "-2";}
 if (sostituzione == "Q312H") {valore_pssm = "-2";}
 if (sostituzione == "A318H") {valore_pssm = "-2";}
 if (sostituzione == "L331Q") {valore_pssm = "-2";}
 if (sostituzione == "G334V") {valore_pssm = "-2";}
 if (sostituzione == "A350T") {valore_pssm = "-2";}
 if (sostituzione == "A352H") {valore_pssm = "-2";}
 if (sostituzione == "N355H") {valore_pssm = "-2";}
 if (sostituzione == "I367C") {valore_pssm = "-2";}
 if (sostituzione == "S371I") {valore_pssm = "-2";}
 if (sostituzione == "Q2S") {valore_pssm = "-2";}
 if (sostituzione == "L32A") {valore_pssm = "-2";}
 if (sostituzione == "L32C") {valore_pssm = "-2";}
 if (sostituzione == "T41F") {valore_pssm = "-2";}
 if (sostituzione == "E48S") {valore_pssm = "-2";}
 if (sostituzione == "W81Y") {valore_pssm = "-2";}
 if (sostituzione == "A97D") {valore_pssm = "-2";}
 if (sostituzione == "Q99D") {valore_pssm = "-2";}
 if (sostituzione == "R112Q") {valore_pssm = "-2";}
 if (sostituzione == "N122F") {valore_pssm = "-2";}
 if (sostituzione == "K127Q") {valore_pssm = "-2";}
 if (sostituzione == "S148K") {valore_pssm = "-2";}
 if (sostituzione == "S148E") {valore_pssm = "-2";}
 if (sostituzione == "N179P") {valore_pssm = "-2";}
 if (sostituzione == "S188C") {valore_pssm = "-2";}
 if (sostituzione == "D244Q") {valore_pssm = "-2";}
 if (sostituzione == "G271D") {valore_pssm = "-2";}
 if (sostituzione == "S276K") {valore_pssm = "-2";}
 if (sostituzione == "S276E") {valore_pssm = "-2";}
 if (sostituzione == "S297N") {valore_pssm = "-2";}
 if (sostituzione == "N298L") {valore_pssm = "-2";}
 if (sostituzione == "Q306H") {valore_pssm = "-2";}
 if (sostituzione == "K308E") {valore_pssm = "-2";}
 if (sostituzione == "P323V") {valore_pssm = "-2";}
 if (sostituzione == "G346M") {valore_pssm = "-2";}
 if (sostituzione == "A348G") {valore_pssm = "-2";}
 if (sostituzione == "S371F") {valore_pssm = "-2";}
 if (sostituzione == "A377I") {valore_pssm = "-2";}
 if (sostituzione == "V413T") {valore_pssm = "-2";}
 if (sostituzione == "Q416T") {valore_pssm = "-2";}
 if (sostituzione == "L425C") {valore_pssm = "-2";}
 if (sostituzione == "L428C") {valore_pssm = "-2";}
 if (sostituzione == "G11K") {valore_pssm = "-2";}
 if (sostituzione == "A13L") {valore_pssm = "-2";}
 if (sostituzione == "A13Y") {valore_pssm = "-2";}
 if (sostituzione == "A20Y") {valore_pssm = "-2";}
 if (sostituzione == "M51S") {valore_pssm = "-2";}
 if (sostituzione == "E58S") {valore_pssm = "-2";}
 if (sostituzione == "P60V") {valore_pssm = "-2";}
 if (sostituzione == "P60H") {valore_pssm = "-2";}
 if (sostituzione == "S78C") {valore_pssm = "-2";}
 if (sostituzione == "D101H") {valore_pssm = "-2";}
 if (sostituzione == "K127V") {valore_pssm = "-2";}
 if (sostituzione == "L129Y") {valore_pssm = "-2";}
 if (sostituzione == "K140C") {valore_pssm = "-2";}
 if (sostituzione == "G150K") {valore_pssm = "-2";}
 if (sostituzione == "A181L") {valore_pssm = "-2";}
 if (sostituzione == "V199C") {valore_pssm = "-2";}
 if (sostituzione == "A257H") {valore_pssm = "-2";}
 if (sostituzione == "L275T") {valore_pssm = "-2";}
 if (sostituzione == "Q280P") {valore_pssm = "-2";}
 if (sostituzione == "T282I") {valore_pssm = "-2";}
 if (sostituzione == "N298P") {valore_pssm = "-2";}
 if (sostituzione == "A352Q") {valore_pssm = "-2";}
 if (sostituzione == "E358P") {valore_pssm = "-2";}
 if (sostituzione == "A381D") {valore_pssm = "-2";}
 if (sostituzione == "R392H") {valore_pssm = "-2";}
 if (sostituzione == "R392E") {valore_pssm = "-2";}
 if (sostituzione == "S405Y") {valore_pssm = "-2";}
 if (sostituzione == "E7S") {valore_pssm = "-2";}
 if (sostituzione == "G11C") {valore_pssm = "-2";}
 if (sostituzione == "V22A") {valore_pssm = "-2";}
 if (sostituzione == "N53K") {valore_pssm = "-2";}
 if (sostituzione == "D83A") {valore_pssm = "-2";}
 if (sostituzione == "A143N") {valore_pssm = "-2";}
 if (sostituzione == "G144N") {valore_pssm = "-2";}
 if (sostituzione == "E178V") {valore_pssm = "-2";}
 if (sostituzione == "N179L") {valore_pssm = "-2";}
 if (sostituzione == "E203A") {valore_pssm = "-2";}
 if (sostituzione == "F211A") {valore_pssm = "-2";}
 if (sostituzione == "F211S") {valore_pssm = "-2";}
 if (sostituzione == "F211C") {valore_pssm = "-2";}
 if (sostituzione == "K213S") {valore_pssm = "-2";}
 if (sostituzione == "P214I") {valore_pssm = "-2";}
 if (sostituzione == "Y216I") {valore_pssm = "-2";}
 if (sostituzione == "Y222S") {valore_pssm = "-2";}
 if (sostituzione == "H225E") {valore_pssm = "-2";}
 if (sostituzione == "D231G") {valore_pssm = "-2";}
 if (sostituzione == "K237W") {valore_pssm = "-2";}
 if (sostituzione == "V269C") {valore_pssm = "-2";}
 if (sostituzione == "Q280G") {valore_pssm = "-2";}
 if (sostituzione == "Q280C") {valore_pssm = "-2";}
 if (sostituzione == "Q283Y") {valore_pssm = "-2";}
 if (sostituzione == "A285R") {valore_pssm = "-2";}
 if (sostituzione == "A288K") {valore_pssm = "-2";}
 if (sostituzione == "A291P") {valore_pssm = "-2";}
 if (sostituzione == "A291C") {valore_pssm = "-2";}
 if (sostituzione == "M296F") {valore_pssm = "-2";}
 if (sostituzione == "Q306R") {valore_pssm = "-2";}
 if (sostituzione == "R332V") {valore_pssm = "-2";}
 if (sostituzione == "P343R") {valore_pssm = "-2";}
 if (sostituzione == "S345H") {valore_pssm = "-2";}
 if (sostituzione == "A348N") {valore_pssm = "-2";}
 if (sostituzione == "E358A") {valore_pssm = "-2";}
 if (sostituzione == "G360T") {valore_pssm = "-2";}
 if (sostituzione == "R392L") {valore_pssm = "-2";}
 if (sostituzione == "G11M") {valore_pssm = "-2";}
 if (sostituzione == "W24V") {valore_pssm = "-2";}
 if (sostituzione == "G35S") {valore_pssm = "-2";}
 if (sostituzione == "K67L") {valore_pssm = "-2";}
 if (sostituzione == "A84Y") {valore_pssm = "-2";}
 if (sostituzione == "R118M") {valore_pssm = "-2";}
 if (sostituzione == "A135L") {valore_pssm = "-2";}
 if (sostituzione == "G150S") {valore_pssm = "-2";}
 if (sostituzione == "D175M") {valore_pssm = "-2";}
 if (sostituzione == "L180N") {valore_pssm = "-2";}
 if (sostituzione == "G183S") {valore_pssm = "-2";}
 if (sostituzione == "K213T") {valore_pssm = "-2";}
 if (sostituzione == "D233P") {valore_pssm = "-2";}
 if (sostituzione == "I239F") {valore_pssm = "-2";}
 if (sostituzione == "S241D") {valore_pssm = "-2";}
 if (sostituzione == "F248C") {valore_pssm = "-2";}
 if (sostituzione == "Q250R") {valore_pssm = "-2";}
 if (sostituzione == "V269Y") {valore_pssm = "-2";}
 if (sostituzione == "M290K") {valore_pssm = "-2";}
 if (sostituzione == "D313L") {valore_pssm = "-2";}
 if (sostituzione == "Q327P") {valore_pssm = "-2";}
 if (sostituzione == "A352R") {valore_pssm = "-2";}
 if (sostituzione == "T366E") {valore_pssm = "-2";}
 if (sostituzione == "A377R") {valore_pssm = "-2";}
 if (sostituzione == "E398H") {valore_pssm = "-2";}
 if (sostituzione == "E418H") {valore_pssm = "-2";}
 if (sostituzione == "T420K") {valore_pssm = "-2";}
 if (sostituzione == "A20I") {valore_pssm = "-2";}
 if (sostituzione == "A20H") {valore_pssm = "-2";}
 if (sostituzione == "A29Y") {valore_pssm = "-2";}
 if (sostituzione == "R30F") {valore_pssm = "-2";}
 if (sostituzione == "E48R") {valore_pssm = "-2";}
 if (sostituzione == "L54G") {valore_pssm = "-2";}
 if (sostituzione == "E58P") {valore_pssm = "-2";}
 if (sostituzione == "P60E") {valore_pssm = "-2";}
 if (sostituzione == "K82M") {valore_pssm = "-2";}
 if (sostituzione == "Y88R") {valore_pssm = "-2";}
 if (sostituzione == "A108G") {valore_pssm = "-2";}
 if (sostituzione == "A108R") {valore_pssm = "-2";}
 if (sostituzione == "S126G") {valore_pssm = "-2";}
 if (sostituzione == "K140P") {valore_pssm = "-2";}
 if (sostituzione == "D153P") {valore_pssm = "-2";}
 if (sostituzione == "L180D") {valore_pssm = "-2";}
 if (sostituzione == "S188N") {valore_pssm = "-2";}
 if (sostituzione == "A190S") {valore_pssm = "-2";}
 if (sostituzione == "Q212V") {valore_pssm = "-2";}
 if (sostituzione == "E218T") {valore_pssm = "-2";}
 if (sostituzione == "N228L") {valore_pssm = "-2";}
 if (sostituzione == "W245I") {valore_pssm = "-2";}
 if (sostituzione == "T282P") {valore_pssm = "-2";}
 if (sostituzione == "I289F") {valore_pssm = "-2";}
 if (sostituzione == "M290H") {valore_pssm = "-2";}
 if (sostituzione == "K314D") {valore_pssm = "-2";}
 if (sostituzione == "W349C") {valore_pssm = "-2";}
 if (sostituzione == "T366K") {valore_pssm = "-2";}
 if (sostituzione == "K426H") {valore_pssm = "-2";}
 if (sostituzione == "R17Y") {valore_pssm = "-2";}
 if (sostituzione == "P27G") {valore_pssm = "-2";}
 if (sostituzione == "A29D") {valore_pssm = "-2";}
 if (sostituzione == "L32Q") {valore_pssm = "-2";}
 if (sostituzione == "D33P") {valore_pssm = "-2";}
 if (sostituzione == "H46E") {valore_pssm = "-2";}
 if (sostituzione == "N53T") {valore_pssm = "-2";}
 if (sostituzione == "S65Y") {valore_pssm = "-2";}
 if (sostituzione == "A108C") {valore_pssm = "-2";}
 if (sostituzione == "R112E") {valore_pssm = "-2";}
 if (sostituzione == "K127E") {valore_pssm = "-2";}
 if (sostituzione == "A143P") {valore_pssm = "-2";}
 if (sostituzione == "R193I") {valore_pssm = "-2";}
 if (sostituzione == "R193C") {valore_pssm = "-2";}
 if (sostituzione == "S197L") {valore_pssm = "-2";}
 if (sostituzione == "V199S") {valore_pssm = "-2";}
 if (sostituzione == "T217V") {valore_pssm = "-2";}
 if (sostituzione == "H225A") {valore_pssm = "-2";}
 if (sostituzione == "H225V") {valore_pssm = "-2";}
 if (sostituzione == "L243C") {valore_pssm = "-2";}
 if (sostituzione == "W245T") {valore_pssm = "-2";}
 if (sostituzione == "V281A") {valore_pssm = "-2";}
 if (sostituzione == "S297R") {valore_pssm = "-2";}
 if (sostituzione == "I303F") {valore_pssm = "-2";}
 if (sostituzione == "S304N") {valore_pssm = "-2";}
 if (sostituzione == "D335A") {valore_pssm = "-2";}
 if (sostituzione == "W349N") {valore_pssm = "-2";}
 if (sostituzione == "G361S") {valore_pssm = "-2";}
 if (sostituzione == "A370I") {valore_pssm = "-2";}
 if (sostituzione == "S371Y") {valore_pssm = "-2";}
 if (sostituzione == "K374T") {valore_pssm = "-2";}
 if (sostituzione == "T410G") {valore_pssm = "-2";}
 if (sostituzione == "L415E") {valore_pssm = "-2";}
 if (sostituzione == "L3C") {valore_pssm = "-2";}
 if (sostituzione == "R4E") {valore_pssm = "-2";}
 if (sostituzione == "L10C") {valore_pssm = "-2";}
 if (sostituzione == "G11H") {valore_pssm = "-2";}
 if (sostituzione == "L14A") {valore_pssm = "-2";}
 if (sostituzione == "L14Y") {valore_pssm = "-2";}
 if (sostituzione == "R17N") {valore_pssm = "-2";}
 if (sostituzione == "P27N") {valore_pssm = "-2";}
 if (sostituzione == "A31D") {valore_pssm = "-2";}
 if (sostituzione == "E58A") {valore_pssm = "-2";}
 if (sostituzione == "E58I") {valore_pssm = "-2";}
 if (sostituzione == "E58F") {valore_pssm = "-2";}
 if (sostituzione == "P60K") {valore_pssm = "-2";}
 if (sostituzione == "I64L") {valore_pssm = "-2";}
 if (sostituzione == "K67F") {valore_pssm = "-2";}
 if (sostituzione == "K67Y") {valore_pssm = "-2";}
 if (sostituzione == "A97C") {valore_pssm = "-2";}
 if (sostituzione == "E103G") {valore_pssm = "-2";}
 if (sostituzione == "R118C") {valore_pssm = "-2";}
 if (sostituzione == "S148V") {valore_pssm = "-2";}
 if (sostituzione == "S148H") {valore_pssm = "-2";}
 if (sostituzione == "A156S") {valore_pssm = "-2";}
 if (sostituzione == "L166G") {valore_pssm = "-2";}
 if (sostituzione == "N179M") {valore_pssm = "-2";}
 if (sostituzione == "A181N") {valore_pssm = "-2";}
 if (sostituzione == "Q280L") {valore_pssm = "-2";}
 if (sostituzione == "A288E") {valore_pssm = "-2";}
 if (sostituzione == "M290Q") {valore_pssm = "-2";}
 if (sostituzione == "A291D") {valore_pssm = "-2";}
 if (sostituzione == "P305R") {valore_pssm = "-2";}
 if (sostituzione == "L310K") {valore_pssm = "-2";}
 if (sostituzione == "Q327E") {valore_pssm = "-2";}
 if (sostituzione == "R332D") {valore_pssm = "-2";}
 if (sostituzione == "S345Q") {valore_pssm = "-2";}
 if (sostituzione == "I367H") {valore_pssm = "-2";}
 if (sostituzione == "L387C") {valore_pssm = "-2";}
 if (sostituzione == "L388C") {valore_pssm = "-2";}
 if (sostituzione == "L394C") {valore_pssm = "-2";}
 if (sostituzione == "L403C") {valore_pssm = "-2";}
 if (sostituzione == "I407A") {valore_pssm = "-2";}
 if (sostituzione == "L414C") {valore_pssm = "-2";}
 if (sostituzione == "L417C") {valore_pssm = "-2";}
 if (sostituzione == "T420I") {valore_pssm = "-2";}
 if (sostituzione == "D427K") {valore_pssm = "-2";}
 if (sostituzione == "N5P") {valore_pssm = "-2";}
 if (sostituzione == "A15H") {valore_pssm = "-2";}
 if (sostituzione == "L16K") {valore_pssm = "-2";}
 if (sostituzione == "A20K") {valore_pssm = "-2";}
 if (sostituzione == "R30Q") {valore_pssm = "-2";}
 if (sostituzione == "A37P") {valore_pssm = "-2";}
 if (sostituzione == "H46K") {valore_pssm = "-2";}
 if (sostituzione == "K67C") {valore_pssm = "-2";}
 if (sostituzione == "L89Y") {valore_pssm = "-2";}
 if (sostituzione == "K127I") {valore_pssm = "-2";}
 if (sostituzione == "Y152C") {valore_pssm = "-2";}
 if (sostituzione == "Y173N") {valore_pssm = "-2";}
 if (sostituzione == "S197Y") {valore_pssm = "-2";}
 if (sostituzione == "W209S") {valore_pssm = "-2";}
 if (sostituzione == "P210Y") {valore_pssm = "-2";}
 if (sostituzione == "D244R") {valore_pssm = "-2";}
 if (sostituzione == "I253C") {valore_pssm = "-2";}
 if (sostituzione == "G274N") {valore_pssm = "-2";}
 if (sostituzione == "S276H") {valore_pssm = "-2";}
 if (sostituzione == "L300C") {valore_pssm = "-2";}
 if (sostituzione == "A309Y") {valore_pssm = "-2";}
 if (sostituzione == "W349L") {valore_pssm = "-2";}
 if (sostituzione == "T366Q") {valore_pssm = "-2";}
 if (sostituzione == "I367Q") {valore_pssm = "-2";}
 if (sostituzione == "S371M") {valore_pssm = "-2";}
 if (sostituzione == "K374S") {valore_pssm = "-2";}
 if (sostituzione == "K393A") {valore_pssm = "-2";}
 if (sostituzione == "L415Q") {valore_pssm = "-2";}
 if (sostituzione == "Q416S") {valore_pssm = "-2";}
 if (sostituzione == "P6Q") {valore_pssm = "-2";}
 if (sostituzione == "L8G") {valore_pssm = "-2";}
 if (sostituzione == "A20C") {valore_pssm = "-2";}
 if (sostituzione == "D25A") {valore_pssm = "-2";}
 if (sostituzione == "D33I") {valore_pssm = "-2";}
 if (sostituzione == "L45Y") {valore_pssm = "-2";}
 if (sostituzione == "H46Q") {valore_pssm = "-2";}
 if (sostituzione == "N53Q") {valore_pssm = "-2";}
 if (sostituzione == "M72L") {valore_pssm = "-2";}
 if (sostituzione == "A73S") {valore_pssm = "-2";}
 if (sostituzione == "E74T") {valore_pssm = "-2";}
 if (sostituzione == "C90K") {valore_pssm = "-2";}
 if (sostituzione == "R118Y") {valore_pssm = "-2";}
 if (sostituzione == "A121S") {valore_pssm = "-2";}
 if (sostituzione == "Y123H") {valore_pssm = "-2";}
 if (sostituzione == "I133L") {valore_pssm = "-2";}
 if (sostituzione == "S148Q") {valore_pssm = "-2";}
 if (sostituzione == "A160S") {valore_pssm = "-2";}
 if (sostituzione == "C174Q") {valore_pssm = "-2";}
 if (sostituzione == "G183T") {valore_pssm = "-2";}
 if (sostituzione == "N215F") {valore_pssm = "-2";}
 if (sostituzione == "W226I") {valore_pssm = "-2";}
 if (sostituzione == "I253H") {valore_pssm = "-2";}
 if (sostituzione == "S276V") {valore_pssm = "-2";}
 if (sostituzione == "M290R") {valore_pssm = "-2";}
 if (sostituzione == "I359A") {valore_pssm = "-2";}
 if (sostituzione == "I359S") {valore_pssm = "-2";}
 if (sostituzione == "I367R") {valore_pssm = "-2";}
 if (sostituzione == "V390C") {valore_pssm = "-2";}
 if (sostituzione == "I407C") {valore_pssm = "-2";}
 if (sostituzione == "T410P") {valore_pssm = "-2";}
 if (sostituzione == "T410D") {valore_pssm = "-2";}
 if (sostituzione == "M1A") {valore_pssm = "-2";}
 if (sostituzione == "L8D") {valore_pssm = "-2";}
 if (sostituzione == "L16S") {valore_pssm = "-2";}
 if (sostituzione == "L16H") {valore_pssm = "-2";}
 if (sostituzione == "N34H") {valore_pssm = "-2";}
 if (sostituzione == "E87P") {valore_pssm = "-2";}
 if (sostituzione == "E87M") {valore_pssm = "-2";}
 if (sostituzione == "C90D") {valore_pssm = "-2";}
 if (sostituzione == "A97P") {valore_pssm = "-2";}
 if (sostituzione == "Q107A") {valore_pssm = "-2";}
 if (sostituzione == "G150Q") {valore_pssm = "-2";}
 if (sostituzione == "I154D") {valore_pssm = "-2";}
 if (sostituzione == "S197M") {valore_pssm = "-2";}
 if (sostituzione == "P214L") {valore_pssm = "-2";}
 if (sostituzione == "N215A") {valore_pssm = "-2";}
 if (sostituzione == "E218M") {valore_pssm = "-2";}
 if (sostituzione == "N224H") {valore_pssm = "-2";}
 if (sostituzione == "D233M") {valore_pssm = "-2";}
 if (sostituzione == "S235G") {valore_pssm = "-2";}
 if (sostituzione == "K240G") {valore_pssm = "-2";}
 if (sostituzione == "S276Q") {valore_pssm = "-2";}
 if (sostituzione == "A291I") {valore_pssm = "-2";}
 if (sostituzione == "N298M") {valore_pssm = "-2";}
 if (sostituzione == "G334F") {valore_pssm = "-2";}
 if (sostituzione == "M353P") {valore_pssm = "-2";}
 if (sostituzione == "G361R") {valore_pssm = "-2";}
 if (sostituzione == "A370P") {valore_pssm = "-2";}
 if (sostituzione == "T420A") {valore_pssm = "-2";}
 if (sostituzione == "Q2N") {valore_pssm = "-2";}
 if (sostituzione == "A15Q") {valore_pssm = "-2";}
 if (sostituzione == "A20G") {valore_pssm = "-2";}
 if (sostituzione == "A37N") {valore_pssm = "-2";}
 if (sostituzione == "D55G") {valore_pssm = "-2";}
 if (sostituzione == "F69L") {valore_pssm = "-2";}
 if (sostituzione == "M72S") {valore_pssm = "-2";}
 if (sostituzione == "E79G") {valore_pssm = "-2";}
 if (sostituzione == "D101T") {valore_pssm = "-2";}
 if (sostituzione == "R105H") {valore_pssm = "-2";}
 if (sostituzione == "A108F") {valore_pssm = "-2";}
 if (sostituzione == "R118F") {valore_pssm = "-2";}
 if (sostituzione == "F145V") {valore_pssm = "-2";}
 if (sostituzione == "C174R") {valore_pssm = "-2";}
 if (sostituzione == "H186A") {valore_pssm = "-2";}
 if (sostituzione == "W209I") {valore_pssm = "-2";}
 if (sostituzione == "D233L") {valore_pssm = "-2";}
 if (sostituzione == "W262Y") {valore_pssm = "-2";}
 if (sostituzione == "V281L") {valore_pssm = "-2";}
 if (sostituzione == "F295T") {valore_pssm = "-2";}
 if (sostituzione == "H302Y") {valore_pssm = "-2";}
 if (sostituzione == "V369Y") {valore_pssm = "-2";}
 if (sostituzione == "T420E") {valore_pssm = "-2";}
 if (sostituzione == "A20E") {valore_pssm = "-2";}
 if (sostituzione == "L21Y") {valore_pssm = "-2";}
 if (sostituzione == "R30T") {valore_pssm = "-2";}
 if (sostituzione == "S65L") {valore_pssm = "-2";}
 if (sostituzione == "E71G") {valore_pssm = "-2";}
 if (sostituzione == "K82F") {valore_pssm = "-2";}
 if (sostituzione == "A84G") {valore_pssm = "-2";}
 if (sostituzione == "E87G") {valore_pssm = "-2";}
 if (sostituzione == "C90S") {valore_pssm = "-2";}
 if (sostituzione == "A97N") {valore_pssm = "-2";}
 if (sostituzione == "A108I") {valore_pssm = "-2";}
 if (sostituzione == "S126C") {valore_pssm = "-2";}
 if (sostituzione == "W204F") {valore_pssm = "-2";}
 if (sostituzione == "P210L") {valore_pssm = "-2";}
 if (sostituzione == "R227K") {valore_pssm = "-2";}
 if (sostituzione == "N228F") {valore_pssm = "-2";}
 if (sostituzione == "N228D") {valore_pssm = "-2";}
 if (sostituzione == "F229I") {valore_pssm = "-2";}
 if (sostituzione == "K240D") {valore_pssm = "-2";}
 if (sostituzione == "F248P") {valore_pssm = "-2";}
 if (sostituzione == "A257R") {valore_pssm = "-2";}
 if (sostituzione == "L286T") {valore_pssm = "-2";}
 if (sostituzione == "P305H") {valore_pssm = "-2";}
 if (sostituzione == "K326G") {valore_pssm = "-2";}
 if (sostituzione == "N355T") {valore_pssm = "-2";}
 if (sostituzione == "E358I") {valore_pssm = "-2";}
 if (sostituzione == "E358F") {valore_pssm = "-2";}
 if (sostituzione == "I359C") {valore_pssm = "-2";}
 if (sostituzione == "T366R") {valore_pssm = "-2";}
 if (sostituzione == "L372T") {valore_pssm = "-2";}
 if (sostituzione == "A377P") {valore_pssm = "-2";}
 if (sostituzione == "V390P") {valore_pssm = "-2";}
 if (sostituzione == "K393D") {valore_pssm = "-2";}
 if (sostituzione == "L425Y") {valore_pssm = "-2";}
 if (sostituzione == "L428Y") {valore_pssm = "-2";}
 if (sostituzione == "M1G") {valore_pssm = "-2";}
 if (sostituzione == "M1P") {valore_pssm = "-2";}
 if (sostituzione == "W24H") {valore_pssm = "-2";}
 if (sostituzione == "E48T") {valore_pssm = "-2";}
 if (sostituzione == "D55F") {valore_pssm = "-2";}
 if (sostituzione == "E71K") {valore_pssm = "-2";}
 if (sostituzione == "K82G") {valore_pssm = "-2";}
 if (sostituzione == "K82N") {valore_pssm = "-2";}
 if (sostituzione == "Y88H") {valore_pssm = "-2";}
 if (sostituzione == "C90E") {valore_pssm = "-2";}
 if (sostituzione == "D101K") {valore_pssm = "-2";}
 if (sostituzione == "E103P") {valore_pssm = "-2";}
 if (sostituzione == "N122Y") {valore_pssm = "-2";}
 if (sostituzione == "N122C") {valore_pssm = "-2";}
 if (sostituzione == "T194V") {valore_pssm = "-2";}
 if (sostituzione == "N228A") {valore_pssm = "-2";}
 if (sostituzione == "S235D") {valore_pssm = "-2";}
 if (sostituzione == "I253R") {valore_pssm = "-2";}
 if (sostituzione == "V256M") {valore_pssm = "-2";}
 if (sostituzione == "S297V") {valore_pssm = "-2";}
 if (sostituzione == "L310R") {valore_pssm = "-2";}
 if (sostituzione == "R332P") {valore_pssm = "-2";}
 if (sostituzione == "E338K") {valore_pssm = "-2";}
 if (sostituzione == "A352G") {valore_pssm = "-2";}
 if (sostituzione == "A381M") {valore_pssm = "-2";}
 if (sostituzione == "T385K") {valore_pssm = "-2";}
 if (sostituzione == "K391H") {valore_pssm = "-2";}
 if (sostituzione == "T400K") {valore_pssm = "-2";}
 if (sostituzione == "T412K") {valore_pssm = "-2";}
 if (sostituzione == "Q416N") {valore_pssm = "-2";}
 if (sostituzione == "M421K") {valore_pssm = "-2";}
 if (sostituzione == "M421H") {valore_pssm = "-2";}
 if (sostituzione == "M423K") {valore_pssm = "-2";}
 if (sostituzione == "M423H") {valore_pssm = "-2";}
 if (sostituzione == "L16R") {valore_pssm = "-2";}
 if (sostituzione == "R17P") {valore_pssm = "-2";}
 if (sostituzione == "F18I") {valore_pssm = "-2";}
 if (sostituzione == "G28S") {valore_pssm = "-2";}
 if (sostituzione == "R30E") {valore_pssm = "-2";}
 if (sostituzione == "L54D") {valore_pssm = "-2";}
 if (sostituzione == "D55A") {valore_pssm = "-2";}
 if (sostituzione == "L68T") {valore_pssm = "-2";}
 if (sostituzione == "R100K") {valore_pssm = "-2";}
 if (sostituzione == "G150T") {valore_pssm = "-2";}
 if (sostituzione == "N179Y") {valore_pssm = "-2";}
 if (sostituzione == "W209C") {valore_pssm = "-2";}
 if (sostituzione == "E218S") {valore_pssm = "-2";}
 if (sostituzione == "K237F") {valore_pssm = "-2";}
 if (sostituzione == "K237Y") {valore_pssm = "-2";}
 if (sostituzione == "G260S") {valore_pssm = "-2";}
 if (sostituzione == "V269P") {valore_pssm = "-2";}
 if (sostituzione == "T282D") {valore_pssm = "-2";}
 if (sostituzione == "A288C") {valore_pssm = "-2";}
 if (sostituzione == "M290S") {valore_pssm = "-2";}
 if (sostituzione == "Q312A") {valore_pssm = "-2";}
 if (sostituzione == "K314N") {valore_pssm = "-2";}
 if (sostituzione == "F337S") {valore_pssm = "-2";}
 if (sostituzione == "W349G") {valore_pssm = "-2";}
 if (sostituzione == "I359K") {valore_pssm = "-2";}
 if (sostituzione == "F383M") {valore_pssm = "-2";}
 if (sostituzione == "K393F") {valore_pssm = "-2";}
 if (sostituzione == "V413A") {valore_pssm = "-2";}
 if (sostituzione == "V413M") {valore_pssm = "-2";}
 if (sostituzione == "D427H") {valore_pssm = "-2";}
 if (sostituzione == "M1S") {valore_pssm = "-2";}
 if (sostituzione == "P6H") {valore_pssm = "-2";}
 if (sostituzione == "E7T") {valore_pssm = "-2";}
 if (sostituzione == "G11E") {valore_pssm = "-2";}
 if (sostituzione == "V22S") {valore_pssm = "-2";}
 if (sostituzione == "V22C") {valore_pssm = "-2";}
 if (sostituzione == "D25V") {valore_pssm = "-2";}
 if (sostituzione == "A37Y") {valore_pssm = "-2";}
 if (sostituzione == "D55I") {valore_pssm = "-2";}
 if (sostituzione == "P60T") {valore_pssm = "-2";}
 if (sostituzione == "E79P") {valore_pssm = "-2";}
 if (sostituzione == "S102I") {valore_pssm = "-2";}
 if (sostituzione == "N192T") {valore_pssm = "-2";}
 if (sostituzione == "P210M") {valore_pssm = "-2";}
 if (sostituzione == "Y216A") {valore_pssm = "-2";}
 if (sostituzione == "S241G") {valore_pssm = "-2";}
 if (sostituzione == "I253K") {valore_pssm = "-2";}
 if (sostituzione == "I289T") {valore_pssm = "-2";}
 if (sostituzione == "M290E") {valore_pssm = "-2";}
 if (sostituzione == "I319L") {valore_pssm = "-2";}
 if (sostituzione == "Q321E") {valore_pssm = "-2";}
 if (sostituzione == "S345V") {valore_pssm = "-2";}
 if (sostituzione == "A350C") {valore_pssm = "-2";}
 if (sostituzione == "E358N") {valore_pssm = "-2";}
 if (sostituzione == "T385I") {valore_pssm = "-2";}
 if (sostituzione == "V390Y") {valore_pssm = "-2";}
 if (sostituzione == "R392M") {valore_pssm = "-2";}
 if (sostituzione == "T400I") {valore_pssm = "-2";}
 if (sostituzione == "T412I") {valore_pssm = "-2";}
 if (sostituzione == "Q2A") {valore_pssm = "-2";}
 if (sostituzione == "R4T") {valore_pssm = "-2";}
 if (sostituzione == "P6E") {valore_pssm = "-2";}
 if (sostituzione == "L14H") {valore_pssm = "-2";}
 if (sostituzione == "A15I") {valore_pssm = "-2";}
 if (sostituzione == "W24E") {valore_pssm = "-2";}
 if (sostituzione == "A31F") {valore_pssm = "-2";}
 if (sostituzione == "D33G") {valore_pssm = "-2";}
 if (sostituzione == "S78I") {valore_pssm = "-2";}
 if (sostituzione == "E87L") {valore_pssm = "-2";}
 if (sostituzione == "I91F") {valore_pssm = "-2";}
 if (sostituzione == "R105Q") {valore_pssm = "-2";}
 if (sostituzione == "Q111P") {valore_pssm = "-2";}
 if (sostituzione == "R112A") {valore_pssm = "-2";}
 if (sostituzione == "R118L") {valore_pssm = "-2";}
 if (sostituzione == "I154Y") {valore_pssm = "-2";}
 if (sostituzione == "G171S") {valore_pssm = "-2";}
 if (sostituzione == "I219N") {valore_pssm = "-2";}
 if (sostituzione == "H225Q") {valore_pssm = "-2";}
 if (sostituzione == "I232A") {valore_pssm = "-2";}
 if (sostituzione == "S235C") {valore_pssm = "-2";}
 if (sostituzione == "L243K") {valore_pssm = "-2";}
 if (sostituzione == "I253A") {valore_pssm = "-2";}
 if (sostituzione == "N263H") {valore_pssm = "-2";}
 if (sostituzione == "L275H") {valore_pssm = "-2";}
 if (sostituzione == "V281S") {valore_pssm = "-2";}
 if (sostituzione == "A288H") {valore_pssm = "-2";}
 if (sostituzione == "R301D") {valore_pssm = "-2";}
 if (sostituzione == "K308T") {valore_pssm = "-2";}
 if (sostituzione == "A318G") {valore_pssm = "-2";}
 if (sostituzione == "N320H") {valore_pssm = "-2";}
 if (sostituzione == "R392A") {valore_pssm = "-2";}
 if (sostituzione == "R402A") {valore_pssm = "-2";}
 if (sostituzione == "D427Q") {valore_pssm = "-2";}
 if (sostituzione == "I26A") {valore_pssm = "-2";}
 if (sostituzione == "R38V") {valore_pssm = "-2";}
 if (sostituzione == "P60R") {valore_pssm = "-2";}
 if (sostituzione == "S65M") {valore_pssm = "-2";}
 if (sostituzione == "R112H") {valore_pssm = "-2";}
 if (sostituzione == "K140G") {valore_pssm = "-2";}
 if (sostituzione == "F149T") {valore_pssm = "-2";}
 if (sostituzione == "G150A") {valore_pssm = "-2";}
 if (sostituzione == "A190T") {valore_pssm = "-2";}
 if (sostituzione == "P214N") {valore_pssm = "-2";}
 if (sostituzione == "N215G") {valore_pssm = "-2";}
 if (sostituzione == "E218A") {valore_pssm = "-2";}
 if (sostituzione == "F229A") {valore_pssm = "-2";}
 if (sostituzione == "I239T") {valore_pssm = "-2";}
 if (sostituzione == "L275Q") {valore_pssm = "-2";}
 if (sostituzione == "L300A") {valore_pssm = "-2";}
 if (sostituzione == "I354G") {valore_pssm = "-2";}
 if (sostituzione == "G360R") {valore_pssm = "-2";}
 if (sostituzione == "G361E") {valore_pssm = "-2";}
 if (sostituzione == "S371L") {valore_pssm = "-2";}
 if (sostituzione == "G375A") {valore_pssm = "-2";}
 if (sostituzione == "V376Y") {valore_pssm = "-2";}
 if (sostituzione == "A381L") {valore_pssm = "-2";}
 if (sostituzione == "G395A") {valore_pssm = "-2";}
 if (sostituzione == "G411A") {valore_pssm = "-2";}
 if (sostituzione == "M421R") {valore_pssm = "-2";}
 if (sostituzione == "M423R") {valore_pssm = "-2";}
 if (sostituzione == "L425A") {valore_pssm = "-2";}
 if (sostituzione == "L425R") {valore_pssm = "-2";}
 if (sostituzione == "L428A") {valore_pssm = "-2";}
 if (sostituzione == "L428R") {valore_pssm = "-2";}
 if (sostituzione == "L3Y") {valore_pssm = "-2";}
 if (sostituzione == "H9K") {valore_pssm = "-2";}
 if (sostituzione == "L10Y") {valore_pssm = "-2";}
 if (sostituzione == "G11Q") {valore_pssm = "-2";}
 if (sostituzione == "L14K") {valore_pssm = "-2";}
 if (sostituzione == "L16E") {valore_pssm = "-2";}
 if (sostituzione == "F18V") {valore_pssm = "-2";}
 if (sostituzione == "W24K") {valore_pssm = "-2";}
 if (sostituzione == "D55P") {valore_pssm = "-2";}
 if (sostituzione == "V77T") {valore_pssm = "-2";}
 if (sostituzione == "E87F") {valore_pssm = "-2";}
 if (sostituzione == "L89K") {valore_pssm = "-2";}
 if (sostituzione == "L89H") {valore_pssm = "-2";}
 if (sostituzione == "C90Q") {valore_pssm = "-2";}
 if (sostituzione == "M96G") {valore_pssm = "-2";}
 if (sostituzione == "D109Q") {valore_pssm = "-2";}
 if (sostituzione == "L120T") {valore_pssm = "-2";}
 if (sostituzione == "V137N") {valore_pssm = "-2";}
 if (sostituzione == "A143D") {valore_pssm = "-2";}
 if (sostituzione == "F145I") {valore_pssm = "-2";}
 if (sostituzione == "D153G") {valore_pssm = "-2";}
 if (sostituzione == "D153F") {valore_pssm = "-2";}
 if (sostituzione == "G171K") {valore_pssm = "-2";}
 if (sostituzione == "E178H") {valore_pssm = "-2";}
 if (sostituzione == "N192L") {valore_pssm = "-2";}
 if (sostituzione == "R193M") {valore_pssm = "-2";}
 if (sostituzione == "S201E") {valore_pssm = "-2";}
 if (sostituzione == "T217H") {valore_pssm = "-2";}
 if (sostituzione == "H225K") {valore_pssm = "-2";}
 if (sostituzione == "F229K") {valore_pssm = "-2";}
 if (sostituzione == "S238E") {valore_pssm = "-2";}
 if (sostituzione == "I253Q") {valore_pssm = "-2";}
 if (sostituzione == "A288Q") {valore_pssm = "-2";}
 if (sostituzione == "Y329I") {valore_pssm = "-2";}
 if (sostituzione == "R332G") {valore_pssm = "-2";}
 if (sostituzione == "A348C") {valore_pssm = "-2";}
 if (sostituzione == "W349M") {valore_pssm = "-2";}
 if (sostituzione == "I354P") {valore_pssm = "-2";}
 if (sostituzione == "I384T") {valore_pssm = "-2";}
 if (sostituzione == "T385A") {valore_pssm = "-2";}
 if (sostituzione == "L387Y") {valore_pssm = "-2";}
 if (sostituzione == "L388Y") {valore_pssm = "-2";}
 if (sostituzione == "L394Y") {valore_pssm = "-2";}
 if (sostituzione == "E398R") {valore_pssm = "-2";}
 if (sostituzione == "T400A") {valore_pssm = "-2";}
 if (sostituzione == "L403Y") {valore_pssm = "-2";}
 if (sostituzione == "H406K") {valore_pssm = "-2";}
 if (sostituzione == "T412A") {valore_pssm = "-2";}
 if (sostituzione == "L414Y") {valore_pssm = "-2";}
 if (sostituzione == "L415S") {valore_pssm = "-2";}
 if (sostituzione == "L417Y") {valore_pssm = "-2";}
 if (sostituzione == "E418R") {valore_pssm = "-2";}
 if (sostituzione == "L425H") {valore_pssm = "-2";}
 if (sostituzione == "L428H") {valore_pssm = "-2";}
 if (sostituzione == "L14R") {valore_pssm = "-2";}
 if (sostituzione == "A15R") {valore_pssm = "-2";}
 if (sostituzione == "L16Q") {valore_pssm = "-2";}
 if (sostituzione == "R49S") {valore_pssm = "-2";}
 if (sostituzione == "E79F") {valore_pssm = "-2";}
 if (sostituzione == "A97I") {valore_pssm = "-2";}
 if (sostituzione == "A108Y") {valore_pssm = "-2";}
 if (sostituzione == "Y123K") {valore_pssm = "-2";}
 if (sostituzione == "D161C") {valore_pssm = "-2";}
 if (sostituzione == "G183N") {valore_pssm = "-2";}
 if (sostituzione == "N192E") {valore_pssm = "-2";}
 if (sostituzione == "V199K") {valore_pssm = "-2";}
 if (sostituzione == "N215C") {valore_pssm = "-2";}
 if (sostituzione == "W245L") {valore_pssm = "-2";}
 if (sostituzione == "G261F") {valore_pssm = "-2";}
 if (sostituzione == "I270M") {valore_pssm = "-2";}
 if (sostituzione == "A285I") {valore_pssm = "-2";}
 if (sostituzione == "A288G") {valore_pssm = "-2";}
 if (sostituzione == "A292K") {valore_pssm = "-2";}
 if (sostituzione == "N298Y") {valore_pssm = "-2";}
 if (sostituzione == "R301I") {valore_pssm = "-2";}
 if (sostituzione == "H302F") {valore_pssm = "-2";}
 if (sostituzione == "K314G") {valore_pssm = "-2";}
 if (sostituzione == "K374N") {valore_pssm = "-2";}
 if (sostituzione == "T385E") {valore_pssm = "-2";}
 if (sostituzione == "K393V") {valore_pssm = "-2";}
 if (sostituzione == "T400E") {valore_pssm = "-2";}
 if (sostituzione == "T412E") {valore_pssm = "-2";}
 if (sostituzione == "S424F") {valore_pssm = "-2";}
 if (sostituzione == "N5F") {valore_pssm = "-2";}
 if (sostituzione == "I26C") {valore_pssm = "-2";}
 if (sostituzione == "N53R") {valore_pssm = "-2";}
 if (sostituzione == "L54N") {valore_pssm = "-2";}
 if (sostituzione == "P60Q") {valore_pssm = "-2";}
 if (sostituzione == "E71H") {valore_pssm = "-2";}
 if (sostituzione == "M76F") {valore_pssm = "-2";}
 if (sostituzione == "K82P") {valore_pssm = "-2";}
 if (sostituzione == "Y86H") {valore_pssm = "-2";}
 if (sostituzione == "C90R") {valore_pssm = "-2";}
 if (sostituzione == "R105E") {valore_pssm = "-2";}
 if (sostituzione == "L131T") {valore_pssm = "-2";}
 if (sostituzione == "Y134H") {valore_pssm = "-2";}
 if (sostituzione == "S148R") {valore_pssm = "-2";}
 if (sostituzione == "Y184H") {valore_pssm = "-2";}
 if (sostituzione == "A190I") {valore_pssm = "-2";}
 if (sostituzione == "N192R") {valore_pssm = "-2";}
 if (sostituzione == "S201K") {valore_pssm = "-2";}
 if (sostituzione == "I219D") {valore_pssm = "-2";}
 if (sostituzione == "S238K") {valore_pssm = "-2";}
 if (sostituzione == "V256S") {valore_pssm = "-2";}
 if (sostituzione == "V256K") {valore_pssm = "-2";}
 if (sostituzione == "S276R") {valore_pssm = "-2";}
 if (sostituzione == "G346L") {valore_pssm = "-2";}
 if (sostituzione == "A348P") {valore_pssm = "-2";}
 if (sostituzione == "T366N") {valore_pssm = "-2";}
 if (sostituzione == "T420Q") {valore_pssm = "-2";}
 if (sostituzione == "S424I") {valore_pssm = "-2";}
 if (sostituzione == "L45P") {valore_pssm = "-2";}
 if (sostituzione == "E58G") {valore_pssm = "-2";}
 if (sostituzione == "E66Q") {valore_pssm = "-2";}
 if (sostituzione == "M72K") {valore_pssm = "-2";}
 if (sostituzione == "K82C") {valore_pssm = "-2";}
 if (sostituzione == "A84P") {valore_pssm = "-2";}
 if (sostituzione == "Q111G") {valore_pssm = "-2";}
 if (sostituzione == "Q111I") {valore_pssm = "-2";}
 if (sostituzione == "Y123Q") {valore_pssm = "-2";}
 if (sostituzione == "L131H") {valore_pssm = "-2";}
 if (sostituzione == "Y151T") {valore_pssm = "-2";}
 if (sostituzione == "S188P") {valore_pssm = "-2";}
 if (sostituzione == "N228C") {valore_pssm = "-2";}
 if (sostituzione == "I232L") {valore_pssm = "-2";}
 if (sostituzione == "K237I") {valore_pssm = "-2";}
 if (sostituzione == "G261A") {valore_pssm = "-2";}
 if (sostituzione == "S297C") {valore_pssm = "-2";}
 if (sostituzione == "L310Q") {valore_pssm = "-2";}
 if (sostituzione == "L310E") {valore_pssm = "-2";}
 if (sostituzione == "V316F") {valore_pssm = "-2";}
 if (sostituzione == "Q327N") {valore_pssm = "-2";}
 if (sostituzione == "S345R") {valore_pssm = "-2";}
 if (sostituzione == "A350K") {valore_pssm = "-2";}
 if (sostituzione == "N355K") {valore_pssm = "-2";}
 if (sostituzione == "I359Q") {valore_pssm = "-2";}
 if (sostituzione == "T366A") {valore_pssm = "-2";}
 if (sostituzione == "V413F") {valore_pssm = "-2";}
 if (sostituzione == "T420R") {valore_pssm = "-2";}
 if (sostituzione == "M1Y") {valore_pssm = "-2";}
 if (sostituzione == "Q2D") {valore_pssm = "-2";}
 if (sostituzione == "G11R") {valore_pssm = "-2";}
 if (sostituzione == "L16Y") {valore_pssm = "-2";}
 if (sostituzione == "H46R") {valore_pssm = "-2";}
 if (sostituzione == "E58N") {valore_pssm = "-2";}
 if (sostituzione == "S78M") {valore_pssm = "-2";}
 if (sostituzione == "E79M") {valore_pssm = "-2";}
 if (sostituzione == "M96P") {valore_pssm = "-2";}
 if (sostituzione == "D109R") {valore_pssm = "-2";}
 if (sostituzione == "P110D") {valore_pssm = "-2";}
 if (sostituzione == "N139A") {valore_pssm = "-2";}
 if (sostituzione == "F145L") {valore_pssm = "-2";}
 if (sostituzione == "A156T") {valore_pssm = "-2";}
 if (sostituzione == "D161I") {valore_pssm = "-2";}
 if (sostituzione == "D161F") {valore_pssm = "-2";}
 if (sostituzione == "N192Q") {valore_pssm = "-2";}
 if (sostituzione == "T217F") {valore_pssm = "-2";}
 if (sostituzione == "H225I") {valore_pssm = "-2";}
 if (sostituzione == "F229E") {valore_pssm = "-2";}
 if (sostituzione == "S241V") {valore_pssm = "-2";}
 if (sostituzione == "I242L") {valore_pssm = "-2";}
 if (sostituzione == "L243R") {valore_pssm = "-2";}
 if (sostituzione == "F248D") {valore_pssm = "-2";}
 if (sostituzione == "A257C") {valore_pssm = "-2";}
 if (sostituzione == "I270T") {valore_pssm = "-2";}
 if (sostituzione == "A292E") {valore_pssm = "-2";}
 if (sostituzione == "P305T") {valore_pssm = "-2";}
 if (sostituzione == "K308Y") {valore_pssm = "-2";}
 if (sostituzione == "G334P") {valore_pssm = "-2";}
 if (sostituzione == "S345N") {valore_pssm = "-2";}
 if (sostituzione == "A348I") {valore_pssm = "-2";}
 if (sostituzione == "W349P") {valore_pssm = "-2";}
 if (sostituzione == "A352P") {valore_pssm = "-2";}
 if (sostituzione == "E358G") {valore_pssm = "-2";}
 if (sostituzione == "L372C") {valore_pssm = "-2";}
 if (sostituzione == "R402N") {valore_pssm = "-2";}
 if (sostituzione == "L3A") {valore_pssm = "-2";}
 if (sostituzione == "L3R") {valore_pssm = "-2";}
 if (sostituzione == "N5I") {valore_pssm = "-2";}
 if (sostituzione == "L10A") {valore_pssm = "-2";}
 if (sostituzione == "L10R") {valore_pssm = "-2";}
 if (sostituzione == "R38E") {valore_pssm = "-2";}
 if (sostituzione == "W44Y") {valore_pssm = "-2";}
 if (sostituzione == "R49Q") {valore_pssm = "-2";}
 if (sostituzione == "M70K") {valore_pssm = "-2";}
 if (sostituzione == "M72Q") {valore_pssm = "-2";}
 if (sostituzione == "S78F") {valore_pssm = "-2";}
 if (sostituzione == "E87Y") {valore_pssm = "-2";}
 if (sostituzione == "L89R") {valore_pssm = "-2";}
 if (sostituzione == "S148N") {valore_pssm = "-2";}
 if (sostituzione == "Y152G") {valore_pssm = "-2";}
 if (sostituzione == "Q157S") {valore_pssm = "-2";}
 if (sostituzione == "R193F") {valore_pssm = "-2";}
 if (sostituzione == "S201H") {valore_pssm = "-2";}
 if (sostituzione == "W204A") {valore_pssm = "-2";}
 if (sostituzione == "P214D") {valore_pssm = "-2";}
 if (sostituzione == "N215L") {valore_pssm = "-2";}
 if (sostituzione == "D233C") {valore_pssm = "-2";}
 if (sostituzione == "S238H") {valore_pssm = "-2";}
 if (sostituzione == "L243S") {valore_pssm = "-2";}
 if (sostituzione == "F248N") {valore_pssm = "-2";}
 if (sostituzione == "Q250T") {valore_pssm = "-2";}
 if (sostituzione == "I253E") {valore_pssm = "-2";}
 if (sostituzione == "L275C") {valore_pssm = "-2";}
 if (sostituzione == "L275R") {valore_pssm = "-2";}
 if (sostituzione == "Q279S") {valore_pssm = "-2";}
 if (sostituzione == "Q279T") {valore_pssm = "-2";}
 if (sostituzione == "A285P") {valore_pssm = "-2";}
 if (sostituzione == "A288I") {valore_pssm = "-2";}
 if (sostituzione == "N355E") {valore_pssm = "-2";}
 if (sostituzione == "I359H") {valore_pssm = "-2";}
 if (sostituzione == "G361C") {valore_pssm = "-2";}
 if (sostituzione == "T366I") {valore_pssm = "-2";}
 if (sostituzione == "I367N") {valore_pssm = "-2";}
 if (sostituzione == "A381Y") {valore_pssm = "-2";}
 if (sostituzione == "L387A") {valore_pssm = "-2";}
 if (sostituzione == "L387R") {valore_pssm = "-2";}
 if (sostituzione == "L388A") {valore_pssm = "-2";}
 if (sostituzione == "L388R") {valore_pssm = "-2";}
 if (sostituzione == "V390N") {valore_pssm = "-2";}
 if (sostituzione == "L394A") {valore_pssm = "-2";}
 if (sostituzione == "L394R") {valore_pssm = "-2";}
 if (sostituzione == "L403A") {valore_pssm = "-2";}
 if (sostituzione == "L403R") {valore_pssm = "-2";}
 if (sostituzione == "L414A") {valore_pssm = "-2";}
 if (sostituzione == "L414R") {valore_pssm = "-2";}
 if (sostituzione == "L417A") {valore_pssm = "-2";}
 if (sostituzione == "L417R") {valore_pssm = "-2";}
 if (sostituzione == "N419G") {valore_pssm = "-2";}
 if (sostituzione == "Q2V") {valore_pssm = "-2";}
 if (sostituzione == "A20Q") {valore_pssm = "-2";}
 if (sostituzione == "W24Q") {valore_pssm = "-2";}
 if (sostituzione == "A37D") {valore_pssm = "-2";}
 if (sostituzione == "I64F") {valore_pssm = "-2";}
 if (sostituzione == "E87C") {valore_pssm = "-2";}
 if (sostituzione == "Q157T") {valore_pssm = "-2";}
 if (sostituzione == "L166D") {valore_pssm = "-2";}
 if (sostituzione == "G171W") {valore_pssm = "-2";}
 if (sostituzione == "C174G") {valore_pssm = "-2";}
 if (sostituzione == "K185H") {valore_pssm = "-2";}
 if (sostituzione == "R193Y") {valore_pssm = "-2";}
 if (sostituzione == "W204Y") {valore_pssm = "-2";}
 if (sostituzione == "F211P") {valore_pssm = "-2";}
 if (sostituzione == "F248G") {valore_pssm = "-2";}
 if (sostituzione == "F273Y") {valore_pssm = "-2";}
 if (sostituzione == "L275K") {valore_pssm = "-2";}
 if (sostituzione == "S276N") {valore_pssm = "-2";}
 if (sostituzione == "V281M") {valore_pssm = "-2";}
 if (sostituzione == "T282F") {valore_pssm = "-2";}
 if (sostituzione == "H302L") {valore_pssm = "-2";}
 if (sostituzione == "L311T") {valore_pssm = "-2";}
 if (sostituzione == "A318C") {valore_pssm = "-2";}
 if (sostituzione == "R332Y") {valore_pssm = "-2";}
 if (sostituzione == "M353G") {valore_pssm = "-2";}
 if (sostituzione == "I359R") {valore_pssm = "-2";}
 if (sostituzione == "K374A") {valore_pssm = "-2";}
 if (sostituzione == "R392F") {valore_pssm = "-2";}
 if (sostituzione == "K393P") {valore_pssm = "-2";}
 if (sostituzione == "T410F") {valore_pssm = "-2";}
 if (sostituzione == "L425K") {valore_pssm = "-2";}
 if (sostituzione == "L428K") {valore_pssm = "-2";}
 if (sostituzione == "M1D") {valore_pssm = "-2";}
 if (sostituzione == "L3H") {valore_pssm = "-2";}
 if (sostituzione == "L10H") {valore_pssm = "-2";}
 if (sostituzione == "R38I") {valore_pssm = "-2";}
 if (sostituzione == "F69Y") {valore_pssm = "-2";}
 if (sostituzione == "E71D") {valore_pssm = "-2";}
 if (sostituzione == "S78Y") {valore_pssm = "-2";}
 if (sostituzione == "K82Y") {valore_pssm = "-2";}
 if (sostituzione == "D101Q") {valore_pssm = "-2";}
 if (sostituzione == "E103I") {valore_pssm = "-2";}
 if (sostituzione == "D109T") {valore_pssm = "-2";}
 if (sostituzione == "N122M") {valore_pssm = "-2";}
 if (sostituzione == "K127T") {valore_pssm = "-2";}
 if (sostituzione == "N139V") {valore_pssm = "-2";}
 if (sostituzione == "A143Y") {valore_pssm = "-2";}
 if (sostituzione == "D153L") {valore_pssm = "-2";}
 if (sostituzione == "F169Q") {valore_pssm = "-2";}
 if (sostituzione == "G171T") {valore_pssm = "-2";}
 if (sostituzione == "V199E") {valore_pssm = "-2";}
 if (sostituzione == "C223S") {valore_pssm = "-2";}
 if (sostituzione == "W226F") {valore_pssm = "-2";}
 if (sostituzione == "V256C") {valore_pssm = "-2";}
 if (sostituzione == "I270F") {valore_pssm = "-2";}
 if (sostituzione == "A288R") {valore_pssm = "-2";}
 if (sostituzione == "A292G") {valore_pssm = "-2";}
 if (sostituzione == "F295C") {valore_pssm = "-2";}
 if (sostituzione == "R301S") {valore_pssm = "-2";}
 if (sostituzione == "Q327K") {valore_pssm = "-2";}
 if (sostituzione == "L331P") {valore_pssm = "-2";}
 if (sostituzione == "G373A") {valore_pssm = "-2";}
 if (sostituzione == "L387H") {valore_pssm = "-2";}
 if (sostituzione == "L388H") {valore_pssm = "-2";}
 if (sostituzione == "L394H") {valore_pssm = "-2";}
 if (sostituzione == "L403H") {valore_pssm = "-2";}
 if (sostituzione == "R404T") {valore_pssm = "-2";}
 if (sostituzione == "L414H") {valore_pssm = "-2";}
 if (sostituzione == "Q416A") {valore_pssm = "-2";}
 if (sostituzione == "L417H") {valore_pssm = "-2";}
 if (sostituzione == "T420H") {valore_pssm = "-2";}
 if (sostituzione == "K426T") {valore_pssm = "-2";}
 if (sostituzione == "R17G") {valore_pssm = "-2";}
 if (sostituzione == "L21G") {valore_pssm = "-2";}
 if (sostituzione == "H46V") {valore_pssm = "-2";}
 if (sostituzione == "E58Y") {valore_pssm = "-2";}
 if (sostituzione == "E58C") {valore_pssm = "-2";}
 if (sostituzione == "G150R") {valore_pssm = "-2";}
 if (sostituzione == "F169T") {valore_pssm = "-2";}
 if (sostituzione == "G171A") {valore_pssm = "-2";}
 if (sostituzione == "V199R") {valore_pssm = "-2";}
 if (sostituzione == "Y207V") {valore_pssm = "-2";}
 if (sostituzione == "W209P") {valore_pssm = "-2";}
 if (sostituzione == "F229R") {valore_pssm = "-2";}
 if (sostituzione == "K237C") {valore_pssm = "-2";}
 if (sostituzione == "L243E") {valore_pssm = "-2";}
 if (sostituzione == "V256Q") {valore_pssm = "-2";}
 if (sostituzione == "V256R") {valore_pssm = "-2";}
 if (sostituzione == "V256E") {valore_pssm = "-2";}
 if (sostituzione == "V269N") {valore_pssm = "-2";}
 if (sostituzione == "A285N") {valore_pssm = "-2";}
 if (sostituzione == "L300Y") {valore_pssm = "-2";}
 if (sostituzione == "A350E") {valore_pssm = "-2";}
 if (sostituzione == "A352Y") {valore_pssm = "-2";}
 if (sostituzione == "E358Y") {valore_pssm = "-2";}
 if (sostituzione == "T410M") {valore_pssm = "-2";}
 if (sostituzione == "Q416V") {valore_pssm = "-2";}
 if (sostituzione == "T420C") {valore_pssm = "-2";}
 if (sostituzione == "T420N") {valore_pssm = "-2";}
 if (sostituzione == "K426S") {valore_pssm = "-2";}
 if (sostituzione == "A20R") {valore_pssm = "-2";}
 if (sostituzione == "L21P") {valore_pssm = "-2";}
 if (sostituzione == "V22H") {valore_pssm = "-2";}
 if (sostituzione == "D25P") {valore_pssm = "-2";}
 if (sostituzione == "P60A") {valore_pssm = "-2";}
 if (sostituzione == "Q107S") {valore_pssm = "-2";}
 if (sostituzione == "Q111C") {valore_pssm = "-2";}
 if (sostituzione == "K127H") {valore_pssm = "-2";}
 if (sostituzione == "V137D") {valore_pssm = "-2";}
 if (sostituzione == "I154C") {valore_pssm = "-2";}
 if (sostituzione == "F169K") {valore_pssm = "-2";}
 if (sostituzione == "F169R") {valore_pssm = "-2";}
 if (sostituzione == "S188Y") {valore_pssm = "-2";}
 if (sostituzione == "S201Q") {valore_pssm = "-2";}
 if (sostituzione == "T217K") {valore_pssm = "-2";}
 if (sostituzione == "S238Q") {valore_pssm = "-2";}
 if (sostituzione == "L243Q") {valore_pssm = "-2";}
 if (sostituzione == "T282M") {valore_pssm = "-2";}
 if (sostituzione == "M296T") {valore_pssm = "-2";}
 if (sostituzione == "S297D") {valore_pssm = "-2";}
 if (sostituzione == "H302M") {valore_pssm = "-2";}
 if (sostituzione == "S304V") {valore_pssm = "-2";}
 if (sostituzione == "W340V") {valore_pssm = "-2";}
 if (sostituzione == "T366Y") {valore_pssm = "-2";}
 if (sostituzione == "V369K") {valore_pssm = "-2";}
 if (sostituzione == "V369R") {valore_pssm = "-2";}
 if (sostituzione == "R402I") {valore_pssm = "-2";}
 if (sostituzione == "A15P") {valore_pssm = "-2";}
 if (sostituzione == "W24A") {valore_pssm = "-2";}
 if (sostituzione == "W24R") {valore_pssm = "-2";}
 if (sostituzione == "A31L") {valore_pssm = "-2";}
 if (sostituzione == "H46G") {valore_pssm = "-2";}
 if (sostituzione == "P60I") {valore_pssm = "-2";}
 if (sostituzione == "M70F") {valore_pssm = "-2";}
 if (sostituzione == "A73T") {valore_pssm = "-2";}
 if (sostituzione == "A121T") {valore_pssm = "-2";}
 if (sostituzione == "S126P") {valore_pssm = "-2";}
 if (sostituzione == "K130Q") {valore_pssm = "-2";}
 if (sostituzione == "D153M") {valore_pssm = "-2";}
 if (sostituzione == "A160T") {valore_pssm = "-2";}
 if (sostituzione == "G171E") {valore_pssm = "-2";}
 if (sostituzione == "C174D") {valore_pssm = "-2";}
 if (sostituzione == "R193L") {valore_pssm = "-2";}
 if (sostituzione == "W209L") {valore_pssm = "-2";}
 if (sostituzione == "Q212Y") {valore_pssm = "-2";}
 if (sostituzione == "W245A") {valore_pssm = "-2";}
 if (sostituzione == "W245C") {valore_pssm = "-2";}
 if (sostituzione == "L275A") {valore_pssm = "-2";}
 if (sostituzione == "A291F") {valore_pssm = "-2";}
 if (sostituzione == "L310S") {valore_pssm = "-2";}
 if (sostituzione == "L311H") {valore_pssm = "-2";}
 if (sostituzione == "Q312V") {valore_pssm = "-2";}
 if (sostituzione == "S345G") {valore_pssm = "-2";}
 if (sostituzione == "I354N") {valore_pssm = "-2";}
 if (sostituzione == "N355D") {valore_pssm = "-2";}
 if (sostituzione == "I359E") {valore_pssm = "-2";}
 if (sostituzione == "R363N") {valore_pssm = "-2";}
 if (sostituzione == "I367Y") {valore_pssm = "-2";}
 if (sostituzione == "T385Q") {valore_pssm = "-2";}
 if (sostituzione == "T400Q") {valore_pssm = "-2";}
 if (sostituzione == "T412Q") {valore_pssm = "-2";}
 if (sostituzione == "M421A") {valore_pssm = "-2";}
 if (sostituzione == "M423A") {valore_pssm = "-2";}
 if (sostituzione == "L14Q") {valore_pssm = "-2";}
 if (sostituzione == "R17D") {valore_pssm = "-2";}
 if (sostituzione == "D25G") {valore_pssm = "-2";}
 if (sostituzione == "P27D") {valore_pssm = "-2";}
 if (sostituzione == "A31N") {valore_pssm = "-2";}
 if (sostituzione == "R38H") {valore_pssm = "-2";}
 if (sostituzione == "T39S") {valore_pssm = "-2";}
 if (sostituzione == "E58L") {valore_pssm = "-2";}
 if (sostituzione == "M72H") {valore_pssm = "-2";}
 if (sostituzione == "D109S") {valore_pssm = "-2";}
 if (sostituzione == "H115N") {valore_pssm = "-2";}
 if (sostituzione == "N122L") {valore_pssm = "-2";}
 if (sostituzione == "F149C") {valore_pssm = "-2";}
 if (sostituzione == "G171Q") {valore_pssm = "-2";}
 if (sostituzione == "G183V") {valore_pssm = "-2";}
 if (sostituzione == "H186V") {valore_pssm = "-2";}
 if (sostituzione == "S188I") {valore_pssm = "-2";}
 if (sostituzione == "H225R") {valore_pssm = "-2";}
 if (sostituzione == "F229Q") {valore_pssm = "-2";}
 if (sostituzione == "L286C") {valore_pssm = "-2";}
 if (sostituzione == "A292C") {valore_pssm = "-2";}
 if (sostituzione == "N355Q") {valore_pssm = "-2";}
 if (sostituzione == "A370F") {valore_pssm = "-2";}
 if (sostituzione == "A370N") {valore_pssm = "-2";}
 if (sostituzione == "K374V") {valore_pssm = "-2";}
 if (sostituzione == "T385R") {valore_pssm = "-2";}
 if (sostituzione == "T400R") {valore_pssm = "-2";}
 if (sostituzione == "T412R") {valore_pssm = "-2";}
 if (sostituzione == "M421C") {valore_pssm = "-2";}
 if (sostituzione == "M423C") {valore_pssm = "-2";}
 if (sostituzione == "L425Q") {valore_pssm = "-2";}
 if (sostituzione == "D427T") {valore_pssm = "-2";}
 if (sostituzione == "L428Q") {valore_pssm = "-2";}
 if (sostituzione == "L3K") {valore_pssm = "-2";}
 if (sostituzione == "L10K") {valore_pssm = "-2";}
 if (sostituzione == "A20L") {valore_pssm = "-2";}
 if (sostituzione == "L32P") {valore_pssm = "-2";}
 if (sostituzione == "M51C") {valore_pssm = "-2";}
 if (sostituzione == "P60F") {valore_pssm = "-2";}
 if (sostituzione == "M72F") {valore_pssm = "-2";}
 if (sostituzione == "M96Y") {valore_pssm = "-2";}
 if (sostituzione == "Q107C") {valore_pssm = "-2";}
 if (sostituzione == "Q111M") {valore_pssm = "-2";}
 if (sostituzione == "K130E") {valore_pssm = "-2";}
 if (sostituzione == "S148C") {valore_pssm = "-2";}
 if (sostituzione == "Y151V") {valore_pssm = "-2";}
 if (sostituzione == "Y151N") {valore_pssm = "-2";}
 if (sostituzione == "I154P") {valore_pssm = "-2";}
 if (sostituzione == "S188F") {valore_pssm = "-2";}
 if (sostituzione == "S188D") {valore_pssm = "-2";}
 if (sostituzione == "N192D") {valore_pssm = "-2";}
 if (sostituzione == "Y216V") {valore_pssm = "-2";}
 if (sostituzione == "D244A") {valore_pssm = "-2";}
 if (sostituzione == "G258K") {valore_pssm = "-2";}
 if (sostituzione == "G261Q") {valore_pssm = "-2";}
 if (sostituzione == "G261K") {valore_pssm = "-2";}
 if (sostituzione == "M267I") {valore_pssm = "-2";}
 if (sostituzione == "L275Y") {valore_pssm = "-2";}
 if (sostituzione == "Q283N") {valore_pssm = "-2";}
 if (sostituzione == "A291M") {valore_pssm = "-2";}
 if (sostituzione == "A292H") {valore_pssm = "-2";}
 if (sostituzione == "V351M") {valore_pssm = "-2";}
 if (sostituzione == "I359Y") {valore_pssm = "-2";}
 if (sostituzione == "A377F") {valore_pssm = "-2";}
 if (sostituzione == "N379G") {valore_pssm = "-2";}
 if (sostituzione == "L387K") {valore_pssm = "-2";}
 if (sostituzione == "L388K") {valore_pssm = "-2";}
 if (sostituzione == "L394K") {valore_pssm = "-2";}
 if (sostituzione == "L403K") {valore_pssm = "-2";}
 if (sostituzione == "N408G") {valore_pssm = "-2";}
 if (sostituzione == "V413C") {valore_pssm = "-2";}
 if (sostituzione == "L414K") {valore_pssm = "-2";}
 if (sostituzione == "L417K") {valore_pssm = "-2";}
 if (sostituzione == "N5Y") {valore_pssm = "-2";}
 if (sostituzione == "V22K") {valore_pssm = "-2";}
 if (sostituzione == "L68C") {valore_pssm = "-2";}
 if (sostituzione == "M72E") {valore_pssm = "-2";}
 if (sostituzione == "E74A") {valore_pssm = "-2";}
 if (sostituzione == "V77L") {valore_pssm = "-2";}
 if (sostituzione == "K82D") {valore_pssm = "-2";}
 if (sostituzione == "L89Q") {valore_pssm = "-2";}
 if (sostituzione == "L106T") {valore_pssm = "-2";}
 if (sostituzione == "F149R") {valore_pssm = "-2";}
 if (sostituzione == "E178D") {valore_pssm = "-2";}
 if (sostituzione == "F211G") {valore_pssm = "-2";}
 if (sostituzione == "K213A") {valore_pssm = "-2";}
 if (sostituzione == "K213V") {valore_pssm = "-2";}
 if (sostituzione == "T217A") {valore_pssm = "-2";}
 if (sostituzione == "Y222P") {valore_pssm = "-2";}
 if (sostituzione == "M267L") {valore_pssm = "-2";}
 if (sostituzione == "N272K") {valore_pssm = "-2";}
 if (sostituzione == "N272E") {valore_pssm = "-2";}
 if (sostituzione == "S276C") {valore_pssm = "-2";}
 if (sostituzione == "V281F") {valore_pssm = "-2";}
 if (sostituzione == "S304G") {valore_pssm = "-2";}
 if (sostituzione == "E338H") {valore_pssm = "-2";}
 if (sostituzione == "S345C") {valore_pssm = "-2";}
 if (sostituzione == "A350G") {valore_pssm = "-2";}
 if (sostituzione == "A350M") {valore_pssm = "-2";}
 if (sostituzione == "A350F") {valore_pssm = "-2";}
 if (sostituzione == "I367P") {valore_pssm = "-2";}
 if (sostituzione == "V376K") {valore_pssm = "-2";}
 if (sostituzione == "V376R") {valore_pssm = "-2";}
 if (sostituzione == "R404S") {valore_pssm = "-2";}
 if (sostituzione == "I407Y") {valore_pssm = "-2";}
 if (sostituzione == "S424Y") {valore_pssm = "-2";}
 if (sostituzione == "D427S") {valore_pssm = "-2";}
 if (sostituzione == "L19T") {valore_pssm = "-2";}
 if (sostituzione == "A31M") {valore_pssm = "-2";}
 if (sostituzione == "P60S") {valore_pssm = "-2";}
 if (sostituzione == "L89E") {valore_pssm = "-2";}
 if (sostituzione == "E103M") {valore_pssm = "-2";}
 if (sostituzione == "E103Y") {valore_pssm = "-2";}
 if (sostituzione == "H115S") {valore_pssm = "-2";}
 if (sostituzione == "D153Y") {valore_pssm = "-2";}
 if (sostituzione == "V199Q") {valore_pssm = "-2";}
 if (sostituzione == "T217E") {valore_pssm = "-2";}
 if (sostituzione == "E218P") {valore_pssm = "-2";}
 if (sostituzione == "G261E") {valore_pssm = "-2";}
 if (sostituzione == "M267V") {valore_pssm = "-2";}
 if (sostituzione == "M284H") {valore_pssm = "-2";}
 if (sostituzione == "Q306V") {valore_pssm = "-2";}
 if (sostituzione == "Q312N") {valore_pssm = "-2";}
 if (sostituzione == "W349D") {valore_pssm = "-2";}
 if (sostituzione == "A350H") {valore_pssm = "-2";}
 if (sostituzione == "E358L") {valore_pssm = "-2";}
 if (sostituzione == "E358C") {valore_pssm = "-2";}
 if (sostituzione == "V369H") {valore_pssm = "-2";}
 if (sostituzione == "L372Y") {valore_pssm = "-2";}
 if (sostituzione == "A377L") {valore_pssm = "-2";}
 if (sostituzione == "G11N") {valore_pssm = "-2";}
 if (sostituzione == "L14E") {valore_pssm = "-2";}
 if (sostituzione == "G28V") {valore_pssm = "-2";}
 if (sostituzione == "D33L") {valore_pssm = "-2";}
 if (sostituzione == "H46D") {valore_pssm = "-2";}
 if (sostituzione == "W47F") {valore_pssm = "-2";}
 if (sostituzione == "R49E") {valore_pssm = "-2";}
 if (sostituzione == "D55C") {valore_pssm = "-2";}
 if (sostituzione == "M72R") {valore_pssm = "-2";}
 if (sostituzione == "W95F") {valore_pssm = "-2";}
 if (sostituzione == "A97F") {valore_pssm = "-2";}
 if (sostituzione == "Q99V") {valore_pssm = "-2";}
 if (sostituzione == "S102F") {valore_pssm = "-2";}
 if (sostituzione == "L131C") {valore_pssm = "-2";}
 if (sostituzione == "N139G") {valore_pssm = "-2";}
 if (sostituzione == "G144R") {valore_pssm = "-2";}
 if (sostituzione == "W162F") {valore_pssm = "-2";}
 if (sostituzione == "G171D") {valore_pssm = "-2";}
 if (sostituzione == "G183D") {valore_pssm = "-2";}
 if (sostituzione == "H186E") {valore_pssm = "-2";}
 if (sostituzione == "A190C") {valore_pssm = "-2";}
 if (sostituzione == "F229L") {valore_pssm = "-2";}
 if (sostituzione == "S241C") {valore_pssm = "-2";}
 if (sostituzione == "W287F") {valore_pssm = "-2";}
 if (sostituzione == "R332M") {valore_pssm = "-2";}
 if (sostituzione == "W340H") {valore_pssm = "-2";}
 if (sostituzione == "R356Q") {valore_pssm = "-2";}
 if (sostituzione == "V369Q") {valore_pssm = "-2";}
 if (sostituzione == "K391S") {valore_pssm = "-2";}
 if (sostituzione == "K393G") {valore_pssm = "-2";}
 if (sostituzione == "Q416D") {valore_pssm = "-2";}
 if (sostituzione == "M1N") {valore_pssm = "-2";}
 if (sostituzione == "Q2P") {valore_pssm = "-2";}
 if (sostituzione == "D25I") {valore_pssm = "-2";}
 if (sostituzione == "I64M") {valore_pssm = "-2";}
 if (sostituzione == "E74P") {valore_pssm = "-2";}
 if (sostituzione == "E103C") {valore_pssm = "-2";}
 if (sostituzione == "V164L") {valore_pssm = "-2";}
 if (sostituzione == "K185S") {valore_pssm = "-2";}
 if (sostituzione == "A190K") {valore_pssm = "-2";}
 if (sostituzione == "Y200V") {valore_pssm = "-2";}
 if (sostituzione == "E218C") {valore_pssm = "-2";}
 if (sostituzione == "E218N") {valore_pssm = "-2";}
 if (sostituzione == "S235P") {valore_pssm = "-2";}
 if (sostituzione == "N272Q") {valore_pssm = "-2";}
 if (sostituzione == "A292Q") {valore_pssm = "-2";}
 if (sostituzione == "S297P") {valore_pssm = "-2";}
 if (sostituzione == "K314I") {valore_pssm = "-2";}
 if (sostituzione == "V316T") {valore_pssm = "-2";}
 if (sostituzione == "L331G") {valore_pssm = "-2";}
 if (sostituzione == "W340I") {valore_pssm = "-2";}
 if (sostituzione == "N355R") {valore_pssm = "-2";}
 if (sostituzione == "Q357T") {valore_pssm = "-2";}
 if (sostituzione == "G361N") {valore_pssm = "-2";}
 if (sostituzione == "A377M") {valore_pssm = "-2";}
 if (sostituzione == "T385C") {valore_pssm = "-2";}
 if (sostituzione == "T385N") {valore_pssm = "-2";}
 if (sostituzione == "T385H") {valore_pssm = "-2";}
 if (sostituzione == "K391T") {valore_pssm = "-2";}
 if (sostituzione == "T400C") {valore_pssm = "-2";}
 if (sostituzione == "T400N") {valore_pssm = "-2";}
 if (sostituzione == "T400H") {valore_pssm = "-2";}
 if (sostituzione == "T412C") {valore_pssm = "-2";}
 if (sostituzione == "T412N") {valore_pssm = "-2";}
 if (sostituzione == "T412H") {valore_pssm = "-2";}
 if (sostituzione == "Q416M") {valore_pssm = "-2";}
 if (sostituzione == "E7A") {valore_pssm = "-2";}
 if (sostituzione == "W24I") {valore_pssm = "-2";}
 if (sostituzione == "G28K") {valore_pssm = "-2";}
 if (sostituzione == "A31Y") {valore_pssm = "-2";}
 if (sostituzione == "D33C") {valore_pssm = "-2";}
 if (sostituzione == "T39V") {valore_pssm = "-2";}
 if (sostituzione == "L120C") {valore_pssm = "-2";}
 if (sostituzione == "V124M") {valore_pssm = "-2";}
 if (sostituzione == "D153C") {valore_pssm = "-2";}
 if (sostituzione == "D161Y") {valore_pssm = "-2";}
 if (sostituzione == "G171R") {valore_pssm = "-2";}
 if (sostituzione == "C174I") {valore_pssm = "-2";}
 if (sostituzione == "Y200I") {valore_pssm = "-2";}
 if (sostituzione == "Y222N") {valore_pssm = "-2";}
 if (sostituzione == "H225N") {valore_pssm = "-2";}
 if (sostituzione == "W226Y") {valore_pssm = "-2";}
 if (sostituzione == "N272T") {valore_pssm = "-2";}
 if (sostituzione == "K308A") {valore_pssm = "-2";}
 if (sostituzione == "I317L") {valore_pssm = "-2";}
 if (sostituzione == "Q321K") {valore_pssm = "-2";}
 if (sostituzione == "V339M") {valore_pssm = "-2";}
 if (sostituzione == "R342Q") {valore_pssm = "-2";}
 if (sostituzione == "A350Q") {valore_pssm = "-2";}
 if (sostituzione == "R392S") {valore_pssm = "-2";}
 if (sostituzione == "M421E") {valore_pssm = "-2";}
 if (sostituzione == "Q422T") {valore_pssm = "-2";}
 if (sostituzione == "M423E") {valore_pssm = "-2";}
 if (sostituzione == "Q2M") {valore_pssm = "-2";}
 if (sostituzione == "L3Q") {valore_pssm = "-2";}
 if (sostituzione == "L10Q") {valore_pssm = "-2";}
 if (sostituzione == "V77S") {valore_pssm = "-2";}
 if (sostituzione == "S78L") {valore_pssm = "-2";}
 if (sostituzione == "W81T") {valore_pssm = "-2";}
 if (sostituzione == "D83Q") {valore_pssm = "-2";}
 if (sostituzione == "L106Y") {valore_pssm = "-2";}
 if (sostituzione == "R112S") {valore_pssm = "-2";}
 if (sostituzione == "K127F") {valore_pssm = "-2";}
 if (sostituzione == "A156K") {valore_pssm = "-2";}
 if (sostituzione == "G163R") {valore_pssm = "-2";}
 if (sostituzione == "P214T") {valore_pssm = "-2";}
 if (sostituzione == "N215M") {valore_pssm = "-2";}
 if (sostituzione == "H225F") {valore_pssm = "-2";}
 if (sostituzione == "K237M") {valore_pssm = "-2";}
 if (sostituzione == "D244G") {valore_pssm = "-2";}
 if (sostituzione == "T282L") {valore_pssm = "-2";}
 if (sostituzione == "A291Y") {valore_pssm = "-2";}
 if (sostituzione == "L300K") {valore_pssm = "-2";}
 if (sostituzione == "L300H") {valore_pssm = "-2";}
 if (sostituzione == "G360V") {valore_pssm = "-2";}
 if (sostituzione == "A370M") {valore_pssm = "-2";}
 if (sostituzione == "V376Q") {valore_pssm = "-2";}
 if (sostituzione == "L387Q") {valore_pssm = "-2";}
 if (sostituzione == "L388Q") {valore_pssm = "-2";}
 if (sostituzione == "L394Q") {valore_pssm = "-2";}
 if (sostituzione == "L403Q") {valore_pssm = "-2";}
 if (sostituzione == "T410L") {valore_pssm = "-2";}
 if (sostituzione == "L414Q") {valore_pssm = "-2";}
 if (sostituzione == "L417Q") {valore_pssm = "-2";}
 if (sostituzione == "H9Q") {valore_pssm = "-2";}
 if (sostituzione == "D25F") {valore_pssm = "-2";}
 if (sostituzione == "D33M") {valore_pssm = "-2";}
 if (sostituzione == "N34K") {valore_pssm = "-2";}
 if (sostituzione == "R38M") {valore_pssm = "-2";}
 if (sostituzione == "I64T") {valore_pssm = "-2";}
 if (sostituzione == "E79Y") {valore_pssm = "-2";}
 if (sostituzione == "E103F") {valore_pssm = "-2";}
 if (sostituzione == "V124T") {valore_pssm = "-2";}
 if (sostituzione == "D136N") {valore_pssm = "-2";}
 if (sostituzione == "F149A") {valore_pssm = "-2";}
 if (sostituzione == "A156I") {valore_pssm = "-2";}
 if (sostituzione == "A156C") {valore_pssm = "-2";}
 if (sostituzione == "G163A") {valore_pssm = "-2";}
 if (sostituzione == "G163E") {valore_pssm = "-2";}
 if (sostituzione == "K168Q") {valore_pssm = "-2";}
 if (sostituzione == "M187L") {valore_pssm = "-2";}
 if (sostituzione == "A190G") {valore_pssm = "-2";}
 if (sostituzione == "V199G") {valore_pssm = "-2";}
 if (sostituzione == "N224K") {valore_pssm = "-2";}
 if (sostituzione == "N272D") {valore_pssm = "-2";}
 if (sostituzione == "Q279N") {valore_pssm = "-2";}
 if (sostituzione == "F295A") {valore_pssm = "-2";}
 if (sostituzione == "K314C") {valore_pssm = "-2";}
 if (sostituzione == "A318P") {valore_pssm = "-2";}
 if (sostituzione == "A318I") {valore_pssm = "-2";}
 if (sostituzione == "Q327H") {valore_pssm = "-2";}
 if (sostituzione == "G334I") {valore_pssm = "-2";}
 if (sostituzione == "P343S") {valore_pssm = "-2";}
 if (sostituzione == "V351F") {valore_pssm = "-2";}
 if (sostituzione == "M353N") {valore_pssm = "-2";}
 if (sostituzione == "I354D") {valore_pssm = "-2";}
 if (sostituzione == "K393M") {valore_pssm = "-2";}
 if (sostituzione == "H406Q") {valore_pssm = "-2";}
 if (sostituzione == "L425E") {valore_pssm = "-2";}
 if (sostituzione == "L428E") {valore_pssm = "-2";}
 if (sostituzione == "W24C") {valore_pssm = "-2";}
 if (sostituzione == "M70Q") {valore_pssm = "-2";}
 if (sostituzione == "E79C") {valore_pssm = "-2";}
 if (sostituzione == "D101R") {valore_pssm = "-2";}
 if (sostituzione == "P110A") {valore_pssm = "-2";}
 if (sostituzione == "L129T") {valore_pssm = "-2";}
 if (sostituzione == "T141S") {valore_pssm = "-2";}
 if (sostituzione == "G144A") {valore_pssm = "-2";}
 if (sostituzione == "P146V") {valore_pssm = "-2";}
 if (sostituzione == "Y151A") {valore_pssm = "-2";}
 if (sostituzione == "A156G") {valore_pssm = "-2";}
 if (sostituzione == "T158S") {valore_pssm = "-2";}
 if (sostituzione == "M187I") {valore_pssm = "-2";}
 if (sostituzione == "A190E") {valore_pssm = "-2";}
 if (sostituzione == "E203V") {valore_pssm = "-2";}
 if (sostituzione == "F211N") {valore_pssm = "-2";}
 if (sostituzione == "T217I") {valore_pssm = "-2";}
 if (sostituzione == "T282Y") {valore_pssm = "-2";}
 if (sostituzione == "Q327R") {valore_pssm = "-2";}
 if (sostituzione == "G328S") {valore_pssm = "-2";}
 if (sostituzione == "Y329G") {valore_pssm = "-2";}
 if (sostituzione == "R332I") {valore_pssm = "-2";}
 if (sostituzione == "F337P") {valore_pssm = "-2";}
 if (sostituzione == "I367D") {valore_pssm = "-2";}
 if (sostituzione == "A370L") {valore_pssm = "-2";}
 if (sostituzione == "L372A") {valore_pssm = "-2";}
 if (sostituzione == "L372R") {valore_pssm = "-2";}
 if (sostituzione == "T410Y") {valore_pssm = "-2";}
 if (sostituzione == "Q422S") {valore_pssm = "-2";}
 if (sostituzione == "S424M") {valore_pssm = "-2";}
 if (sostituzione == "V22E") {valore_pssm = "-2";}
 if (sostituzione == "N34D") {valore_pssm = "-2";}
 if (sostituzione == "W47Y") {valore_pssm = "-2";}
 if (sostituzione == "M72A") {valore_pssm = "-2";}
 if (sostituzione == "W95Y") {valore_pssm = "-2";}
 if (sostituzione == "A108L") {valore_pssm = "-2";}
 if (sostituzione == "F113Y") {valore_pssm = "-2";}
 if (sostituzione == "S148G") {valore_pssm = "-2";}
 if (sostituzione == "F149K") {valore_pssm = "-2";}
 if (sostituzione == "W162Y") {valore_pssm = "-2";}
 if (sostituzione == "L166W") {valore_pssm = "-2";}
 if (sostituzione == "K168E") {valore_pssm = "-2";}
 if (sostituzione == "Q212I") {valore_pssm = "-2";}
 if (sostituzione == "K213G") {valore_pssm = "-2";}
 if (sostituzione == "K213P") {valore_pssm = "-2";}
 if (sostituzione == "N215Y") {valore_pssm = "-2";}
 if (sostituzione == "N224D") {valore_pssm = "-2";}
 if (sostituzione == "N228G") {valore_pssm = "-2";}
 if (sostituzione == "W245P") {valore_pssm = "-2";}
 if (sostituzione == "G261R") {valore_pssm = "-2";}
 if (sostituzione == "N272R") {valore_pssm = "-2";}
 if (sostituzione == "V281N") {valore_pssm = "-2";}
 if (sostituzione == "L286H") {valore_pssm = "-2";}
 if (sostituzione == "W287Y") {valore_pssm = "-2";}
 if (sostituzione == "A291L") {valore_pssm = "-2";}
 if (sostituzione == "F295H") {valore_pssm = "-2";}
 if (sostituzione == "D299H") {valore_pssm = "-2";}
 if (sostituzione == "L300R") {valore_pssm = "-2";}
 if (sostituzione == "Y329C") {valore_pssm = "-2";}
 if (sostituzione == "V339F") {valore_pssm = "-2";}
 if (sostituzione == "A350R") {valore_pssm = "-2";}
 if (sostituzione == "R363G") {valore_pssm = "-2";}
 if (sostituzione == "V369E") {valore_pssm = "-2";}
 if (sostituzione == "A370D") {valore_pssm = "-2";}
 if (sostituzione == "L372H") {valore_pssm = "-2";}
 if (sostituzione == "K374D") {valore_pssm = "-2";}
 if (sostituzione == "V376H") {valore_pssm = "-2";}
 if (sostituzione == "R4A") {valore_pssm = "-2";}
 if (sostituzione == "E7P") {valore_pssm = "-2";}
 if (sostituzione == "G11Y") {valore_pssm = "-2";}
 if (sostituzione == "V22R") {valore_pssm = "-2";}
 if (sostituzione == "I26Y") {valore_pssm = "-2";}
 if (sostituzione == "N34E") {valore_pssm = "-2";}
 if (sostituzione == "M42L") {valore_pssm = "-2";}
 if (sostituzione == "E58M") {valore_pssm = "-2";}
 if (sostituzione == "E71R") {valore_pssm = "-2";}
 if (sostituzione == "A73G") {valore_pssm = "-2";}
 if (sostituzione == "A73K") {valore_pssm = "-2";}
 if (sostituzione == "D83N") {valore_pssm = "-2";}
 if (sostituzione == "L89S") {valore_pssm = "-2";}
 if (sostituzione == "A121G") {valore_pssm = "-2";}
 if (sostituzione == "A121K") {valore_pssm = "-2";}
 if (sostituzione == "S126I") {valore_pssm = "-2";}
 if (sostituzione == "D136K") {valore_pssm = "-2";}
 if (sostituzione == "A160G") {valore_pssm = "-2";}
 if (sostituzione == "A160K") {valore_pssm = "-2";}
 if (sostituzione == "S201R") {valore_pssm = "-2";}
 if (sostituzione == "E203G") {valore_pssm = "-2";}
 if (sostituzione == "Q212M") {valore_pssm = "-2";}
 if (sostituzione == "T217Q") {valore_pssm = "-2";}
 if (sostituzione == "T217R") {valore_pssm = "-2";}
 if (sostituzione == "N224S") {valore_pssm = "-2";}
 if (sostituzione == "N224E") {valore_pssm = "-2";}
 if (sostituzione == "D234N") {valore_pssm = "-2";}
 if (sostituzione == "S238R") {valore_pssm = "-2";}
 if (sostituzione == "I253S") {valore_pssm = "-2";}
 if (sostituzione == "A257I") {valore_pssm = "-2";}
 if (sostituzione == "S276G") {valore_pssm = "-2";}
 if (sostituzione == "V281Y") {valore_pssm = "-2";}
 if (sostituzione == "L286A") {valore_pssm = "-2";}
 if (sostituzione == "A288P") {valore_pssm = "-2";}
 if (sostituzione == "L311C") {valore_pssm = "-2";}
 if (sostituzione == "R332F") {valore_pssm = "-2";}
 if (sostituzione == "D335V") {valore_pssm = "-2";}
 if (sostituzione == "Q357S") {valore_pssm = "-2";}
 if (sostituzione == "E358M") {valore_pssm = "-2";}
 if (sostituzione == "T366C") {valore_pssm = "-2";}
 if (sostituzione == "I367G") {valore_pssm = "-2";}
 if (sostituzione == "K426A") {valore_pssm = "-2";}
 if (sostituzione == "A20M") {valore_pssm = "-2";}
 if (sostituzione == "G28H") {valore_pssm = "-2";}
 if (sostituzione == "L32G") {valore_pssm = "-2";}
 if (sostituzione == "M42I") {valore_pssm = "-2";}
 if (sostituzione == "P60C") {valore_pssm = "-2";}
 if (sostituzione == "E66K") {valore_pssm = "-2";}
 if (sostituzione == "L68A") {valore_pssm = "-2";}
 if (sostituzione == "Q111Y") {valore_pssm = "-2";}
 if (sostituzione == "V124F") {valore_pssm = "-2";}
 if (sostituzione == "T141V") {valore_pssm = "-2";}
 if (sostituzione == "I154G") {valore_pssm = "-2";}
 if (sostituzione == "A156E") {valore_pssm = "-2";}
 if (sostituzione == "T158V") {valore_pssm = "-2";}
 if (sostituzione == "M187V") {valore_pssm = "-2";}
 if (sostituzione == "S201N") {valore_pssm = "-2";}
 if (sostituzione == "Q212F") {valore_pssm = "-2";}
 if (sostituzione == "E218G") {valore_pssm = "-2";}
 if (sostituzione == "S235I") {valore_pssm = "-2";}
[truncated: 247,029 more chars]
