# Supplementary figures and images for: E-Learning for Rare Diseases: An Example Using Fabry Disease
Source: Int J Mol Sci. 2017 Sep 24;18(10):2049. doi: 10.3390/ijms18102049 (PMC5666731; doi:10.3390/ijms18102049)

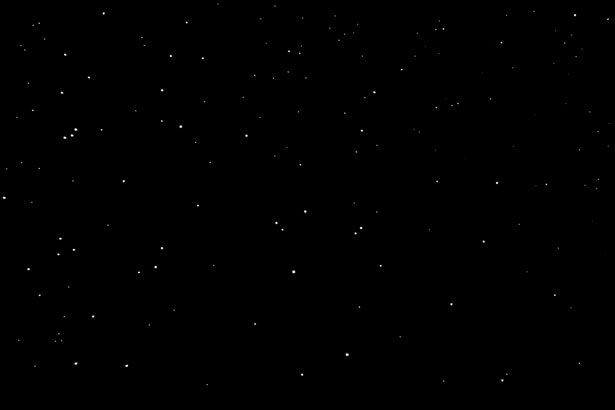

Supplement: Supplementary File 1 [file ijms-18-02049-s001.zip › sito_fabry_2.3/bg.jpg]

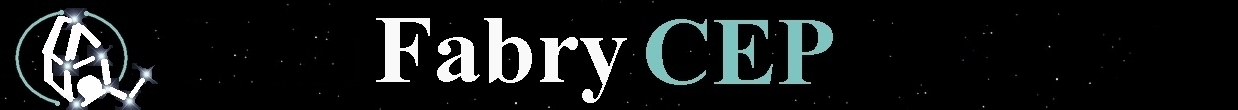

Supplement: Supplementary File 1 [file ijms-18-02049-s001.zip › sito_fabry_2.3/cep.jpg]

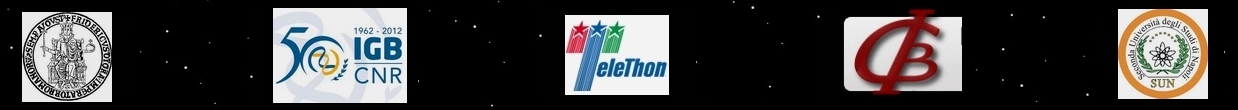

Supplement: Supplementary File 1 [file ijms-18-02049-s001.zip › sito_fabry_2.3/end.jpg]

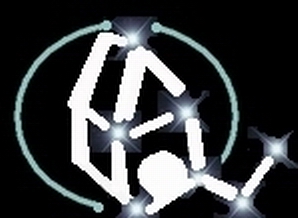

Supplement: Supplementary File 1 [file ijms-18-02049-s001.zip › sito_fabry_2.3/icon.jpg]

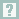

Supplement: Supplementary File 1 [file ijms-18-02049-s001.zip › sito_fabry_2.3/info.png]
